# N_2_H_4_ as traceless mediator for homo- and cross- aryl coupling

**DOI:** 10.1038/s41467-018-07198-7

**Published:** 2018-11-09

**Authors:** Leiyang Lv, Zihang Qiu, Jianbin Li, Mingxin Liu, Chao-Jun Li

**Affiliations:** 1 Department of Chemistry and FRQNT Center for Green Chemistry and Catalysis, McGill University, 801 Sherbrooke Street West, Montreal, QC H3A 0B8 Canada; 2 State Key Laboratory of Applied Organic Chemistry, Lanzhou University, 222 Tianshui Road, Lanzhou, Gansu 730000 China

## Abstract

Transition-metal catalyzed couplings of aryl halides or arenes with aryl organometallics, as well as direct reductive coupling of two aryl halides, are the predominant methods to synthesize biaryls. However, stoichiometric amounts of metals are inevitably utilized in these reactions, either in the pre-generation of organometallic reagents or acting as reductant in situ, thus producing quantitative metal waste. Herein, we demonstrate that this longstanding challenge can be overcome with N_2_H_4_ as a metal surrogate. The fundamental innovation of this strategy is that N_2_ and H_2_ are generated as side products, which readily escape from the system after the reaction. The success of both homo- and cross-coupling of various aryl electrophiles bearing a wide range of functional groups manifests the powerfulness and versatility of this strategy. Furthermore, both homo- and cross-couplings of a series of alkaloids, amino acids and steroids exemplify application of this protocol in the functionalization of biologically active molecules.

## Abstract (web summary)

Synthesis of biaryls by coupling strategies is unavoidably accompanied by stoichiometric metal waste. Here, the authors report the homo- and cross-electrophile coupling by using hydrazine as metal surrogate, only liberating N_2_ and H_2_ as side products.

## Introduction

**Fig. 1:**
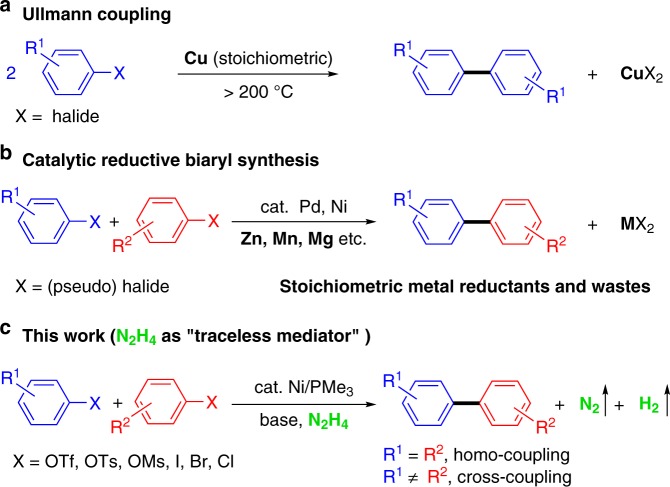
Reductive homo- and cross-coupling of aryl electrophiles to synthesize biaryl structures. **a** Traditional Ullmann coupling with stoichiometric copper under high temperature; **b** Pd or Ni catalyzed Ullmann coupling with stoichiometric metals and wastes; **c** N_2_H_4_ as traceless mediator for homo- and cross-aryl coupling

The development of efficient and practical method to prepare biaryl skeleton is particularly important in modern synthetic chemistry, due to its broad applications in a wide range of fields such as pharmaceuticals, agrochemicals, pigments, natural products and polymers^1–4^. The past few decades have witnessed extraordinary progress in the transition-metal catalyzed cross-coupling strategies^5–8^, in which the direct reductive coupling of aryl halides in situ (without prior preparation of organometallic reagents) is one of the most convenient and rapid approaches for the preparation of biaryl motifs and interlocked molecules. In 1901, Ullmann reported the first homo-coupling of aryl halides to synthesize symmetrical biaryls in the presence of stoichiometric amount of copper under high temperature (>200 °C) (Fig. 1a)^9^. Inspired by this pioneering work, various modified Ullmann-type coupling reactions have been developed, including the formation of C–C, C–N, and C–O bonds^10,11^. The utilization of catalytic Pd or Ni complexes in the presence of metal reductant such as Zn, Mn, and Mg is of particular importance, making the reductive Ullmann coupling occur under much milder conditions (Fig. 1b)^12^. Furthermore, such modifications allow less reactive pseudo-halides to act as suitable cross-coupling partners via C–O bond cleavage^13–17^. Notably, in 2015 Weix and co-workers made a ground-breaking advancement in the cross-Ullmann reaction using a Pd/Ni bi-metallic synergistic strategy with Zn as the sacrificial reductant^18^. Nevertheless, stoichiometric quantities of metals, such as Zn, Mn, Mg, etc., were essential reductants in the reported literatures, thus producing extra metal waste. This issue is particularly problematic in the large-scale experiment, not only complicating the synthetic operations but also raising environmental concerns. Besides, the metal reductant often needs to be activated prior to use, otherwise lower yields and side reactions were obtained.

Inspired by the recent work reported by our group, namely simple hydrazones acting as carbanion equivalents instead of organometallic reagents in the catalytic nucleophilic additions^19^, we postulate to use N_2_H_4_ as a traceless metal surrogate to overcome those troublesome limitations in homo- and cross-electrophile coupling reactions^20–23^ (Fig. 1c). As the highlight of this strategy, N_2_ and H_2_ are generated as readily escapable side products, thus making the coupling reactions dramatically clean and easy to handle.

## Results

### Rational design

To realize this hypothesis, several challenges must be addressed. Firstly, the relative reducing ability of hydrazine and metal catalyst should be carefully balanced. As shown in the literature, N_2_H_4_ has a strong negative reduction potential (*E*^*θ*^ = −1.49 V or −1.16 V)^24^ vs. Ni^2+^/Ni^0^ (*E*^*θ*^ = −0.25 V)^25^. This suggests that N_2_H_4_ has the capacity to reduce the high-valent Ni complex to generate catalytically active Ni(0) species (though the relative potential value may vary in organic solvents). Secondly, N_2_H_4_ readily forms very stable complexes with transition metal catalysts, which are generally inert for further reactions. The use of a suitable ligand, which displays stronger affinity toward metal catalyst over N_2_H_4_, may be a solution to maintain the activity of the metal catalyst^26^. Thirdly, it is well known that transition metal catalysts including Pd and Ni can insert into the C–X/C–O bonds of aryl electrophiles. This facilitates the hydro-dehalogenation/desulfonylation of aryl electrophiles to generate arenes, especially in the presence of active N–H bonds in hydrazine^27^. Considering the alkalinity of hydrazine (pK_b_ = 5.89)^28^, the employment of an appropriate catalytic system with elaborately selected base would be crucial to achieve selective formation of the homo-coupling product over competing reduction.

### Screening of reaction conditions

To start the research, *p*-tolyl tosylate (**1a**) and hydrazine (**2a**) were selected as the model substrates to explore the reaction conditions (Table 1). To our delight, the homo-coupling product **3a** was obtained in 55% yield when the reaction was carried out in 1,4-dioxane with 10 mol % of Ni(cod)_2_ as the catalyst and 20 mol % of PMe_3_ as the ligand at 110 °C (entry 1). Intriguingly, ligand investigations revealed that other mono-dentate P-ligands and bi-dentate P- or N-ligands were ineffective for this transformation (entries 2-4). This is possibly because that the competing formation of stable complexes of N_2_H_4_ with Ni deactivated the metal catalyst. Increasing the amount of PMe_3_ to 40 mol% gives **3a** in 91% yield (entry 5). Changing the solvent to toluene or THF did not improve the efficiency of this transformation (entries 6 and 7), while the commonly used DMF or DMAc in the reductive coupling afforded no desired product (entries 8 and 9). Gratifyingly, the more convenient Ni(II) pre-catalysts also facilitate this homo-coupling reaction, and moderate to good yields of **3a** were obtained (entries 10–12). Different catalyst precursors, such as Pd(OAc)_2_, Fe(acac)_3_, CuCl_2_, CoCl_2_ were also examined, yet they were all inactive and the starting material was recovered quantitatively (entries 13–16). It should be noteworthy that the choice of base also has a great influence on the reaction efficiency^29^. For example, **3a** was obtained in only 10% yield without adding a base (entry 17). Strong bases, such as ^*t*^BuOK and ^*t*^BuOLi, delivered toluene exclusively as the hydro-desulfonylation product (entries 18 and 19). Both **3a** and toluene were obtained without any selectivity when LiOH, NaOH and Cs_2_CO_3_ were used as bases (entries 20–22). Besides, other bases were less effective in this transformation (entries 23–26). **3a** was obtained in 57% yield when 5 mol % of Ni(cod)_2_ was used, accompanied with 40% of competing toluene formation. Control experiments showed that in absence of a catalyst or ligand, the homo-coupling product **3a** was not observed (entries 28 and 29).

**Table 1:** Optimization of the reaction conditions


Entry	Catalyst	Ligand	Base	3a (%)^a^
1^b^	Ni(cod)_2_	PMe_3_	K_3_PO_4_	55
2	Ni(cod)_2_	PPh_3_	K_3_PO_4_	0
3	Ni(cod)_2_	PCy_3_	K_3_PO_4_	0
4	Ni(cod)_2_	bi-dentates^*c*^	K_3_PO_4_	0
5	Ni(cod)_2_	PMe_3_	K_3_PO_4_	91 (88)
6^d^	Ni(cod)_2_	PMe_3_	K_3_PO_4_	75
7^*e*^	Ni(cod)_2_	PMe_3_	K_3_PO_4_	64
8^f^	Ni(cod)_2_	PMe_3_	K_3_PO_4_	0
9^g^	Ni(cod)_2_	PMe_3_	K_3_PO_4_	0
10	NiCl_2_	PMe_3_	K_3_PO_4_	76
11	NiBr_2_•glyme	PMe_3_	K_3_PO_4_	71
12	Ni(acac)_2_	PMe_3_	K_3_PO_4_	42
13	Pd(OAc)_2_	PMe_3_	K_3_PO_4_	0
14	Fe(acac)_3_	PMe_3_	K_3_PO_4_	0
15	CuCl_2_	PMe_3_	K_3_PO_4_	0
16	CoCl_2_	PMe_3_	K_3_PO_4_	0
17	Ni(cod)_2_	PMe_3_	—	10
18	Ni(cod)_2_	PMe_3_	^*t*^BuOK	0
19	Ni(cod)_2_	PMe_3_	^*t*^BuOLi	0
20	Ni(cod)_2_	PMe_3_	NaOH	61
21	Ni(cod)_2_	PMe_3_	LiOH	40
22	Ni(cod)_2_	PMe_3_	Cs_2_CO_3_	52
23	Ni(cod)_2_	PMe_3_	CsF	0
24	Ni(cod)_2_	PMe_3_	K_2_CO_3_	29
25	Ni(cod)_2_	PMe_3_	Et_3_N	10
26	Ni(cod)_2_	PMe_3_	DABCO	22
27^b,h^	Ni(cod)_2_	PMe_3_	K_3_PO_4_	57
28	Ni(cod)_2_	—	K_3_PO_4_	0
29	—	PMe_3_	K_3_PO_4_	0

Reaction conditions: **1a** (0.2 mmol), N_2_H_4_ (0.1 mmol), [Ni] (10 mol%), ligand (40 mol% for PMe_3_, 20 mol% for other monodentate ligand, 10 mol% for bidentate ligand), base (0.6 mmol), 1,4-dioxane (1.0 mL), 110 °C, 12 h under N_2_ unless otherwise noted

^a^Yields were determined by crude ^1^H NMR using mesitylene as an internal standard (isolated one in parenthesis)

^b^20 mol% PMe_3_

^c^dppe, dppp, dppb, dcype, Xantphos, DPEphos, *rac*-BINAP, bipy, 1,10-phenanthroline were tested, respectively

^d^PhMe as solvent

^e^THF as solvent

^f^DMF as solvent

^*g*^ DMAc as solvent

^h^[Ni] (5 mol%)

**Fig. 2:**
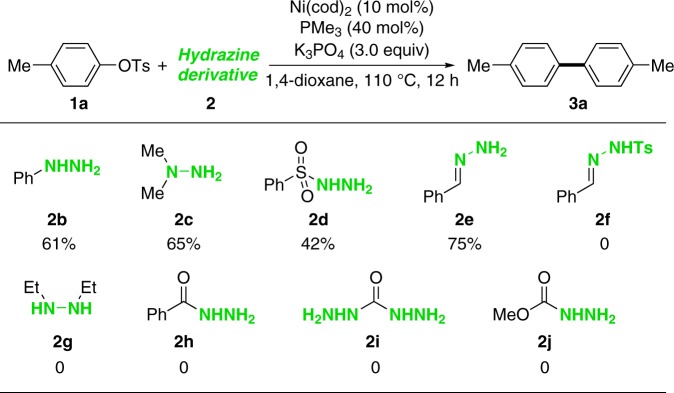
Exploration of hydrazine derivatives. Reaction conditions: **1a** (0.2 mmol), **2** (0.2 mmol), Ni(cod)_2_ (10 mol%), PMe_3_ (40 mol%), K_3_PO_4_ (0.6 mmol), 1,4-dioxane (1.0 mL), 110 °C, 12 h under N_2_. Yields were determined by crude ^1^H NMR using mesitylene as an internal standard

We then examined the feasibility of the homo-coupling reaction with other hydrazine derivatives as reductants. As shown in Fig. 2, phenyl hydrazine (**2b**), 1,1-dimethylhydrazine (**2c**), sulfonohydrazide (**2d**) and hydrazone (**2e**) can also serve as the sacrificial reductants, but all lead to lower productivity. Other hydrazine derivatives, such as *N*-Ts hydrazone (**2f**), 1,2-diethylhydrazine (**2g**) and hydrazides (**2h–j**) were totally inactive.

### Scope of the Ullmann coupling

**Fig. 3:**
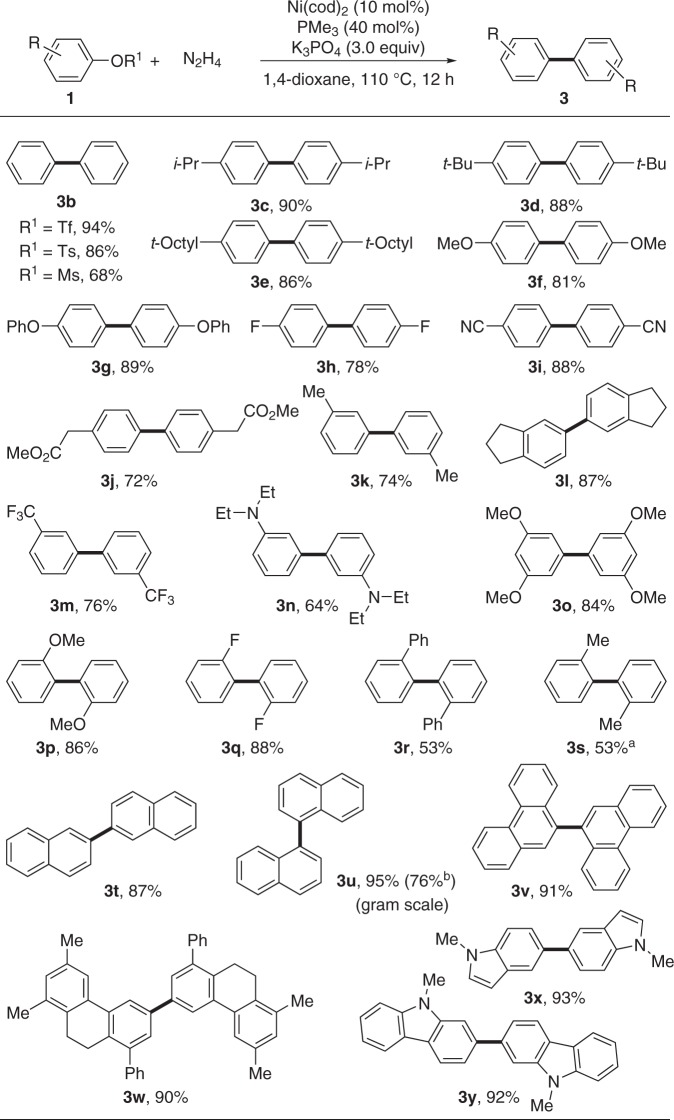
Scope of aryl tosylates **1**. Reaction conditions: **1** (0.2 mmol), N_2_H_4_ (0.1 mmol), Ni(cod)_2_ (10 mol%), PMe_3_ (40 mol%), K_3_PO_4_ (0.6 mmol), 1,4-dioxane (1.0 mL), 110 °C, 12 h under N_2._. Reported yields are the isolated ones. ^a^The reaction was carried out at 130 °C. ^b^The reaction was carried out on 10 mmol scale, and **3u** was obtained in 0.96 g

With the optimized conditions identified, the scope of the phenol derivatives was subsequently investigated (Fig. 3). Aryl tosylate, triflate, and mesylate were all homo-coupled efficiently to give the desired biaryl **3b**, and the yields were directly proportional to their reactivity order. Tosylates **1** with electron-donating (-*i-*Pr, -*t-*Bu, -*t-*Octyl, -OMe, -OPh, etc.) and electron-withdrawing (-F, -CN) groups on the *para*-position of phenyl ring all proceeded smoothly to give the corresponding homo-coupling products **3c–i** in good yields. The sensitive ester group (-CO_2_Me) was compatible under the standard conditions (**3j**). Slightly lower yields were observed (**3k–o**) with *meta*-substituted phenyl tosylates.

This catalytic homo-coupling reaction was found to be sensitive to the steric environment adjacent to the active site of the substrates. For example, the homo-coupling products **3p** and **3q** were obtained in good yields with less-hindered -OMe or -F at the *ortho-*position of the aryl tosylates, while only 53% yield of the desired **3r** was obtained in the presence of an *ortho-* phenyl group. In the case of *ortho*- methyl group, increased temperature was necessary to overcome the steric hindrance (**3s**). Gratifyingly, this method allowed the synthesis of sterically demanding 1,1′-binaphthalene and 2,2′-binaphthalene derivatives **3t** and **3u** efficiently. Gram-scale synthesis of **3u** was performed to highlight the practicality of this method. Further expansion of the *π* conjugation to phenanthrene tosylate maintained its excellent reactivity (**3v** and **3w**). These valuable structures may find wide applications in material sciences. Besides, the homo-dimerization of alkaloids, such as indole and carbazole, proceeded smoothly and the corresponding products (**3×** and **3y**) were obtained in 93% and 92% yields, respectively.

**Fig. 4:**
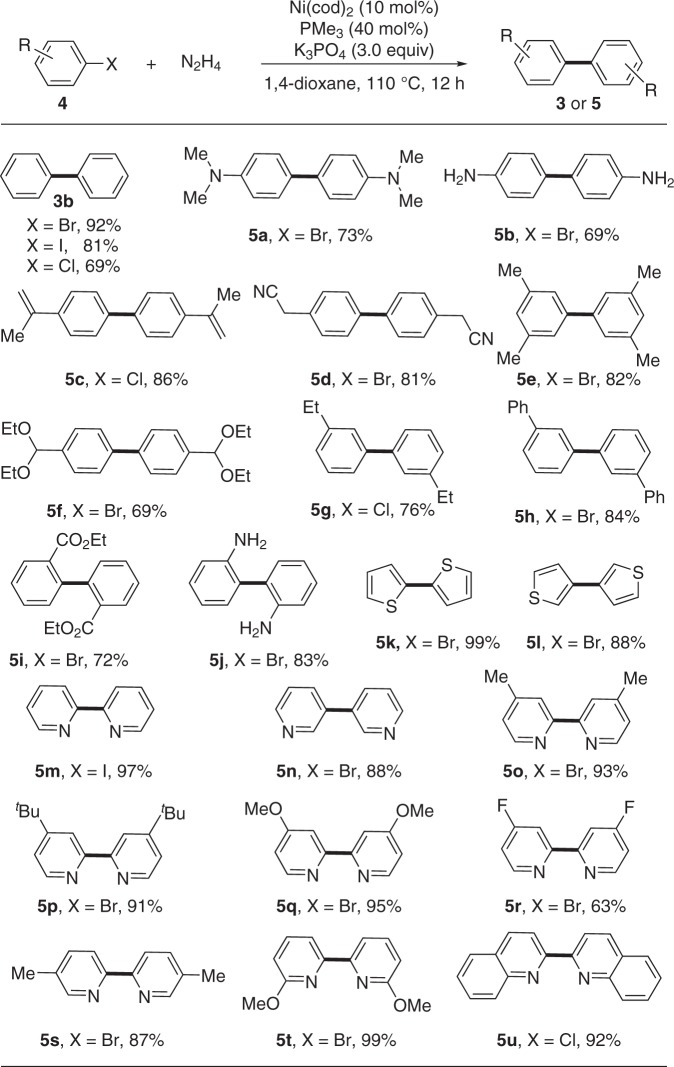
Scope of aryl halides **4**. Reaction conditions: **4** (0.2 mmol), N_2_H_4_ (0.1 mmol), Ni(cod)_2_ (10 mol%), PMe_3_ (40 mol%), K_3_PO_4_ (0.6 mmol), 1,4-dioxane (1.0 mL), 110 °C, 12 h under N_2._. Reported yields are the isolated ones

To our delight, this protocol also worked well for the reductive homo-coupling of aryl halides, which are low-cost and abundant from petrochemical industry (Fig. 4). For example, aryl bromide, iodide and even less reactive chloride could readily undergo the dimerization to give the desired product **3b** efficiently. Besides, a broad range of sensitive functional groups, including tertiary amine (**5a**), *ortho-* and *para-* free amines (**5j** and **5b**), alkene (**5c**), nitrile (**5d**), acetal (**5f**) and ester (**5i**), were all compatible and left intact under the standard conditions. The *meta*-substituted aryl halides delivered the desired products (**5e**, **5g**, and **5h**) in good yields. Notably, both 2- and 3-bomothiophenes could also serve as competent coupling partners (**5k** and **5l**). Electron-rich *N*-heterocycles, which are frequently problematic with transition metal catalyst due to their strong coordination capability, reacted efficiently with this protocol. The resulted various kinds of bi-pyridines **5m–t** and bi-quinoline **5u** are very powerful ligands in metal-catalyzed transformations.

**Fig. 5:**
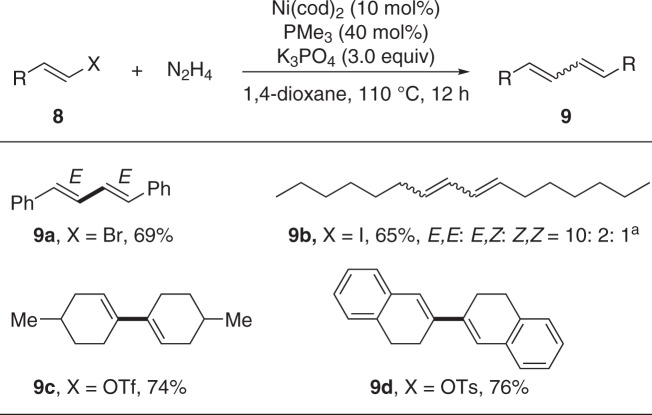
Homo-coupling of vinyl electrophiles to build conjugated dienes. Reaction conditions: **8** (0.2 mmol), N_2_H_4_ (0.1 mmol), Ni(cod)_2_ (10 mol%), PMe_3_ (40 mol%), K_3_PO_4_ (0.6 mmol), 1,4-dioxane (1.0 mL), 110 °C, 12 h under N_2_. Reported yields are the isolated ones. ^a^The *E/Z* ratio was determined by GC analysis

Of particular note, this strategy could also be extended to the intra-molecular reductive coupling. The reductive cyclization of dibromo **6** gave the desired 9,10-dihydrophenanthrene (**7**) in 51% yield (see Supplementary Figure 2). Furthermore, both linear and cyclic conjugated dienes **9a–9d** were prepared efficiently when vinyl electrophiles **8** were applied under the standard conditions, thus expanding the coupling precursors to the abundant alkenes and ketones (Fig. 5).

### Scope of the cross-Ullmann reaction

**Fig. 6:**
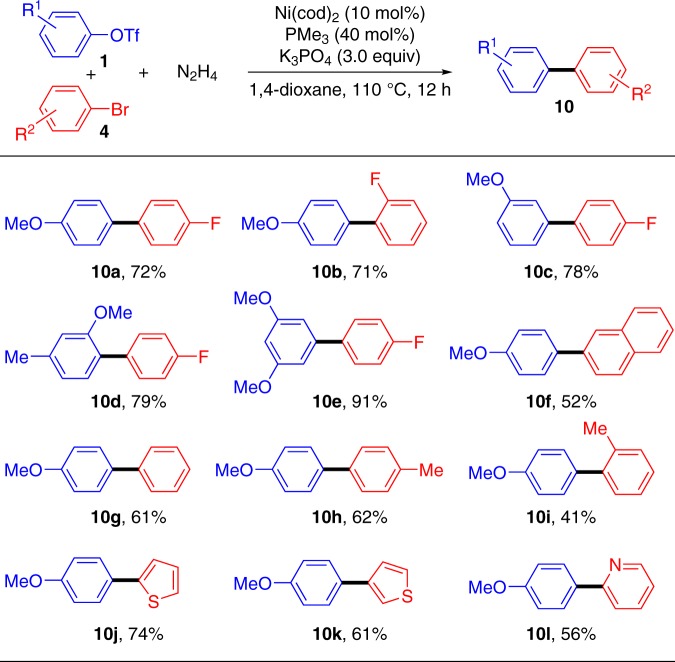
Cross-coupling between aryl triflates **1** and bromides **4**. Reaction conditions: **1** (0.1 mmol), **4** (0.3 mmol), N_2_H_4_ (0.2 mmol), Ni(cod)_2_ (10 mol%), PMe_3_ (40 mol%), K_3_PO_4_ (0.6 mmol), 1,4-dioxane (1.0 mL), 110 °C, 12 h under N_2_. Reported yields are the isolated ones

Given the success of homo-couplings using hydrazine as the traceless mediator, we turned our attention to the cross-Ullmann reaction. Aryl triflates and aryl bromides were selected as the suitable cross-coupling partners, and the results were summarized in Fig. 6. Excellent yields of cross-coupled products **10a–10e** were obtained between electron-rich aryl triflates and electron-deficient fluoro-aryl bromides. Moderate yields of the desired products **10f–10h** were achieved when more electron-neutral bromides were applied instead. Steric hindrance on the *ortho*-position of aryl ring has a negative effect on the yield (**10i**). Moreover, heterocyclic bromides, such as 2-bromothiophene, 3-bromothiophene, and 2-bromopyridine, were all proved to be suitable cross-coupling partners to react with aryl triflates efficiently (**10j–10l**).

**Fig. 7:**
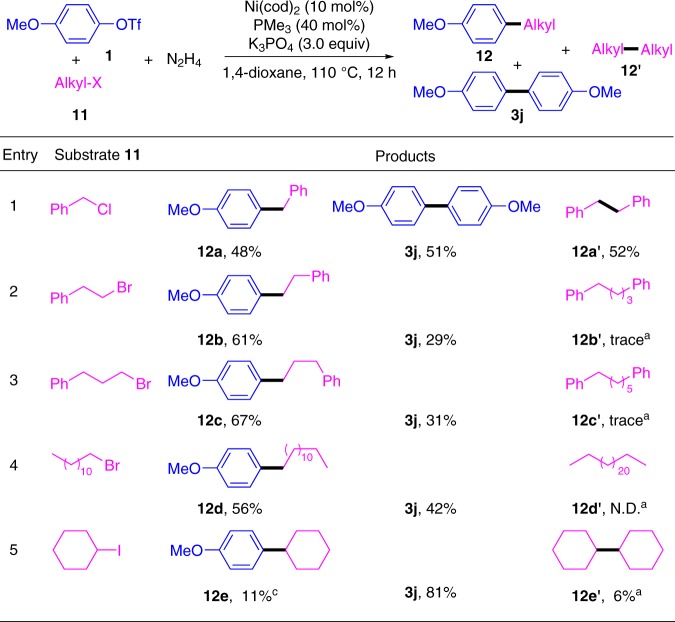
Cross-coupling between aryl triflates **1** and alkyl halides **11**. Reaction conditions: **1** (0.1 mmol), **11** (0.3 mmol), N_2_H_4_ (0.2 mmol), Ni(cod)_2_ (10 mol%), PMe_3_ (40 mol%), K_3_PO_4_ (0.6 mmol), 1,4-dioxane (1.0 mL), 110 °C, 12 h under N_2_. Reported yields are the isolated ones. ^a^The yield was determined by GC

Encouraged by these results, we further investigated C(*sp*^2^)-C(*sp*^3^) cross-coupling with this protocol^30–34^. The preliminary results were summarized in Fig. 7. The cross-coupling of aryl triflates **1** with primary alkyl halides, such as benzyl chloride (**11a**), (2-bromoethyl) benzene (**11b**), (3-bromoethyl) benzene (**11c**) and 1-bromododecane (**11d**), gave the desired alkylated benzenes **12a–d** in moderate yields. In the case of cyclohexyl iodide, lower yield of **12e** was obtained under the same conditions.

### Synthetic applications

**Fig. 8:**
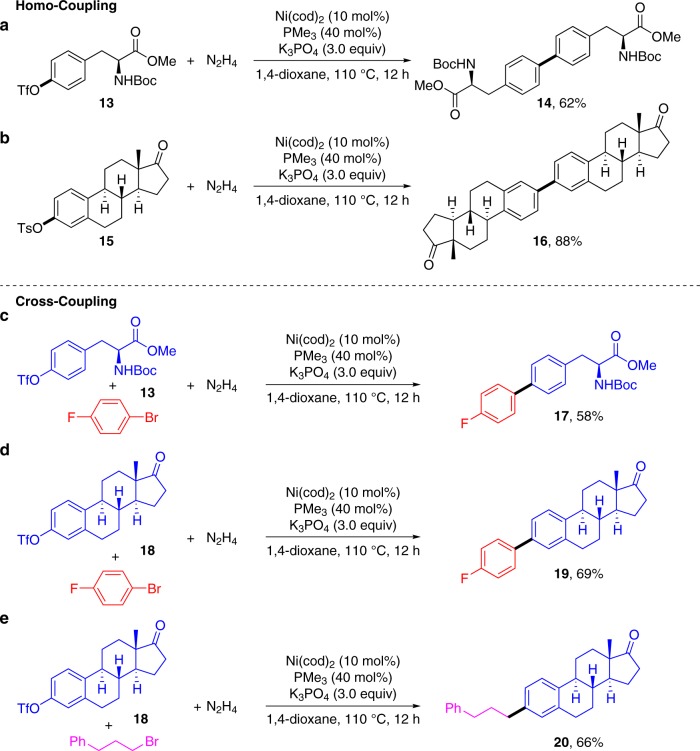
Applications in the homo- and cross-coupling of bioactive molecules. **a** Homo-coupling of tyrosine triflate; **b** Homo-coupling of estrone tosylate; **c** cross-coupling of tyrosine triflate with 1-bromo-4-fluorobenzene; **d** cross- coupling of estrone tosylate with 1-bromo-4-fluorobenzene; **e** Cross-coupling of estrone triflate with (3-bromopropyl)benzene

To evaluate the synthetic potentials of both the homo- and cross-coupling, bioactive molecules with sensitive functionalities, such as ketone, ester and carbamate, were examined. The homo-coupling of tyrosine (**13**) and estrone (**15**) derivatives (Fig. 8a, b) gave the dimerized products efficiently. As described in the literature, linking up two natural product monomers together has a dramatic influence on the properties of the obtained dimers^35^. Furthermore, the cross-coupling of the two bioactive molecules **13** and **18** with 1-bromo-4-fluorobenzene also proceeded smoothly, delivering the desired products **17** and **19** in 58% and 69% yields, respectively (Fig. 8c, d). Moreover, the alkylated cross-coupling product of estrone **20** was synthesized efficiently when **18** and (3-bromopropyl) benzene were subjected to the standard conditions (Fig. 8e). These results exemplified the utility and generality of our protocol in the late stage functionalization of complex molecules.

### Mechanistic investigation

**Fig. 9:**
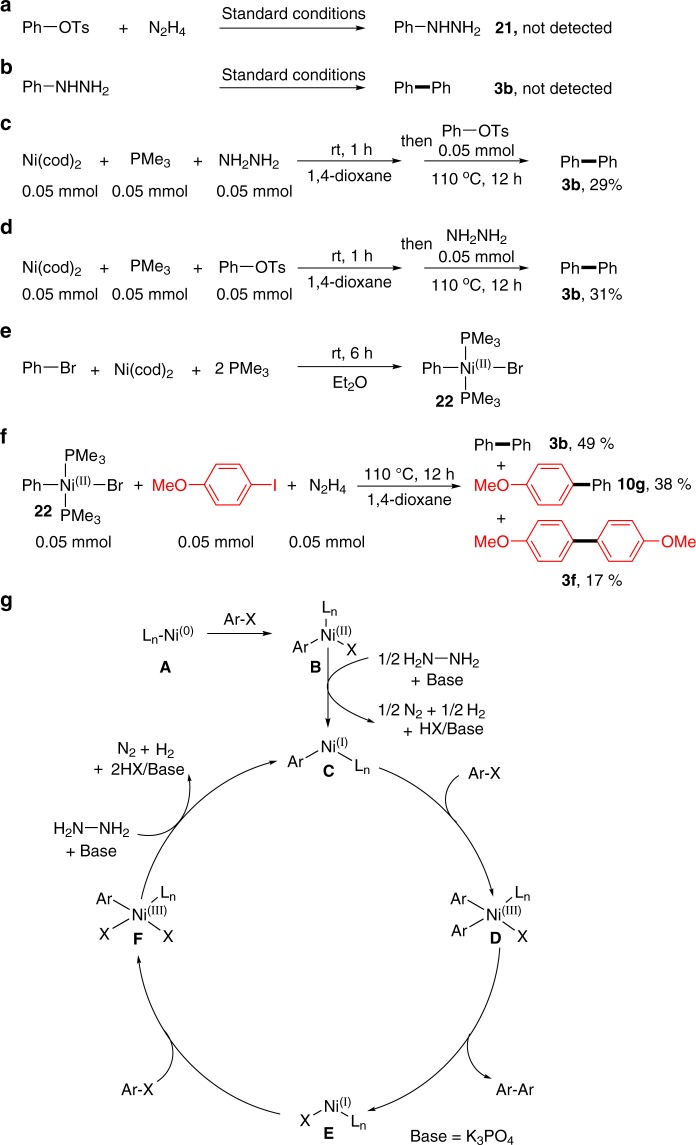
Mechanistic studies. **a** Reaction of phenyl tosylate with hydrazine did not give phenyl hydrazine; **b** reaction of phenyl hydrazine under the standard conditions did not give the biphenyl product; **c** preparation of *trans*-[Ni(II)PhBr(Me_3_P)_2_] complex; **d** stoichiometric reaction of nickel complex with an aryl iodide in the presence of hydrazine; **e** pre-mix equimolar Ni(cod)_2_/Me_3_P/hydrazine in dioxane for 1 h, then add the phenyl tosylate; **f** Pre-mix equimolar Ni(cod)_2_/Me_3_P/ phenyl tosylate in dioxane for 1 h, then add the hydrazine; **g** proposed pathway for the coupling of aryl electrophiles with N_2_H_4_ as traceless mediator

With respect to the mechanism, as reported in the literature, biaryl skeleton can be accessed by metal-catalyzed oxidative homo-coupling of aryl hydrazine^36^. In order to determine if our reaction proceeds through the aryl hydrazine intermediate, two control experiments were carried out (Fig. 9). First, phenyl hydrazine (**21**) was not detected during the reaction of phenyl tosylate with hydrazine, which demonstrated that the Buchwald-Hartwig type amination reaction could not take place under our conditions (Fig. 9a)^37,38^. Moreover, when phenyl hydrazine (**21**) was tested under the standard conditions, no desired product **3b** was detected, which ruled out the pathway involving the homo-coupling of aryl hydrazine (Fig. 9b). To investigate if L_n_Ni-NH_2_NH_2_ species was involved in the reaction, two control experiments were then carried out. When equimolar Ni(cod)_2_/Me_3_P/hydrazine was premixed in 1,4-dioxane for 1 h, and then phenyl tosylate was added and reacted under 110 °C for 12 h, the desired **3b** was obtained in 29% yield (Fig. 9c). If equimolar Ni(cod)_2_/Me_3_P/ phenyl tosylate was pre-mixed first in 1,4-dioxane for 1 h, then hydrazine was added and reacted, 31% yield of **3b** was observed (Fig. 9d). These results suggested that the charging sequence of hydrazine or phenyl tosylate made no difference regarding the yield, and L_n_Ni-NH_2_NH_2_ species may not be involved in starting the reaction. To probe the possible reaction pathway, *trans*-[PhNi(II)Br(Me_3_P)_2_] complex **22** was then prepared according to the literature (Fig. 9e)^39^. Stoichiometric reaction of this nickel complex with an aryl iodide in the presence of hydrazine delivered both the homo- and cross-coupling products **3b** (49% yield), **3f** (17% yield) and **10g** (38% yield) (Fig. 9f). These data indicated that L_n_Ni(II)ArX may serve as the intermediate of the reaction.

Although the detailed course of this reductive coupling reaction is not completely clear at this stage, based on the above experiments and literature reports^40–45^, a tentative mechanism is proposed in Fig. 9g. First, oxidative addition of Ar-X to a Ni(0) species **A** forms ArNi(II)XL_n_ (**B**), which undergoes one electron reduction by hydrazine to give ArNi(I)L_n_ (**C**), thus initiating the catalytic cycle. Oxidative addition of another Ar-X to this species delivers a Ni(Ш) complex (**D**), which undergoes rapid reductive elimination to form the desired homo- and/or cross-coupling product and generates Ni(I)L_n_ (**E**). Then oxidative addition of Ar-X to **E**, followed by hydrazine reduction to form ArNi(I)L_n_ (**C**) species again.

## Discussion

In summary, we have demonstrated that N_2_H_4_ can be used as a traceless mediator for the aryl homo- and cross-coupling reactions. This discovery resolved a longstanding challenge of inevitably using stoichiometric amounts of metals (no matter pre-synthesized organometallics or employed in situ) in the transition-metal catalyzed aryl coupling reactions. The fundamental innovation of this strategy is that N_2_ and H_2_ are generated as gaseous side products, thus making the coupling reactions clean and easy to handle. The success of both dimerization and cross-coupling of various aryl electrophiles bearing a wide range of functional groups demonstrated the powerfulness and versatility of this strategy. Furthermore, the homo- and cross-coupling of a series of alkaloids, amino acids and steroids exemplifies the robustness of this protocol in the functionalization of biologically active molecules. During the preparation of our manuscript, Xu and co-workers reported a Pd@PANIs-catalyzed Ullmann reaction of simple aryl iodides with stoichiometric N_2_H_4_ as a reductant^46^. We anticipate that this traceless mediator conception will inspire strategies and opportunities to utilize hydrazine in sustainable chemical synthesis. Further studies on the mechanism and synthetic applications of this protocol are undergoing in our laboratory.

## Methods

### General procedure for aryl homo-coupling

In a glove box, a flame-dried reaction tube (10 mL) equipped with a magnetic stir bar was charged with Ni(cod)_2_ (5.6 mg, 10 mol%), PMe_3_ (8.5 µL, 40 mol%) and 1,4-dioxane (1.0 mL) before being sealed with a rubber septum and taken out of the glove box. The reaction mixture was stirred at room temperature for 30 min. Then aryl electrophile (0.2 mmol), hydrazine solution (1 M in THF, 0.1 mmol, 100 µL) and K_3_PO_4_ (0.6 mmol, 127 mg) were added sequentially. Afterwards, the reaction mixture was sealed with aluminum cap and stirred at 110 °C for 12 h. After the mixture was cooled to room temperature, the resulting solution was directly filtered through a pad of silica and washed with EtOAc (3.0 mL). The solvent was evaporated in vacuo to give the crude product. The residue was purified by preparative TLC (ethyl acetate/petroleum ether) to give the pure corresponding product.

### General procedure for aryl-alkyl cross-coupling

In a glove box, a flame-dried reaction tube (10 cm^3^) equipped with a magnetic stir bar was charged with Ni(cod)_2_ (5.6 mg, 10 mol%), PMe_3_ (8.5 µL, 40 mol%) and 1,4-dioxane (1.0 mL) before being sealed with a rubber septum and taken out of the glove box. The reaction mixture was stirred at room temperature for 30 min. Then aryl electrophile (0.1 mmol), aryl or alkyl bromide (0.3 mmol), hydrazine solution (1 M in THF, 0.2 mmol, 200 µL) and K_3_PO_4_ (0.6 mmol, 127 mg) were added sequentially. After that, the reaction mixture was sealed with aluminum cap and stirred at 110 °C for 12 h. After the mixture was cooled to room temperature, the resulting solution was directly filtered through a pad of silica and washed with EtOAc (3.0 mL). The solvent was evaporated in vacuo to give the crude product. The residue was purified by preparative TLC (ethyl acetate/petroleum ether) to give the pure corresponding product.

## Electronic supplementary material

Supplementary Information

## Data Availability

The authors declare that the data supporting the findings of this study are available within the article and Supplementary Information file, or from the corresponding author upon reasonable request.
