# Supplementary material for: N2H4 as traceless mediator for homo- and cross- aryl coupling
Source: Nat Commun. 2018 Nov 9;9:4739. doi: 10.1038/s41467-018-07198-7 (PMC6226487; doi:10.1038/s41467-018-07198-7)
Supplement: Supplementary file 1 — Supplementary Information [file 41467_2018_7198_MOESM1_ESM.pdf]

Supporting Information

N_2H_4 as Traceless Mediator for Homo- and Cross- Aryl Coupling

Lv et al

Supplementary Notes

General information: ^{1}H NMR spectra were recorded on Bruker 400 or 500 MHz spectrometer and the chemical shifts were reported in parts per million (δ) relative to internal standard TMS (0 ppm) for $CDCl_3$. The peak patterns are indicated as follows: s, singlet; d, doublet; dd, doublet of doublet; t, triplet; q, quartet; m, multiplet. The coupling constants, *J*, are reported in Hertz (Hz). ^{13}C NMR spectra were obtained at Bruker 100 or 125 MHz and referenced to the internal solvent signals (central peak is 77.0 ppm in $CDCl_3$). $CDCl_3$ was used as the NMR solvent. APEX II (Bruker Inc.) was used for HRMS and APCI-MS. Preparative TLC was performed over silica gel 200-300. The reagents (PMe_3, $Ni(COD)_2$) were weighed and handled in a glove box. All reagents were purchased from Alfa, Acros, Aldrich, or TCI and used without further purification. The reduction potentials of some metals and hydrazine are shown in Supplementary Figure 1.

(a) $$Ni^{2+} + 2e^- \xrightarrow{E^\theta = -0.25\ V} Ni$$

(b) $$Zn^{2+} + 2e^- \xrightarrow{E^\theta = -0.76\ V} Zn$$

(c) $$Mn^{2+} + 2e^- \xrightarrow{E^\theta = -1.19\ V} Mn$$

(d) $$Mg^{2+} + 2e^- \xrightarrow{E^\theta = -2.37\ V} Mg$$

(e) $$N_2 + 2H_2O + H_2 + 2e^- \xrightarrow{E^\theta = -1.49\ V} N_2H_4 + 2OH^-$$

(f) $$N_2 + 4H_2O + 4e^- \xrightarrow{E^\theta = -1.16\ V} N_2H_4 + 4OH^-$$

Supplementary Figure 1. Reduction potentials of some metals and hydrazine. **(a)** Reduction potential of Ni^{2+}/Ni; **(b)** Reduction potential of Zn^{2+}/Zn; **(c)** Reduction potential of Mn^{2+}/Mn; **(d)** Reduction potential of Mg^{2+}/Mg; **(e)** One kind of reduction potential of N_2H_4; **(f)** Another kind of reduction potential of N_2H_4.

Supplementary Methods

Experimental procedures for aryl homo-coupling

In a glove box, a flame-dried reaction tube (10 mL) equipped with a magnetic stir bar was charged with $Ni(cod)_2$ (5.6 mg, 10 mol%), PMe_3 (8.5 μL, 40 mol%) and 1,4-dioxane (1.0 mL) before being sealed with a rubber septum and taken out of the glove box. The reaction mixture was stirred at room temperature for 30 min. Then aryl electrophile **1** or **4** (0.2 mmol), hydrazine solution (1 M in THF, 0.1 mmol, 100 μL) and K_3PO_4 (0.6 mmol, 127 mg) were added sequentially. After that, the reaction mixture was sealed with aluminum cap and stirred at 110 ℃ for 12 h. After the mixture was cooled to room temperature, the resulting solution was directly filtered through a pad of

silica and washed with EtOAc (3.0 mL). The solvent was evaporated *in vacuo* to give the crude product. The residue was purified by preparative TLC (ethyl acetate/petroleum ether) to give the pure corresponding product **3** or **5**.

Experimental procedures for aryl-alkyl cross-coupling

In a glove box, a flame-dried reaction tube (10 cm^3) equipped with a magnetic stir bar was charged with $Ni(cod)_2$ (5.6 mg, 10 mol%), PMe_3 (8.5 μL, 40 mol%) and 1,4-dioxane (1.0 mL) before being sealed with a rubber septum and taken out of the glove box. The reaction mixture was stirred at room temperature for 30 min. Then aryl triflate **1** (0.1 mmol), aryl or alkyl bromide (0.3 mmol), hydrazine solution (1 M in THF, 0.2 mmol, 200 μL) and K_3PO_4 (0.6 mmol, 127 mg) were added sequentially. After that, the reaction mixture was sealed with aluminum cap and stirred at 110 ℃ for 12 h. After the mixture was cooled to room temperature, the resulting solution was directly filtered through a pad of silica and washed with EtOAc (3.0 mL). The solvent was evaporated *in vacuo* to give the crude product. The residue was purified by preparative TLC (ethyl acetate/petroleum ether) to give the pure corresponding product **10**.

Data analyst

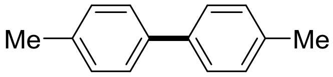

4,4'-Dimethyl-1,1'-biphenyl (3a). (16 mg, 88%). Isolated by preparative TLC (hexane, R_f = 0.7); ^{1}H NMR (500 MHz, $CDCl_3$) δ 7.52 (d, J = 8.2 Hz, 4H), 7.28 (d, J = 8.2 Hz, 4H), 2.43 (s, 6H); ^{13}C NMR (125 MHz, $CDCl_3$) δ 138.4, 136.8, 129.6, 126.9, 21.2; EI-MS (m/z): 182.1. The spectroscopic data for this product match the literature data.[1]

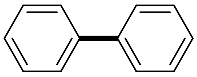

1,1'-Biphenyl (3b). (X = OTf, 14.5 mg, 94%; X = OTs, 13.3 mg, 86%; X = OMs, 10.5 mg, 68%; X = Cl, 10.6 mg, 69%, X = Br, 14.2 mg, 92%; X = I, 12.5 mg, 81%). Isolated by preparative TLC (hexane, R_f = 0.7); ^{1}H NMR (500 MHz, $CDCl_3$) δ 7.66 (d, J = 7.2 Hz, 4H), 7.50 (t, J = 7.2 Hz, 4H), 7.41 (t, J = 7.6 Hz, 2H); ^{13}C NMR (100 MHz, $CDCl_3$) δ 141.3, 128.8, 127.3, 127.2; EI-MS (m/z): 154.1. The spectroscopic data for this product match the literature data.[1]

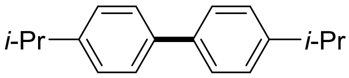

4,4'-Di-*iso*propyl-1,1'-biphenyl (3c). (21.5 mg, 90%). Isolated by preparative TLC (hexane, R_f = 0.7); ^{1}H NMR (500 MHz, $CDCl_3$) δ 7.55 (d, J = 8.2 Hz, 4H), 7.32 (d, J = 8.2 Hz, 4H), 3.03-2.94 (m, 2H), 1.33 (s, 6H), 1.32 (s,

6H); ^{13}C NMR (125 MHz, $CDCl_3$) δ 147.7, 138.7, 127.0, 126.8, 33.8, 24.0; EI-MS (m/z): 238.2. The spectroscopic data for this product match the literature data.[2]

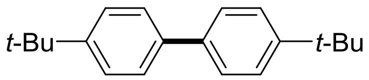

4,4'-Di-*tert*-butyl-1,1'-biphenyl (3d). (23.4 mg, 88%). Isolated by preparative TLC (hexane, R_f = 0.7); ^{1}H NMR (500 MHz, $CDCl_3$) δ 7.57 (d, J = 8.4 Hz, 4H), 7.49 (d, J = 8.4 Hz, 4H), 1.40 (s, 18H); ^{13}C NMR (125 MHz, $CDCl_3$) δ 149.9, 138.2, 126.7, 125.7, 34.5, 31.4; EI-MS (m/z): 266.1. The spectroscopic data for this product match the literature data.[1]

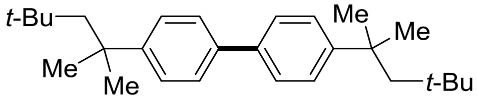

4,4'-Bis(2,4,4-trimethylpentan-2-yl)-1,1'-biphenyl (3e). (32.5 mg, 86%). Isolated by preparative TLC (hexane, R_f = 0.5); IR (neat): ν_{max} 2952, 2905, 2871, 1498, 1469, 1393, 1364, 819, 589 cm^{-1}; ^{1}H NMR (500 MHz, $CDCl_3$) δ 7.57 (d, J = 8.4 Hz, 4H), 7.45 (d, J = 8.4 Hz, 4H), 1.80 (s, 4H), 1.43 (s, 12H), 0.78 (s, 18H); ^{13}C NMR (125 MHz, $CDCl_3$) δ 148.9, 137.7, 126.5, 126.1, 56.9, 38.4, 32.4, 31.8, 31.5; EI-MS (m/z): 378.3; HRMS (APCI) calcd for $C_{28}H_{43}$ [M + H$^+$], 379.3359; found: 379.3356.

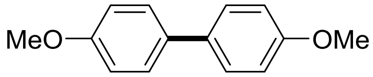

4,4'-Dimethoxy-1,1'-biphenyl (3f). (19.5 mg, 81%). Isolated by preparative TLC (hexane: ethyl acetate = 30:1, R_f = 0.6); ^{1}H NMR (500 MHz, $CDCl_3$) δ 7.50 (d, J = 8.8 Hz, 4H), 6.98 (d, J = 8.8 Hz, 4H), 3.87 (s, 6H); ^{13}C NMR (125 MHz, $CDCl_3$) δ 158.7, 133.5, 127.7, 114.2, 55.4; EI-MS (m/z): 214.1. The spectroscopic data for this product match the literature data.[1]

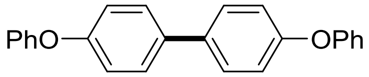

4,4'-Diphenoxy-1,1'-biphenyl (3g). (30 mg, 89%). Isolated by preparative TLC (hexane: ethyl acetate = 20:1, R_f = 0.6); ^{1}H NMR (500 MHz, $CDCl_3$) δ 7.56 (d, J = 8.6 Hz, 4H), 7.39 (t, J = 7.6 Hz, 4H), 7.16 (t, J = 7.6 Hz, 2H), 7.10 (d, J = 8.6 Hz, 4H), 7.09 (t, J = 7.6 Hz, 4H); ^{13}C NMR (125 MHz, $CDCl_3$) δ 157.2, 156.7, 135.7, 129.8, 128.2, 123.4, 119.1, 119.0; EI-MS (m/z): 338.0. The spectroscopic data for this product match the literature data.[1]

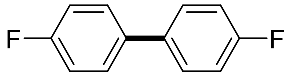

4,4'-Difluoro-1,1'-biphenyl (3h). (14.8 mg, 78%). Isolated by preparative TLC (hexane, R_f = 0.7); ^{1}H NMR (500 MHz, $CDCl_3$) δ 7.53-7.50 (m, 4H), 7.15 (t, J = 8.6 Hz, 4H); ^{13}C NMR (125 MHz, $CDCl_3$) δ 162.5 (d, J =

246.7 Hz), 136.4 (d, J = 3.0 Hz), 128.6 (d, J = 8.1 Hz), 115.7 (d, J = 21.3 Hz); ^{19}F NMR (470 MHz, $CDCl_3$) δ -115.8; EI-MS (m/z): 190.1. The spectroscopic data for this product match the literature data.[1]

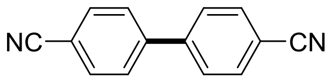

[1,1'-Biphenyl]-4,4'-dicarbonitrile (3i).[1] (18 mg, 88%). Isolated by preparative TLC (hexane: ethyl acetate = 20:1, R_f = 0.5); ^{1}H NMR (500 MHz, $CDCl_3$) δ 7.82-7.80 (m, 4 H), 7.73-7.71 (m, 4 H); ^{13}C NMR (125 MHz, $CDCl_3$) δ 143.5, 132.9, 127.9, 118.4, 112.5; EI-MS (m/z): 204.1. The spectroscopic data for this product match the literature data.[1]

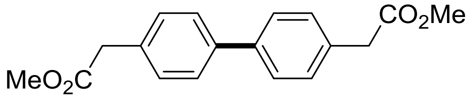

Dimethyl 2,2'-([1,1'-biphenyl]-4,4'-diyl)diacetate (3j). (21.5 mg, 72%). Isolated by preparative TLC (hexane: ethyl acetate = 10:1, R_f = 0.6); ^{1}H NMR (500 MHz, $CDCl_3$) δ 7.57 (d, J = 8.2 Hz, 4H), 7.37 (d, J = 8.2 Hz, 4H), 3.74 (s, 6H), 3.70 (s, 4H); ^{13}C NMR (125 MHz, $CDCl_3$) δ 172.0, 139.7, 133.0, 129.7, 127.3, 52.1, 40.8; EI-MS (m/z): 298.2. The spectroscopic data for this product match the literature data.[3]

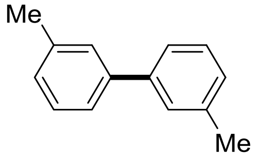

3,3'-Dimethyl-1,1'-biphenyl (3k). (13.5 mg, 74%). Isolated by preparative TLC (hexane, R_f = 0.7); ^{1}H NMR (500 MHz, $CDCl_3$) δ 7.44-7.42 (m, 4H), 7.36 (t, J = 7.4 Hz, 2H), 7.19 (d, J = 7.4 Hz, 2H), 2.46 (s, 6H); ^{13}C NMR (125 MHz, $CDCl_3$) δ 141.4, 138.3, 128.6, 128.0, 127.9, 124.3, 21.6; EI-MS (m/z): 182.1. The spectroscopic data for this product match the literature data.[4]

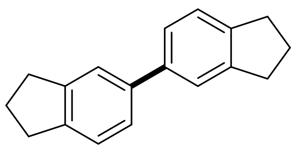

2,2',3,3'-Tetrahydro-1H,1'H-5,5'-biindene (3l). (20.4 mg, 87%). Isolated by preparative TLC (hexane, R_f = 0.6); IR (neat): ν_{max} 2924, 2838, 1475, 1431, 1034, 895, 815 cm^{-1}; ^{1}H NMR (500 MHz, $CDCl_3$) δ 7.48 (s, 2H), 7.39 (d, J = 7.8 Hz, 2H), 7.31 (d, J = 7.8 Hz, 2H), 3.02-2.97 (m, 8H), 2.19-2.13 (m, 4H); ^{13}C NMR (125 MHz, $CDCl_3$) δ 144.8, 143.0, 140.0, 125.3, 124.5, 123.2, 32.9, 32.6, 25.6; EI-MS (m/z): 234.2; HRMS (APCI) calcd for $C_{18}H_{19}$ [M + H$^+$], 235.1481; found: 235.1476.

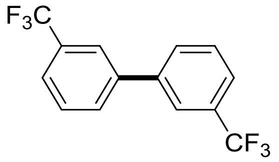

3,3'-Bis(trifluoromethyl)-1,1'-biphenyl (3m). (22 mg, 76%). Isolated by preparative TLC (hexane, R_f = 0.7); ^{1}H NMR (500 MHz, $CDCl_3$) δ 7.86 (s, 2H), 7.80 (d, J = 7.6 Hz, 2H), 7.69 (d, J = 7.6 Hz, 2H), 7.63 (t, J = 7.6 Hz, 2H); ^{13}C NMR (125 MHz, $CDCl_3$) δ 140.6, 131.5 (q, J = 32.4 Hz), 130.6, 129.5, 124.7 (q, J = 3.7 Hz), 124.1 (q, J = 3.7 Hz), 124.0 (q, J = 272.4 Hz), ^{19}F NMR (470 MHz, $CDCl_3$) δ -62.7; EI-MS (m/z): 290.1. The spectroscopic data for this product match the literature data.[5]

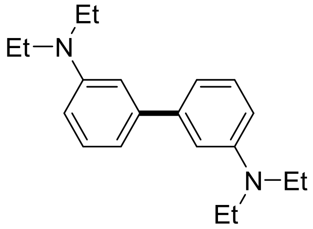

***N*^{3},*N*^{3},*N*$^{3'}$,*N*$^{3'}$-Tetraethyl-[1,1'-biphenyl]-3,3'-diamine (3n)**. (19 mg, 64%). Isolated by preparative TLC (hexane:ethyl acetate = 10:1, R_f = 0.5); ^{1}H NMR (500 MHz, $CDCl_3$) δ 7.29 (t, J = 8.0 Hz, 2H), 6.91-6.88 (m, 4H), 6.71 (dd, J = 8.0, 2.4 Hz, 2H), 3.43 (q, J = 7.0 Hz, 8H), 1.23 (t, J = 7.0 Hz, 12H); ^{13}C NMR (125 MHz, $CDCl_3$) δ 148.0, 143.9, 129.4, 114.9, 111.1, 110.6, 44.5, 12.7; EI-MS (m/z): 296.3. The spectroscopic data for this product match the literature data.[5]

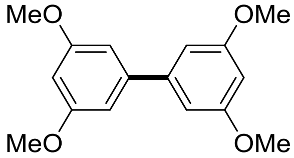

3,3',5,5'-Tetramethoxy-1,1'-biphenyl (3o). (23 mg, 84%). Isolated by preparative TLC (hexane: ethyl acetate = 5:1, R_f = 0.5); ^{1}H NMR (500 MHz, $CDCl_3$) δ 6.74 (d, J = 2.2 Hz, 4H), 6.50 (t, J = 2.2 Hz, 2H), 3.87 (s ,12H); ^{13}C NMR (125 MHz, $CDCl_3$) δ 160.1, 143.5, 105.5, 99.5, 55.5; EI-MS (m/z): 274.2. The spectroscopic data for this product match the literature data.[4]

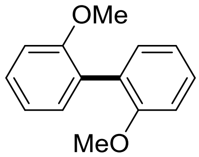

2,2'-Dimethoxy-1,1'-biphenyl (3p). (18.4 mg, 86%). Isolated by preparative TLC (hexane: ethyl acetate = 20:1, R_f = 0.6); ^{1}H NMR (500 MHz, $CDCl_3$) δ 7.36 (td, J = 7.8, 1.8 Hz, 2H), 7.28 (dd, J = 7.4, 1.8 Hz, 2H), 7.04 (td, J = 7.4, 1.0 Hz, 2H), 7.01 (d, J = 7. 8 Hz, 2H), 3.80 (s, 6H); ^{13}C NMR (125 MHz, $CDCl_3$) δ 157.2, 131.5, 128.6, 127.8, 120.4, 111.1, 55.7; EI-MS (m/z): 214.1. The spectroscopic data for this product match the literature data.[1]

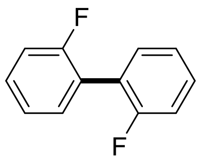

2,2'-Difluoro-1,1'-biphenyl (3q). (16.7 mg, 88%). Isolated by preparative TLC (hexane, R_f = 0.7); ^{1}H NMR (500 MHz, $CDCl_3$) δ 7.44-7.38 (m, 4H), 7.25 (td, J = 7.4, 1.2 Hz, 2H), 7.22-7.18 (m, 2H); ^{13}C NMR (125 MHz, $CDCl_3$) δ 159.9 (dd, J = 248.6, 13.1 Hz), 131.7 (t, J = 2.6 Hz), 129.9 (t, J = 4.0 Hz), 124.2 (t, J = 2.2 Hz), 115.9 (d, J = 6.4 Hz), 115.6 (d, J = 6.4 Hz); ^{19}F NMR (470 MHz, $CDCl_3$) δ -114.8; EI-MS (m/z): 190.0. The spectroscopic data for this product match the literature data.[6]

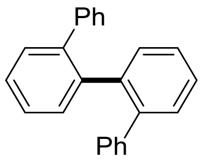

1,1':2',1'':2'',1'''-Quaterphenyl (3r). (16.2 mg, 53%). Isolated by preparative TLC (hexane, R_f = 0.5); ^{1}H NMR (500 MHz, $CDCl_3$) δ 7.45-7.43 (m, 2H), 7.40-7.34 (m, 4H), 7.21-7.19 (m, 2H), 7.13-7.09 (m, 2H), 7.04-7.01 (m, 4H), 6.65-6.63 (m, 4H); ^{13}C NMR (125 MHz, $CDCl_3$) δ 141.0, 140.9, 140.0, 131.7, 130.0, 129.3, 127.5, 127.4, 127.1, 125.9; EI-MS (m/z): 306.2. The spectroscopic data for this product match the literature data.[5]

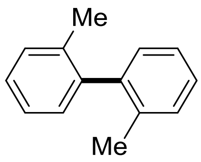

2,2'-Dimethyl-1,1'-biphenyl (3s). (9.6 mg, 53%). Isolated by preparative TLC (hexane, R_f = 0.8); ^{1}H NMR (500 MHz, $CDCl_3$) δ 7.31-7.24 (m, 6 H), 7.15-7.13 (m, 2 H), 2.09 (s, 6 H); ^{13}C NMR (125 MHz, $CDCl_3$) δ 141.6, 135.8, 129.8, 129.3, 127.2, 125.5, 19.8; EI-MS (m/z): 182.1. The spectroscopic data for this product match the literature data.[1]

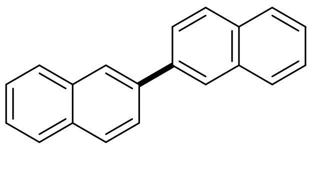

2,2'-Binaphthalene (3t). (22.1 mg, 87%). Isolated by preparative TLC (hexane, R_f = 0.7); ^{1}H NMR (500 MHz, $CDCl_3$) δ 8.21 (s, 2H), 8.01-7.96 (m, 4H), 7.94-7.90 (m, 4H), 7.58-7.52 (m, 4H); ^{13}C NMR (125 MHz, $CDCl_3$) δ 138.4, 133.8, 132.7, 128.5, 128.3, 127.7, 126.4, 126.1, 126.0, 125.8; EI-MS (m/z): 254.1. The spectroscopic data for this product match the literature data.[2]

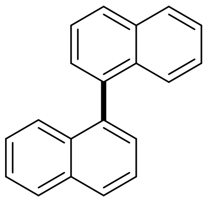

1,1'-Binaphthalene (3u). (24.1 mg, 95%). Isolated by preparative TLC (hexane, R_f = 0.7); ^1H NMR (500 MHz, $CDCl_3$) δ 8.00-7.97 (m, 4H), 7.65-7.62 (m, 2H), 7.55-7.50 (m, 4H), 7.44 (d, J = 8.4 Hz, 2H), 7.34-7.31 (m, 2H); ^{13}C NMR (125 MHz, $CDCl_3$) δ 138.5, 133.6, 132.9, 128.2, 127.9, 127.8, 126.6, 126.0, 125.8, 125.4; EI-MS (m/z): 254.1. The spectroscopic data for this product match the literature data.[1]

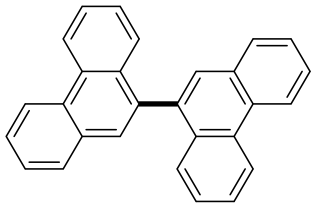

5,6-Dihydro-9,9'-biphenanthrene (3v). (16.2 mg, 91%, 0.1 mmol scale). Isolated by preparative TLC (hexane, R_f = 0.7); ^1H NMR (500 MHz, $CDCl_3$) δ 8.85 (t, J = 7.6 Hz, 4H), 7.96 (dd, J = 7.8, 1.2 Hz, 2H), 7.89 (s, 2 H), 7.78-7.75 (m, 2H), 7.71-7.67 (m, 4H), 7.54 (dd, J = 8.2, 1.0 Hz, 2H), 7.42-7.39 (m, 2H); ^{13}C NMR (125 MHz, $CDCl_3$) δ 137.2, 132.2, 131.6, 130.3, 128.7, 128.5, 127.6, 126.9, 126.8, 126.6, 126.5, 122,8, 122.7; EI-MS (m/z): 354.2. The spectroscopic data for this product match the literature data.[7]

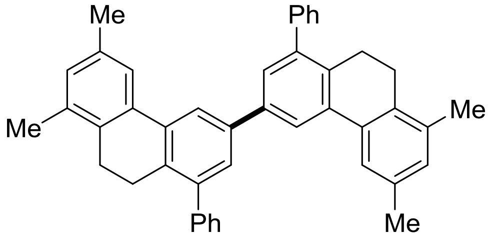

6,6',8,8'-Tetramethyl-1,1'-diphenyl-9,9',10,10'-tetrahydro-3,3'-biphenanthrene (3w). (25.5 mg, 90%, 0.1 mmol scale). Isolated by preparative TLC (hexane, R_f = 0.5); IR (neat): ν_{max} 3053, 3026, 2945, 2915, 2856, 2833, 1597, 1574, 1495, 1425, 851, 758, 698 cm^{-1}; ^1H NMR (500 MHz, $CDCl_3$) δ 8.06 (d, J = 1.7 Hz, 2H), 7.61-7.59 (m, 4H), 7.51-7.46 (m, 8H), 7.44-7.41 (m, 2H), 7.03 (s, 2H), 2.88-2.85 (m, 4H), 2.75-2.72 (m, 4H), 2.43 (s, 6H), 2.35 (s, 6H); ^{13}C NMR (125 MHz, $CDCl_3$) δ 141.5, 141.3, 139.5, 136.2, 135.7, 134.9, 134.8, 134.0, 133.2, 130.4, 129.5, 128.2, 127.7, 127.1, 122.9, 122.5, 26.2, 24.5, 21.3, 19.7; HRMS (APCI) calcd for $C_{44}H_{39}$ [M + H^+], 567.3046; found: 567.3049.

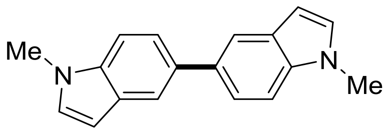

1,1'-Dimethyl-1H,1'H-5,5'-biindole (3x). (24.2 mg, 93%). Isolated by preparative TLC (hexane:ethyl acetate = 4:1, R_f = 0.5); IR (neat): ν_{max} 3120, 3097, 3015, 2918, 2820, 1510, 1474, 1420, 1326, 1242, 802, 760 cm^{-1}; ^{1}H NMR (500 MHz, $CDCl_3$) δ 7.91 (d, J = 1.4 Hz, 2H), 7.58 (dd, J = 8.4, 1.6 Hz, 2H), 7.42 (d, J = 8.4 Hz, 2H), 7.10 (d, J = 2.8 Hz, 2H), 6.57 (d, J = 2.8 Hz, 2H), 3.86 (s, 6H); ^{13}C NMR (100 MHz, $CDCl_3$) δ 135.9, 134.3, 129.2, 129.0, 122.0, 119.5, 109.2, 101.2, 32.9; HRMS (APCI) calcd for $C_{18}H_{17}N_2$ [M + H$^+$], 261.1386; found: 261.1388.

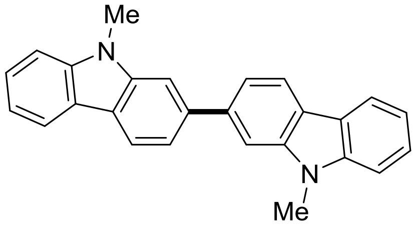

9,9'-Dimethyl-9H,9'H-2,2'-bicarbazole (3y). (33.2 mg, 92%). Isolated by preparative TLC (hexane:ethyl acetate = 4:1, R_f = 0.5); ^{1}H NMR (500 MHz, DMSO-*d6*) δ 8.26 (d, J = 8.0 Hz, 2H), 8.19 (d, J = 7.4 Hz, 2H), 8.05 (s, 2H), 7.71 (d, J = 8.0 Hz, 2H), 7.62 (d, J = 8.0 Hz, 2H), 7.49 (t, J = 7.4 Hz, 2H), 7.24 (t, J = 7.4 Hz, 2H), 4.00 (s, 6H); ^{13}C NMR (125 MHz, DMSO-*d6*) δ 141.8, 141.7, 139.5, 126.2, 122.3, 121.6, 121.0, 120.7, 119.3, 118.9, 109.6, 108.1, 29.6; HRMS (APCI) calcd for $C_{26}H_{21}N_2$ [M + H$^+$], 361.1699; found: 361.1697. The spectroscopic data for this product match the literature data.[7]

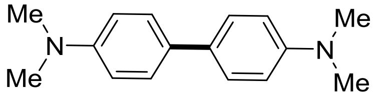

***N*^{4},*N*^{4},*N*$^{4'}$,*N*$^{4'}$-tetramethyl-[1,1'-biphenyl]-4,4'-diamine (5a)**. (17.5 mg, 73%). Isolated by preparative TLC (hexane: ethyl acetate = 5:1, R_f = 0.6); ^{1}H NMR (500 MHz, $CDCl_3$) δ 7.49 (d, J = 8.8 Hz, 4H), 6.84 (d, J = 8.8 Hz, 4H), 3.00 (s, 12 H); ^{13}C NMR (125 MHz, $CDCl_3$) δ 149.3, 129.9, 127.0, 113.1, 40.8; EI-MS (m/z): 240.2. The spectroscopic data for this product match the literature data.[5]

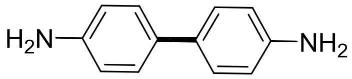

Benzidine (5b). (12.7 mg, 69%). Isolated by preparative TLC (hexane: ethyl acetate = 1:1, R_f = 0.4); ^{1}H NMR (500 MHz, $CDCl_3$) δ 7.37 (d, J = 8.4 Hz, 4H), 6.75 (d, J = 8.4 Hz, 4H), 3.69 (br, 4H); ^{13}C NMR (125 MHz, $CDCl_3$) δ 145.0, 131.9, 127.3, 115.5; EI-MS (m/z): 184.1. The spectroscopic data for this product match the literature data.[8]

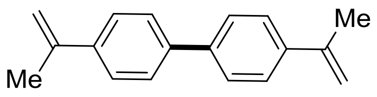

4,4'-Di(prop-1-en-2-yl)-1,1'-biphenyl (5c). (20.1 mg, 86%). Isolated by preparative TLC (hexane, R_f = 0.4); ^{1}H NMR (500 MHz, $CDCl_3$) δ 7.61 (d, J = 8.6 Hz, 4H), 7.58 (d, J = 8.6 Hz, 4H), 5.46 (q, J = 0.6 Hz, 2H), 5.15-5.14 (m, 2H), 2.22 (q, J = 0.6 Hz, 6H); ^{13}C NMR (125 MHz, $CDCl_3$) δ 142.8, 140.1, 139.7, 126.7, 125.9, 112.5, 21.8; EI-MS (m/z): 234.2. The spectroscopic data for this product match the literature data.[9]

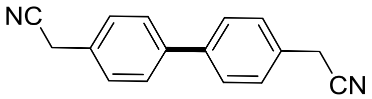

2,2'-([1,1'-Biphenyl]-4,4'-diyl)diacetonitrile (5d). (18.8 mg, 81%). Isolated by preparative TLC (hexane: ethyl acetate = 3:1, R_f = 0.4); ^{1}H NMR (500 MHz, $CDCl_3$) δ 7.62 (d, J = 8.4 Hz, 4H), 7.44 (d, J = 8.4 Hz, 4H), 3.83 (s, 4H); ^{13}C NMR (125 MHz, $CDCl_3$) δ 140.1, 129.3, 128.5, 127.8, 117.7, 23.4; EI-MS (m/z): 232.1. The spectroscopic data for this product match the literature data.[10]

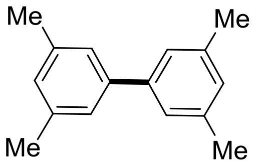

3,3',5,5'-Tetramethyl-1,1'-biphenyl (5e). (17.3 mg, 82%). Isolated by preparative TLC (hexane, R_f = 0.7); ^{1}H NMR (500 MHz, $CDCl_3$) δ 7.23 (s, 4H), 7.02 (s, 2H), 2.42 (s, 12H); ^{13}C NMR (125 MHz, $CDCl_3$) δ 141.5, 138.1, 128.7, 125.1, 21.4; EI-MS (m/z): 210.2. The spectroscopic data for this product match the literature data.[11]

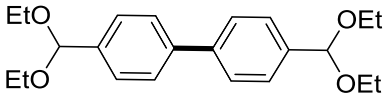

4,4'-Bis(diethoxymethyl)-1,1'-biphenyl (5f). (24.7 mg, 69%). Isolated by preparative TLC (hexane: ethyl acetate = 10:1, R_f = 0.4); IR (neat): ν_{max} 3054, 2978, 2880, 1264, 1095, 1053 cm^{-1}; ^{1}H NMR (500 MHz, $CDCl_3$) δ 7.61 (d, J = 8.4 Hz, 4H), 7.56 (d, J = 8.4 Hz, 4H), 5.57 (s, 2H), 3.72-3.66 (m, 4H), 3.63-3.57 (m, 4H), 1.29 (t, J = 7.0 Hz, 12H); ^{13}C NMR (125 MHz, $CDCl_3$) δ 140.9, 138.2, 127.1, 127.0, 101.5, 61.1, 15.2; EI-MS (m/z): 358.2. HRMS (ESI) calcd for $C_{22}H_{31}O_4$ [M + H$^+$], 359.2217; found: 359.2221.

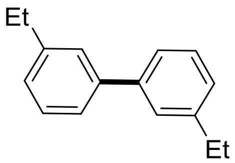

3,3'-Diethyl-1,1'-biphenyl (5g). (16 mg, 76%). Isolated by preparative TLC (hexane, R_f = 0.7); ^{1}H NMR (500 MHz, $CDCl_3$) δ 7.46-7.44 (m, 4H), 7.39 (t, J = 7.6 Hz, 2H), 7.22 (d, J = 7.6 Hz, 2H), 2.76 (q, J = 7.6 Hz, 4H), 1.33 (t, J = 7.6 Hz, 6H); ^{13}C NMR (125 MHz, $CDCl_3$) δ 144.7, 141.6, 128.7, 126.9, 126.7, 124.6, 29.0, 15.7; EI-MS (m/z): 210.2. The spectroscopic data for this product match the literature data.[5]

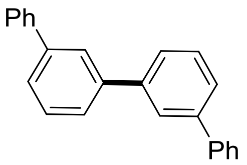

1,1':3',1'':3'',1'''-Quaterphenyl (5h). (25.7 mg, 84%). Isolated by preparative TLC (hexane, R_f = 0.5); ^{1}H NMR (500 MHz, $CDCl_3$) δ 7.90 (t, *J* = 1.6 Hz, 2H), 7.72-7.69 (m, 4H), 7.68-7.67 (m, 2H), 7.66-7.63 (m, 2H), 7.59-7.56 (m, 2H), 7.53-7.49 (m, 4H), 7.43-7.40 (m, 2H); ^{13}C NMR (125 MHz, $CDCl_3$) δ 141.9, 141.8, 141.2, 129.3, 128.9, 127.5, 127.3, 126.3, 126.2; EI-MS (m/z): 306.2. The spectroscopic data for this product match the literature data.[7]

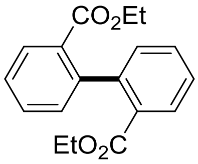

Diethyl [1,1'-biphenyl]-2,2'-dicarboxylate (5i). (21.5 mg, 72%). Isolated by preparative TLC (hexane: ethyl acetate = 5:1, R_f = 0.6); ^{1}H NMR (500 MHz, $CDCl_3$) δ 8.03 (dd, *J* = 7.8, 1.0 Hz, 2H), 7.54 (td, *J* = 7.6, 1.2 Hz, 2H), 7.45 (dd, *J* = 7.6, 1.2 Hz, 2H), 7.23 (dd, *J* = 7.8, 1.0 Hz, 2H), 4.06 (q, *J* = 7.2 Hz, 4H), 1.00 (t, *J* = 7.2 Hz, 6H); ^{13}C NMR (125 MHz, $CDCl_3$) δ 167.2, 143.3, 131.2, 130.2, 129.9, 129.8, 127.1, 60.6, 13.7; EI-MS (m/z): 298.2. The spectroscopic data for this product match the literature data.[11]

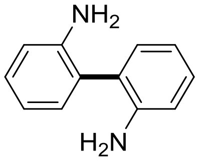

[1,1'-Biphenyl]-2,2'-diamine (5j). (15.3 mg, 83%). Isolated by preparative TLC (hexane: ethyl acetate = 3:1, R_f = 0.4); ^{1}H NMR (500 MHz, $CDCl_3$) δ 7.21 (td, *J* = 7.8, 1.6 Hz, 2H), 7.15 (dd, *J* = 7.8, 1.6 Hz, 2H), 6.86 (td, *J* = 7.8, 1.2 Hz, 2H), 6.81 (dd, *J* = 7.8, 1.2 Hz, 2H), 3.72 (br, 4H); ^{13}C NMR (125 MHz, $CDCl_3$) δ 144.2, 131.1, 128.8, 124.6, 118.8, 115.6; EI-MS (m/z): 184.1. The spectroscopic data for this product match the literature data.[12]

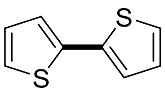

2,2'-Bithiophene (5k). (16.4 mg, 99%). Isolated by preparative TLC (hexane, R_f = 0.7); ^{1}H NMR (500 MHz, $CDCl_3$) δ 7.24 (dd, *J* = 5.1, 1.2 Hz, 2H), 7.21 (dd, *J* = 3.6, 1.2 Hz, 2H), 7.04 (dd, *J* = 5.1, 3.6 Hz, 2H); ^{13}C NMR (125 MHz, $CDCl_3$) δ 137.4, 127.8, 124.4, 123.8; EI-MS (m/z): 166.0. The spectroscopic data for this product match the literature data.[1]

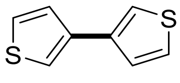

3,3'-Bithiophene (5l). (14.6 mg, 88%). Isolated by preparative TLC (hexane, R_f = 0.7); ^{1}H NMR (500 MHz, $CDCl_3$) δ 7.41 (dd, J = 2.8, 1.4 Hz, 2H), 7.39-7.35 (m, 4H); ^{13}C NMR (125 MHz, $CDCl_3$) δ 137.3, 126.4, 126.1, 119.8; EI-MS (m/z): 166.0. The spectroscopic data for this product match the literature data.[1]

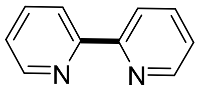

2,2'-Bipyridine (5m). (15.2 mg, 97%). Isolated by preparative TLC (dichloromethane: methanol = 5:1, R_f = 0.5); ^{1}H NMR (500 MHz, $CDCl_3$) δ 8.69 (d, J = 4.2 Hz, 2H), 8.41 (d, J = 8.0 Hz, 2H), 7.81 (td, J = 8.0, 1.6 Hz, 2H), 7.30 (td, J = 6.2, 1.6 Hz, 2H); ^{13}C NMR (125 MHz, $CDCl_3$) δ 156.2, 149.2, 136.9, 123.7, 121.1; EI-MS (m/z): 156.1. The spectroscopic data for this product match the literature data.[13]

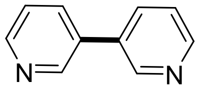

3,3'-Bipyridine (5n). (13.7 mg, 88%). Isolated by preparative TLC (dichloromethane: methanol = 20:1, R_f = 0.4); ^{1}H NMR (500 MHz, $CDCl_3$) δ 8.88 (s, 2H), 8.69 (d, J = 3.4 Hz, 2H), 7.92 (ddd, J = 7.8, 2.2, 1.6 Hz, 2H), 7.45 (dd, J = 7.8, 4.6 Hz, 2H); ^{13}C NMR (125 MHz, $CDCl_3$) δ 149.4, 148.3, 134.4, 133.6, 123.8; EI-MS (m/z): 156.1. The spectroscopic data for this product match the literature data.[14]

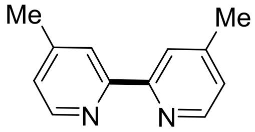

4,4'-Dimethyl-2,2'-bipyridine (5o). (17.2 mg, 93%). Isolated by preparative TLC (dichloromethane: methanol = 5:1, R_f = 0.5); ^{1}H NMR (500 MHz, $CDCl_3$) δ 8.54 (d, J = 5.0 Hz, 2H), 8.24 (t, J = 0.8 Hz, 2H), 7.13 (dd, J = 5.0, 0.8 Hz, 2H), 2.44 (s, 6H); ^{13}C NMR (125 MHz, $CDCl_3$) δ 156.1, 148.9, 148.1, 124.6, 122.0, 21.2; EI-MS (m/z): 184.1. The spectroscopic data for this product match the literature data.[13]

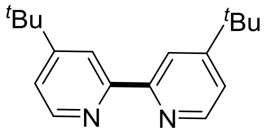

4,4'-Di-*tert*-butyl-2,2'-bipyridine (5p). (24.4 mg, 91%). Isolated by preparative TLC (dichloromethane: methanol = 5:1, R_f = 0.5); ^{1}H NMR (500 MHz, $CDCl_3$) δ 8.62 (dd, J = 5.2, 0.6 Hz, 2H), 8.43 (dd, J = 2.0, 0.6 Hz, 2H), 7.32 (dd, J = 5.2, 2.0 Hz, 2H), 1.41 (s, 18H); ^{13}C NMR (125 MHz, $CDCl_3$) δ 161.0, 156.5, 149.0, 120.7, 118.3, 35.0, 30.6; EI-MS (m/z): 268.2. The spectroscopic data for this product match the literature data.[13]

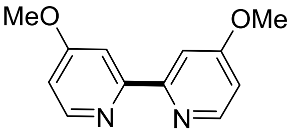

4,4'-Dimethoxy-2,2'-bipyridine (5q). (20.5 mg, 95%). Isolated by preparative TLC (dichloromethane: methanol = 5:1, R_f = 0.4); ^{1}H NMR (500 MHz, $CDCl_3$) δ 8.48 (d, J = 5.6 Hz, 2H), 7.99 (d, J = 2.4 Hz, 2H), 6.86 (dd, J = 5.6, 2.4 Hz, 2H), 3.96 (s, 6H); ^{13}C NMR (125 MHz, $CDCl_3$) δ 166.7, 157.9, 150.1, 111.1, 106.1, 55.3; EI-MS (m/z): 216.1. The spectroscopic data for this product match the literature data.[13]

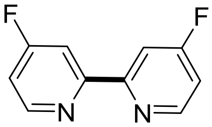

4,4'-Difluoro-2,2'-bipyridine (5r). (12.1 mg, 63%). Isolated by preparative TLC (dichloromethane: methanol = 5:1, R_f = 0.6); ^{1}H NMR (500 MHz, $CDCl_3$) δ 8.67 (t, J = 6.4 Hz, 2H), 8.20 (dd, J = 10.2, 2.2 Hz, 2H), 7.12-7.08 (m, 2H); ^{13}C NMR (125 MHz, $CDCl_3$) δ 169.7 (d, J_{C-F} = 261.6 Hz), 158.3, 151.6 (d, J_{C-F} = 7.0 Hz), 112.1 (d, J_{C-F} = 16.6 Hz), 109.2 (d, J_{C-F} = 18.8 Hz); ^{19}F NMR (470 MHz, $CDCl_3$) δ -101.7; EI-MS (m/z): 192.0. The spectroscopic data for this product match the literature data.[15]

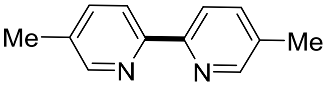

5,5'-Dimethyl-2,2'-bipyridine (5s). (16 mg, 87%). Isolated by preparative TLC (dichloromethane: methanol = 5:1, R_f = 0.6); ^{1}H NMR (500 MHz, $CDCl_3$) δ 8.51 (s, 2H), 8.26 (d, J = 8.0 Hz, 2H), 7.63 (dd, J = 8.0, 1.6 Hz, 2H), 2.41 (s, 6H); ^{13}C NMR (125 MHz, $CDCl_3$) δ 153.8, 149.6, 137.4, 133.0, 120.3, 18.3; EI-MS (m/z): 184.1. The spectroscopic data for this product match the literature data.[15]

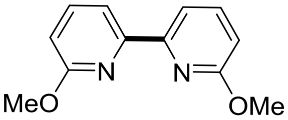

6,6'-Dimethoxy-2,2'-bipyridine (5t). (21.4 mg, 99%). Isolated by preparative TLC (hexane:dichloromethane = 10:1, R_f = 0.5); ^{1}H NMR (500 MHz, $CDCl_3$) δ 8.04 (dd, J = 7.4, 0.6 Hz, 2H), 7.71 (dd, J = 7.4, 8.2 Hz, 2H), 6.78 (dd, J = 8.2, 0.6 Hz, 2H), 4.06 (s, 6H); ^{13}C NMR (125 MHz, $CDCl_3$) δ 163.4, 153.4, 139.2, 113.6, 110.9, 53.2; EI-MS (m/z): 216.1. The spectroscopic data for this product match the literature data.[16]

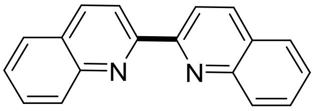

2,2'-Biquinoline (5u). (23.6 mg, 92%). Isolated by preparative TLC (hexane: ethyl acetate = 10:1, R_f = 0.5); ^{1}H NMR (500 MHz, $CDCl_3$) δ 8.88 (d, J = 8.6 Hz, 2H), 8.35 (d, J = 8.6 Hz, 2H), 8.26 (d, J = 8.2 Hz, 2H), 7.90 (d, J = 8.2 Hz, 2H), 7.78 (td, J = 7.6, 1.2 Hz, 2H), 7.60 (td, J = 7.6, 0.8 Hz, 2H); ^{13}C NMR (125 MHz, $CDCl_3$) δ 156.3, 148.0, 136.8, 130.0, 129.6, 128.5, 127.7, 127.0, 119.5; EI-MS (m/z): 256.1. The spectroscopic data for this product match the literature data.[14]

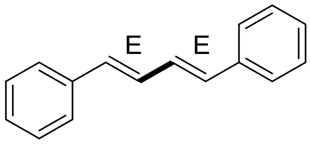

(1*E*,3*E*)-1,4-diphenylbuta-1,3-diene (9a). (14.2 mg, 69%). Isolated by preparative TLC (hexane, R_f = 0.7); ^{1}H NMR (500 MHz, $CDCl_3$) δ 7.48-7.46 (m, 4H), 7.38-7.35 (m, 4H), 7.28-7.25 (m, 2H), 7.02-6.96 (m, 2H), 6.74-6.68 (m, 2H); ^{13}C NMR (125 MHz, $CDCl_3$) δ 137.4, 132.8, 129.3, 128.7, 127.6, 126.4; EI-MS (m/z): 206.1. The spectroscopic data for this product match the literature data.[5]

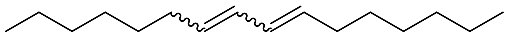

E,E: *E,Z*: *Z,Z* = 10: 2: 1

Hexadeca-7,9-diene (9b). (23.4 mg, 65%). Isolated by preparative TLC (hexane, R_f = 0.8); ^{1}H NMR (500 MHz, $CDCl_3$) δ 6.36-5.95 (m, 2H), 5.74-5.28 (m, 2H), 2.20-2.05 (m, 4H), 1.40-1.24 (m, 16H), 0.92-0.89 (m, 6H); ^{13}C NMR (125 MHz, $CDCl_3$) δ 132.4, 130.3, 32.6, 31.8, 29.4, 28.9, 22.6, 14.1; EI-MS (m/z): 222.3. The spectroscopic data for this product match the literature data.[11]

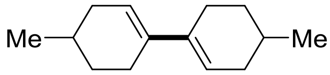

4,4'-Dimethyl-[1,1'-bi(cyclohexane)]-1,1'-diene (9c). (14.1 mg, 74%). Isolated by preparative TLC (hexane, R_f = 0.7); ^{1}H NMR (500 MHz, $CDCl_3$) δ 5.78-5.75 (m, 2H), 2.34-2.13 (m, 6H), 1.82-1.62 (m, 6H), 1.30-1.24 (m, 2H), 0.98 (d, J = 6.6 Hz, 6H); ^{13}C NMR (125 MHz, $CDCl_3$) δ 136.3, 136.2, 121.2, 121.1, 34.5, 31.5, 31.4, 28.5, 28.4, 25.8, 25.5, 21.9, 21.7; EI-MS (m/z): 190.2. The spectroscopic data for this product match the literature data.[18]

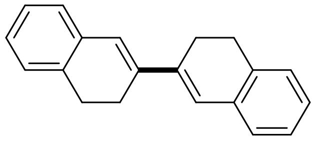

3,3',4,4'-Tetrahydro-2,2'-binaphthalene (9d). (19.6 mg, 76%). Isolated by preparative TLC (hexane, R_f = 0.6); ^{1}H NMR (500 MHz, $CDCl_3$) δ 7.21-7.16 (m, 5H), 7.15-7.12 (m, 3H), 6.76 (s, 2H), 2.94 (t, J = 8.2 Hz, 4H), 2.70 (t, J = 8.2 Hz, 4H); ^{13}C NMR (125 MHz, $CDCl_3$) δ 138.3, 135.6, 134.9, 127.1, 126.9, 126.7, 126.5, 123.3, 28.1, 24.4; EI-MS (m/z): 258.2. The spectroscopic data for this product match the literature data.[19]

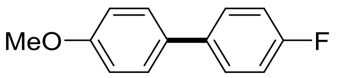

4-Fluoro-4'-methoxy-1,1'-biphenyl (10a). (14.6 mg, 72%). Isolated by preparative TLC (hexane: ethyl acetate = 30:1, R_f = 0.5); ^{1}H NMR (500 MHz, $CDCl_3$) δ 7.53-7.49 (m, 4H), 7.14-7.10 (m, 2H), 7.01-6.98 (m, 2H), 3.88 (s, 3H); ^{13}C NMR (125 MHz, $CDCl_3$) δ 162.1 (d, $J_{C\text{-}F}$ = 245.6 Hz), 159.1, 137.0 (d, $J_{C\text{-}F}$ = 2.8 Hz), 132.9, 128.2 (d, $J_{C\text{-}F}$ = 7.7 Hz), 128.0, 115.5 (d, $J_{C\text{-}F}$ = 21.2 Hz), 114.3, 55.4;^{19}F NMR (470 MHz, $CDCl_3$) δ -116.8; EI-MS (m/z): 202.1. The spectroscopic data for this product match the literature data.[20]

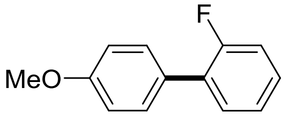

2-Fluoro-4'-methoxy-1,1'-biphenyl (10b). (14.3 mg, 71%). Isolated by preparative TLC (hexane:dichloromethane = 10:1, R_f = 0.3); ^{1}H NMR (500 MHz, $CDCl_3$) δ 7.55-7.52 (m, 2H), 7.45 (td, J = 7.6, 1.8 Hz, 1H), 7.33-7.29 (m, 1H), 7.22 (td, J = 7.6, 1.2 Hz, 1H), 7.19-7.14 (m, 1H), 7.02 (d, J = 8.8 Hz, 2H), 3.89 (s, 3H); ^{13}C NMR (125 MHz, $CDCl_3$) δ 159.8 (d, $J_{C\text{-}F}$ = 247.4 Hz), 159.2, 130.5 (d, $J_{C\text{-}F}$ =3.4 Hz), 130.2, 130.1 (d, $J_{C\text{-}F}$ =3.2 Hz), 128.8, 128.7, 128.4 (d, $J_{C\text{-}F}$ = 8.2 Hz), 128.2, 124.3 (d, $J_{C\text{-}F}$ = 3.6 Hz), 116.1 (d, $J_{C\text{-}F}$ = 22.8 Hz), 113.9, 55.3; ^{19}F NMR (470 MHz, $CDCl_3$) δ -118.2; EI-MS (m/z): 202.1. The spectroscopic data for this product match the literature data.[20]

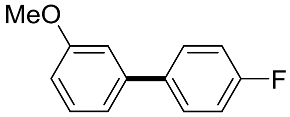

4'-Fluoro-3-methoxy-1,1'-biphenyl (10j). (15.8 mg, 78%). Isolated by preparative TLC (hexane:dichloromethane = 10:1, R_f = 0.3); ^{1}H NMR (500 MHz, $CDCl_3$) δ 7.58-7.55 (m, 2H), 7.38 (t, J = 8.0 Hz, 1H), 7.16-7.13 (m, 3H), 7.10 (t, J = 2.1 Hz, 1H), 6.92 (dd, J = 8.0, 2.4 Hz, 1H), 3.89 (s, 3H); ^{13}C NMR (125 MHz, $CDCl_3$) δ 162.5 (d, $J_{C\text{-}F}$ = 246.4 Hz), 160.0, 141.8, 137.2 (d, $J_{C\text{-}F}$ =3.5 Hz), 129.8, 128.7 (d, $J_{C\text{-}F}$ = 8.0 Hz), 119.5, 115.6 (d, $J_{C\text{-}F}$ = 21.7 Hz), 112.9, 112.6, 55.3; ^{19}F NMR (470 MHz, $CDCl_3$) δ -115.6; EI-MS (m/z): 202.1. The spectroscopic data for this product match the literature data.[21]

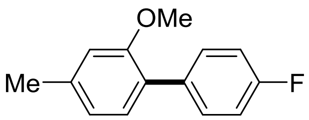

4'-Fluoro-2-methoxy-4-methyl-1,1'-biphenyl (10k). (17.1 mg, 79%). Isolated by preparative TLC (hexane:dichloromethane = 10:1, R_f = 0.3); IR (neat): ν_{max} 2934, 1602, 1493, 1277, 1159, 1039, 838, 808 cm^{-1}; ^{1}H NMR (500 MHz, $CDCl_3$) δ 7.52-7.49 (m, 2H), 7.20 (d, J = 7.6 Hz, 1H), 7.12-7.09 (m, 2H), 6.87 (d, J = 7.6 Hz, 1H), 6.83 (s, 1H), 3.83 (s, 3H), 2.43 (s, 3H); ^{13}C NMR (125 MHz, $CDCl_3$) δ 161.9 (d, $J_{C\text{-}F}$ = 245.6 Hz), 156.2, 138.9,

134.4 (d, $J_{C\text{-}F}$ = 2.9 Hz), 131.0 (d, $J_{C\text{-}F}$ = 7.6 Hz), 130.5, 126.8, 121.5, 114.8 (d, $J_{C\text{-}F}$ = 21.1 Hz), 112.2, 55.5, 21.6; ^{19}F NMR (470 MHz, $CDCl_3$) δ -116.3; HRMS (APCI) calcd for $C_{14}H_{14}FO$ [M + H^+], 217.1023; found: 217.1013.

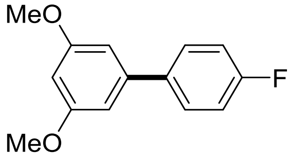

4'-Fluoro-3,5-dimethoxy-1,1'-biphenyl (10l). (21.1 mg, 91%). Isolated by preparative TLC (hexane:dichloromethane = 1:1, R_f = 0.6); IR (neat): ν_{max} 3000, 2935, 2838, 1600, 1586, 1512, 1457, 1217, 1203, 1153, 821 cm^{-1}; ^{1}H NMR (500 MHz, $CDCl_3$) δ 7.57-7.54 (m, 2H), 7.15-7.12 (m, 2H), 6.70 (d, J = 2.2 Hz, 2H), 6.49 (t, J = 2.2 Hz, 1H), 3.87 (s, 6H); ^{13}C NMR (125 MHz, $CDCl_3$) δ 162.5 (d, $J_{C\text{-}F}$ = 246.2 Hz), 161.1, 142.5, 137.3 (d, $J_{C\text{-}F}$ = 2.9 Hz), 128.7 (d, $J_{C\text{-}F}$ = 7.8 Hz), 115.6 (d, $J_{C\text{-}F}$ = 21.7 Hz), 105.4, 99.2, 55.4; ^{19}F NMR (470 MHz, $CDCl_3$) δ -115.3; HRMS (APCI) calcd for $C_{14}H_{14}FO_2$ [M + H^+], 233.0972; found: 233.0972.

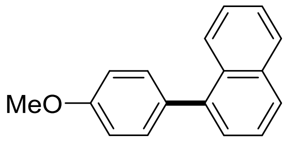

1-(4-Methoxyphenyl)naphthalene (10i). (12.2 mg, 52%). Isolated by preparative TLC (hexane:ethyl acetate = 40:1, R_f = 0.5); ^{1}H NMR (500 MHz, $CDCl_3$) δ 7.95 (d, J = 8.6 Hz, 1H), 7.93 (d, J = 8.4 Hz, 1H), 7.87 (d, J = 8.4 Hz, 1H), 7.55-7.50 (m, 2H), 7.47-7.43 (m, 4H), 7.06 (d, J = 8.6 Hz, 2H), 3.93 (s, 3H); ^{13}C NMR (125 MHz, $CDCl_3$) δ 159.0, 139.9, 133.9, 133.1, 131.8, 131.1, 128.3, 127.3, 126.9, 126.1, 125.9, 125.7, 125.4, 113.7, 55.4; EI-MS (m/z): 234.1. The spectroscopic data for this product match the literature data.[22]

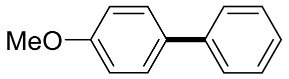

4-Methoxy-1,1'-biphenyl. (**10a**) (11.2 mg, 61%). Isolated by preparative TLC (hexane:ethyl acetate = 40:1, R_f = 0.5); ^{1}H NMR (500 MHz, $CDCl_3$) δ 7.60-7.55 (m, 4H), 7.46-7.43 (m, 2H), 7.35-7.32 (m, 1H), 7.01 (d, J = 8.8 Hz, 2H), 3.89 (s, 3H); ^{13}C NMR (125 MHz, $CDCl_3$) δ 159.2, 140.9, 133.8, 128.7, 128.2, 126.8, 126.7, 114.2, 55.4; EI-MS (m/z): 184.1. The spectroscopic data for this product match the literature data.[22]

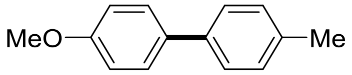

4-Methoxy-4'-methyl-1,1'-biphenyl (10b). (12.3 mg, 62%). Isolated by preparative TLC (hexane: ethyl acetate = 40:1, R_f = 0.6); ^{1}H NMR (500 MHz, $CDCl_3$) δ 7.54 (d, J = 8.8 Hz, 2H), 7.48 (d, J = 8.2 Hz, 2H), 7.26 (d, J = 8.2 Hz, 2H), 7.00 (d, J = 8.8 Hz, 2H), 3.88 (s, 3H), 2.42 (s, 3H); ^{13}C NMR (125 MHz, $CDCl_3$) δ 158.9, 138.0, 136.4, 133.8, 129.4, 128.0, 126.6, 114.2, 55.4, 21.1; EI-MS (m/z): 198.1. The spectroscopic data for this product match the literature data.[22]

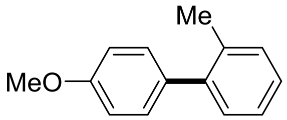

4'-Methoxy-2-methyl-1,1'-biphenyl (10c). (8.2 mg, 41%). Isolated by preparative TLC (hexane: ethyl acetate = 30:1, R_f = 0.5); ^{1}H NMR (500 MHz, $CDCl_3$) δ 7.29-7.24 (m, 6H), 6.99-6.97 (m, 2H), 3.88 (s, 3H), 2.30 (s, 3H); ^{13}C NMR (125 MHz, $CDCl_3$) δ 158.5, 141.6, 135.5, 134.4, 130.3, 130.2, 129.9, 127.0, 125.8, 113.5, 55.3, 20.5; EI-MS (m/z): 198.1. The spectroscopic data for this product match the literature data.[22]

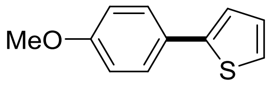

2-(4-Methoxyphenyl)thiophene (10g). (14.1 mg, 74%). Isolated by preparative TLC (hexane:ethyl acetate = 40:1, R_f = 0.5); ^{1}H NMR (500 MHz, $CDCl_3$) δ 7.56 (d, J = 8.8 Hz, 2H), 7.24 (dd, J = 5.0, 1.2 Hz, 1H), 7.22 (dd, J = 3.6, 1.2 Hz, 1H), 7.08 (dd, J = 5.0, 3.6 Hz, 1H), 6.94 (d, J = 8.8 Hz, 2H), 3.86 (s, 3H); ^{13}C NMR (125 MHz, $CDCl_3$) δ 159.2, 144.4, 127.9, 127.3, 127.2, 123.8, 122.1, 114.3, 55.4; EI-MS (m/z): 190.1. The spectroscopic data for this product match the literature data.[23]

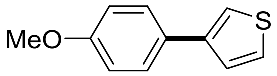

3-(4-Methoxyphenyl)thiophene (10h).[23] (11.6 mg, 61%). Isolated by preparative TLC (hexane:ethyl acetate = 40:1, R_f = 0.5); ^{1}H NMR (500 MHz, $CDCl_3$) δ 7.56 (d, J = 8.8 Hz, 2H), 7.40-7.37 (m, 3H), 6.97 (d, J = 8.8 Hz, 2H), 3.87 (s, 3H); ^{13}C NMR (125 MHz, $CDCl_3$) δ 158.9, 142.0, 128.8, 127.6, 126.3, 126.1, 118.9, 114.2, 55.4; EI-MS (m/z): 190.1. The spectroscopic data for this product match the literature data.[23]

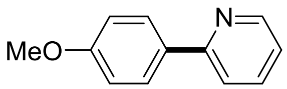

2-(4-Methoxyphenyl)pyridine (10f). [23] (10.4 mg, 56%). Isolated by preparative TLC (hexane:ethyl acetate = 3:1, R_f = 0.5); ^{1}H NMR (500 MHz, $CDCl_3$) δ 8.68-8.67 (m, 1H), 7.97 (d, J = 8.8 Hz, 2H), 7.75-7.68 (m, 2H), 7.21-7.18 (m, 1H), 7.02 (d, J = 8.8 Hz, 2H), 3.89 (s, 3H); ^{13}C NMR (125 MHz, $CDCl_3$) δ 160.5, 157.2, 149.6, 136.7, 132.1, 128.2, 121.4, 119.8, 114.1, 55.4; EI-MS (m/z): 185.1. The spectroscopic data for this product match the literature data.[23]

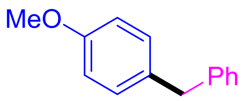

1-Benzyl-4-methoxybenzene (12a). (9.5 mg, 48%). Isolated by preparative TLC (ethyl acetate: hexane = 1:40, R_f = 0.6); ^{1}H NMR (500 MHz, $CDCl_3$) δ 7.33-7.28 (m, 2H), 7.24-7.19 (m, 3H), 7.13 (d, J = 8.5 Hz, 2H), 6.86 (d, J =

8.0 Hz, 2H), 3.96 (s, 2H), 3.81 (s, 3H); ^{13}C NMR (125 MHz, $CDCl_3$) δ 158.0, 141.6, 133.3, 130.0, 128.8, 128.4, 126.0, 113.9, 55.3, 41.0; EI-MS (m/z): 198.1. The spectroscopic data for this product match the literature data.[24]

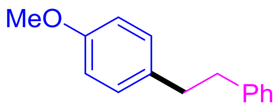

1-Methoxy-4-phenethylbenzene (12b). (13 mg, 61%). Isolated by preparative TLC (ethyl acetate: hexane = 1:40, R_f = 0.6); ^{1}H NMR (500 MHz, $CDCl_3$) δ 7.33-7.30 (m, 2H), 7.24-7.20 (m, 3H), 7.13 (d, J = 8.6 Hz, 2H), 6.86 (d, J = 8.6 Hz, 2H), 3.82 (s, 3H), 2.95-2.87 (m, 4H); ^{13}C NMR (125 MHz, $CDCl_3$) δ 157.9, 141.9, 133.9, 129.4, 128.5, 128.3, 125.9, 113.7, 55.3, 38.2, 37.0; EI-MS (m/z): 212.2. The spectroscopic data for this product match the literature data.[25]

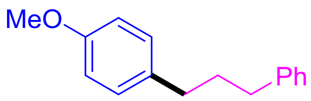

1-Methoxy-4-(3-phenylpropyl)benzene (12c). (15.2 mg, 67%). Isolated by preparative TLC (hexane:ethyl acetate = 40:1, R_f = 0.5; ^{1}H NMR (500 MHz, $CDCl_3$) δ 7.34-7.30 (m, 2H), 7.23-7.20 (m, 3H), 7.14 (d, J = 8.6 Hz, 2H), 6.87 (d, J = 8.6 Hz, 2H), 3.83 (s, 3H), 2.68 (t, J = 7.6 Hz, 2H), 2.64 (t, J = 7.6 Hz, 2H), 2.00-1.94 (m, 2H); ^{13}C NMR (125 MHz, $CDCl_3$) δ 157.7, 142.4, 134.4, 129.3, 128.5, 128.3, 125.7, 113.7, 55.3, 35.4, 34.5, 33.2; EI-MS (m/z): 226.2. The spectroscopic data for this product match the literature data.[25]

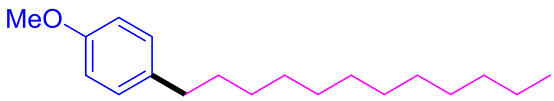

1-Dodecyl-4-methoxybenzene (12d).[26] (15.5 mg, 56%). Isolated by preparative TLC (hexane:ethyl acetate = 40:1, R_f = 0.6; ^{1}H NMR (500 MHz, $CDCl_3$) δ 7.12 (d, J = 8.6 Hz, 2H), 6.85 (d, J = 8.6 Hz, 2H), 3.82 (s, 3H), 2.57 (t, J = 7.8 Hz, 2H), 1.63-1.58 (m, 2H), 1.34-1.28 (m, 18H), 0.92 (t, J = 7.2 Hz, 3H); ^{13}C NMR (125 MHz, $CDCl_3$) δ 157.6, 135.1, 129.2, 113.6, 55.3, 35.1, 31.9, 31.8, 29.7, 29.6, 29.5, 29.4, 29.3, 22.7; EI-MS (m/z): 276.3. The spectroscopic data for this product match the literature data.[26]

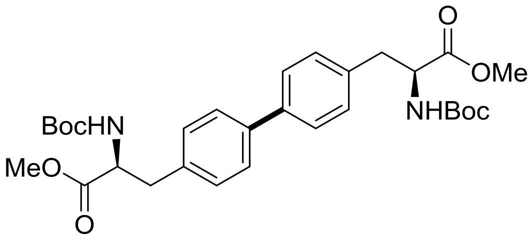

Dimethyl 3,3'-([1,1'-biphenyl]-4,4'-diyl)(2S,2'S)-bis(2-((*tert*-butoxycarbonyl)amino)propanoate) (14). (17.3 mg, 62%, 0.1 mmol scale). Isolated by preparative TLC (hexane: ethyl acetate = 2:1, R_f = 0.5); IR (neat): ν_{max} 3352, 2955, 2928, 1742, 1700, 1514, 1503, 1367, 1222, 1152, 1061, 1020 cm^{-1}; ^{1}H NMR (500 MHz, $CDCl_3$) δ 7.52 (d, J = 8.2 Hz, 4H), 7.21 (d, J = 8.2 Hz, 4H), 5.03 (d, J = 8.0 Hz, 2H), 4.64 (q, J =6.8 Hz, 2H), 3.76 (s, 6H), 3.20-3.09

(m, 4H), 1.45 (s, 18H);^{13}C NMR (125 MHz, $CDCl_3$) δ 173.4, 155.1, 139.5, 135.1, 129.8, 127.1, 80.0, 54.4, 52.3, 38.0, 29.7, 28.3; HRMS (ESI) calcd for $C_{30}H_{40}N_2NaO_8$ [M + Na$^+$], 579.2677; found: 579.2673.

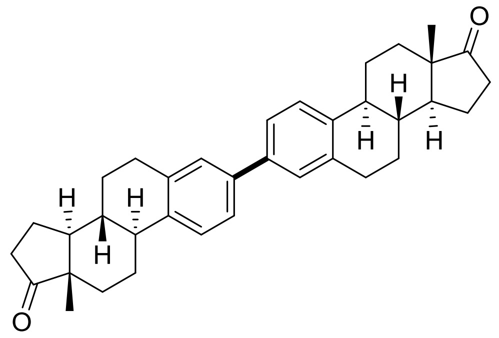

Estrone dimer (16) (22.5 mg, 88%, 0.1 mmol scale). Isolated by preparative TLC (hexane: ethyl acetate = 20:1, R_f = 0.7); IR (neat): ν_{max} 2930, 2887, 2854, 1732, 1488, 1454, 1084, 1005, 883, 800 cm^{-1}; ^{1}H NMR (500 MHz, $CDCl_3$) δ 7.41-7.37 (m, 4H), 7.34 (s, 2H), 3.00 (dd, J = 8.8, 4.0 Hz, 4H), 2.56-2.46 (m, 4H), 2.37 (td, J = 10.6, 3.6 Hz, 2H), 2.21-1.98 (m, 8H), 1.71-1.47 (m, 12H), 0.94 (s, 6H); ^{13}C NMR (125 MHz, $CDCl_3$) δ 220.9, 138.7, 138.6, 136.8, 127.6, 125.8, 124.5, 50.5, 48.0, 44.4, 38.2, 35.9, 31.6, 29.6, 26.6, 25.8, 21.6, 13.9; HRMS (APCI) calcd for $C_{36}H_{43}O_2$ [M + H$^+$], 507.3258; found: 507.3255.

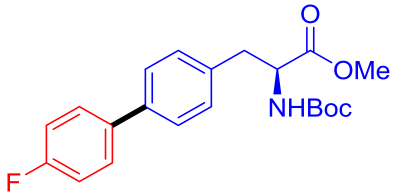

Methyl (S)-2-((*tert*-butoxycarbonyl)amino)-3-(4'-fluoro-[1,1'-biphenyl]-4-yl)propanoate (17). (21.6 mg, 58%, 0.1 mmol scale). Isolated by preparative TLC (hexane:ethyl acetate = 3:1, R_f = 0.6); IR (neat): ν_{max} 3350, 2978, 1710, 1496, 1366, 1159, 1058, 819 cm^{-1}; ^{1}H NMR (500 MHz, $CDCl_3$) δ 7.56-7.53 (m, 2H), 7.49 (d, J = 8.2 Hz, 2H), 7.21 (d, J = 8.2 Hz, 2H), 7.15-7.12 (m, 2H), 5.04 (d, J = 7.6 Hz, 1H), 4.67-4.63 (m, 1H), 3.76 (s, 3H), 3.21-3.09 (m, 2H), 1.45 (s, 9H); ^{13}C NMR (125 MHz, $CDCl_3$) δ 172.3, 162.4 (d, J_{C-F} = 246.1 Hz), 155.1, 138.9, 136.9 (d, J_{C-F} = 3.4 Hz), 135.1, 129.8, 128.5 (d, J_{C-F} = 8.0 Hz), 127.1, 115.6 (d, J_{C-F} = 21.6 Hz), 80.0, 54.4, 52.3, 38.0, 28.3; ^{19}F NMR (470 MHz, $CDCl_3$) δ -115.8; HRMS (ESI) calcd for $C_{21}H_{24}FNNaO_4$ [M + Na$^+$], 396.1582; found: 396.1580.

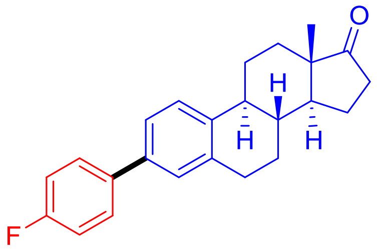

(8R,9S,13S,14S)-3-(4-Fluorophenyl)-13-methyl-6,7,8,9,11,12,13,14,15,16-decahydro-17H-cyclopenta[a]phenanthren-17-one (19). (24 mg, 69%, 0.1 mmol scale). Isolated by preparative TLC (hexane: ethyl acetate = 10:1, R_f = 0.5); IR (neat): ν_{max} 2969, 2918, 2890, 1732, 1492, 1213, 1159, 1084, 1008, 841, 803, 779 cm^{-1}; ^{1}H NMR (500 MHz, $CDCl_3$) δ 7.57-7.54 (m, 2H), 7.41-7.36 (m, 2H), 7.32 (s, 1H), 7.15-7.12 (m, 2H), 3.03-3.01 (m, 2H), 2.58-2.52 (m, 1H), 2.51-2.48 (m, 1H), 2.41-2.36 (m, 1H), 2.22-2.07 (m, 3H), 2.03-2.00 (m, 1H), 1.72-1.48 (m, 6H), 0.96 (s, 3H); ^{13}C NMR (125 MHz, $CDCl_3$) δ 220.8, 162.4 (d, $J_{C\text{-}F}$ = 244.5 Hz), 139.0, 137.8, 137.1 (d, $J_{C\text{-}F}$ = 2.8 Hz), 137.0, 128.5 (d, $J_{C\text{-}F}$ = 7.7 Hz), 127.6, 125.9, 124.4, 115.6 (d, $J_{C\text{-}F}$ = 21.2 Hz), 50.5, 48.0, 44.4, 38.2, 35.9, 31.6, 29.5, 26.5, 25.8, 21.6, 13.9; HRMS (ESI) calcd for $C_{24}H_{26}OF$ [M + Na^+], 349.1962; found: 349.1965.

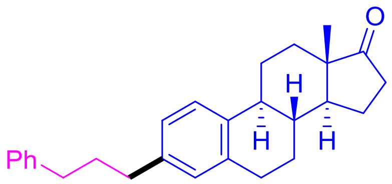

(8R,9S,13S,14S)-13-Methyl-3-(3-phenylpropyl)-6,7,8,9,11,12,13,14,15,16-decahydro-17H-cyclopenta[a]phenanthren-17-one (20). (24.6 mg, 66%, 0.1 mmol scale). Isolated by preparative TLC (hexane: ethyl acetate = 10:1, R_f = 0.6); IR (neat): ν_{max} 3024, 2928, 2857, 1736, 1453, 737, 699 cm^{-1}; ^{1}H NMR (500 MHz, $CDCl_3$) δ 7.33-7.30 (m, 2H), 7.25-7.20 (m, 4H), 7.02 (d, J = 8.0 Hz, 1H), 6.96 (s, 1H), 2.94-2.91 (m, 2H), 2.70 (t, J = 7.8 Hz, 2H), 2.63 (t, J = 7.8 Hz, 2H), 2.56-2.51 (m, 1H), 2.47-2.43 (m, 1H), 2.34-2.30 (m, 1H), 2.21-2.14 (m, 1H), 2.12-2.03 (m, 2H), 2.01-1.95 (m, 3H), 1.70-1.45 (m, 6H), 0.94 (s, 3H); ^{13}C NMR (125 MHz, $CDCl_3$) δ 221.0, 142.4, 139.8, 137.1, 136.3, 129.1, 128.5, 128.3, 125.9, 125.7, 125.3, 50.5, 48.0, 44.3, 38.3, 35.9, 35.6, 34.9, 32.9, 31.6, 29.4, 26.6, 25.8, 21.6, 13.9; HRMS (APCI) calcd for $C_{27}H_{33}O$ [M + H^+], 373.2526; found: 373.2542.

Intra-molecular reductive

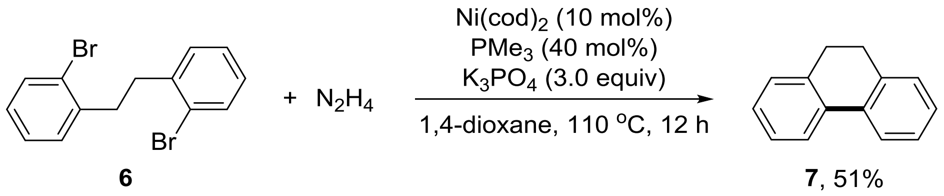

Supplementary Figure 2 Intra-molecular reductive cyclization to give 9,10-dihydrophenanthrene.

The procedure was similar to the homo-couplings. **9,10-Dihydrophenanthrene (7)**. (18.4 mg, 51%). Isolated by preparative TLC (hexane, R_f = 0.7); ^{1}H NMR (400 MHz, $CDCl_3$) δ 7.82 (d, J = 7.4 Hz, 2H), 7.39-7.35 (m, 2H), 7.31-7.28 (m, 4H), 2.95-2.94 (m, 4H); ^{13}C NMR (100 MHz, $CDCl_3$) δ 137.5, 134.6, 128.2, 127.5, 127.0, 123.8, 29.1; EI-MS (m/z): 180.1. The spectroscopic data for this product match the literature data.[17]

The kinetics profile of the model reaction

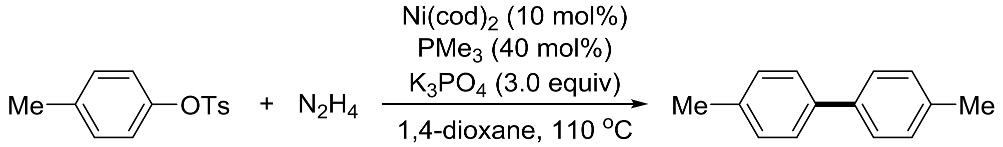

1a **3a**

The procedure was similar to the homo-couplings. Seven parallel tubes were charged with the same starting materials and reacted simultaneously. The reaction tube was quickly cooled to 0°C with ice as indicated by time in Supplementary Figure 3. The crude mixture was directly filtered through a pad of silica and analyzed by ^{1}H NMR using mesitylene as an internal standard. As we can see, the model reaction went to completion about 2 h under 110 °C in 1,4-dioxane and the desired product **3b** was observed in 91% yield. The yield of **3b** did not decrease with the extension of time to 12 h.

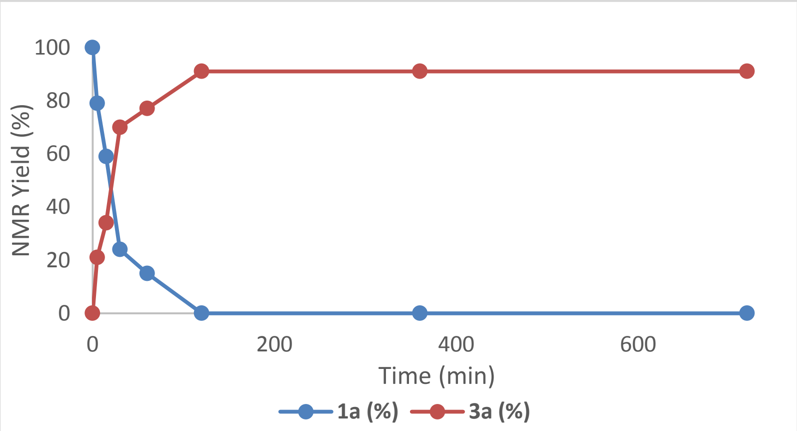

Time (min)	Yield (%) 1a	3a
0	100	0
5	79	21
15	59	34
30	24	70
60	15	77
120	0	91
360	0	91
720	0	91

Supplementary Figure 3 The kinetics profile of the model reaction

Mechanistic studies

The phenol tosylate and hydrazine were selected as the coupling partners under the standard conditions and GC was used to monitor the rection. After the reaction was performed for 1 h, the reaction tube was quickly cooled to 0°C with ice. The crude mixture was directly filtered through a pad of silica and analyzed by GC immediately. Similarly, the crude mixture was analyzed when the reaction was performed for 3h. In both cases, aryl hydrazine **21** was not observed. Moreover, when phenyl-hydrazine **21** was tested under the standard conditions, no desired product **3b** was detected. These results ruled out the pathway involving the homo-coupling of aryl hydrazine (see Supplementary Figure 4a & 4b).

To investigate if $L_nNi\text{-}NH_2NH_2$ species is involved in the reaction, the control experiments were then carried out. Pre-mixing equimolar $Ni(cod)_2/Me_3P$/hydrazine in dioxane for 1 h, then adding phenyl tosylate and reacting at 110 °C for 12 h, the desired **3b** was obtained in 29% yield . If equimolar $Ni(cod)_2/Me_3P$/ phenyl tosylate was pre-mixed first in dioxane for 1 h, then adding hydrazine and reacting under the same conditions, 31% yield of **3b** was

observed. These results suggested that changing the sequence of hydrazine or phenyl tosylate made no difference regarding the yield, and $L_nNi\text{-}NH_2NH_2$ species may not be involved in starting the reaction. (Supplementary Figure 4c & 4d).

Preparation of *trans*-[PhNi(II)Br(Me$_3$P)$_2$] complex 22. The procedure was same as the literature.[27] To a stirred suspension of $Ni(cod)_2$ (1.0 mmol) in diethyl ether (30.0 mL), cooled to -70°C, an excess of PhBr (1.3 mmol) and PMe_3 (2.0 mmol) were added. The mixture was warmed and stirred at room temperature for 6 h. The resulting mixture was filtered to give a clear brown solution. After partial removal of the solvent and cooling overnight, the yellow-brown crystals of the desired nickel complex **22** were collected (197 mg, 54%). (Supplementary Figure 4e). ^{1}H NMR (500 MHz, CD_2Cl_2) δ 7.28 (d, J = 7.2 Hz, 2H), 6.90 (t, J = 7.2 Hz, 2H), 6.72 (t, J = 6.8 Hz, 1H), 1.05 (s, 18H); ^{13}C NMR (125 MHz, CD_2Cl_2) δ 156.2 (t, $J_{C\text{-}P}$ = 32.0 Hz), 135.7, 126.6, 121.4, 13.3; ^{31}P NMR (202 MHz, CD_2Cl_2) δ -15.4. The spectroscopic data for this product match the literature data.[27] Stoichiometric reaction of this nickel complex with an aryl iodide in the presence of hydrazine (without adding base) in 1,4-dioxane at 110°C for 12 h delivered both the homo- and cross-coupling products **3b** (49% yield), **3f** (17% yield) and **10g** (38% yield) (Supplementary Figure 4f). Theoretically, one molecule of hydrazine can provide at least 2 electrons (H_2 was detected by GC), thus explaining that 0.5 equiv of hydrazine is able to reduce 1.0 equiv of aryl electrophile to give the corresponding coupling product. At this stage, we speculated that hydrazine played a similar role as zinc in the reductive aryl homo-couplings as reported by Percec.[28]

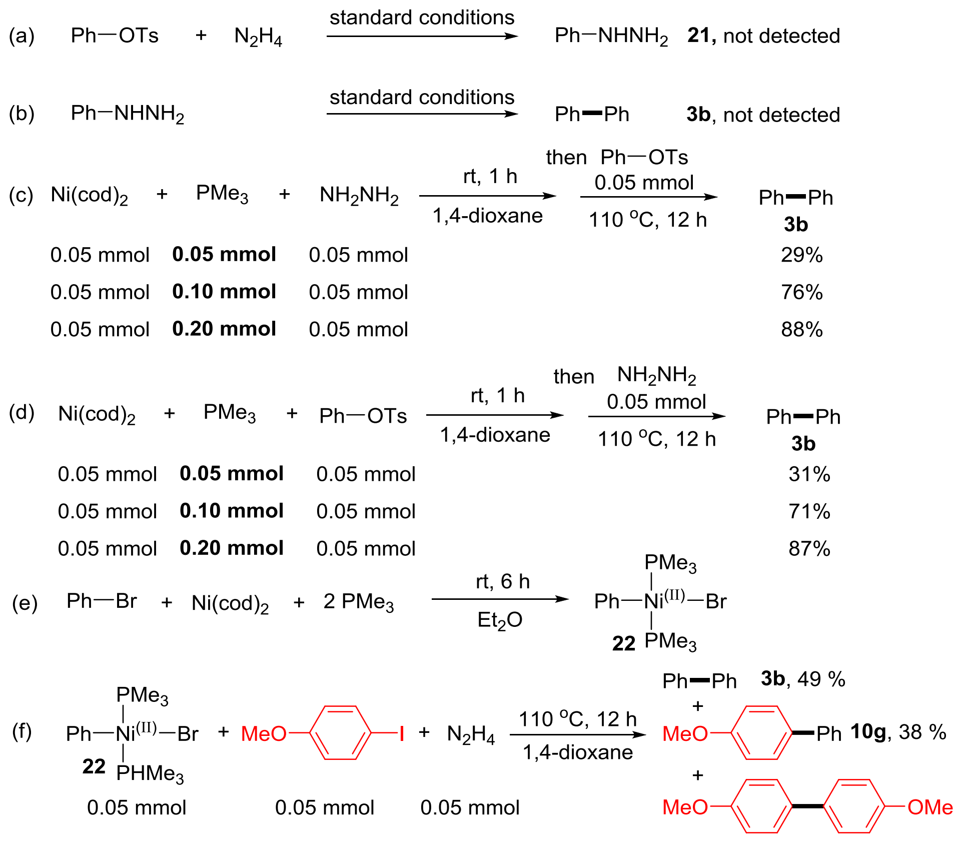

Supplementary Figure 4 The mechanistic studies. **(a)** Reaction of phenyl tosylate with hydrazine did not give phenyl hydrazine; **(b)** Reaction of phenyl hydrazine under the standard conditions did not give the biphenyl product; **(c)** Pre-mix different molar $Ni(cod)_2/Me_3P$/hydrazine in 1,4-dioxane for 1 h, then add the phenyl tosylate; **(d)** Pre-mix different molar $Ni(cod)_2/Me_3P$/ phenyl tosylate in 1,4-dioxane for 1 h, then add the hydrazine; **(e)** Preparation of *trans*-$[PhNi(II)Br(Me_3P)_2]$ complex; **(f)** Stoichiometric reaction of nickel complex with an aryl iodide in the presence of hydrazine

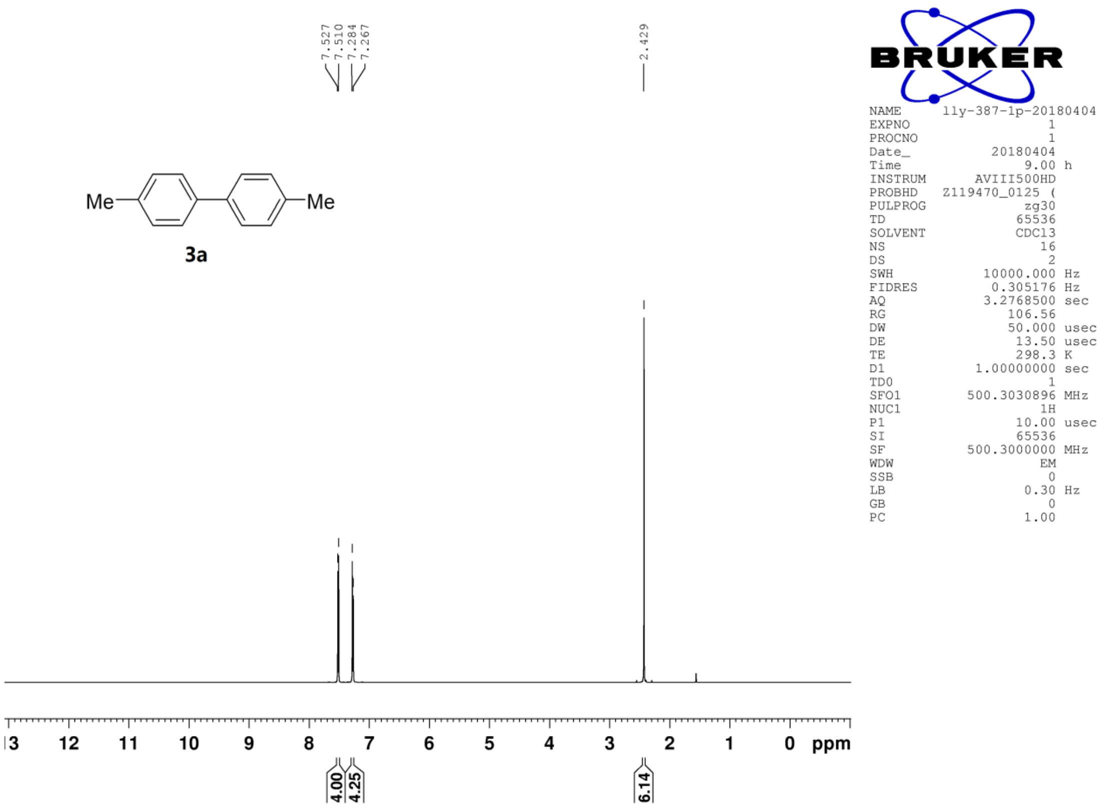

Supplementary Figure 5. ^{1}H NMR spectra for compound **3a**

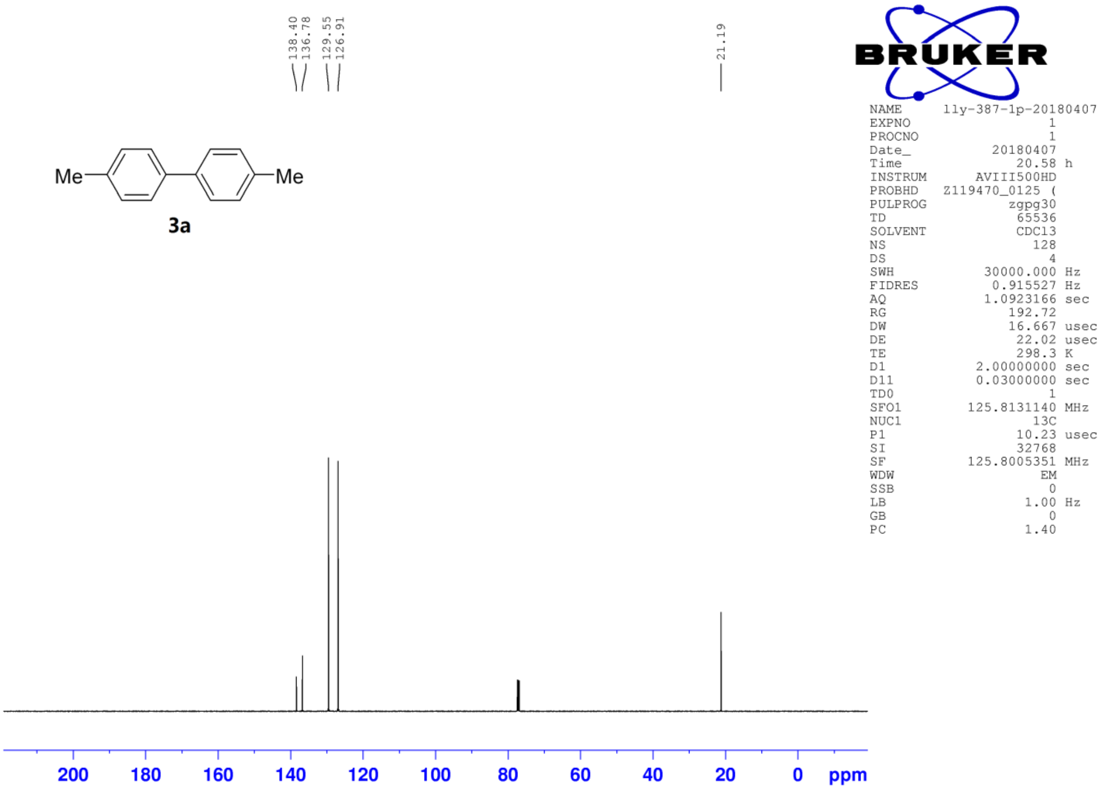

Supplementary Figure 6. ^{13}C NMR spectra for compound **3a**

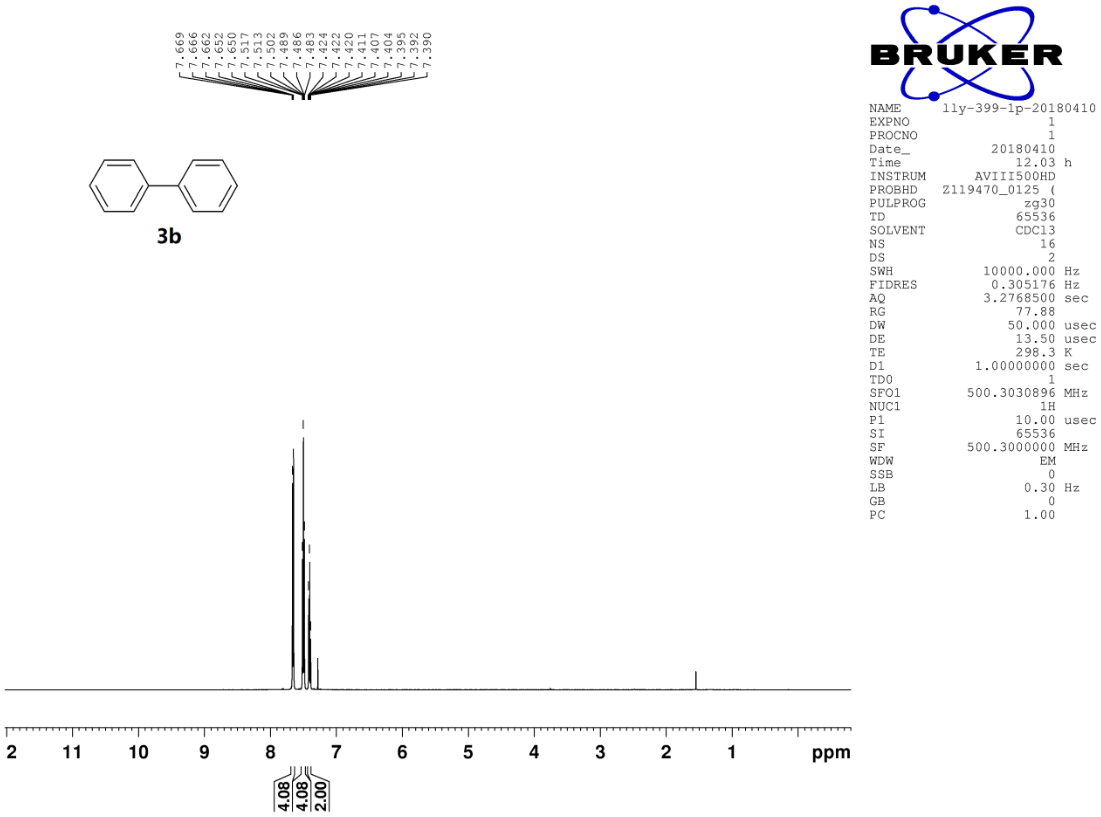

Supplementary Figure 7. ^{1}H NMR spectra for compound **3b**

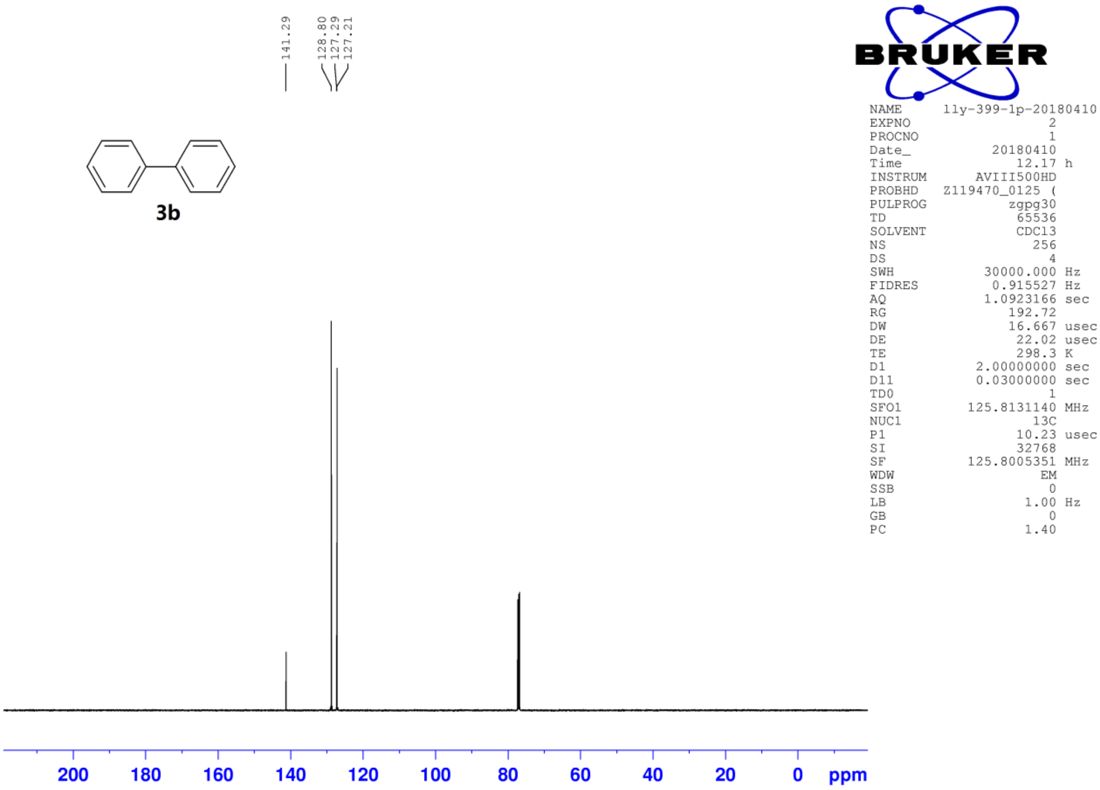

Supplementary Figure 8. ^{13}C NMR spectra for compound **3b**

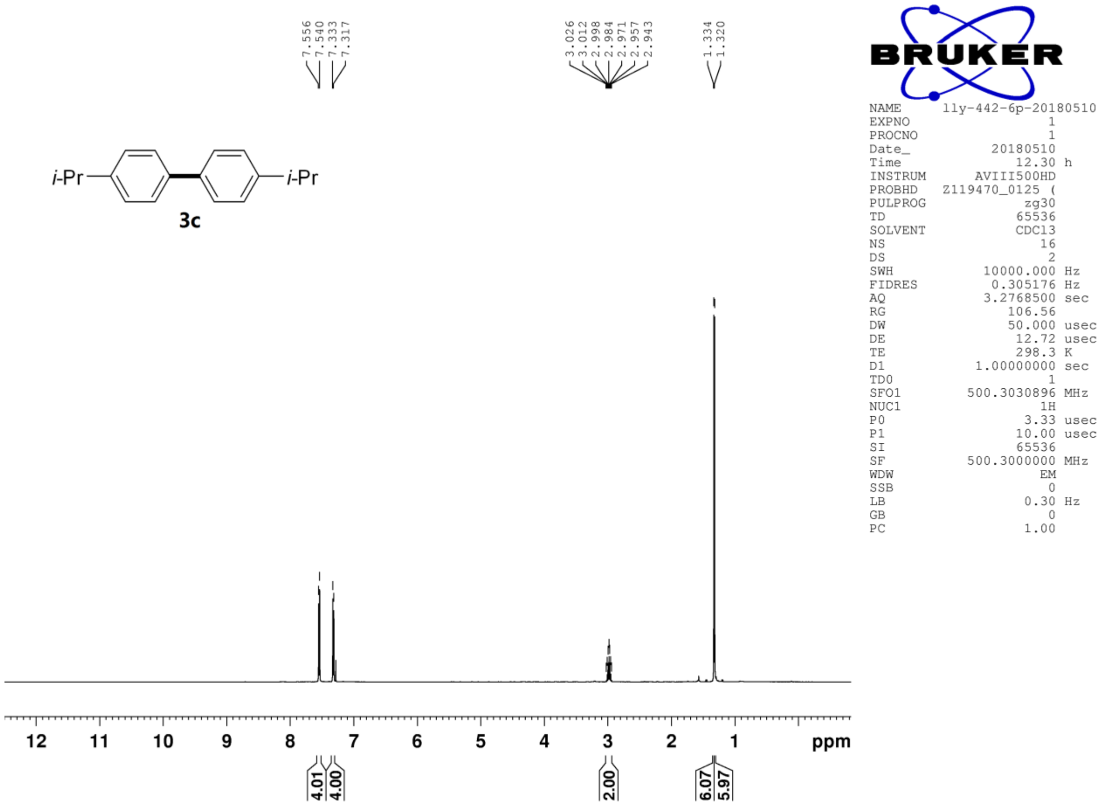

Supplementary Figure 9. ^{1}H NMR spectra for compound **3c**

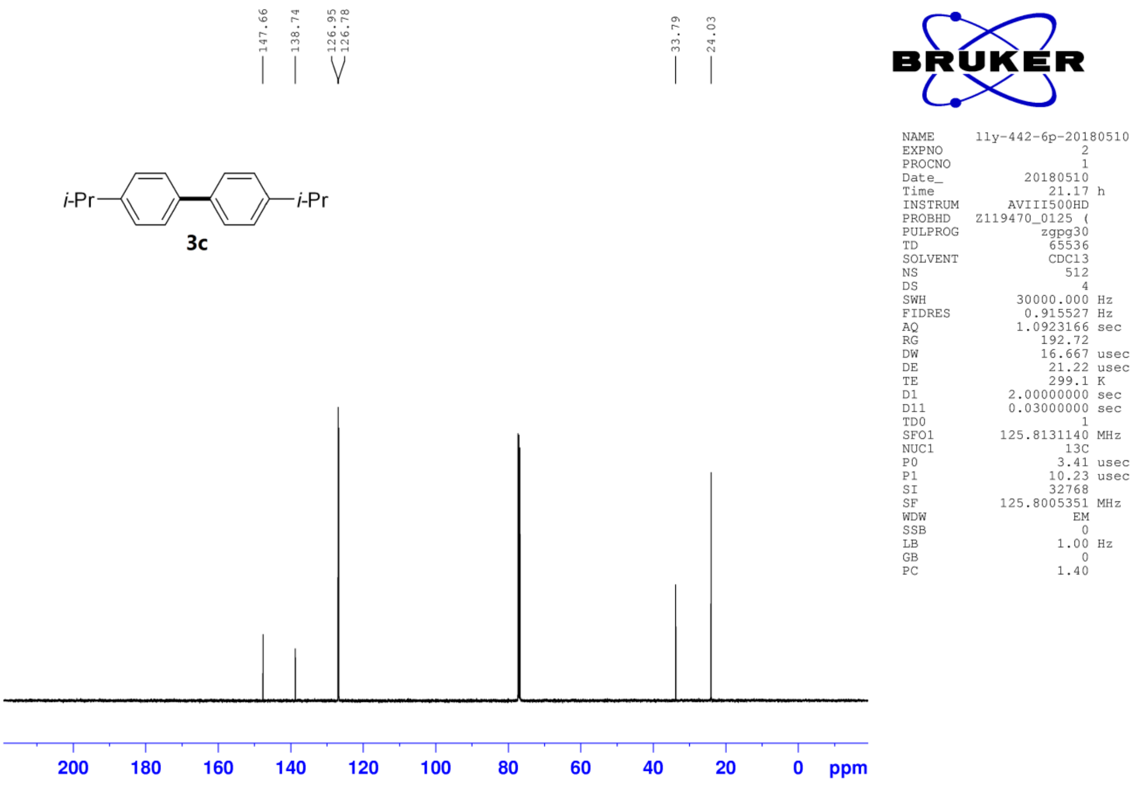

Supplementary Figure 10. ^{13}C NMR spectra for compound **3c**

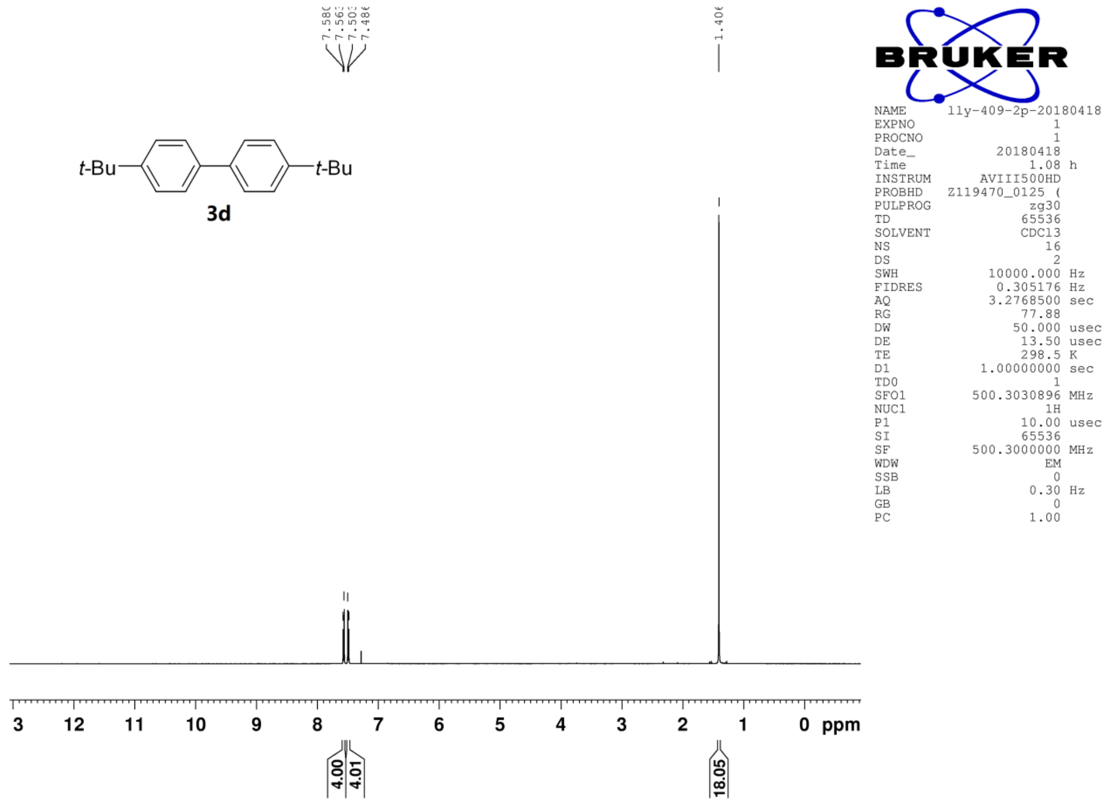

Supplementary Figure 11. ^{1}H NMR spectra for compound **3d**

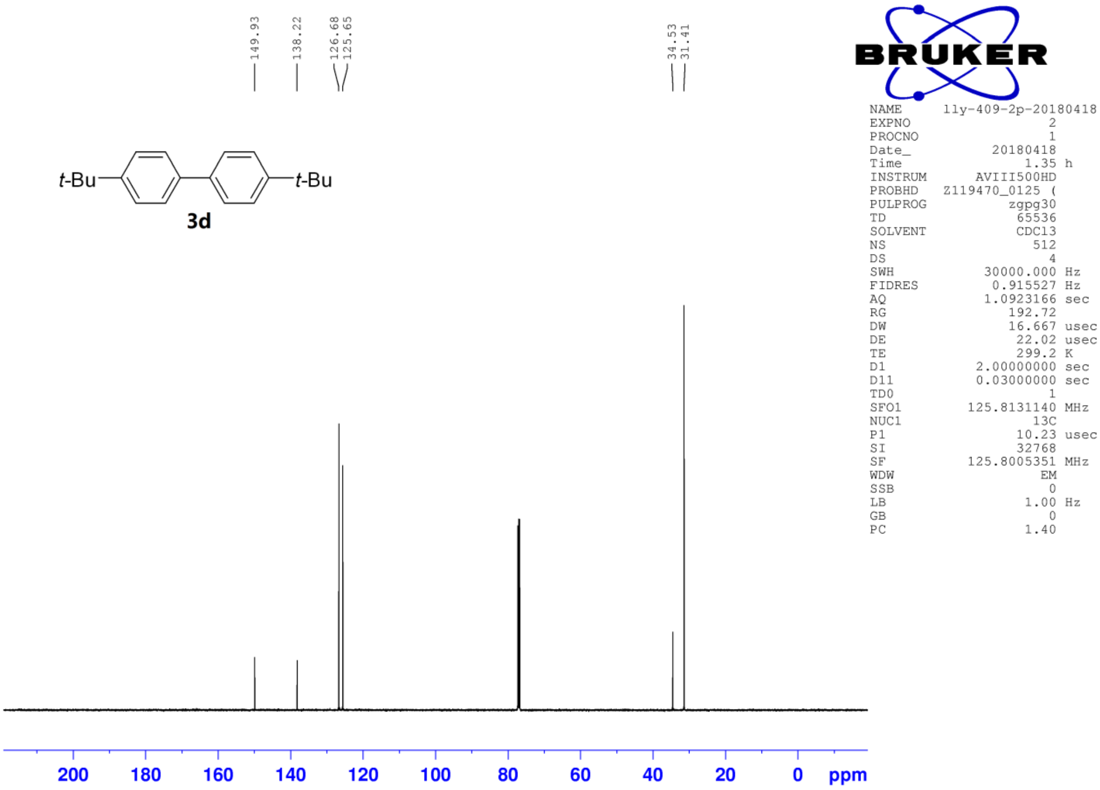

Supplementary Figure 12. ^{13}C NMR spectra for compound **3d**

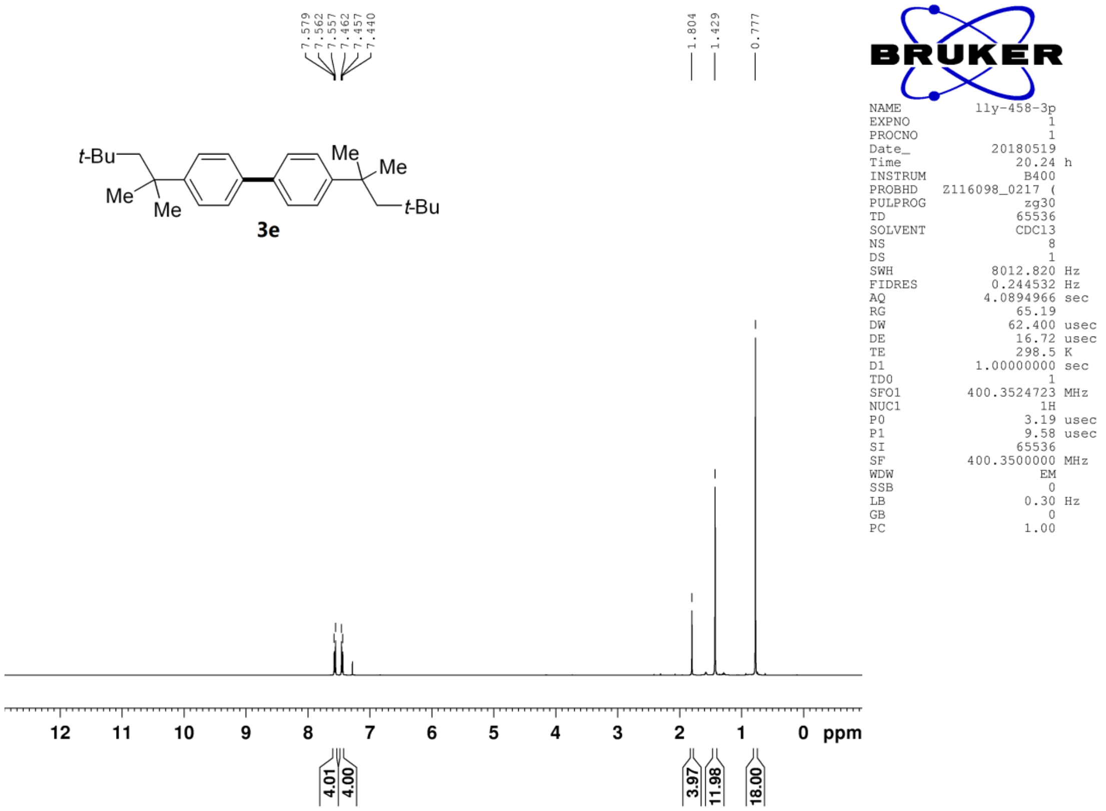

Supplementary Figure 13. ^{1}H NMR spectra for compound **3e**

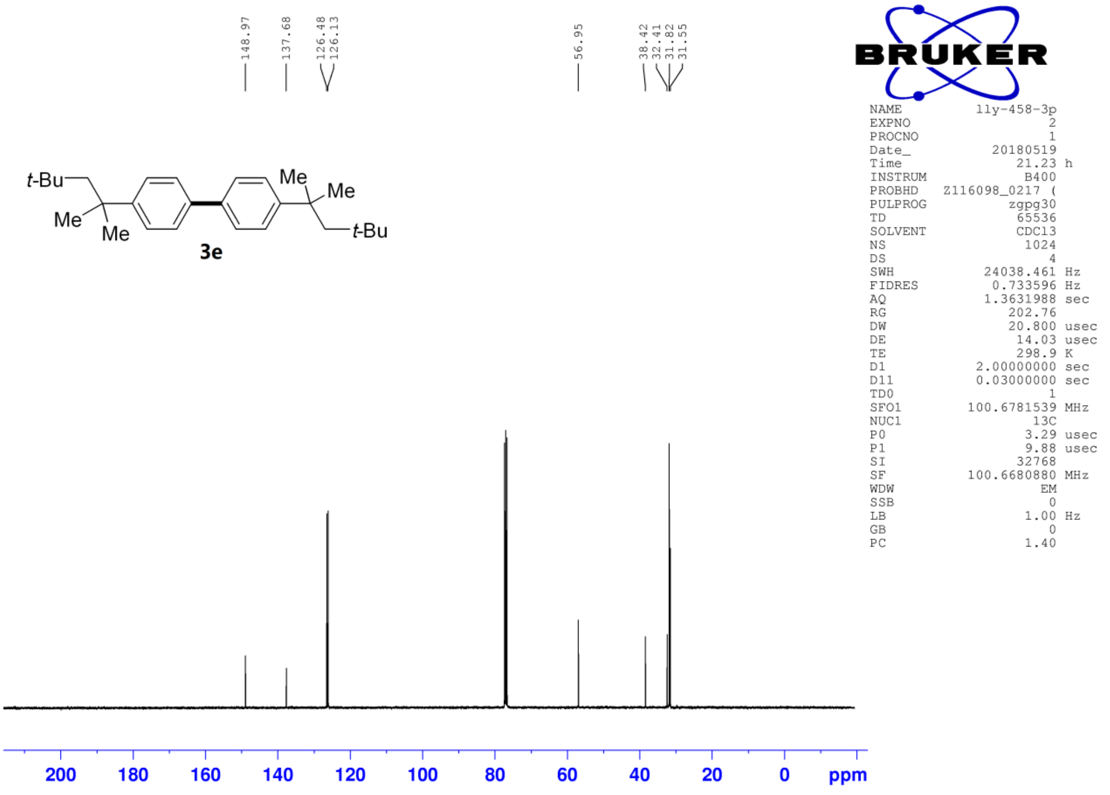

Supplementary Figure 14. ^{13}C NMR spectra for compound **3e**

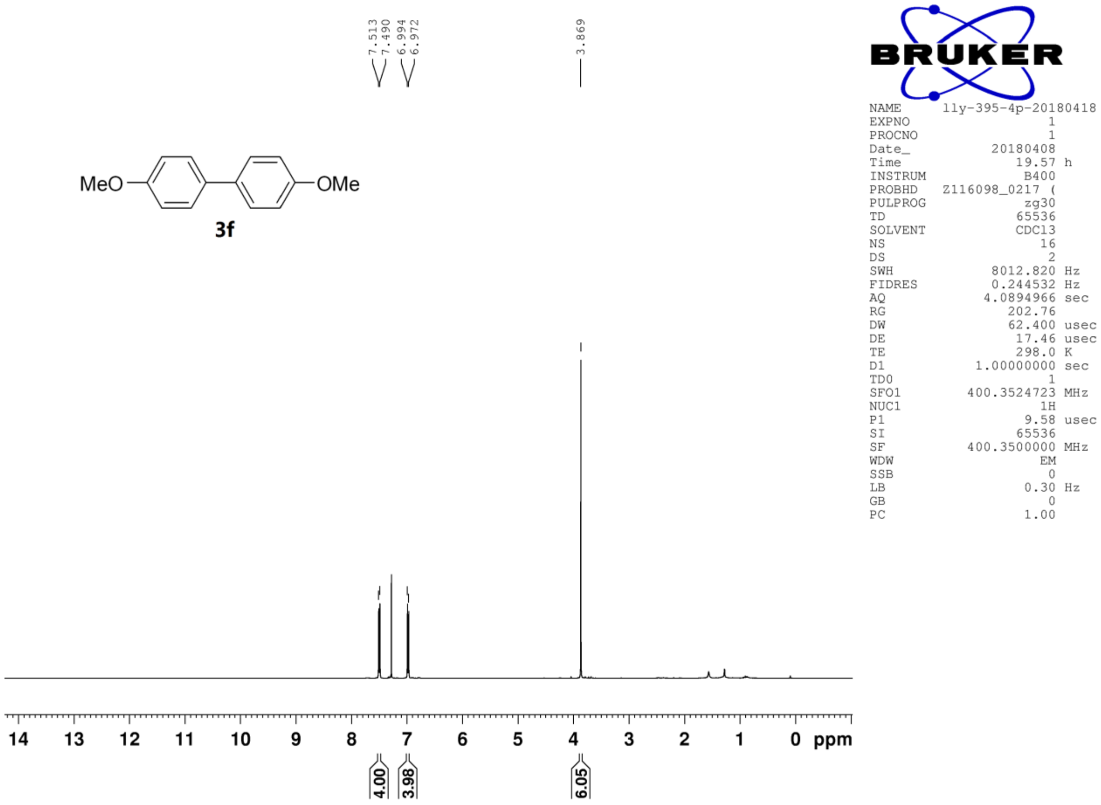

Supplementary Figure 15. ^{1}H NMR spectra for compound **3f**

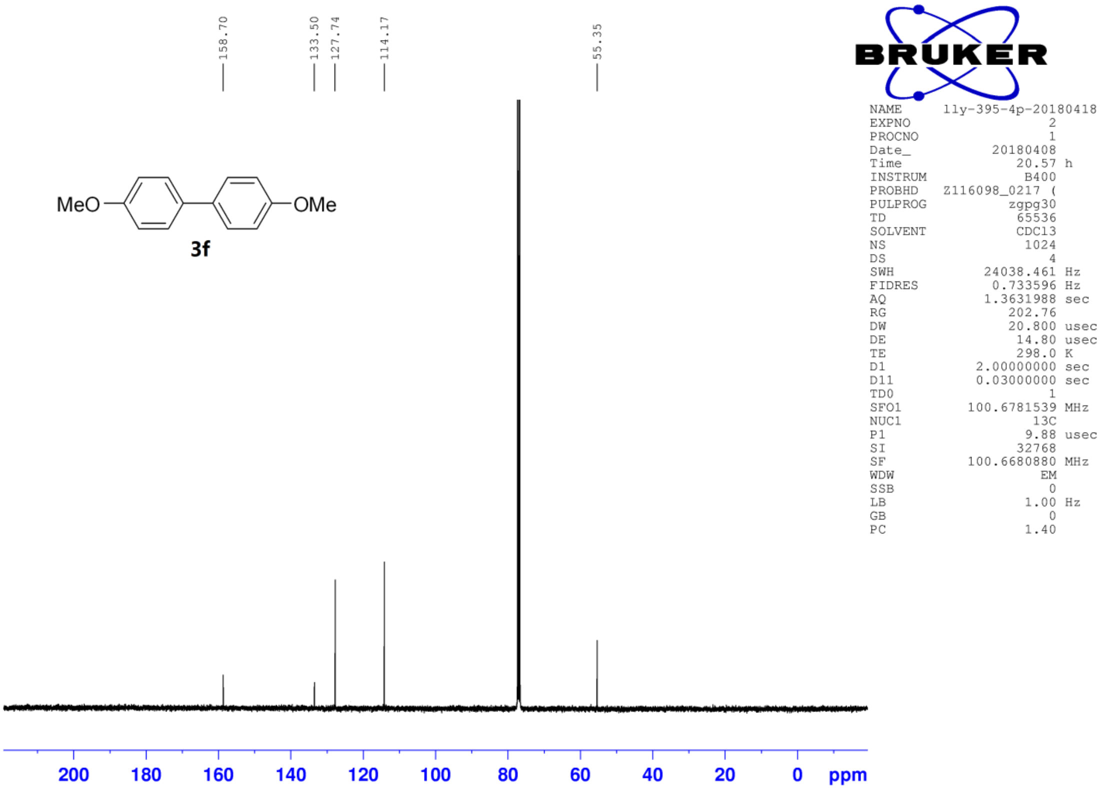

Supplementary Figure 16. ^{13}C NMR spectra for compound **3f**

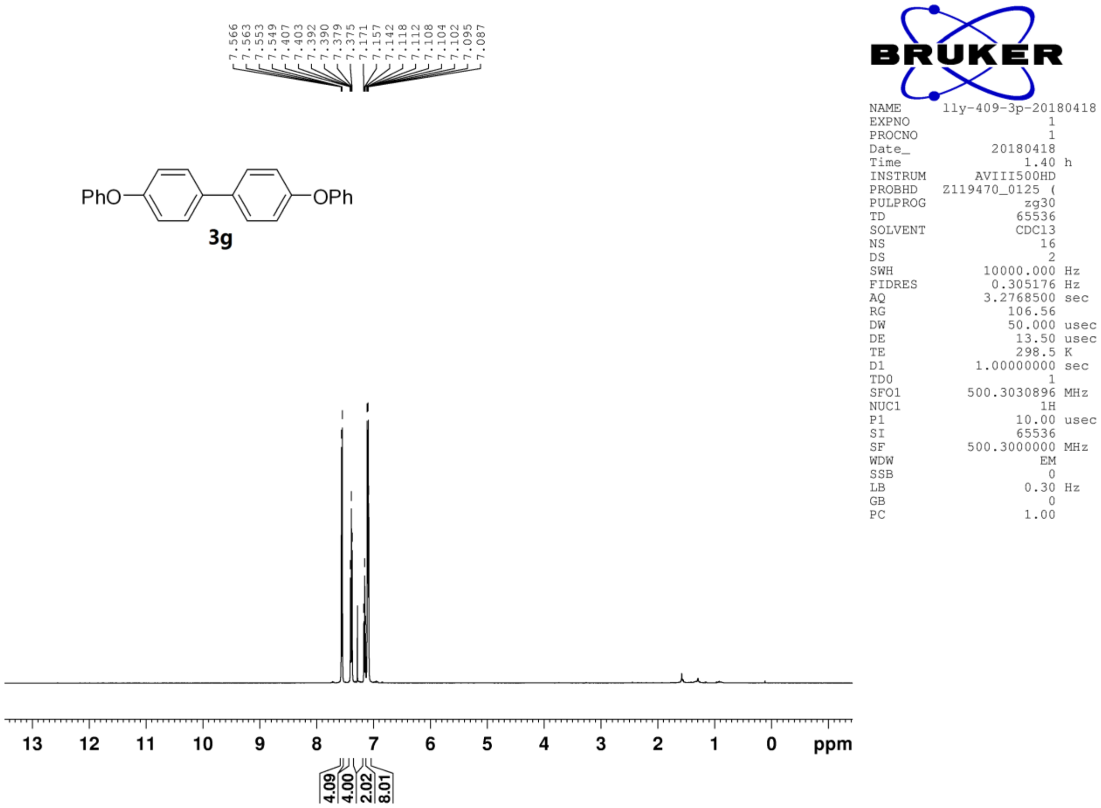

Supplementary Figure 17. ^{1}H NMR spectra for compound **3g**

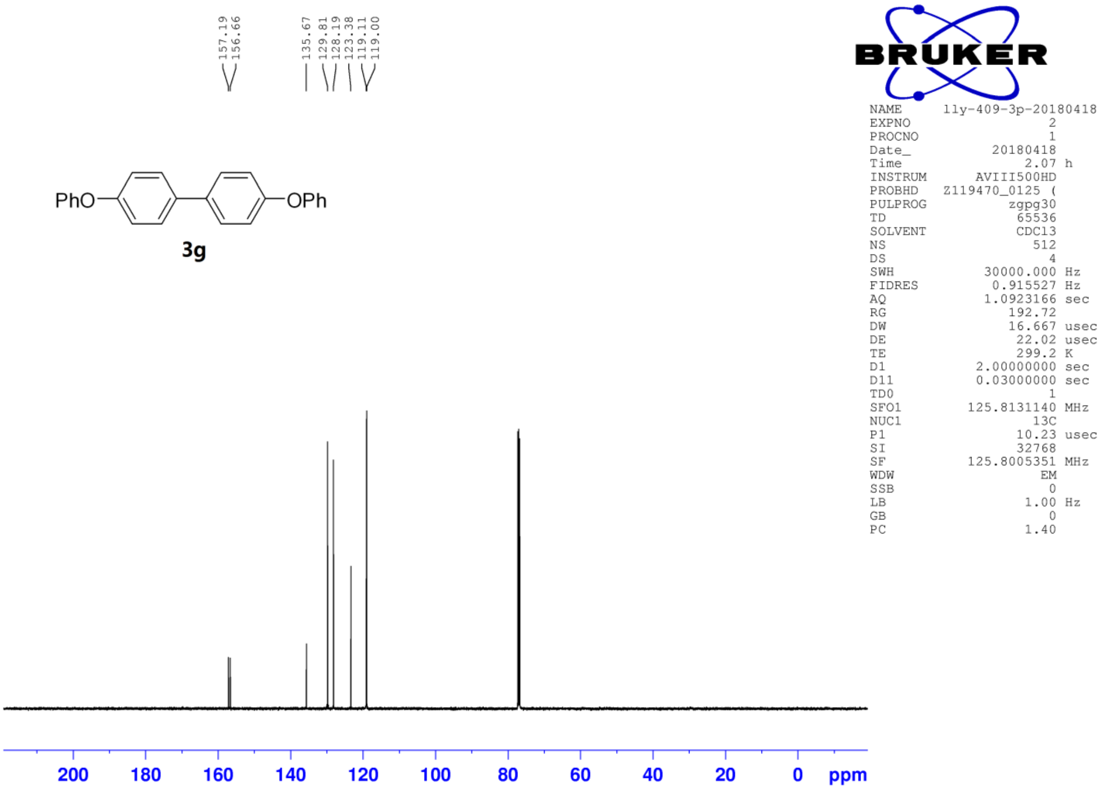

Supplementary Figure 18. ^{13}C NMR spectra for compound **3g**

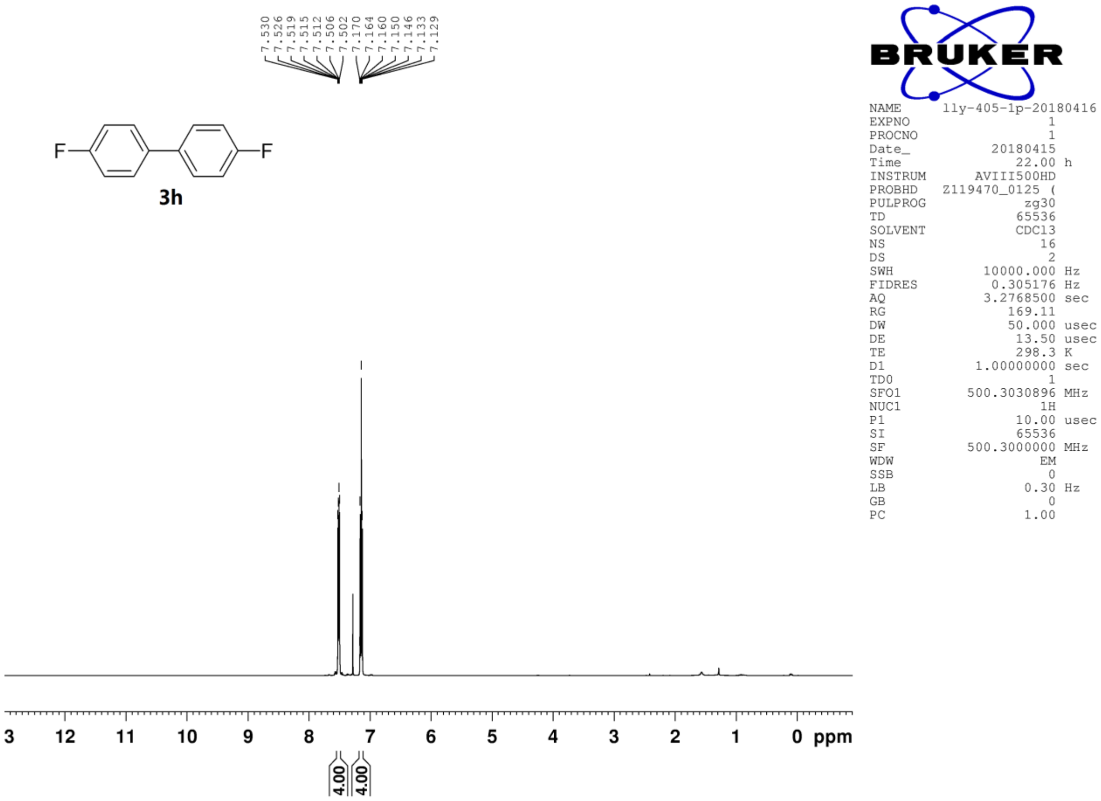

Supplementary Figure 19. ^{1}H NMR spectra for compound **3h**

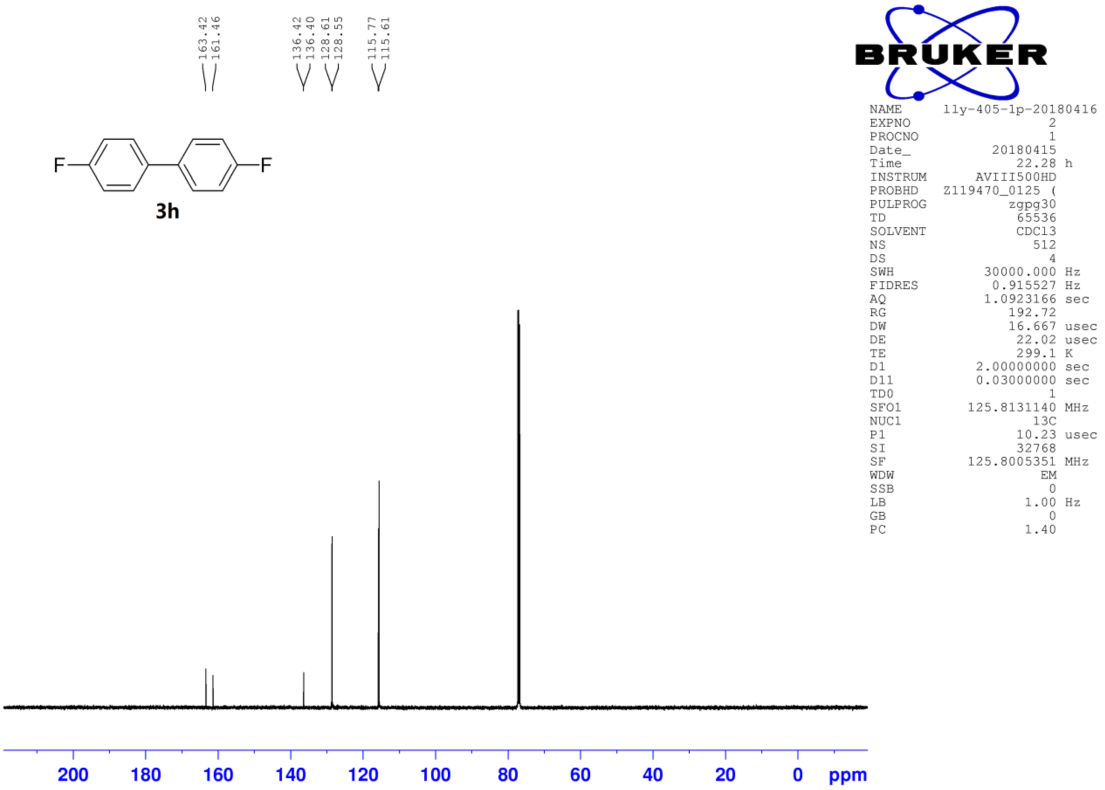

Supplementary Figure 20. ^{13}C NMR spectra for compound **3h**

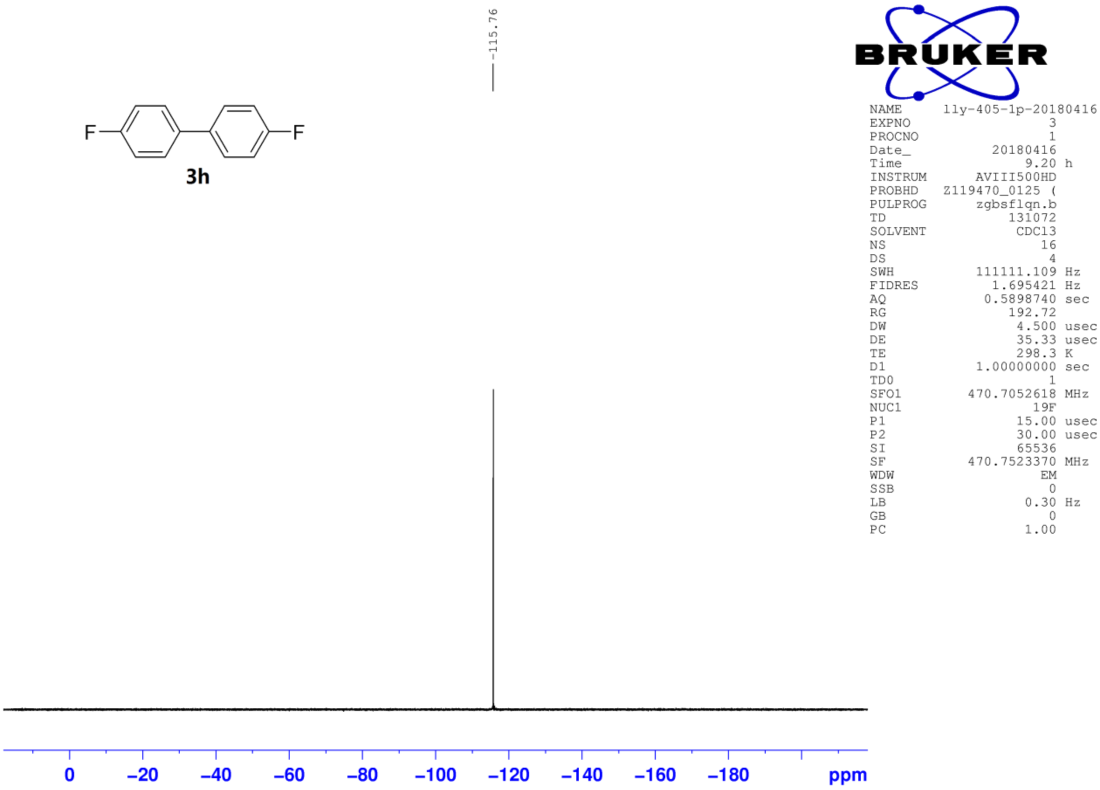

Supplementary Figure 21. ^{19}F NMR spectra for compound **3h**

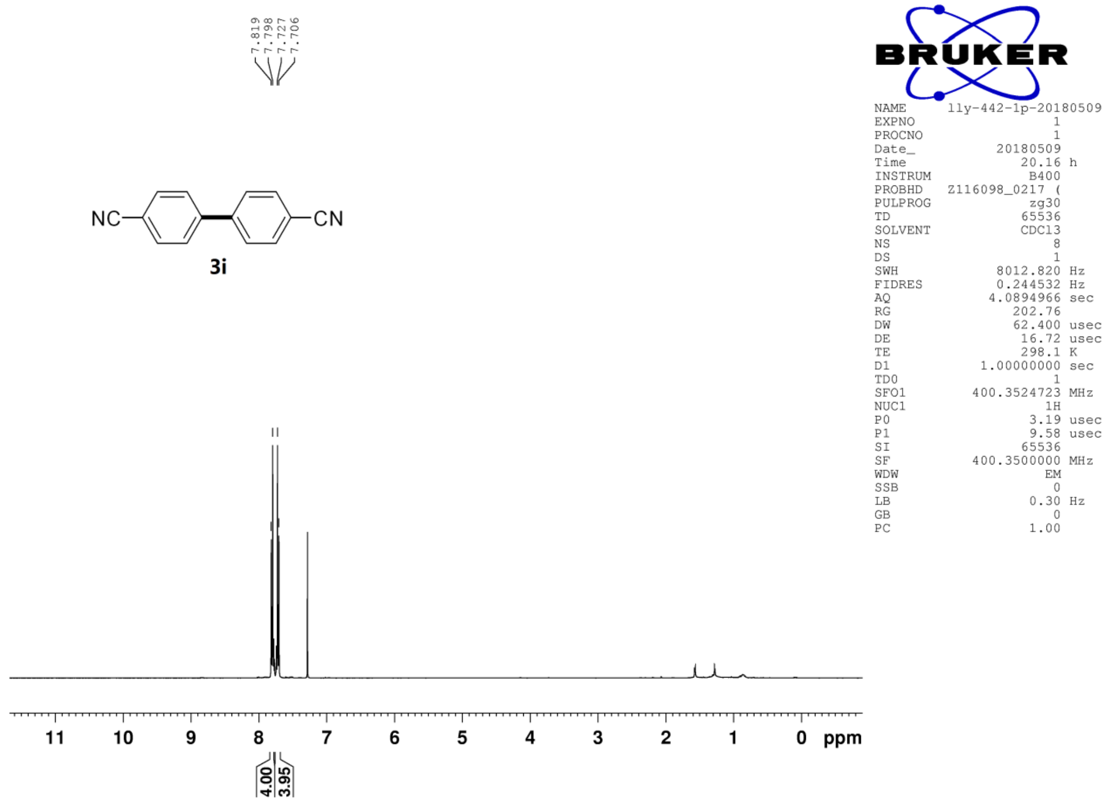

Supplementary Figure 22. ^{1}H NMR spectra for compound **3i**

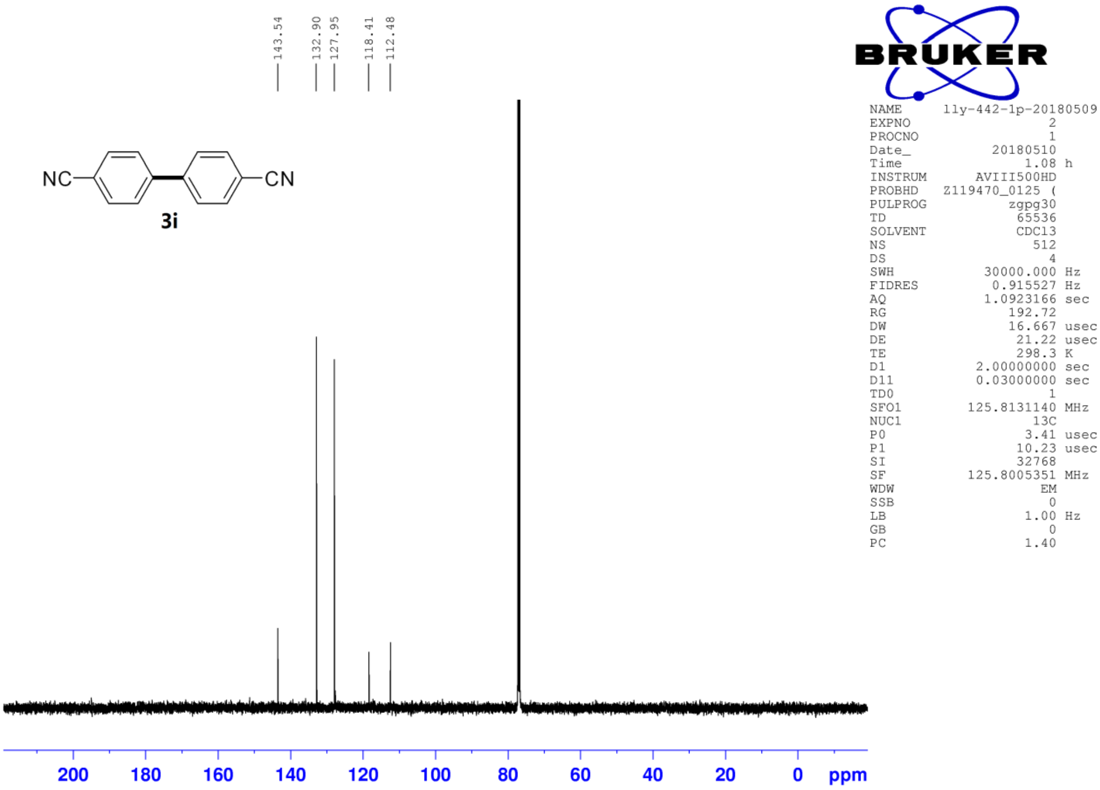

Supplementary Figure 23. ^{13}C NMR spectra for compound **3i**

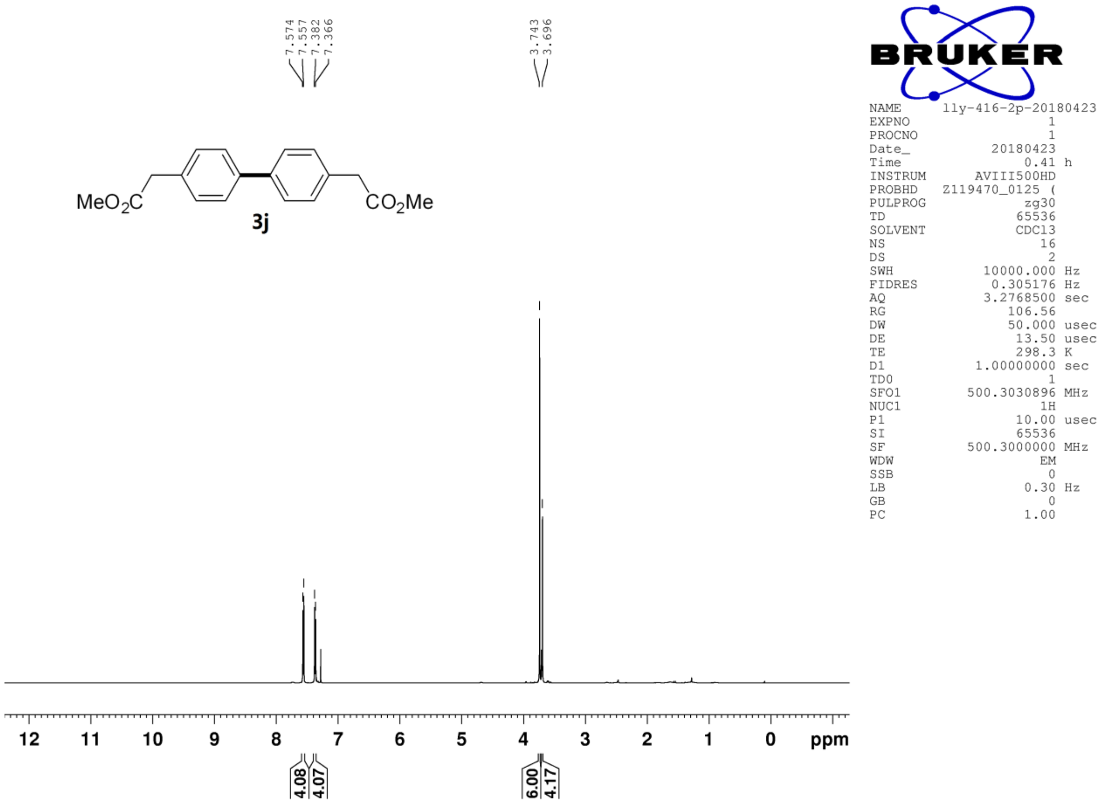

Supplementary Figure 24. ^{1}H NMR spectra for compound **3j**

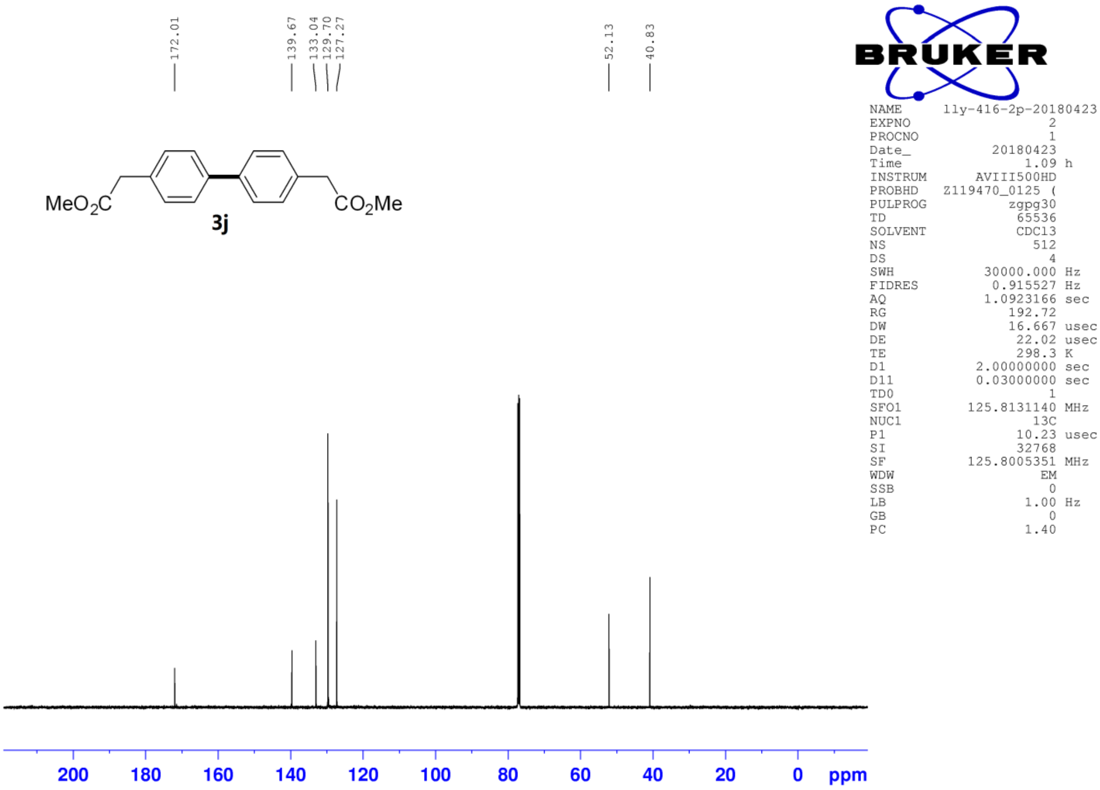

Supplementary Figure 25. ^{13}C NMR spectra for compound **3j**

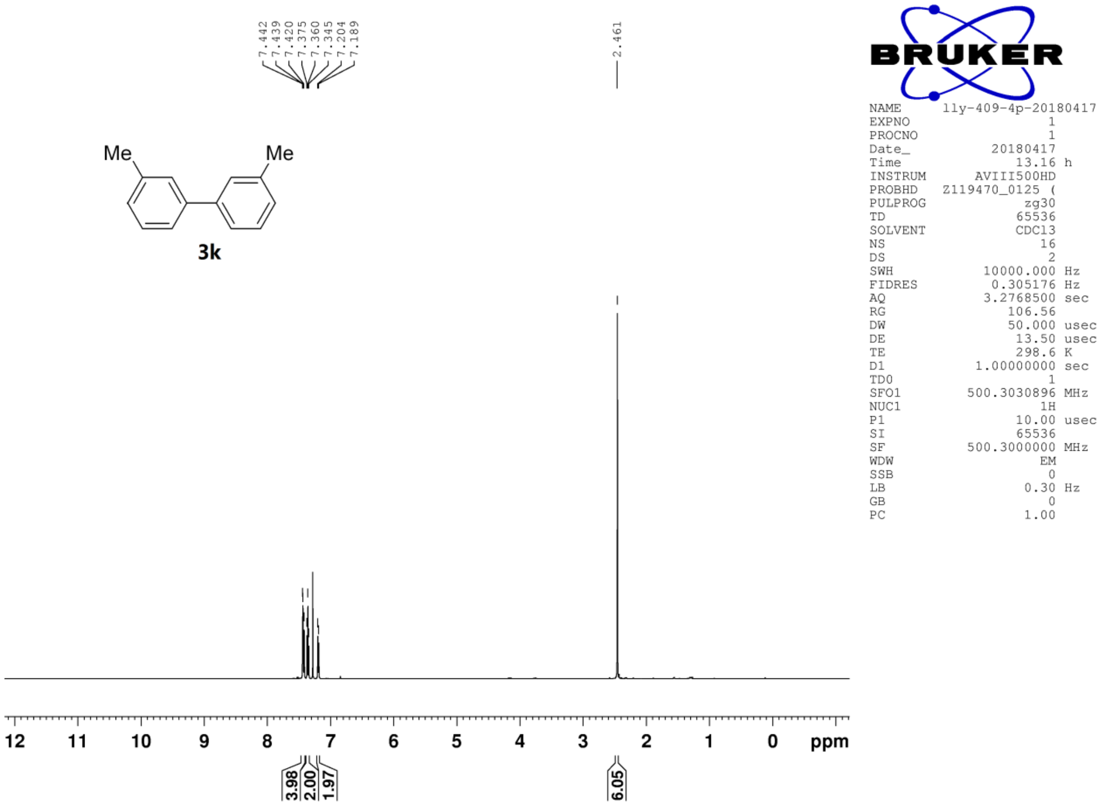

Supplementary Figure 26. ^{1}H NMR spectra for compound **3k**

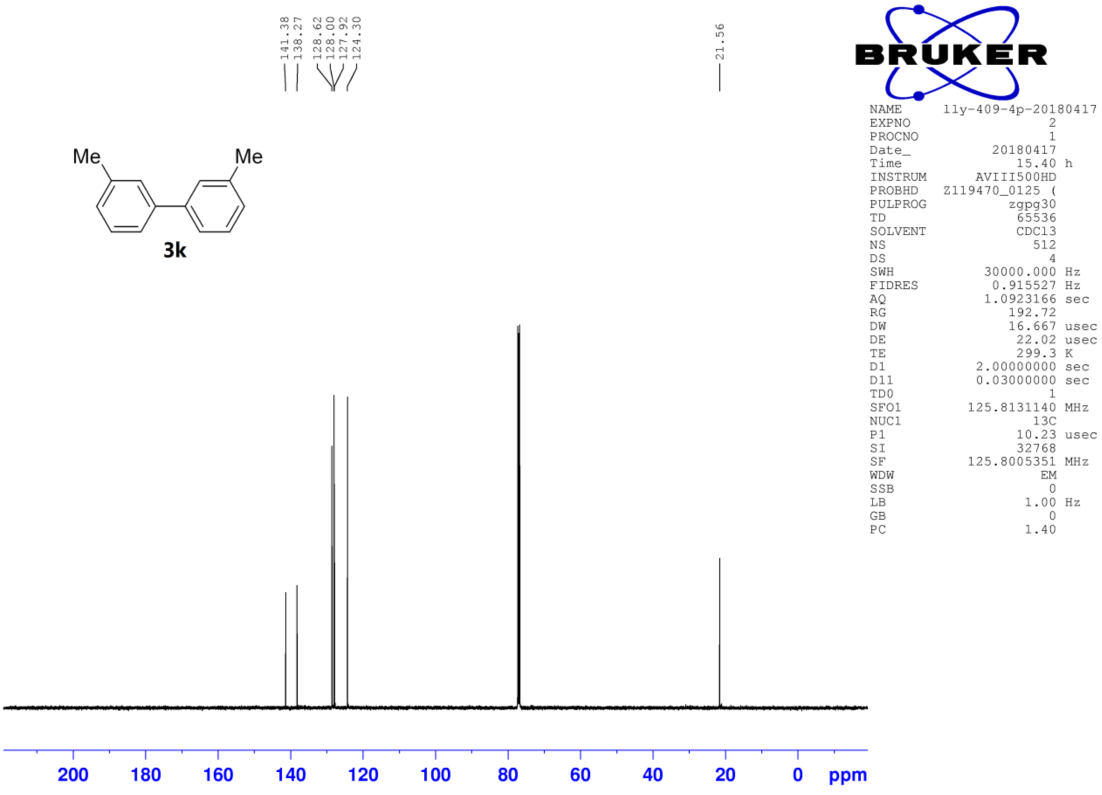

Supplementary Figure 27. ^{13}C NMR spectra for compound **3k**

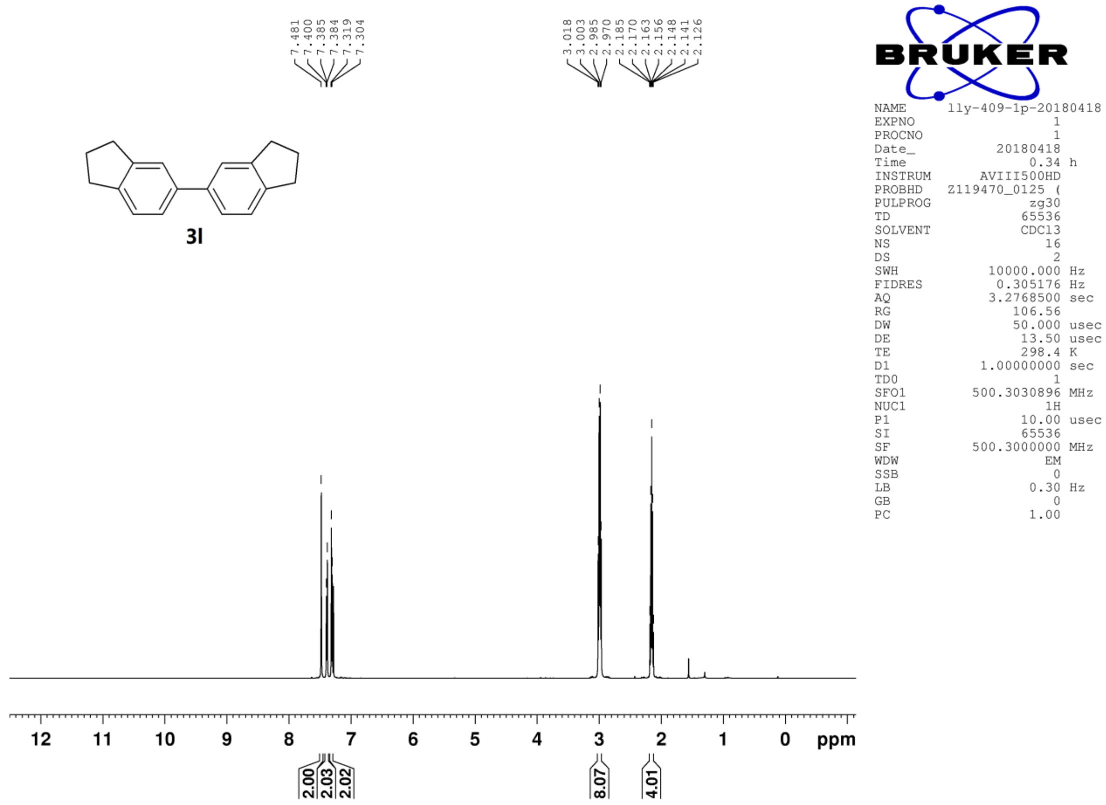

Supplementary Figure 28. ^{1}H NMR spectra for compound **3l**

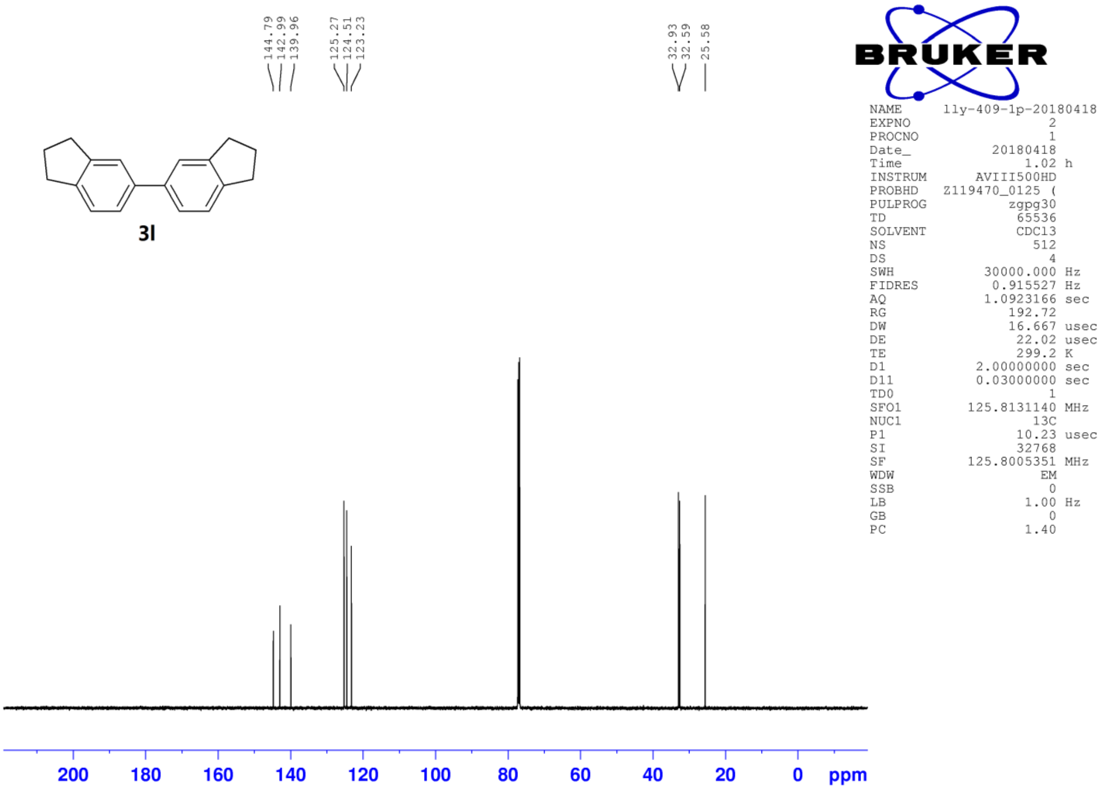

Supplementary Figure 29. ^{13}C NMR spectra for compound **3l**

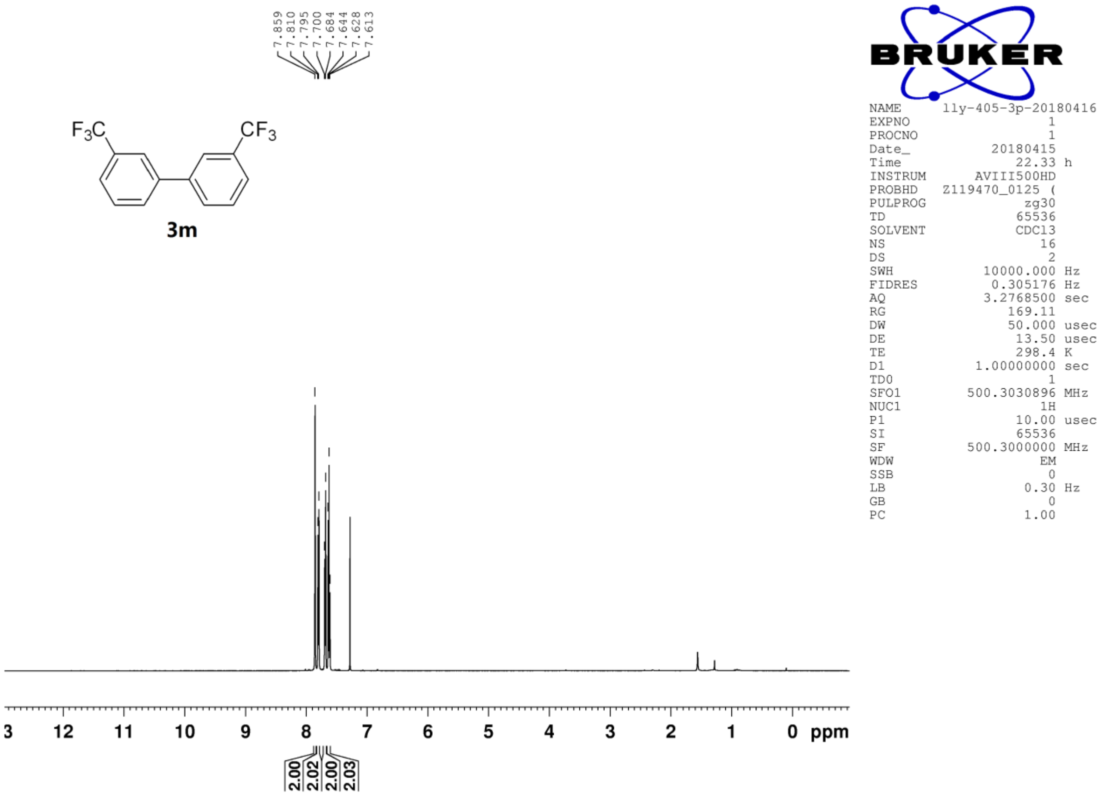

Supplementary Figure 30. ^{1}H NMR spectra for compound **3m**

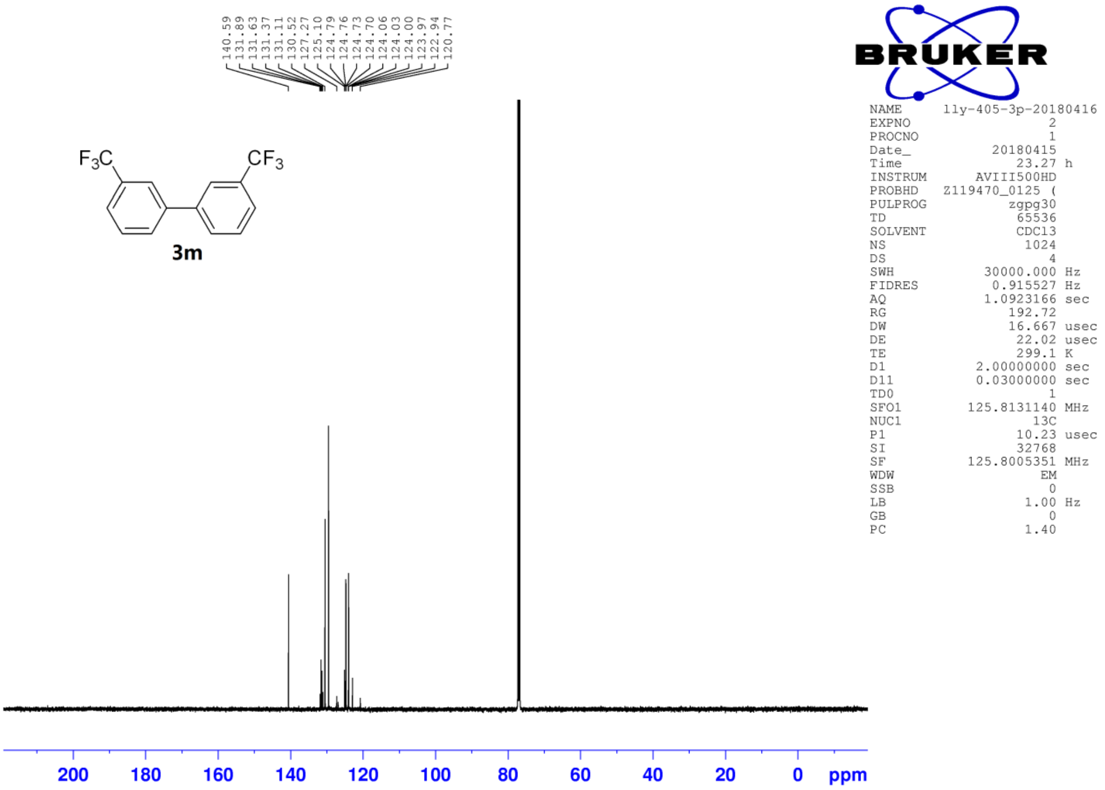

Supplementary Figure 31. ^{13}C NMR spectra for compound **3m**

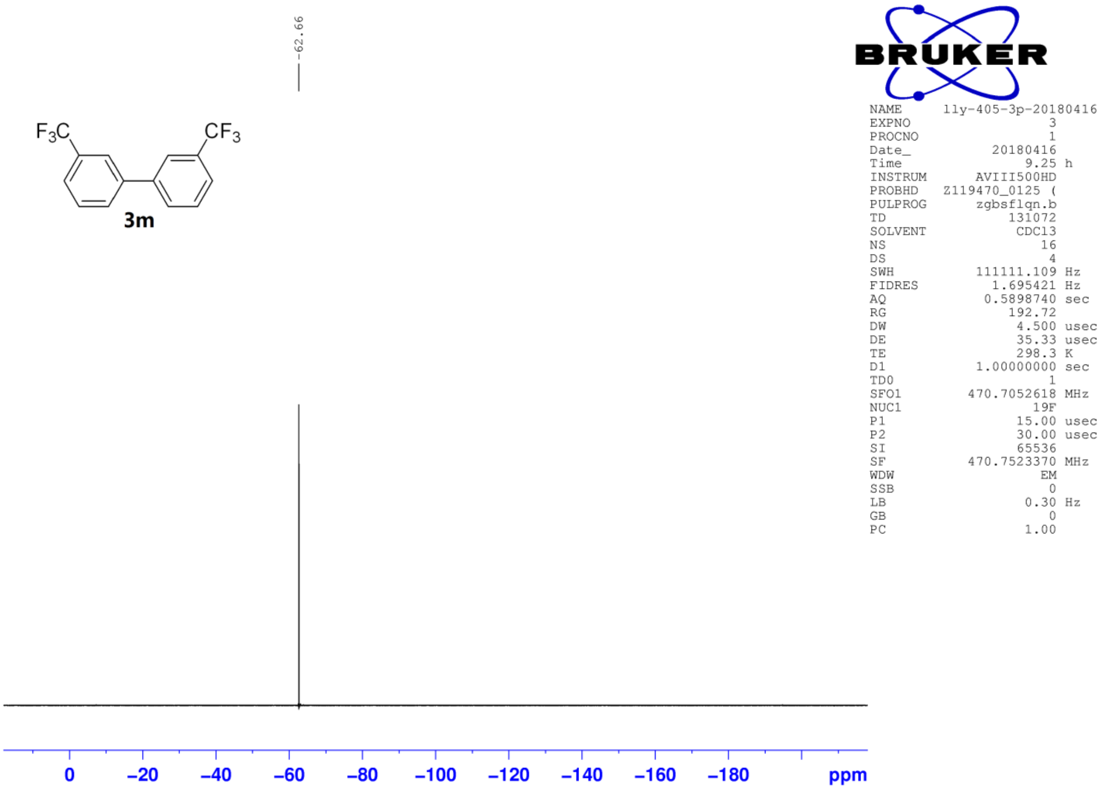

Supplementary Figure 32. ^{19}F NMR spectra for compound **3m**

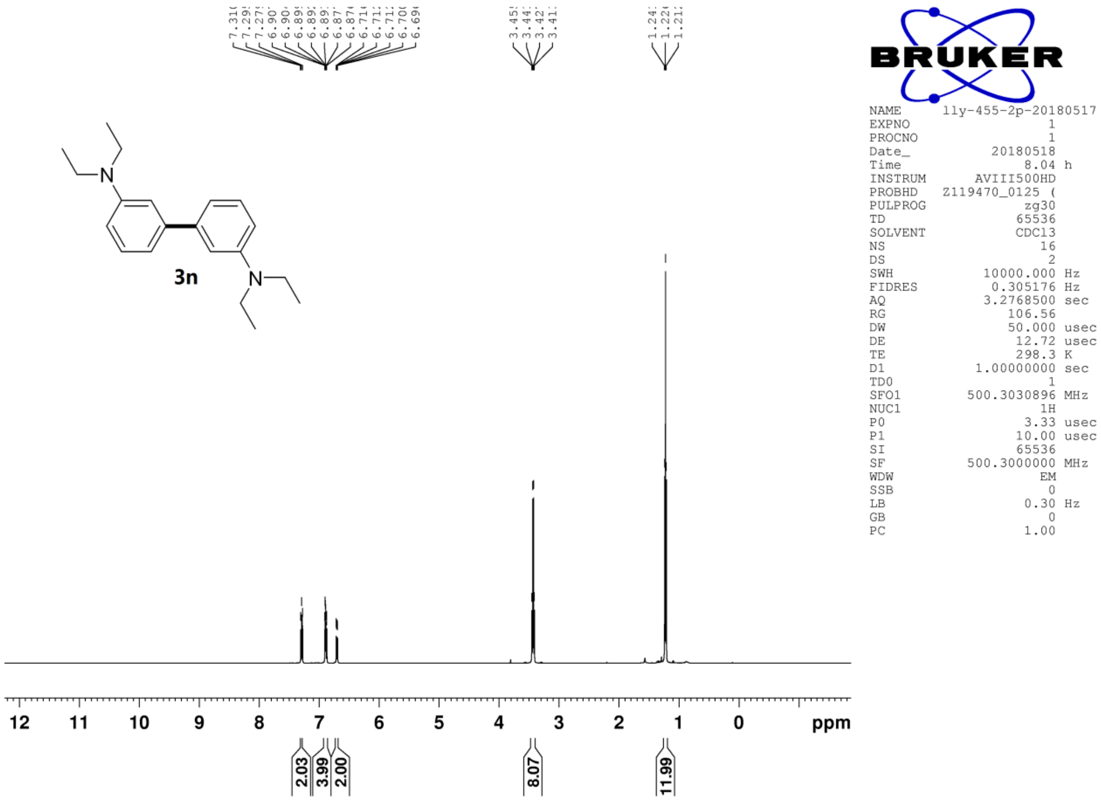

Supplementary Figure 33. ^{1}H NMR spectra for compound **3n**

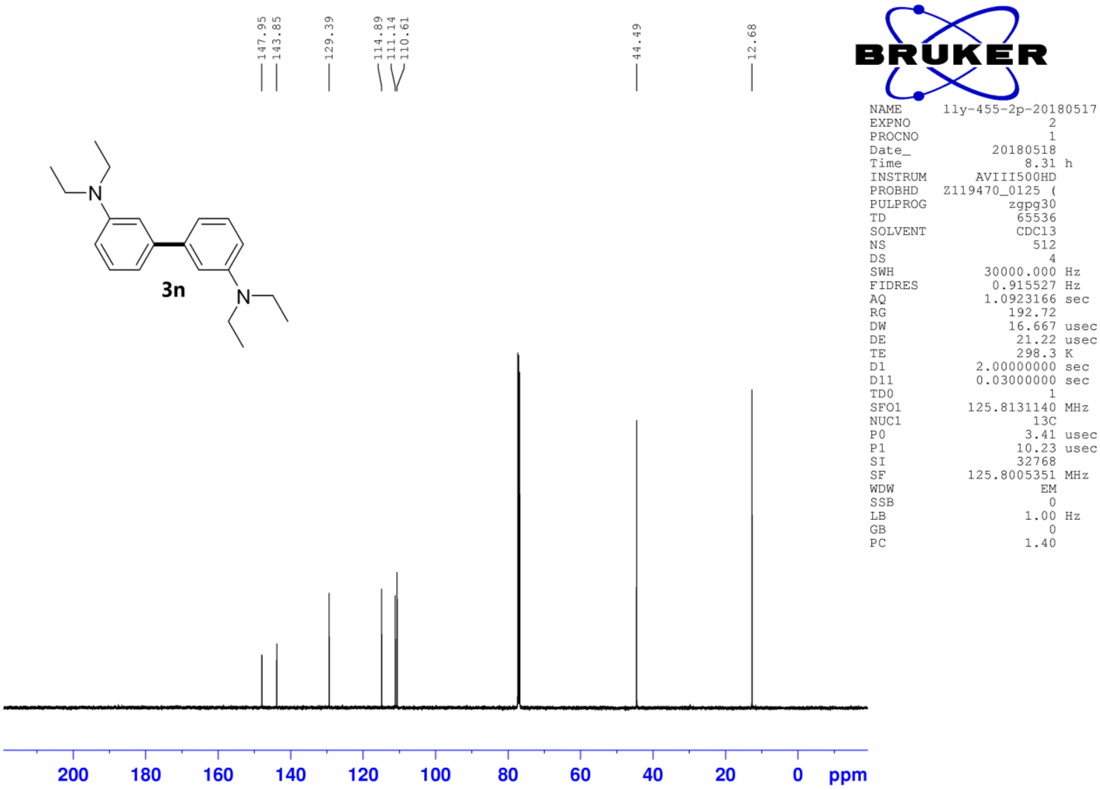

Supplementary Figure 34. ^{13}C NMR spectra for compound **3n**

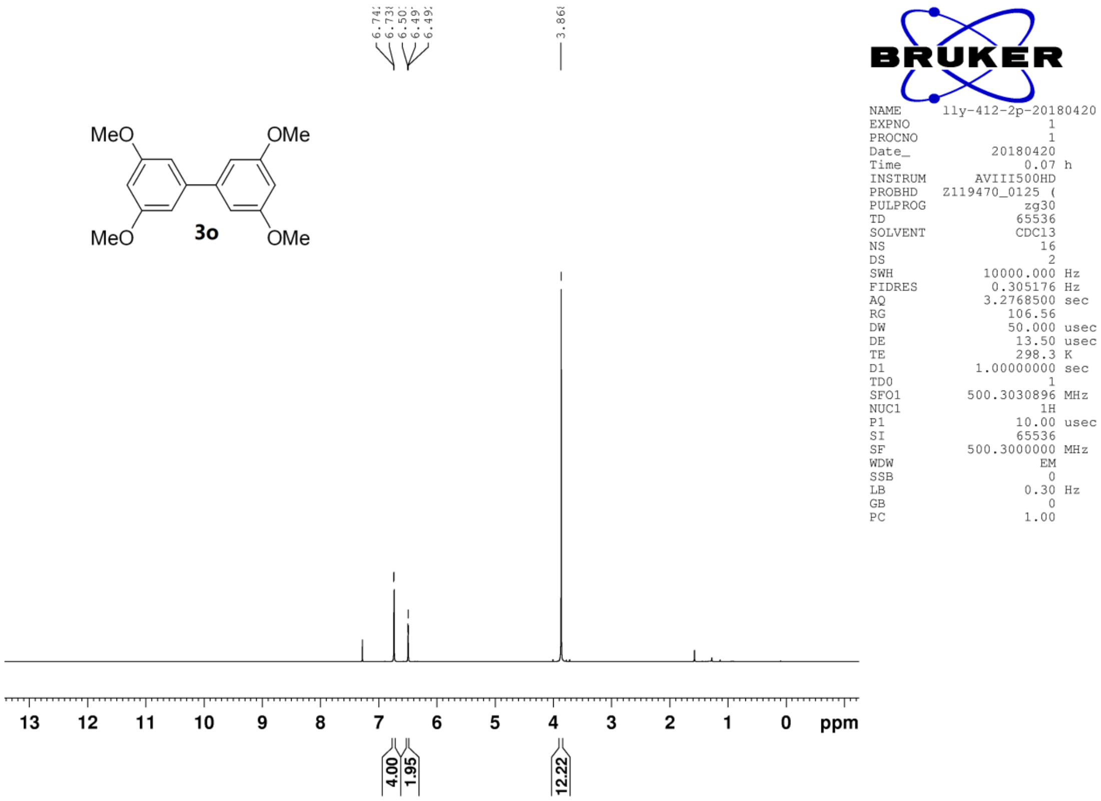

Supplementary Figure 35. ^{1}H NMR spectra for compound **3o**

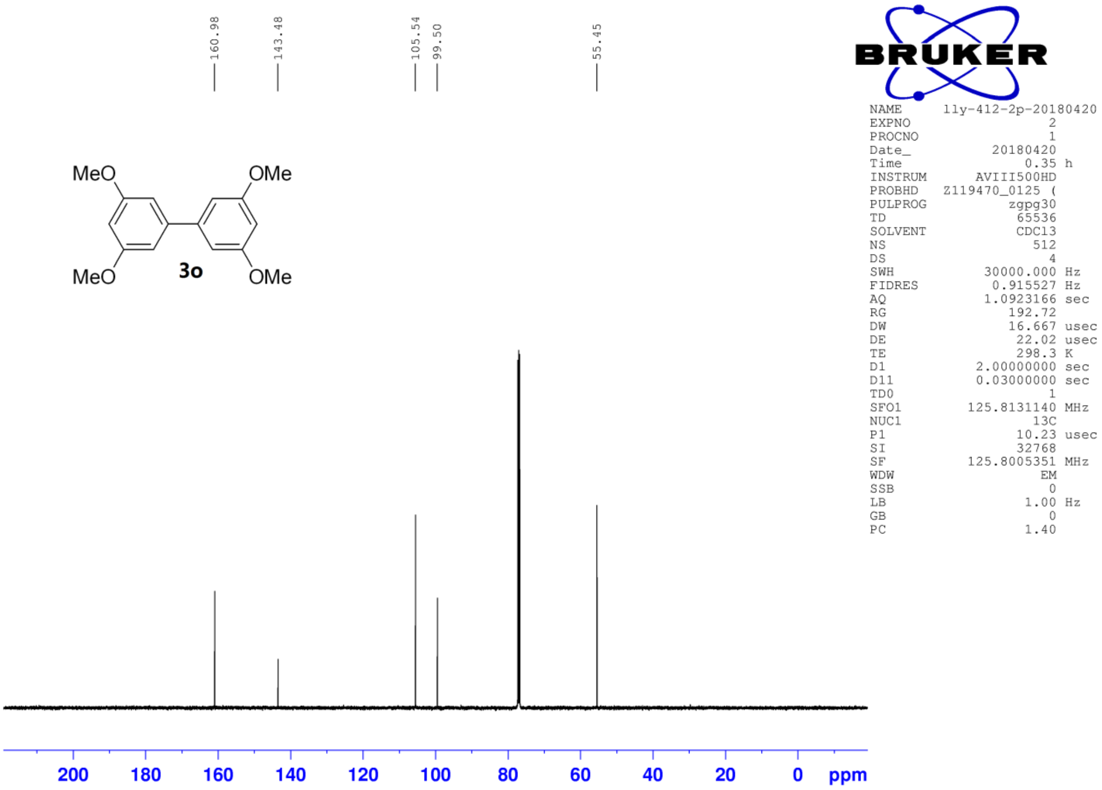

Supplementary Figure 36. ^{13}C NMR spectra for compound **3o**

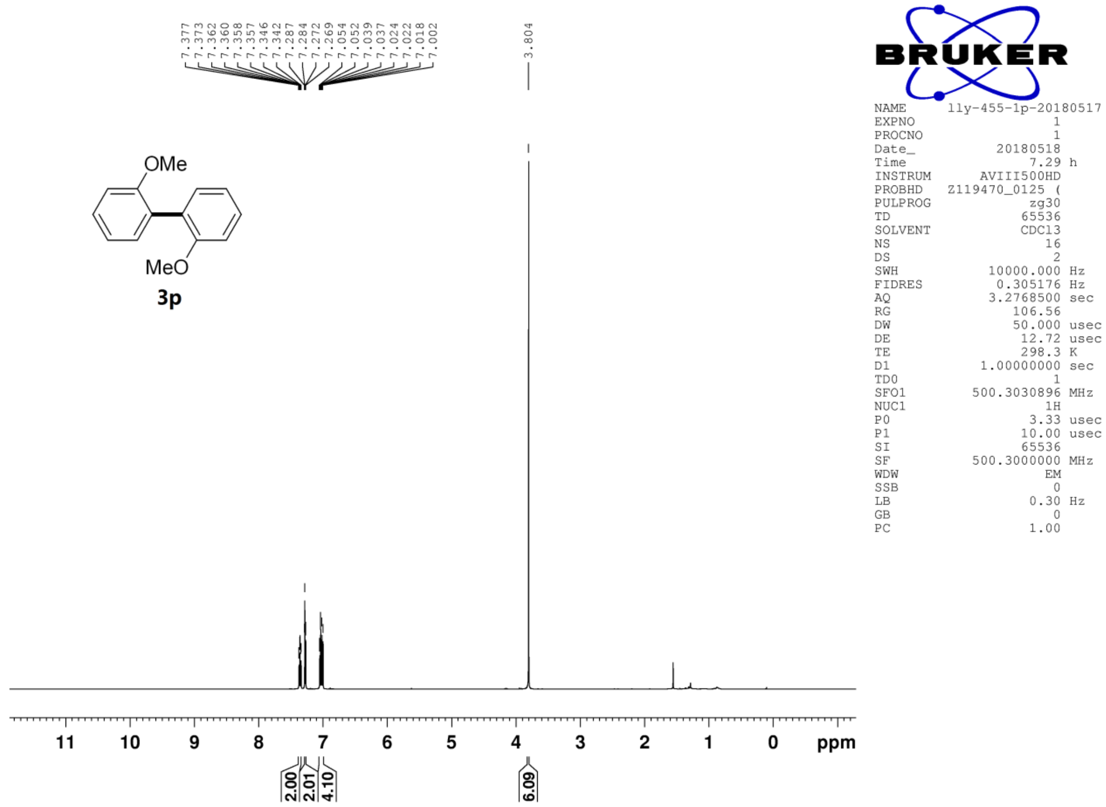

Supplementary Figure 37. ^{1}H NMR spectra for compound **3p**

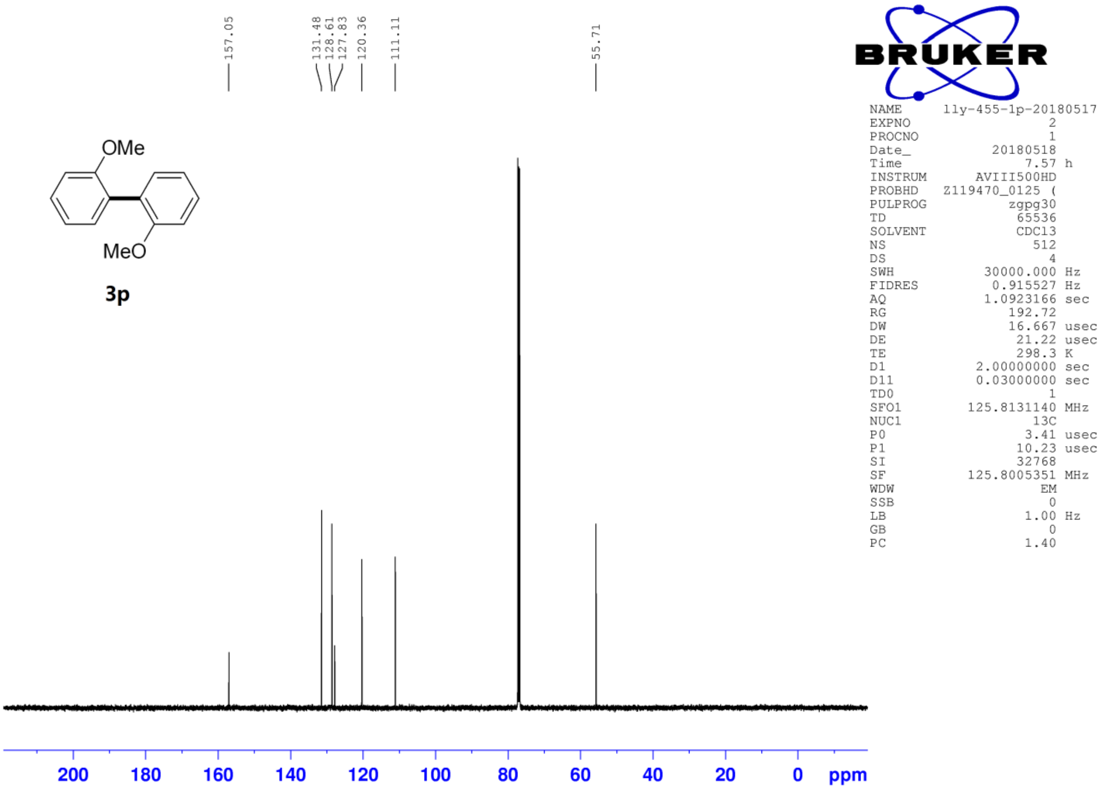

Supplementary Figure 38. ^{13}C NMR spectra for compound **3p**

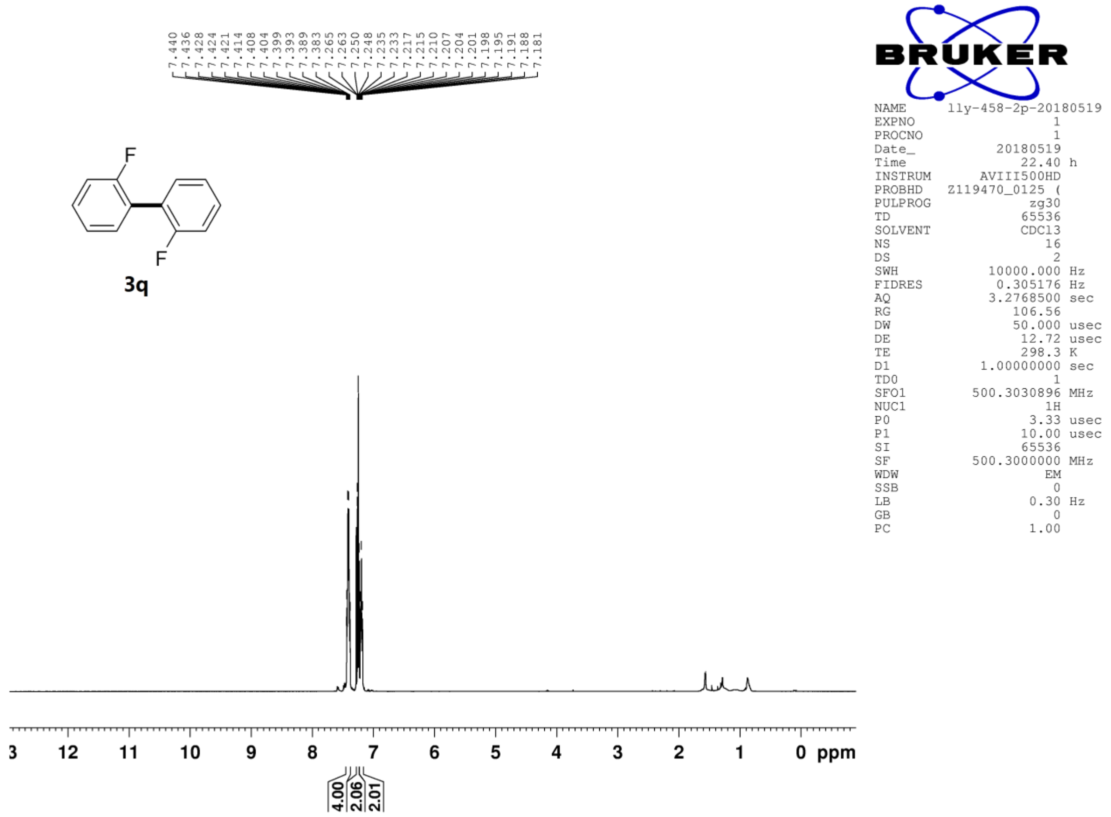

Supplementary Figure 39. ^{1}H NMR spectra for compound **3q**

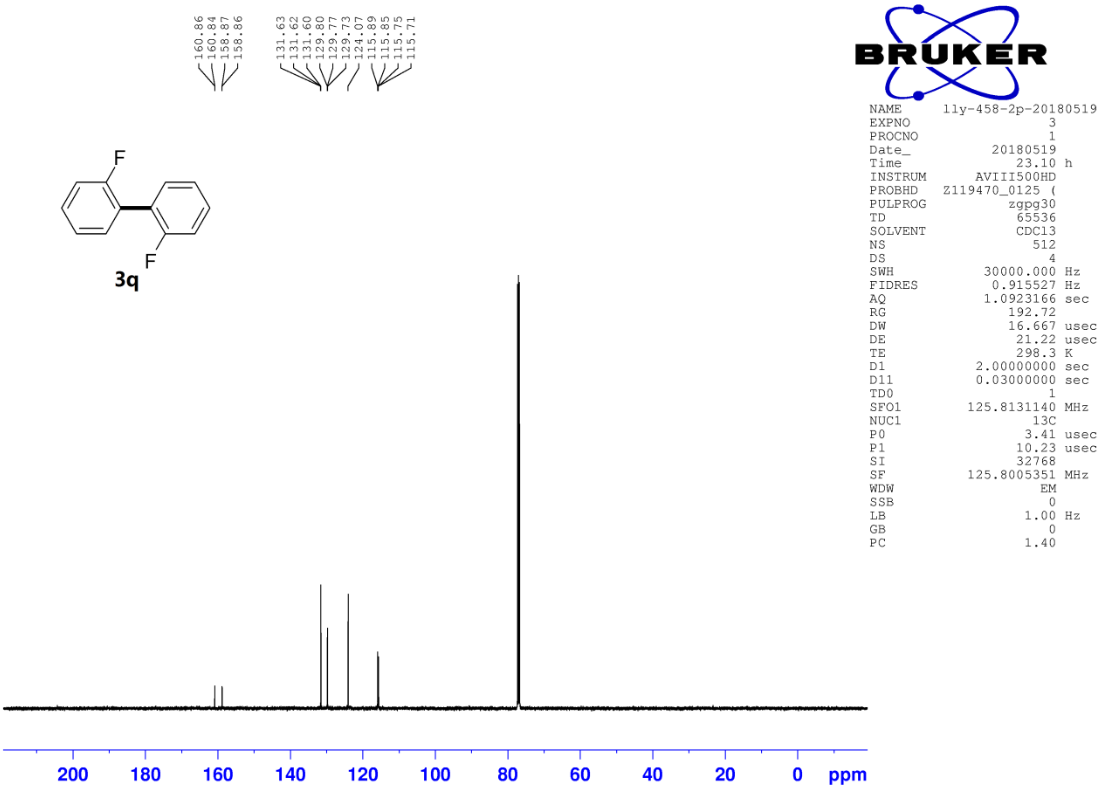

Supplementary Figure 40. ^{13}C NMR spectra for compound **3q**

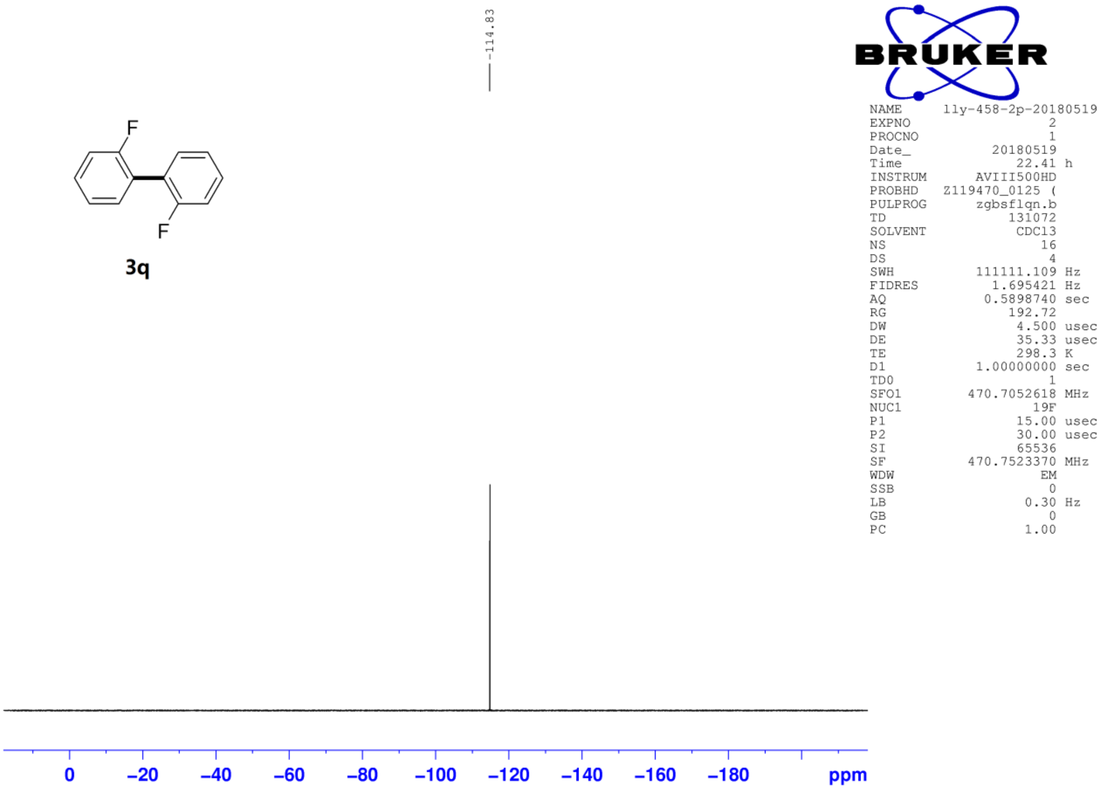

Supplementary Figure 41. ^{19}F NMR spectra for compound **3q**

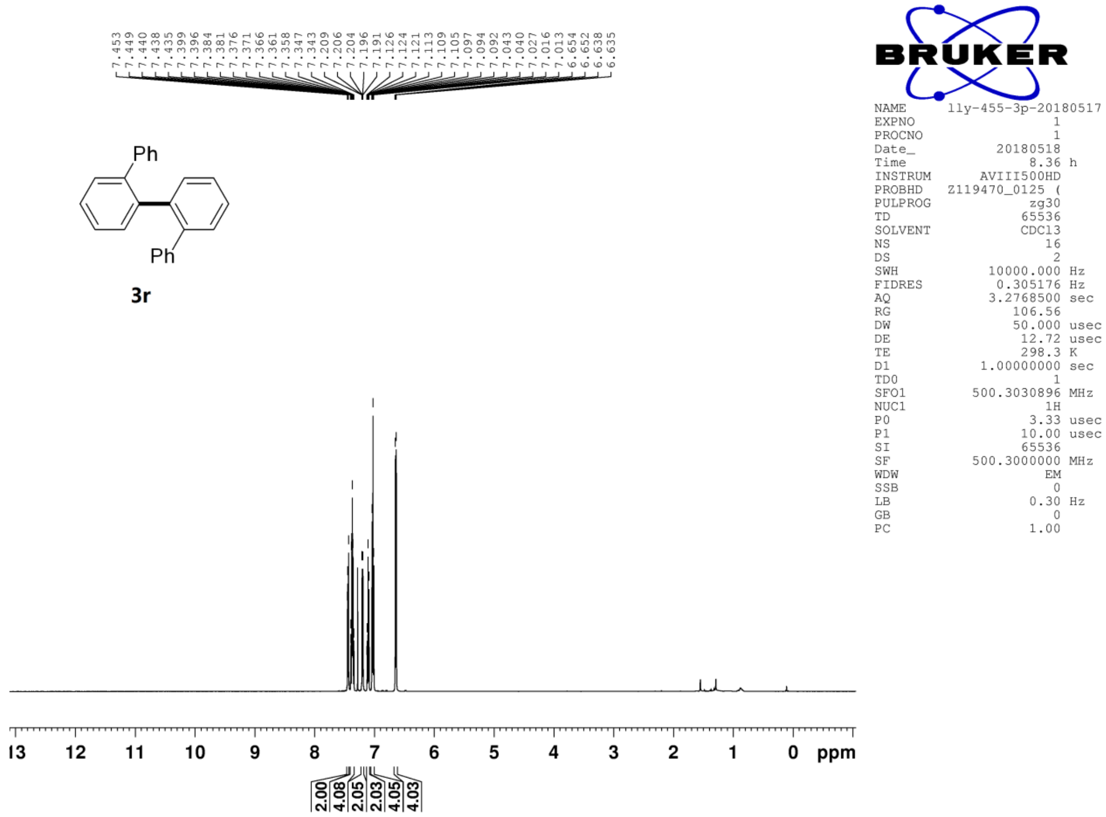

Supplementary Figure 42. ^{1}H NMR spectra for compound **3r**

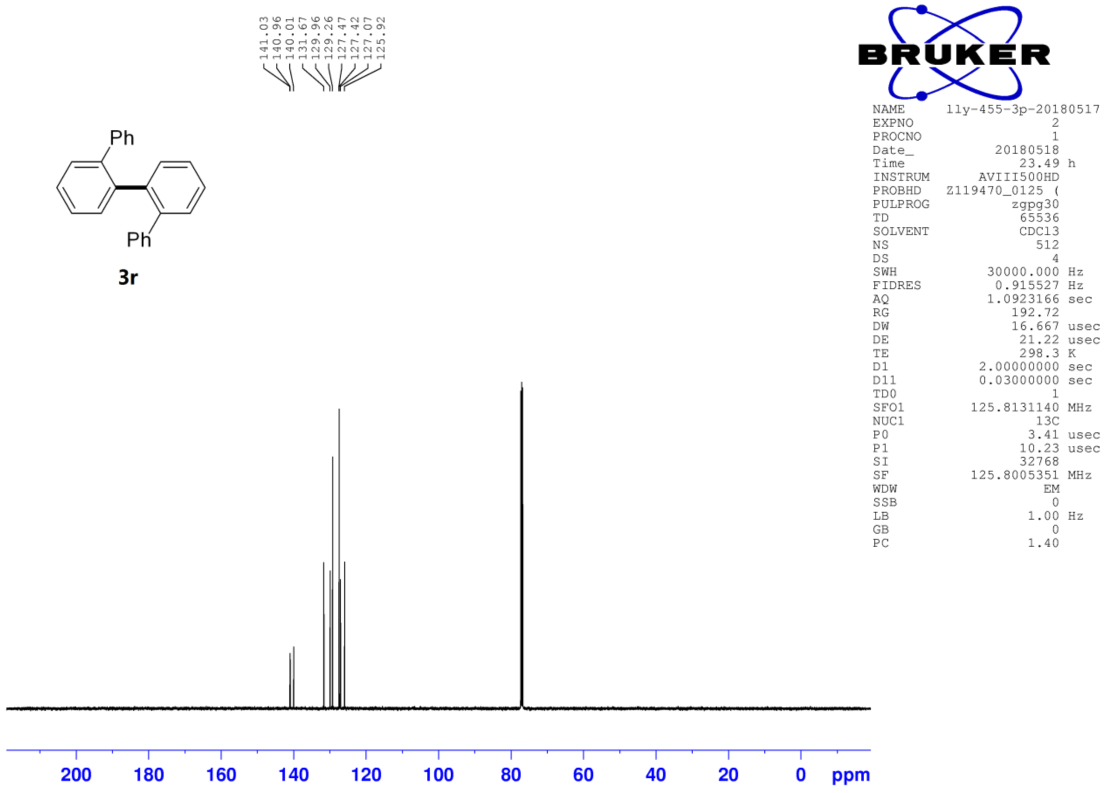

Supplementary Figure 43. ^{13}C NMR spectra for compound **3r**

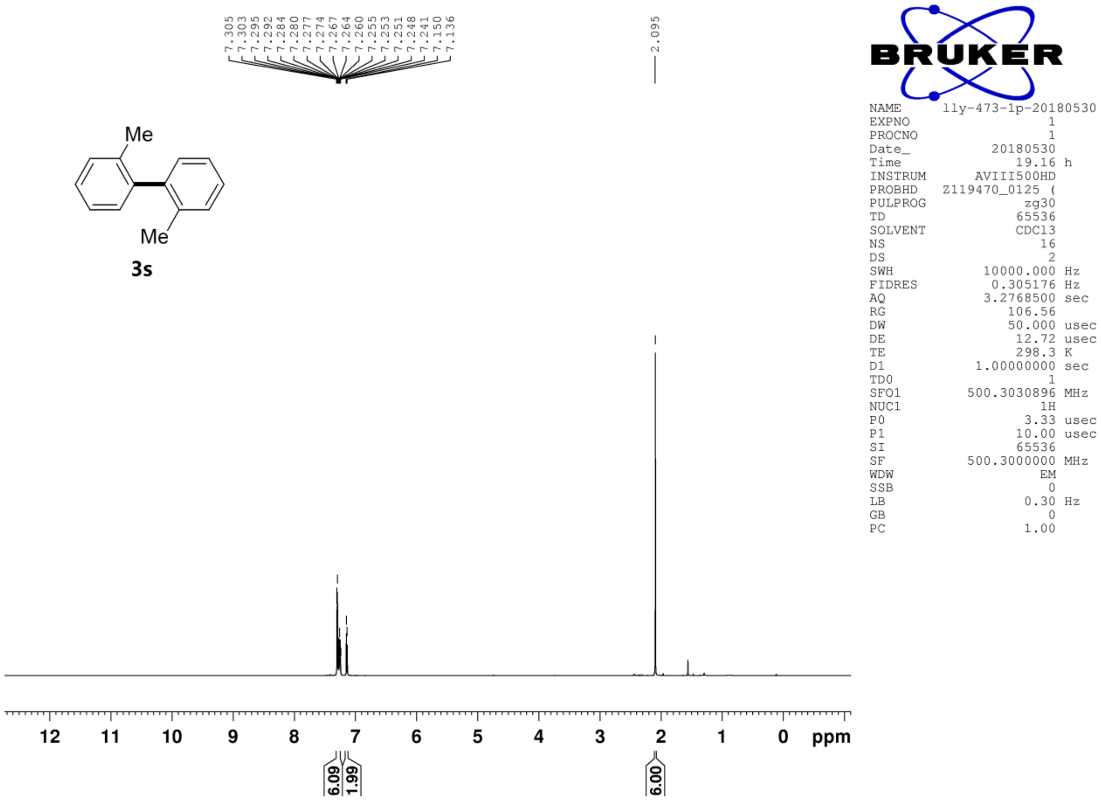

Supplementary Figure 44. ^{1}H NMR spectra for compound **3s**

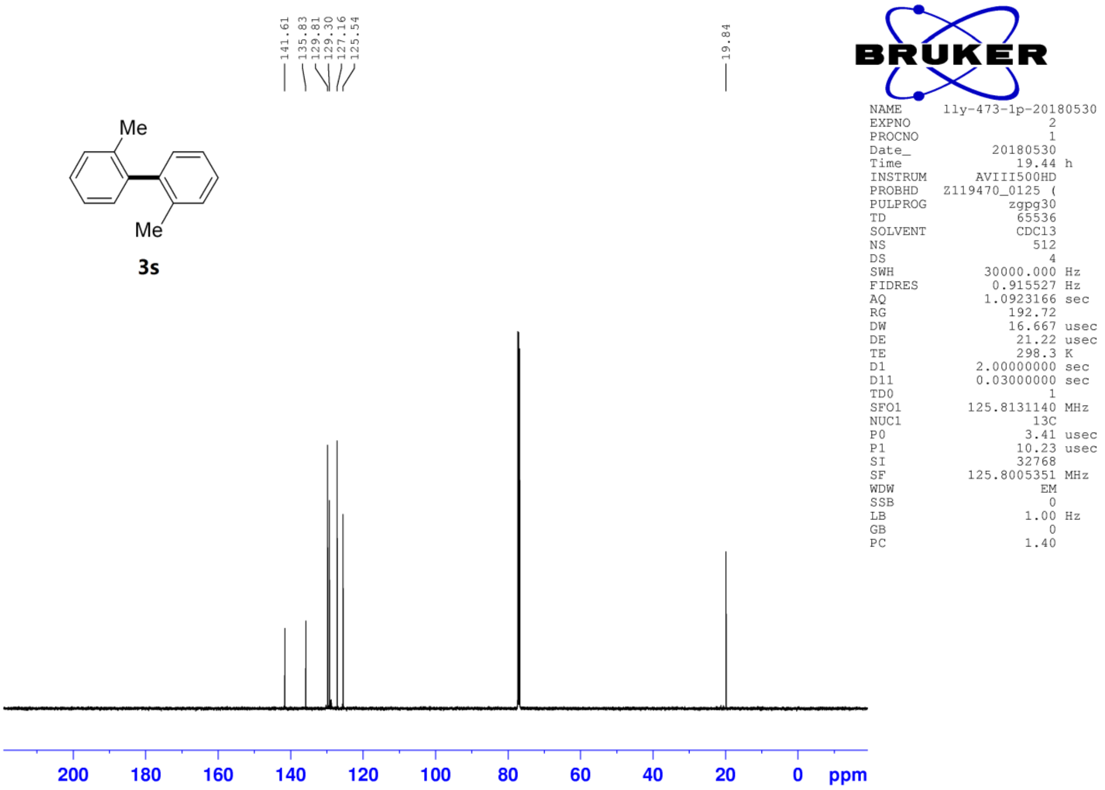

Supplementary Figure 45 . ^{13}C NMR spectra for compound **3s**

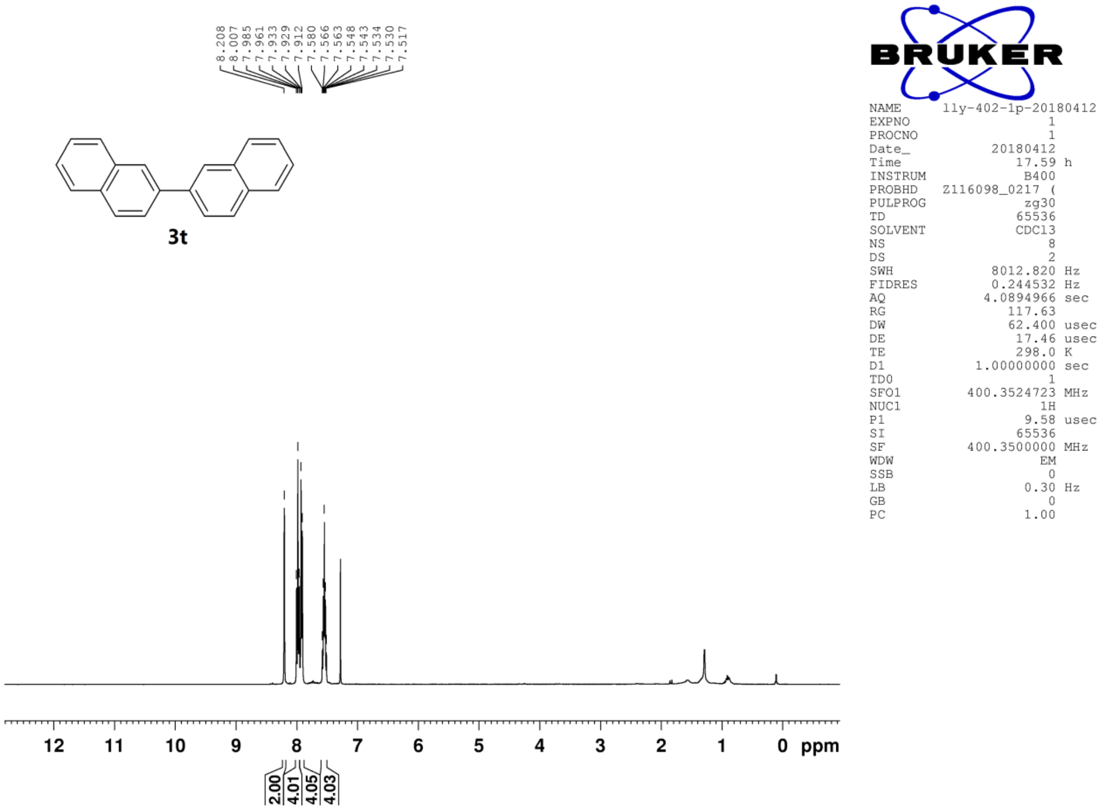

Supplementary Figure 46. ^{1}H NMR spectra for compound **3t**

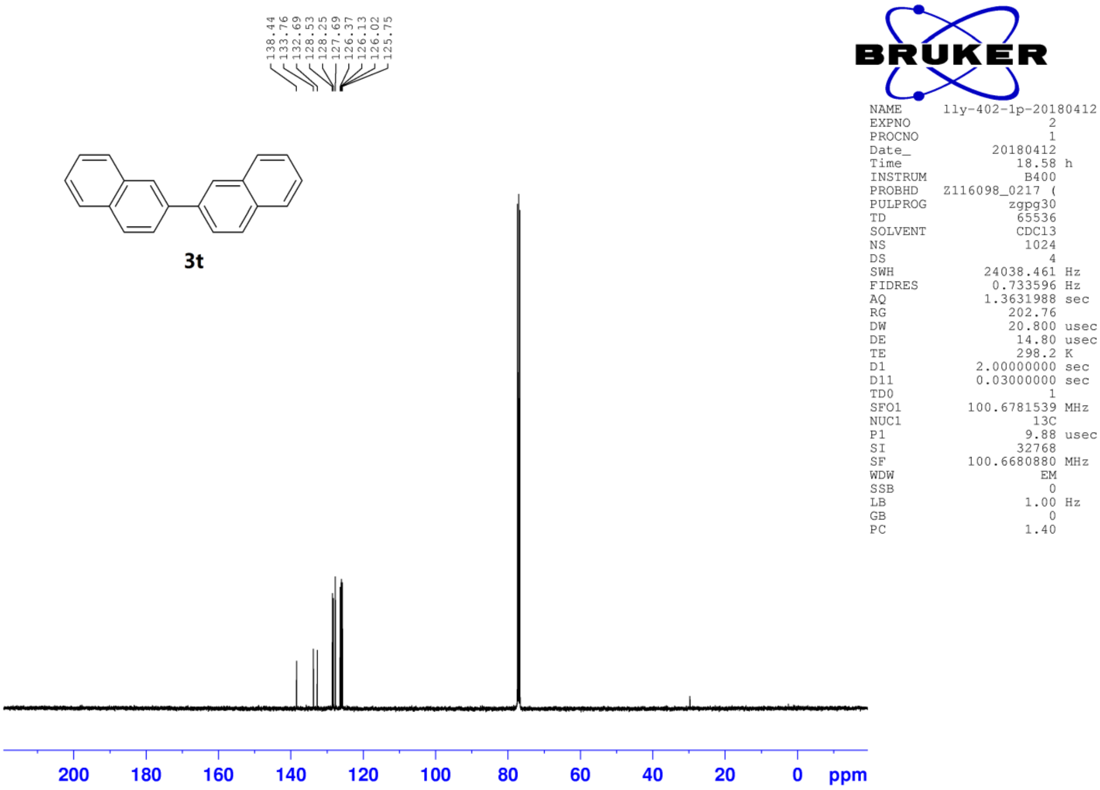

Supplementary Figure 47. ^{13}C NMR spectra for compound **3t**

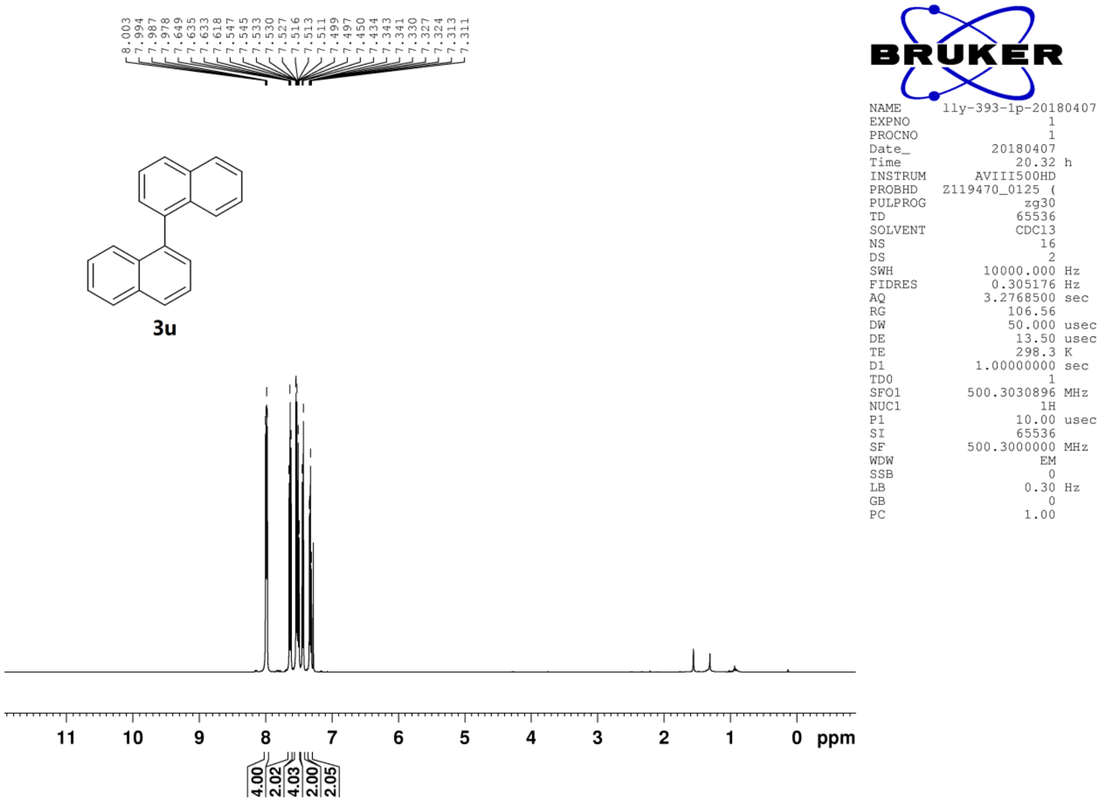

Supplementary Figure 48. ^{1}H NMR spectra for compound **3u**

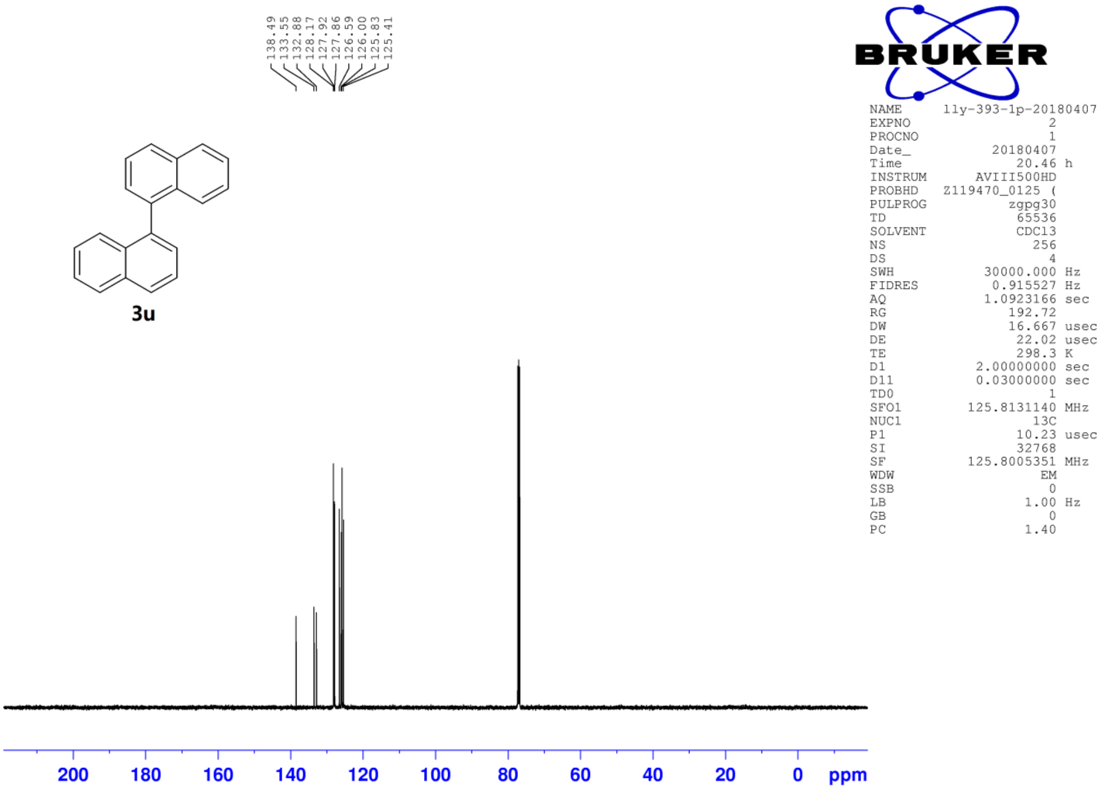

Supplementary Figure 49. ^{13}C NMR spectra for compound **3u**

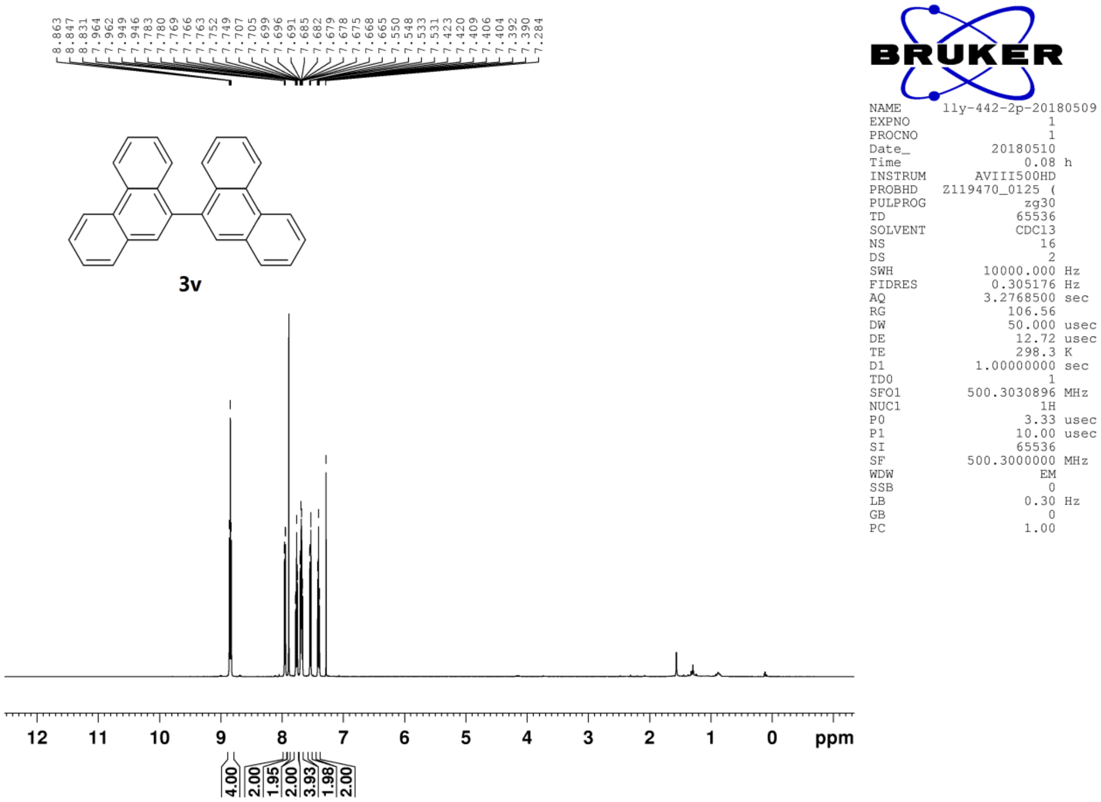

Supplementary Figure 50. ^{1}H NMR spectra for compound **3v**

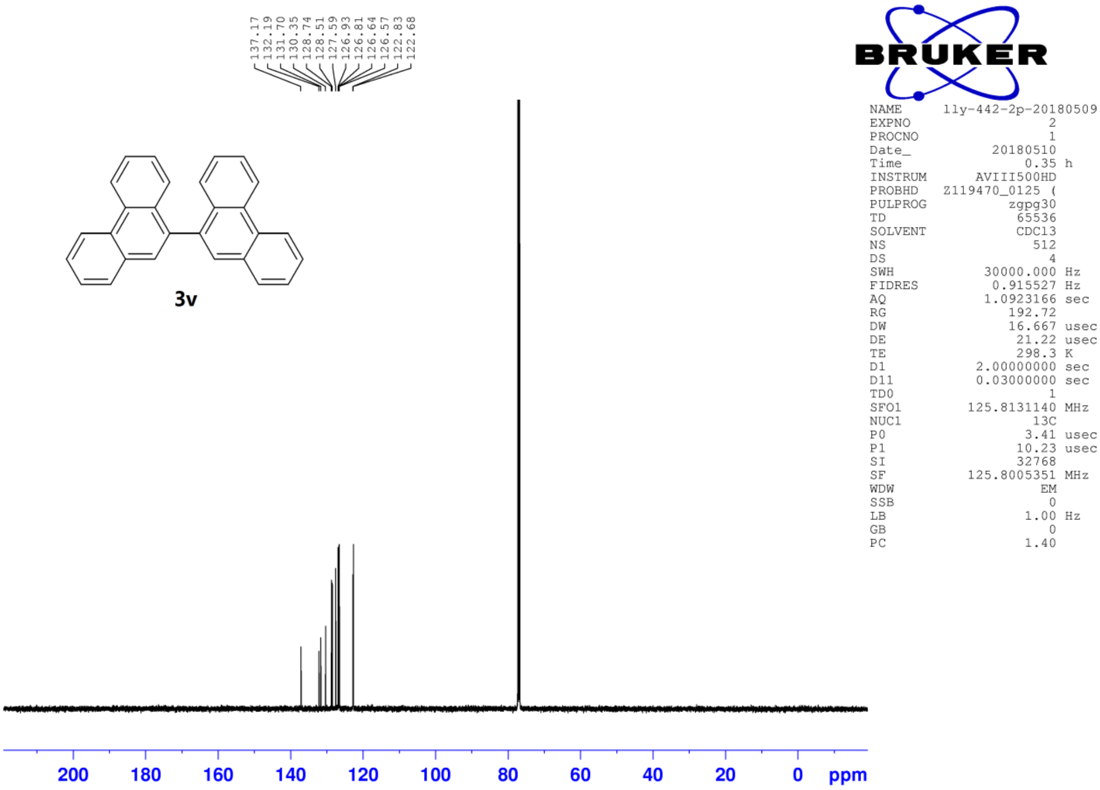

Supplementary Figure 51. ^{13}C NMR spectra for compound **3v**

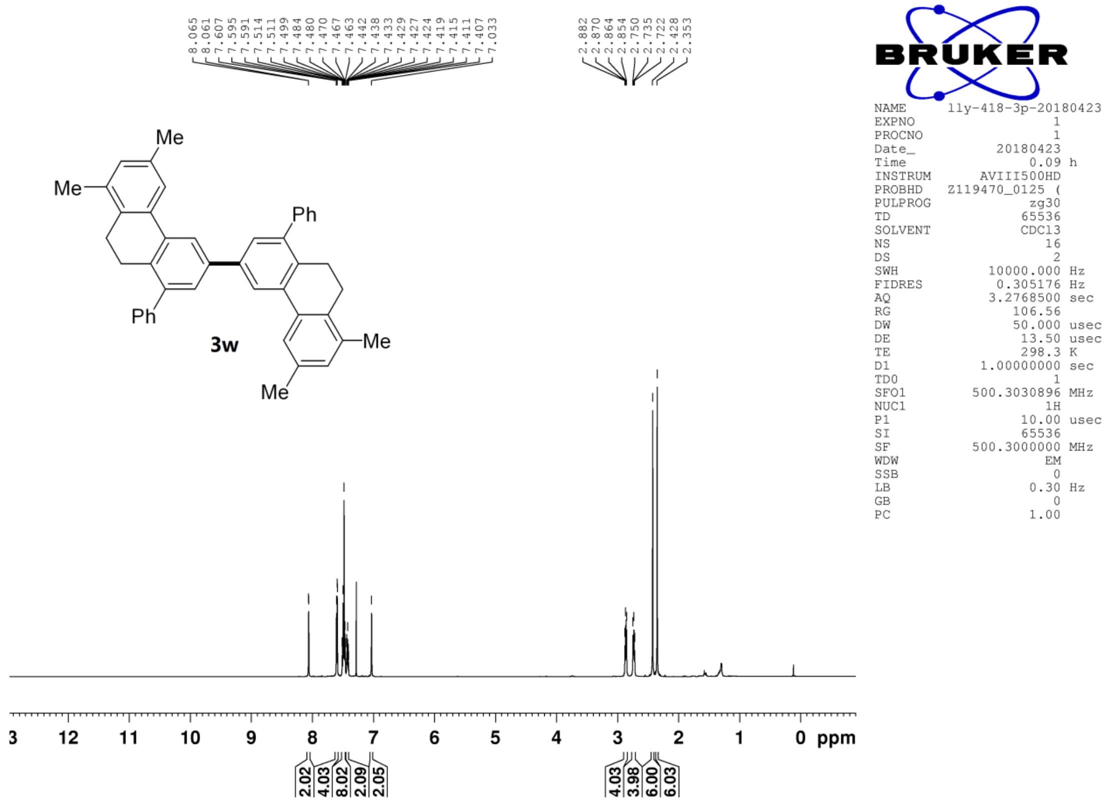

Supplementary Figure 52. ^{1}H NMR spectra for compound **3w**

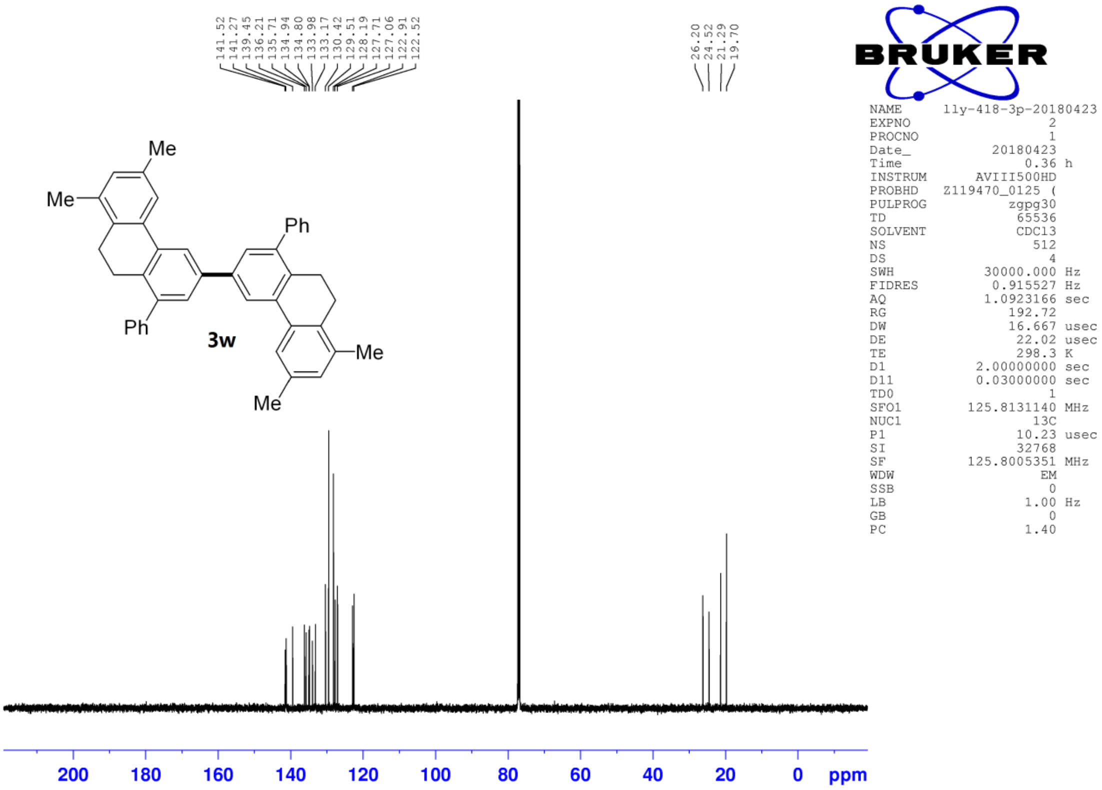

Supplementary Figure 53. ^{13}C NMR spectra for compound **3w**

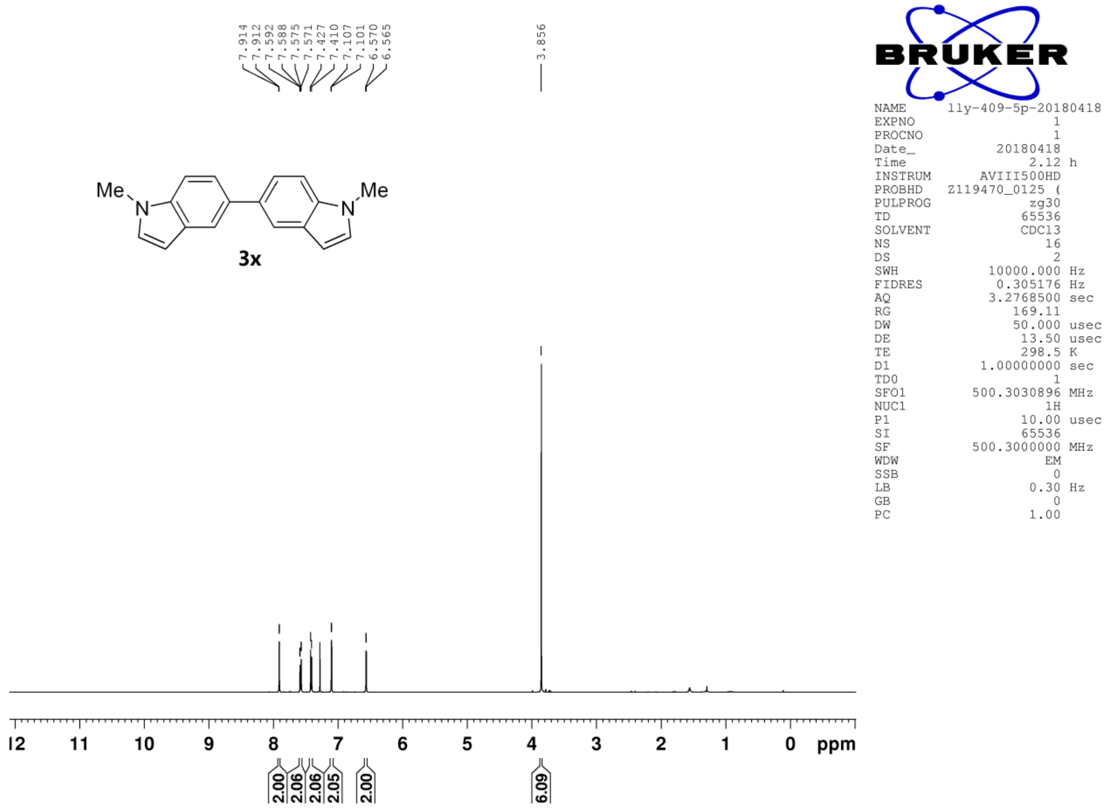

Supplementary Figure 54. ^{1}H NMR spectra for compound **3x**

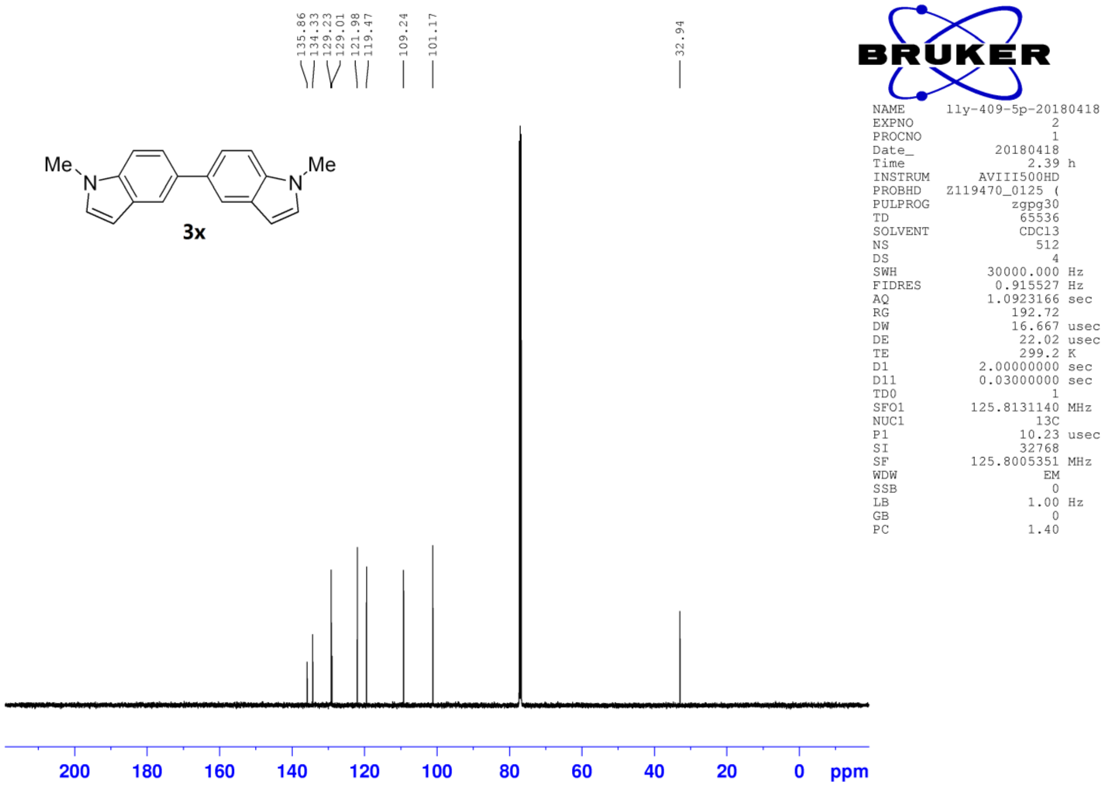

Supplementary Figure 55. ^{13}C NMR spectra for compound **3x**

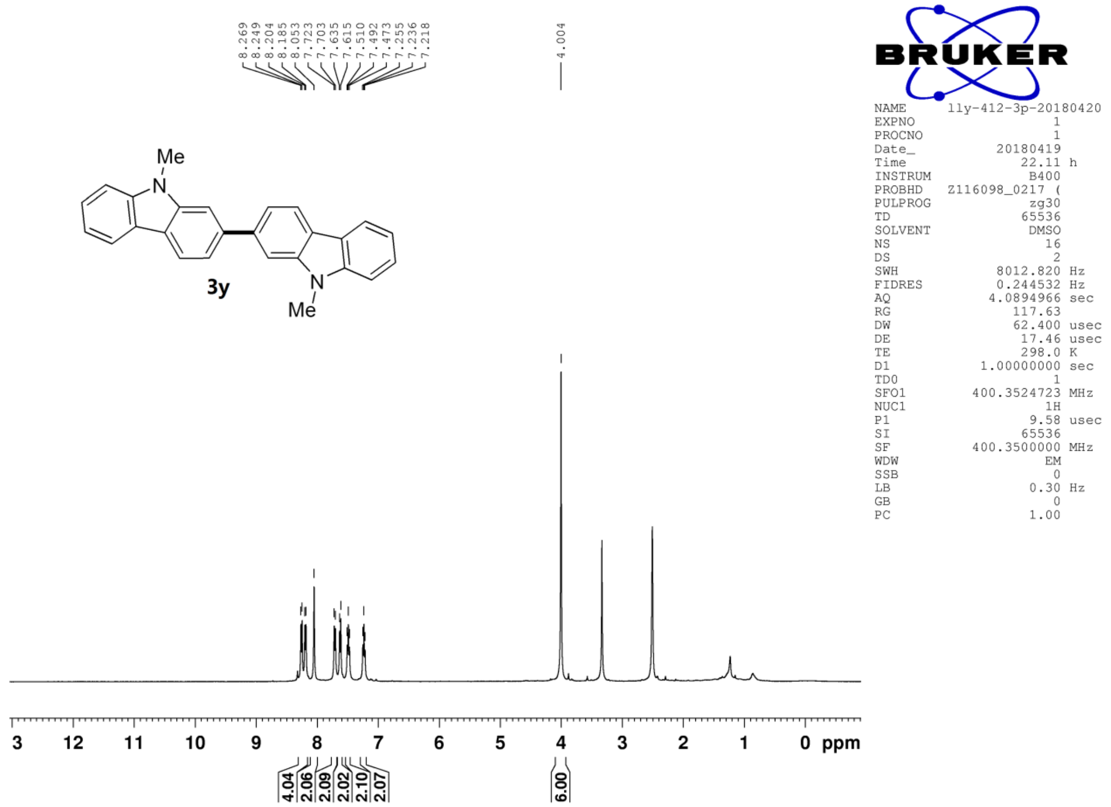

Supplementary Figure 56. ^{1}H NMR spectra for compound **3y**

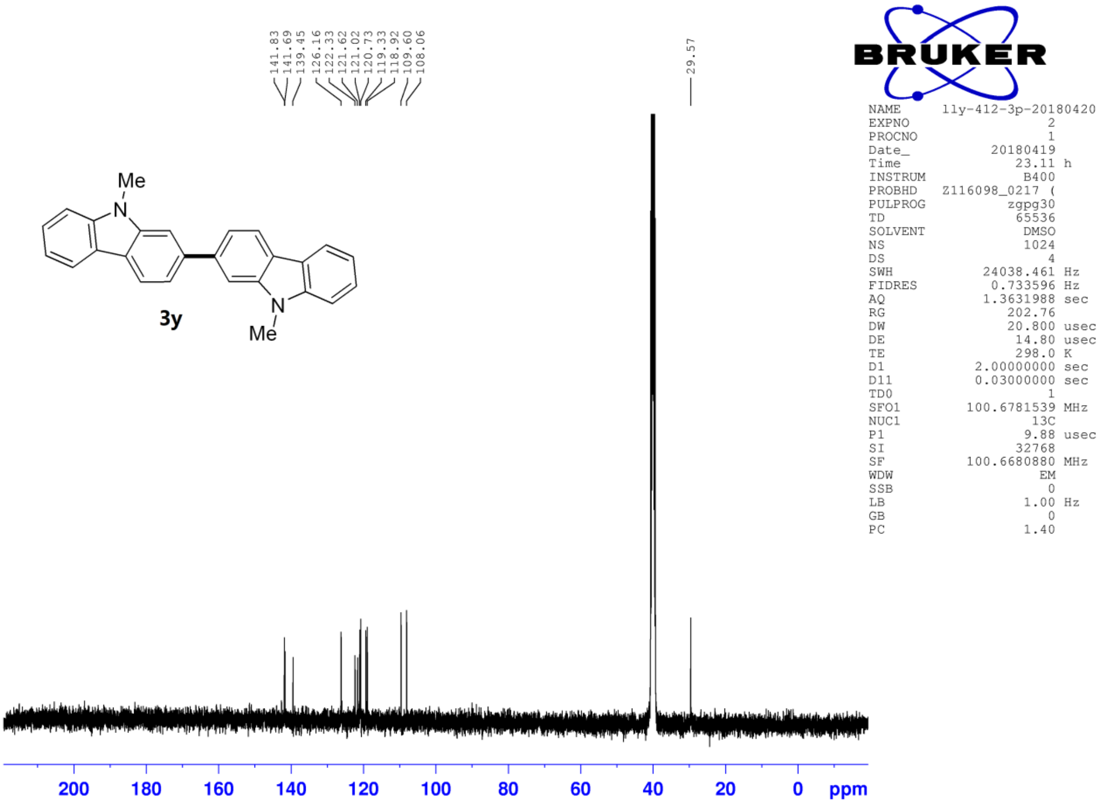

Supplementary Figure 57. ^{13}C NMR spectra for compound **3y**

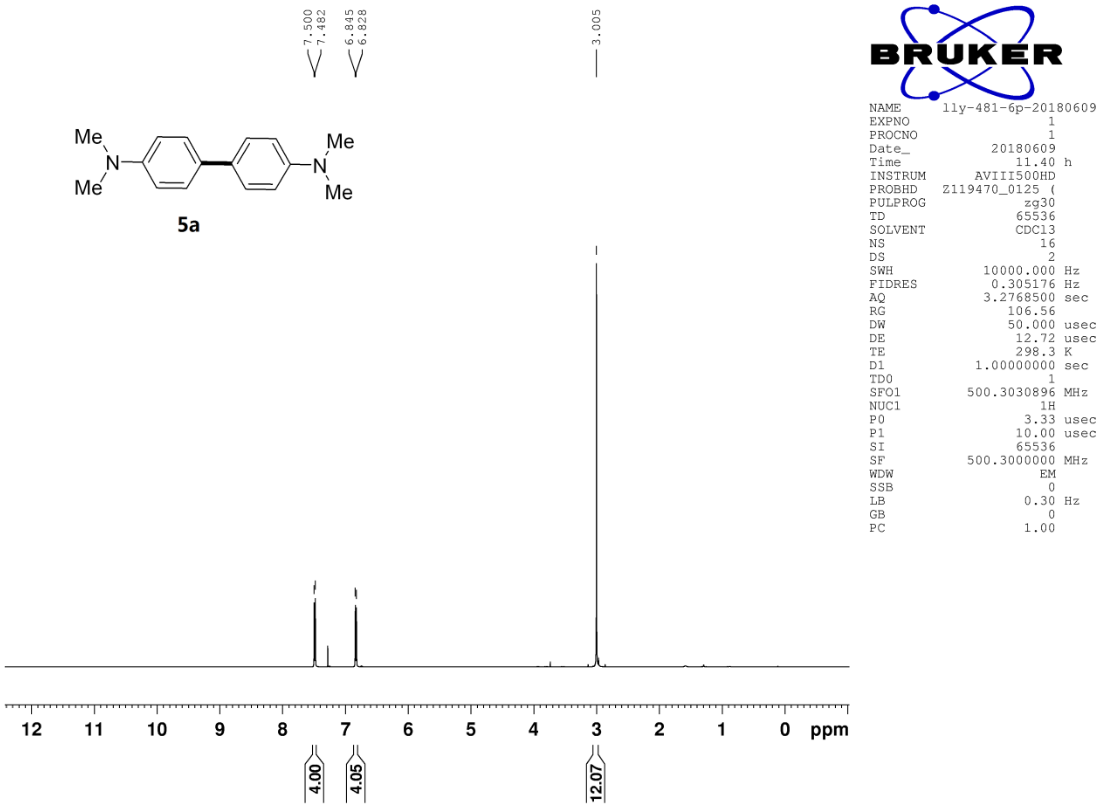

Supplementary Figure 58. ^{1}H NMR spectra for compound **5a**

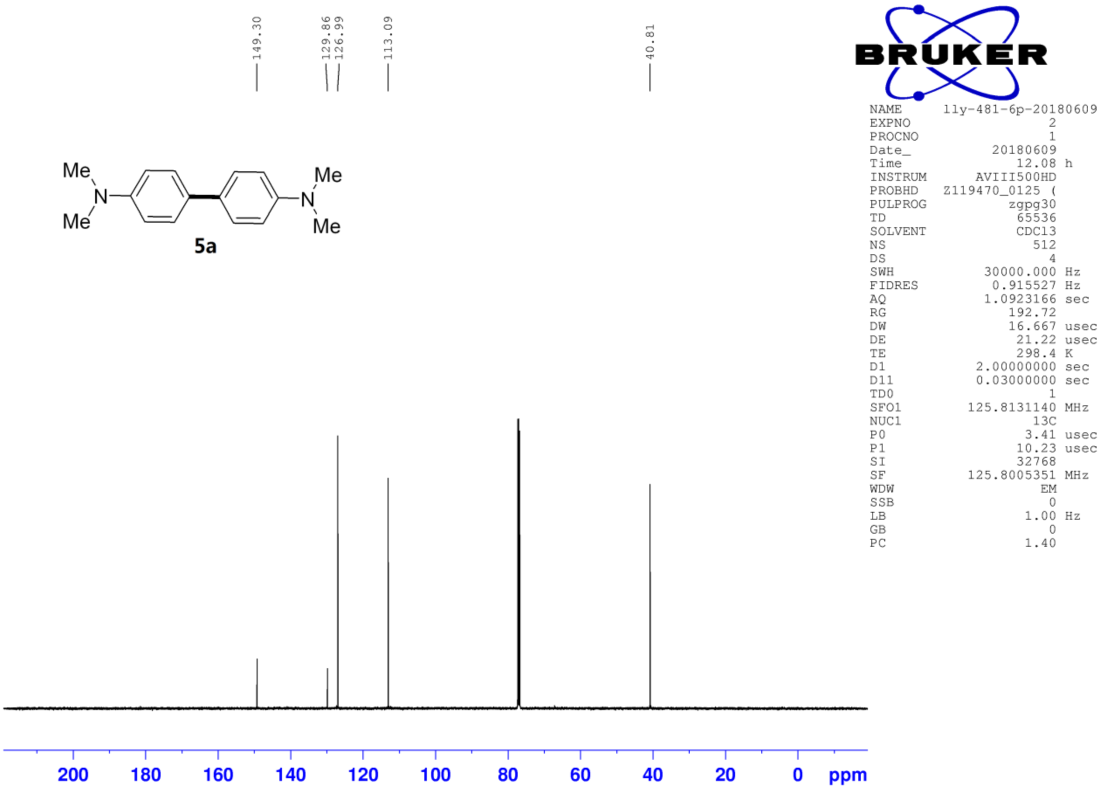

Supplementary Figure 59. ^{13}C NMR spectra for compound **5a**

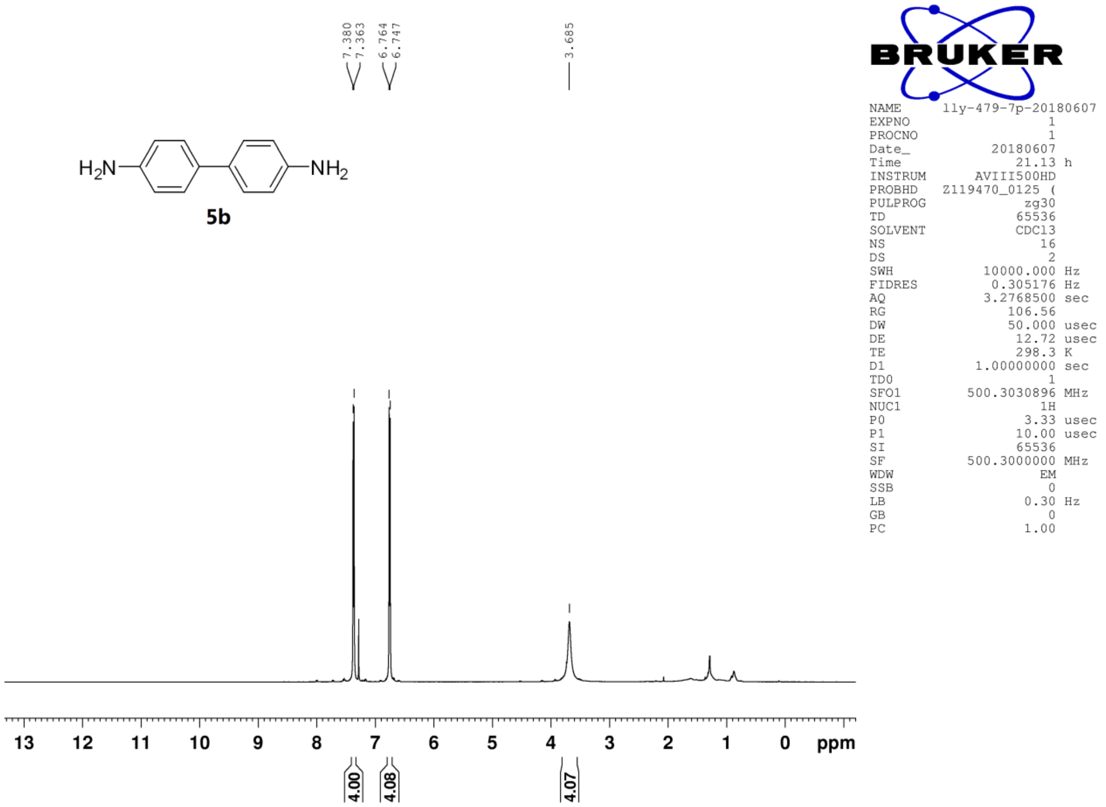

Supplementary Figure 60. ^{1}H NMR spectra for compound **5b**

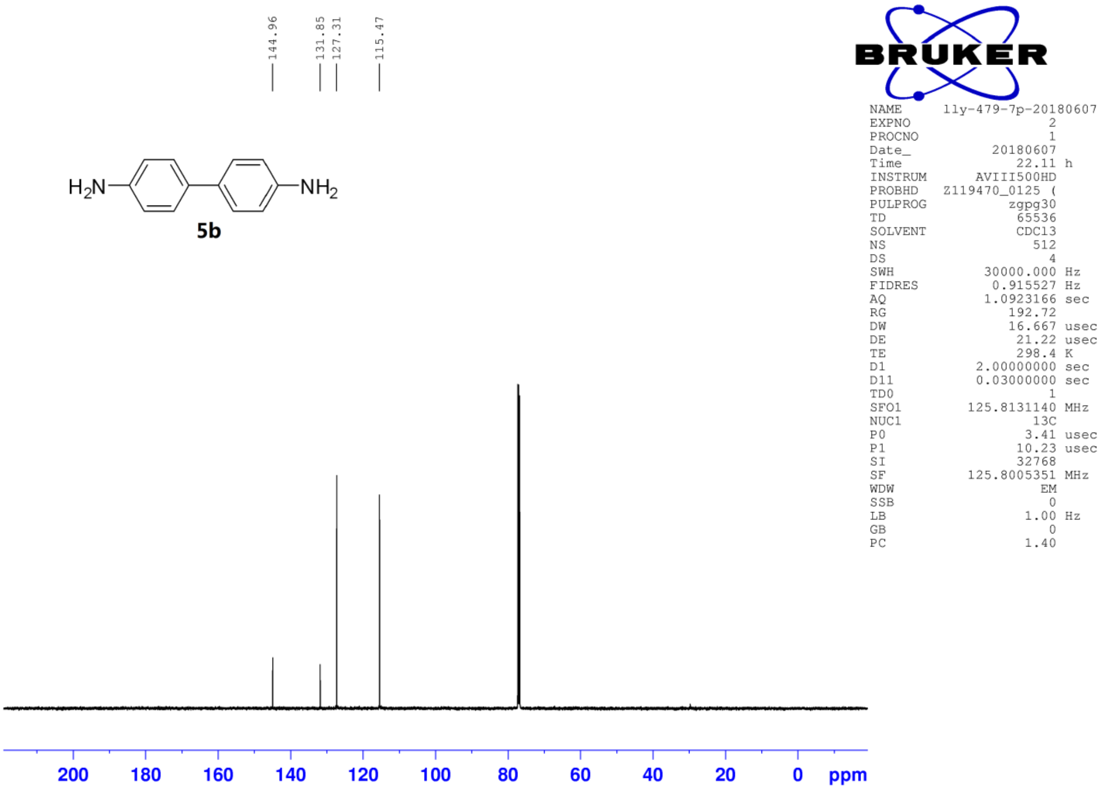

Supplementary Figure 61. ^{13}C NMR spectra for compound **5b**

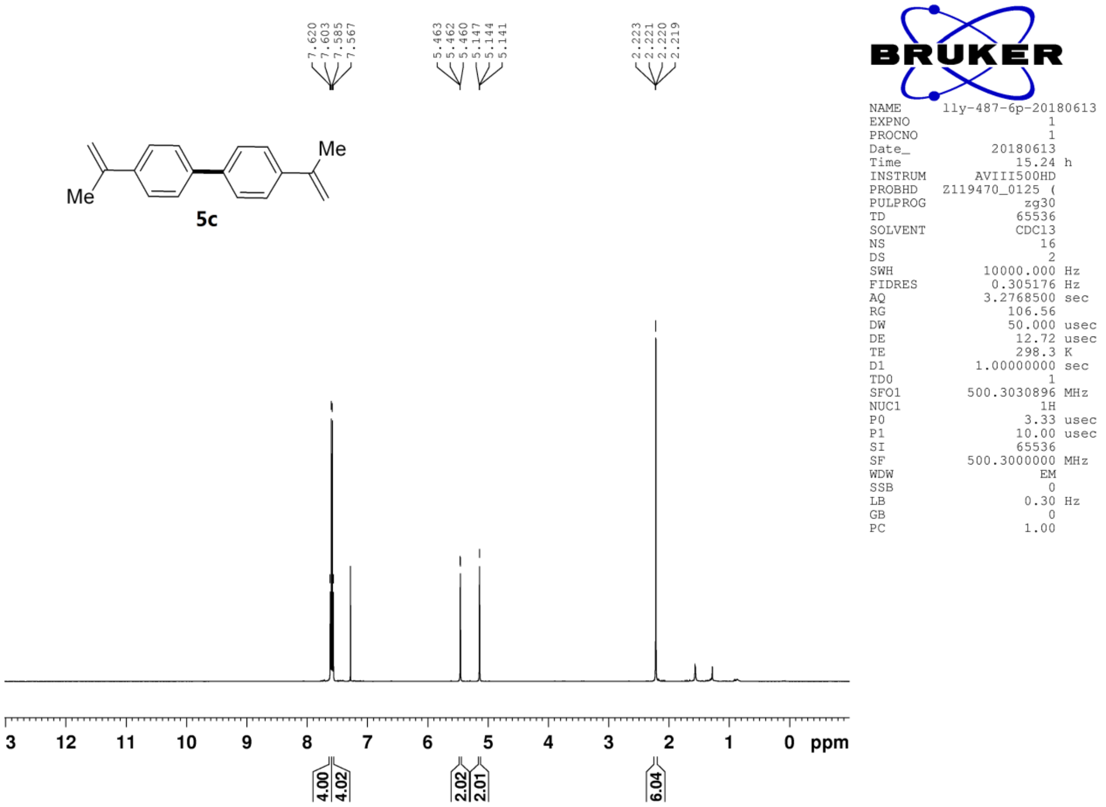

Supplementary Figure 62. ^{1}H NMR spectra for compound **5c**

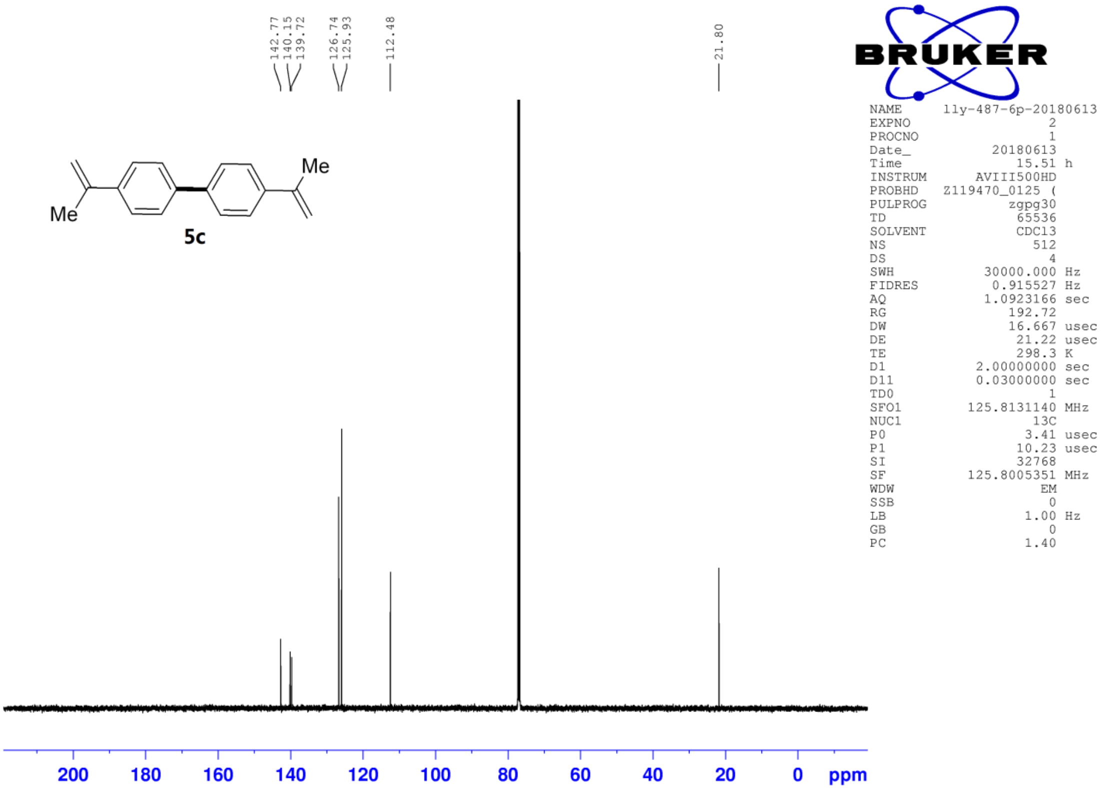

Supplementary Figure 63. ^{13}C NMR spectra for compound **5c**

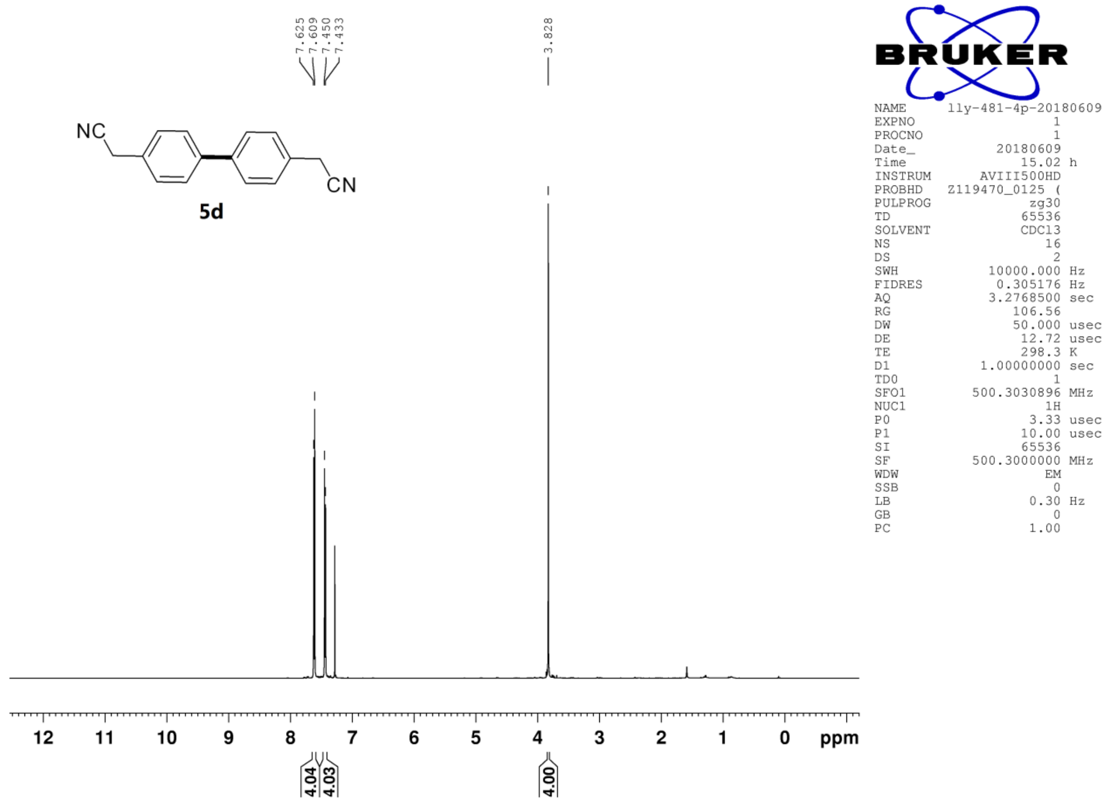

Supplementary Figure 64. ^{1}H NMR spectra for compound **5d**

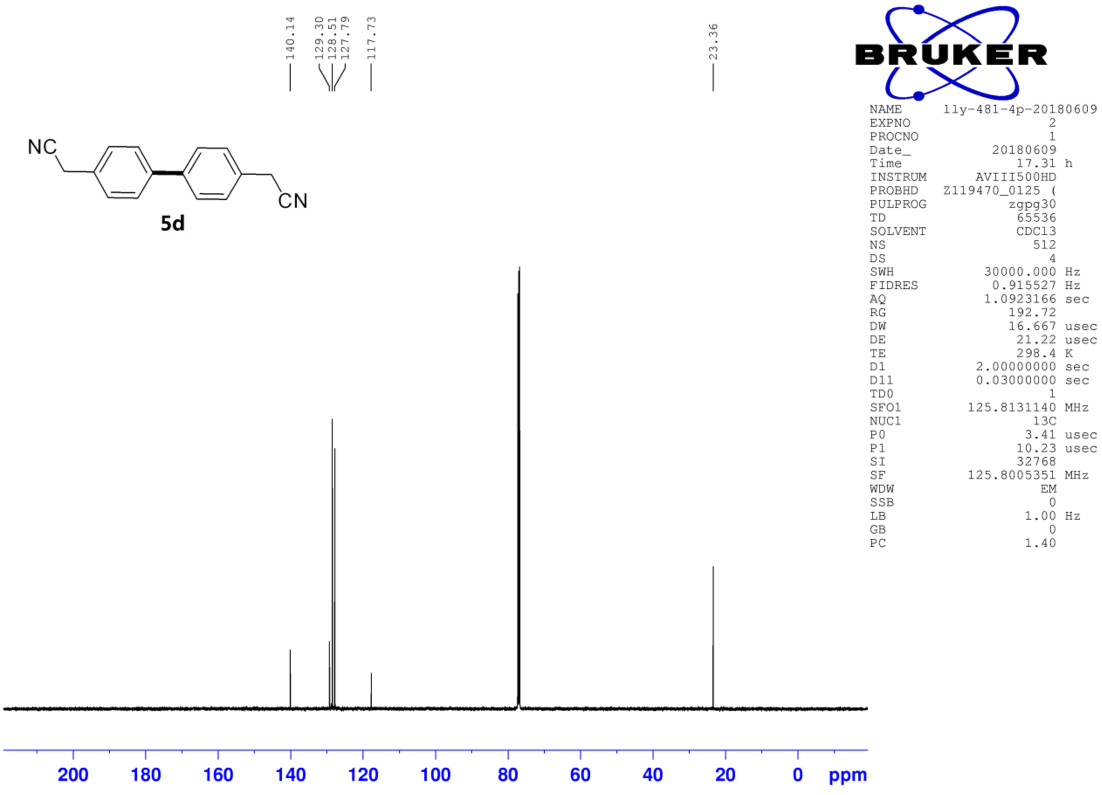

Supplementary Figure 65. ^{13}C NMR spectra for compound **5d**

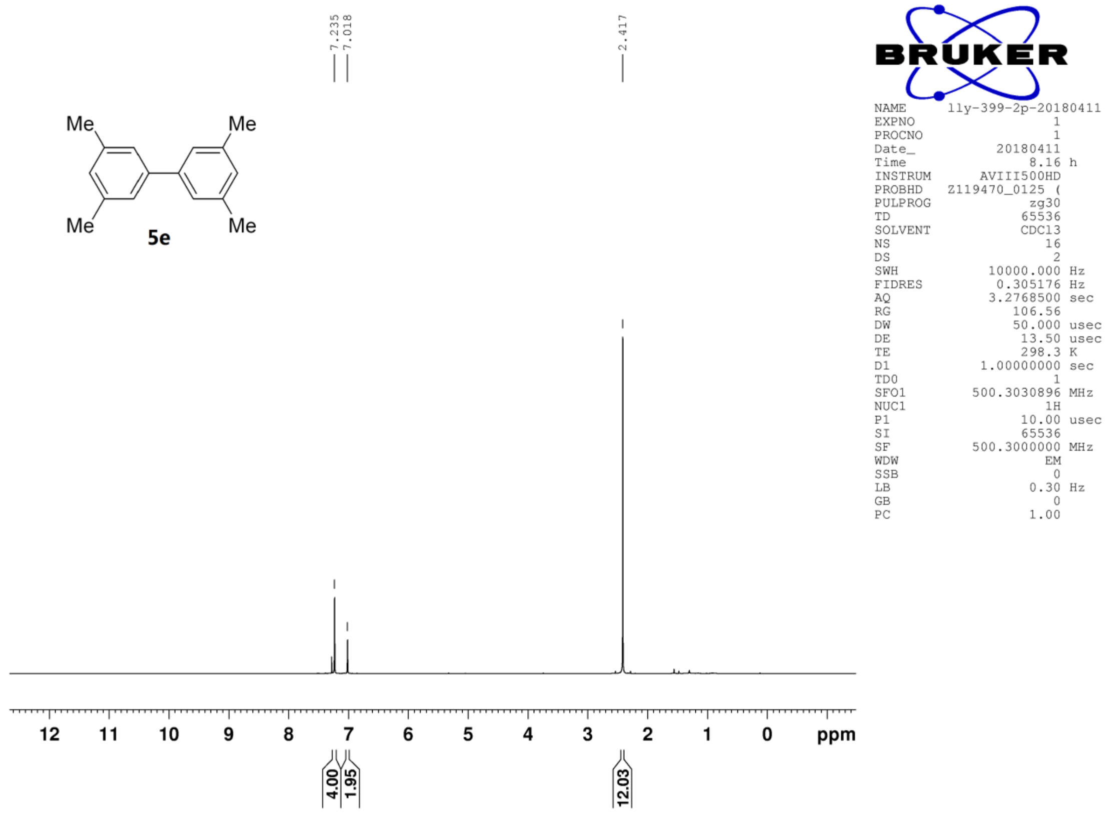

Supplementary Figure 66. ^{1}H NMR spectra for compound **5e**

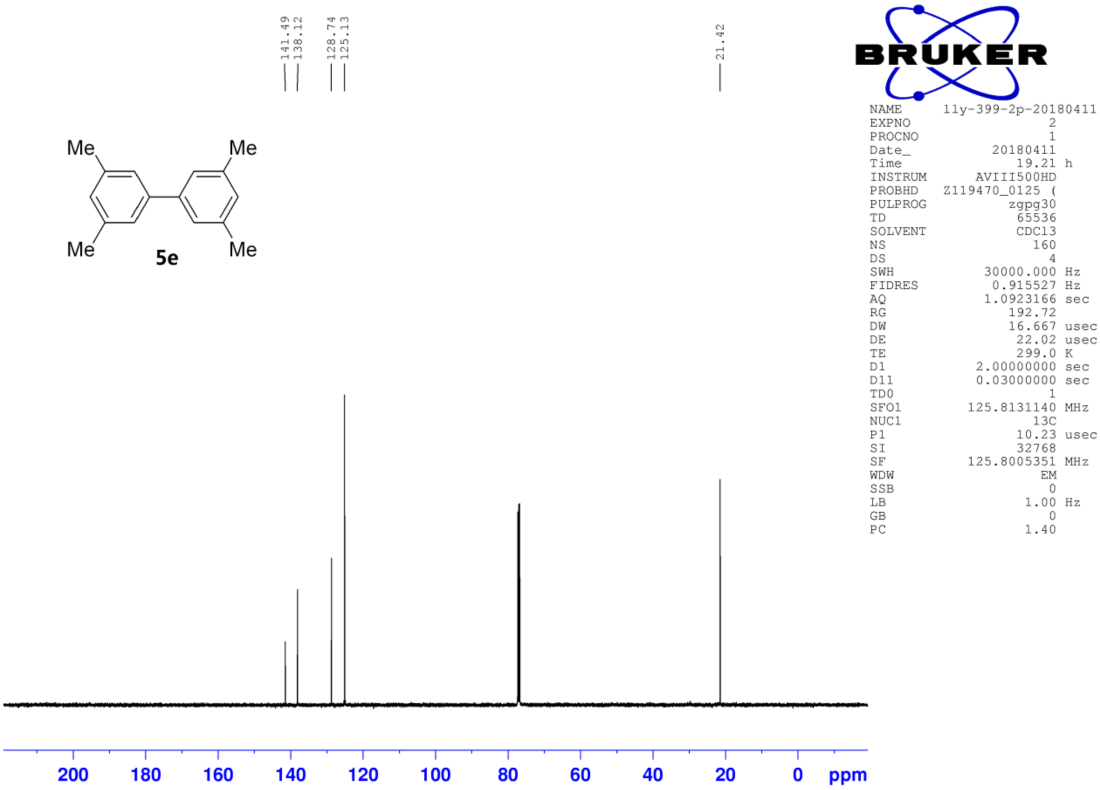

Supplementary Figure 67. ^{13}C NMR spectra for compound **5e**

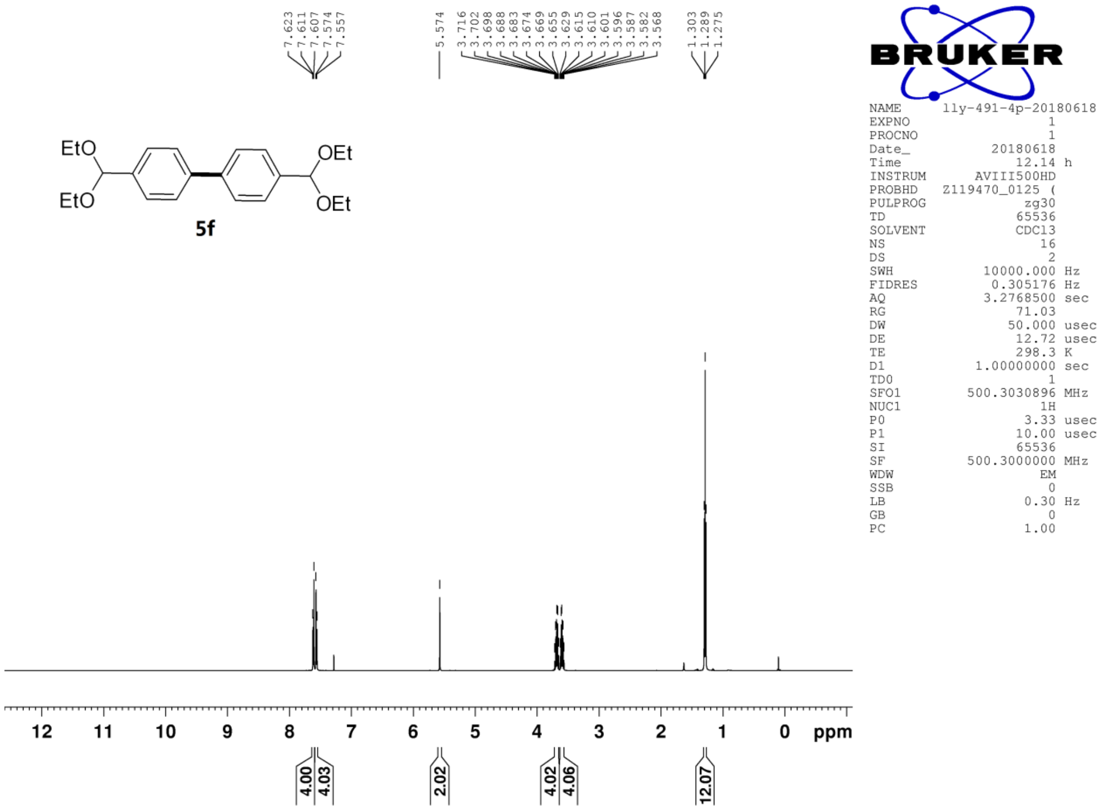

Supplementary Figure 68. ^{1}H NMR spectra for compound **5f**

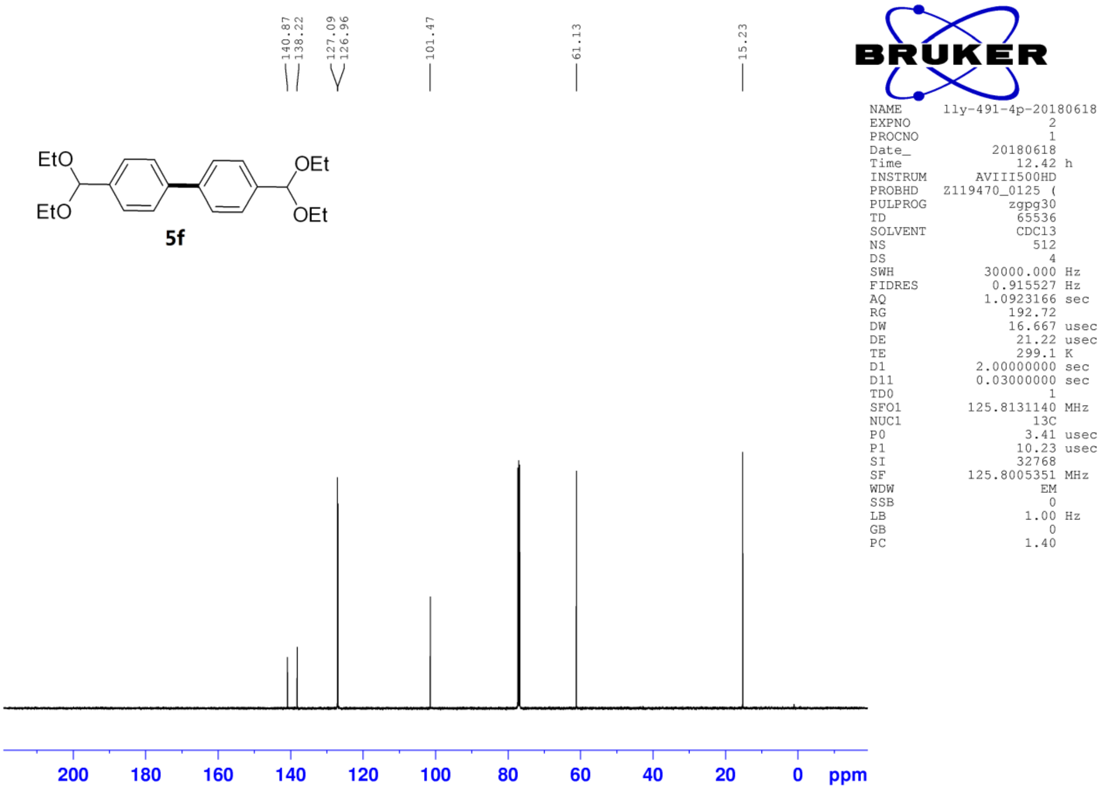

Supplementary Figure 69. ^{13}C NMR spectra for compound **5f**

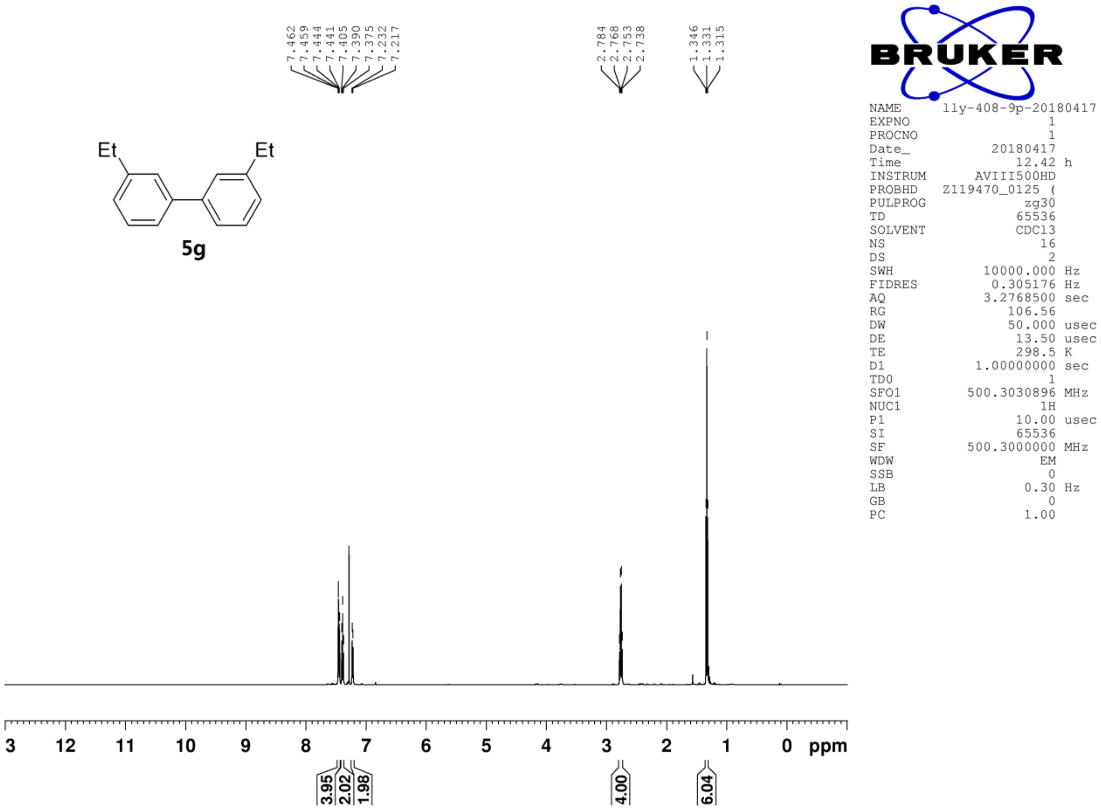

Supplementary Figure 70. ^{1}H NMR spectra for compound **5g**

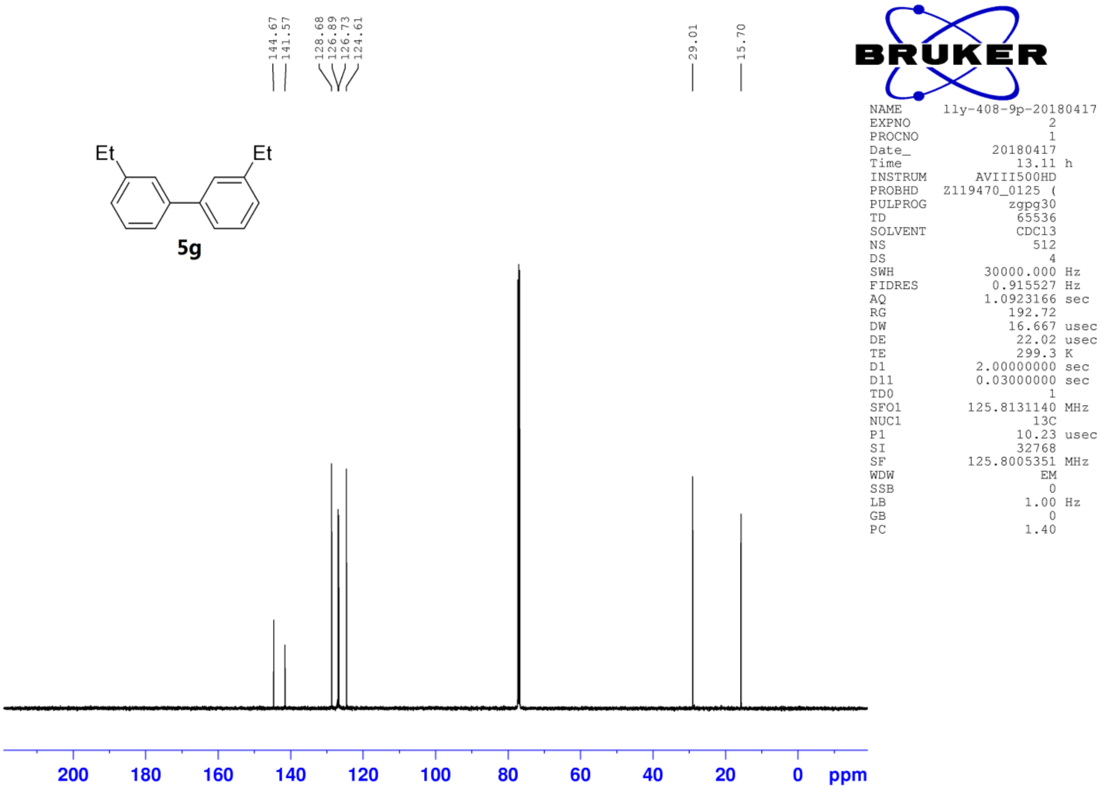

Supplementary Figure 71. ^{13}C NMR spectra for compound **5g**

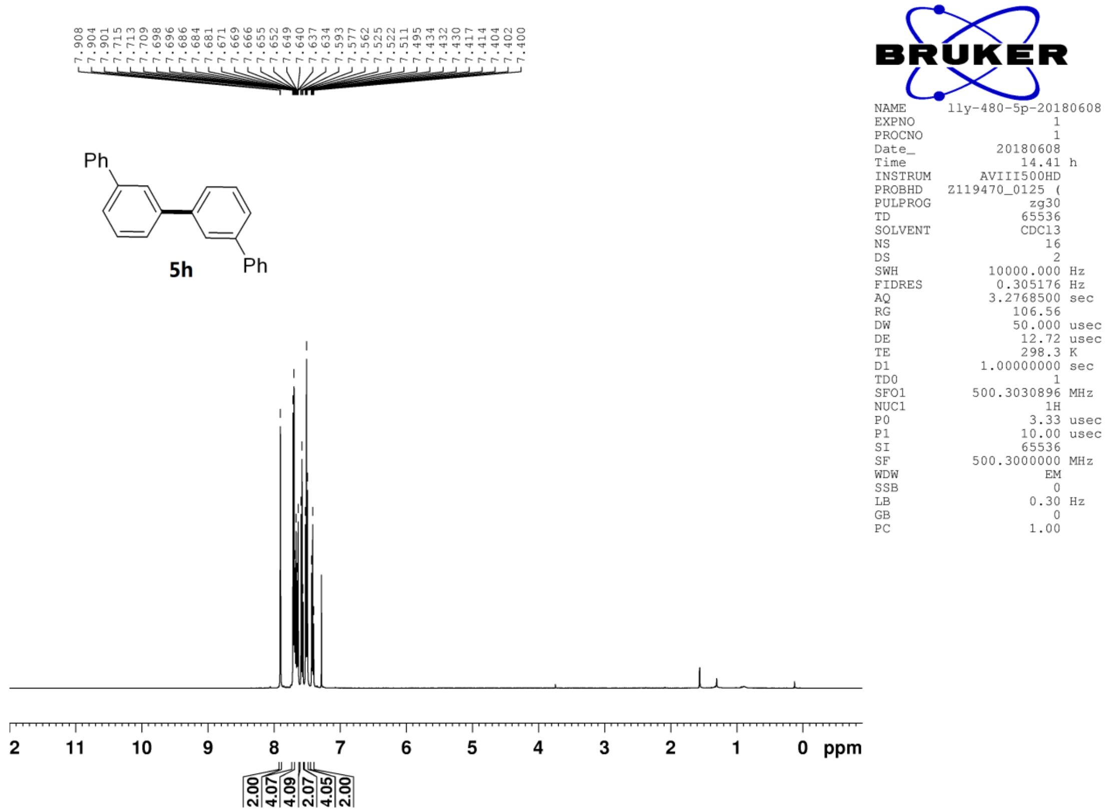

Supplementary Figure 72. ^{1}H NMR spectra for compound **5h**

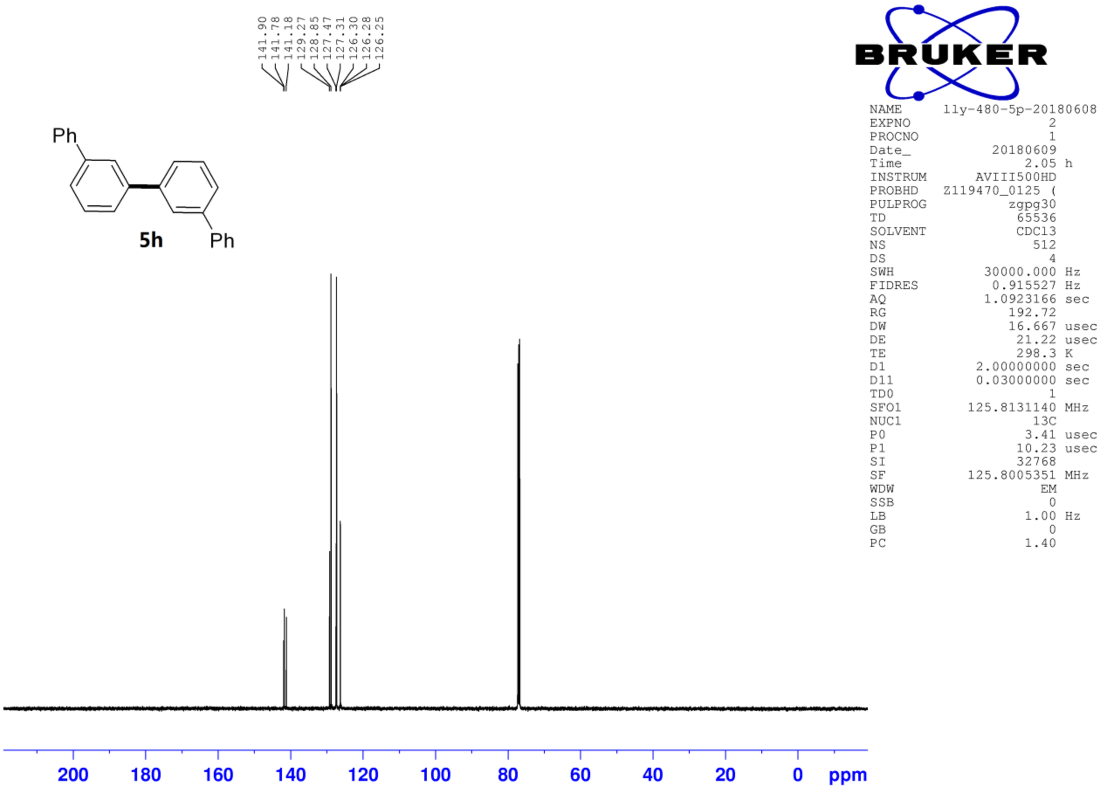

Supplementary Figure 73. ^{13}C NMR spectra for compound **5h**

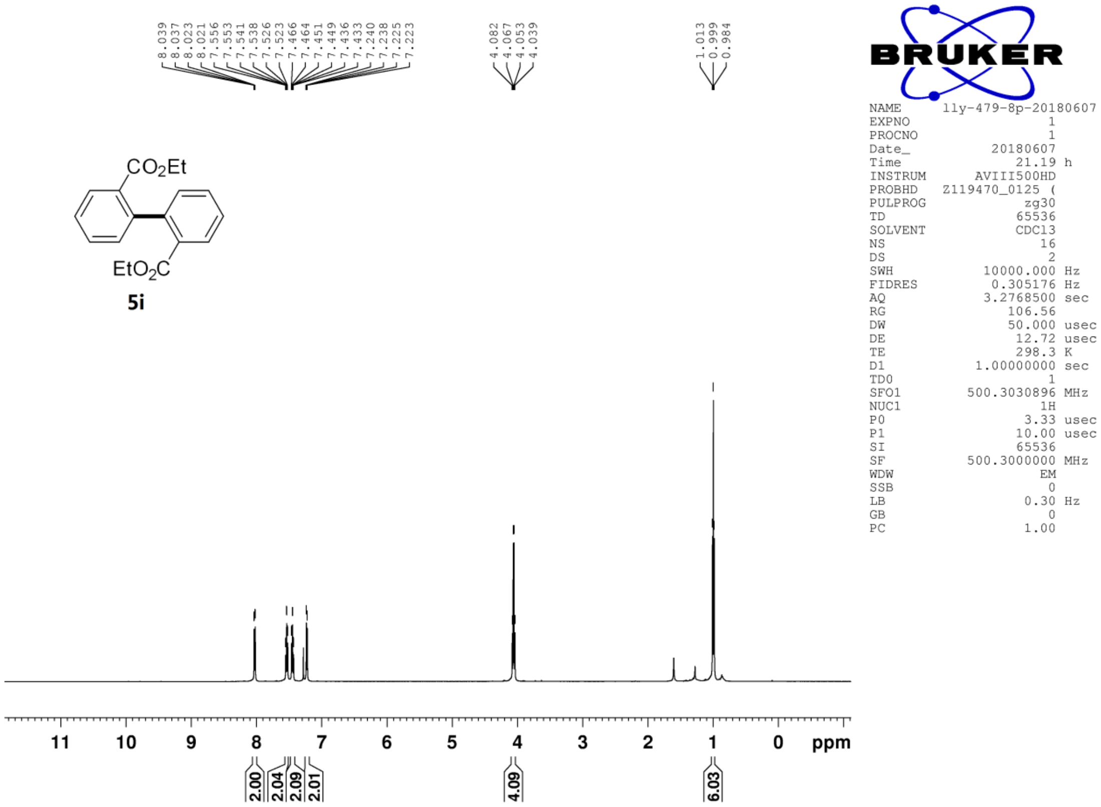

Supplementary Figure 74. ^{1}H NMR spectra for compound **5i**

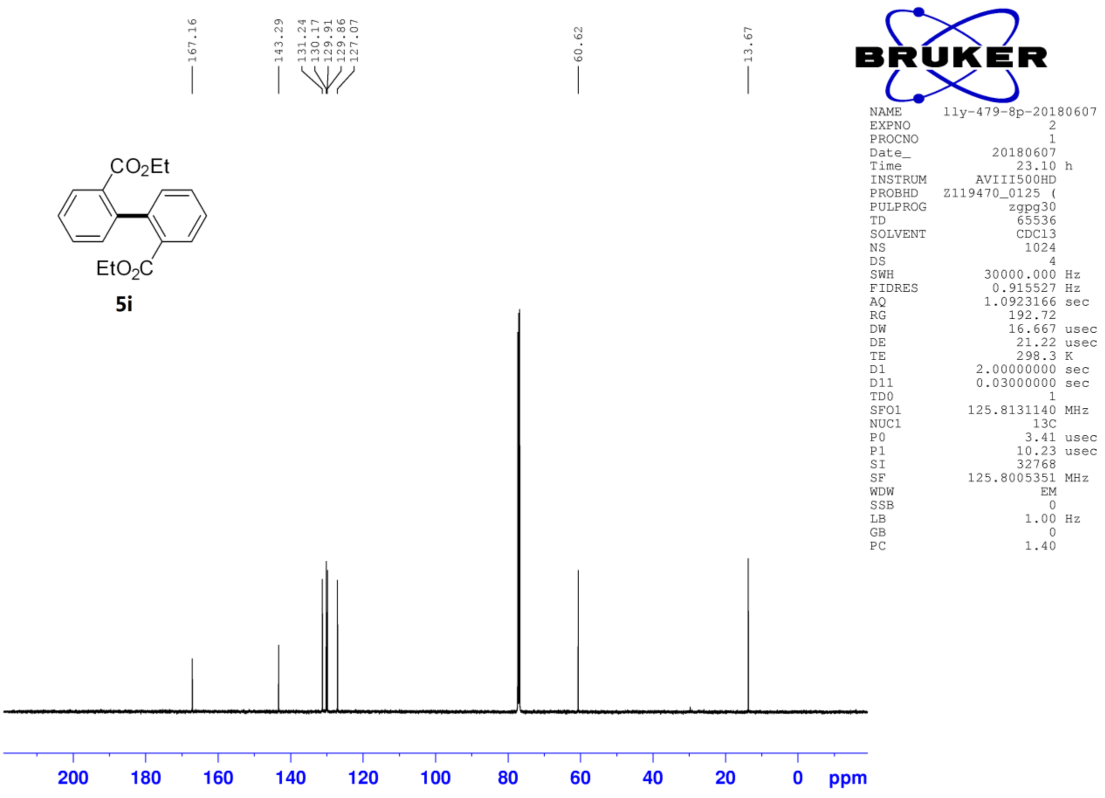

Supplementary Figure 75. ^{13}C NMR spectra for compound **5i**

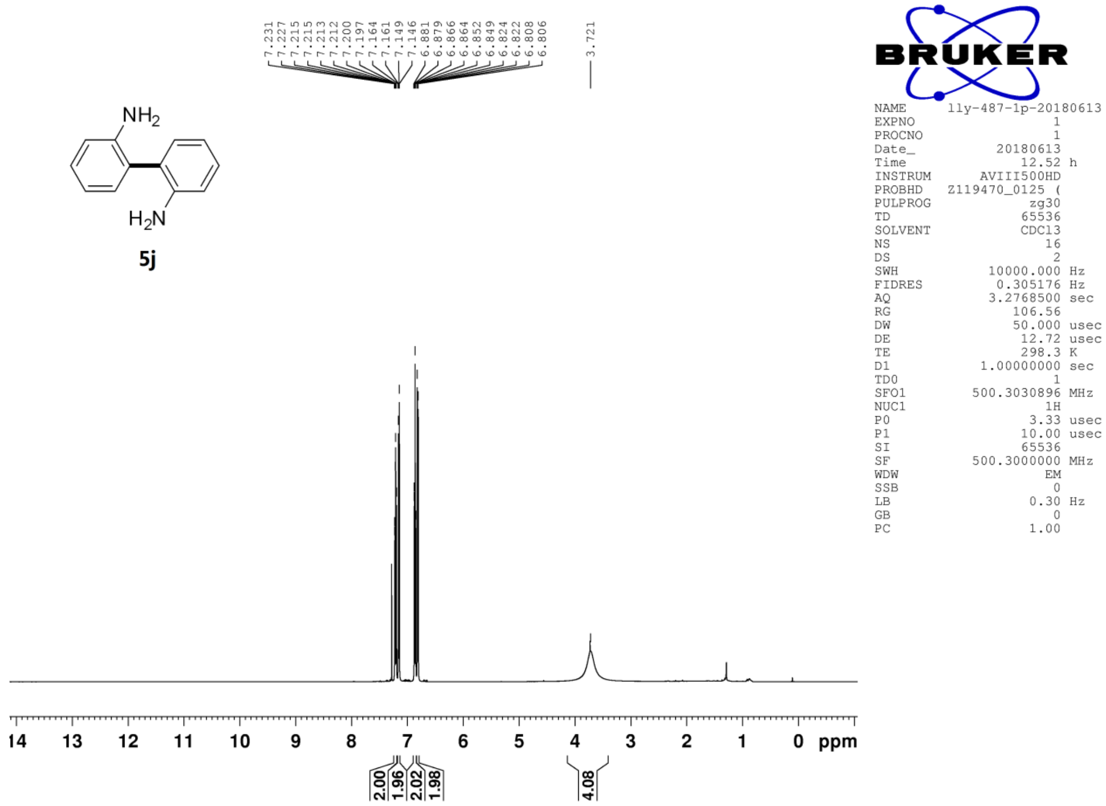

Supplementary Figure 76. ^{1}H NMR spectra for compound **5j**

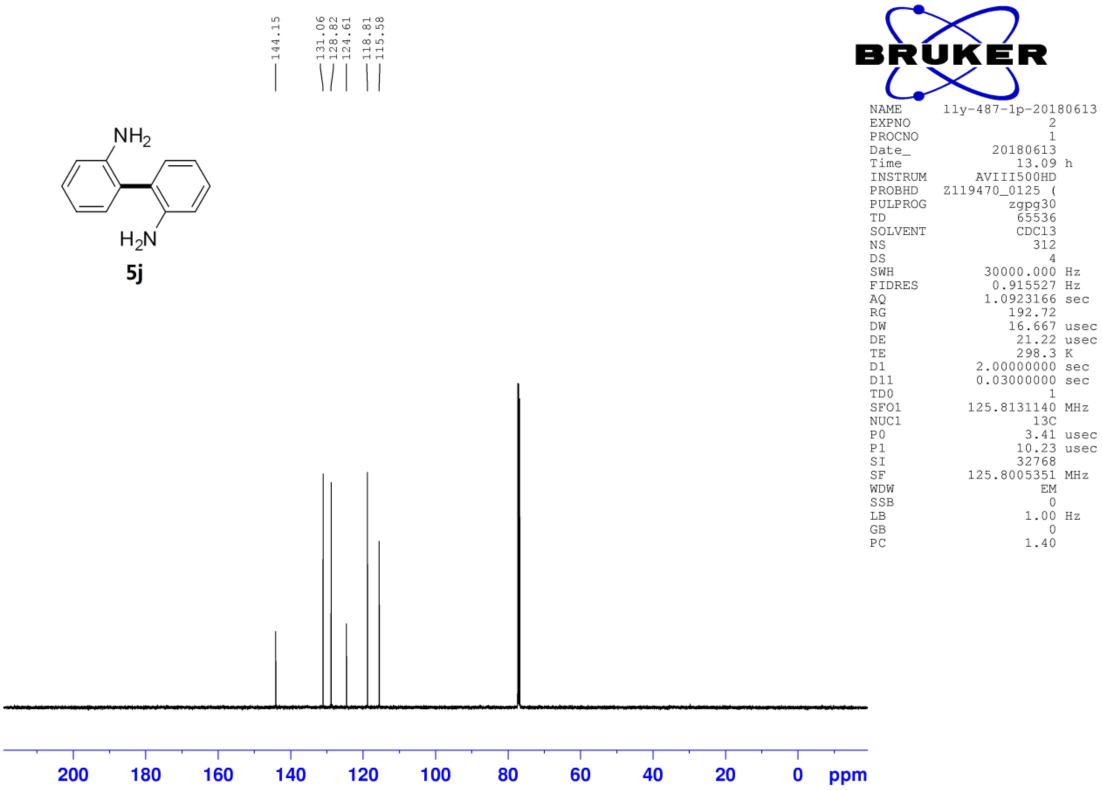

Supplementary Figure 77. ^{13}C NMR spectra for compound **5j**

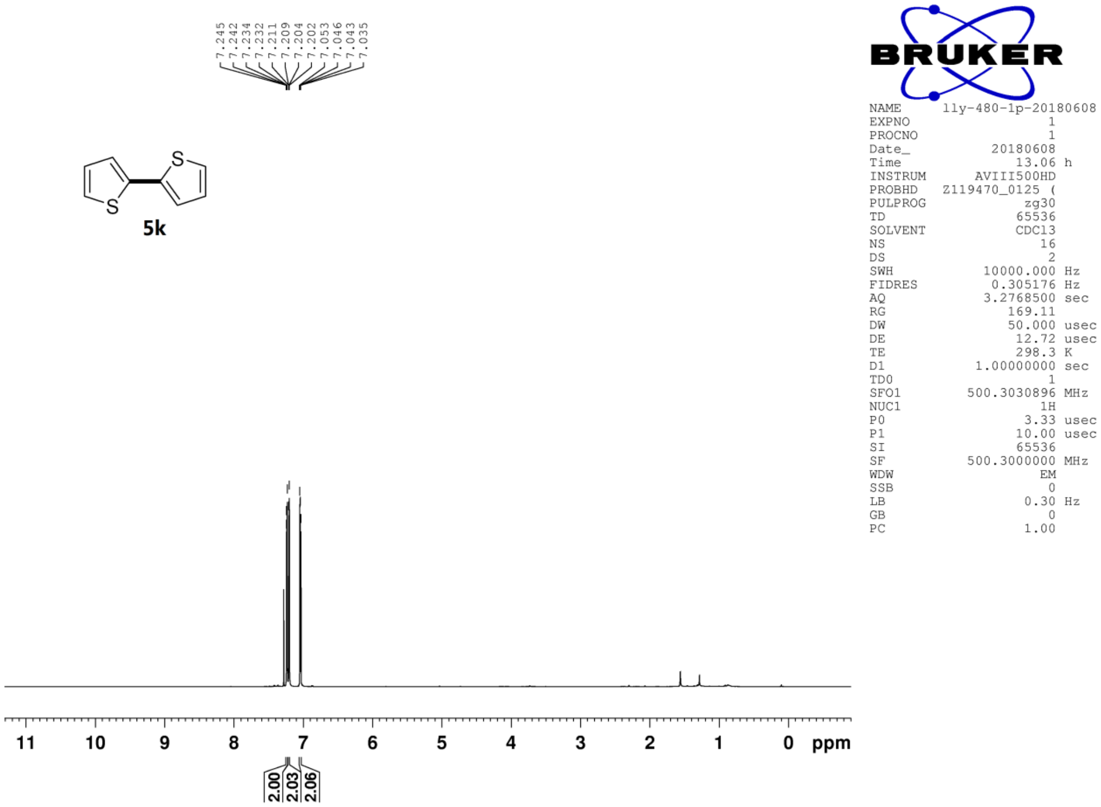

Supplementary Figure 78. ^{1}H NMR spectra for compound **5k**

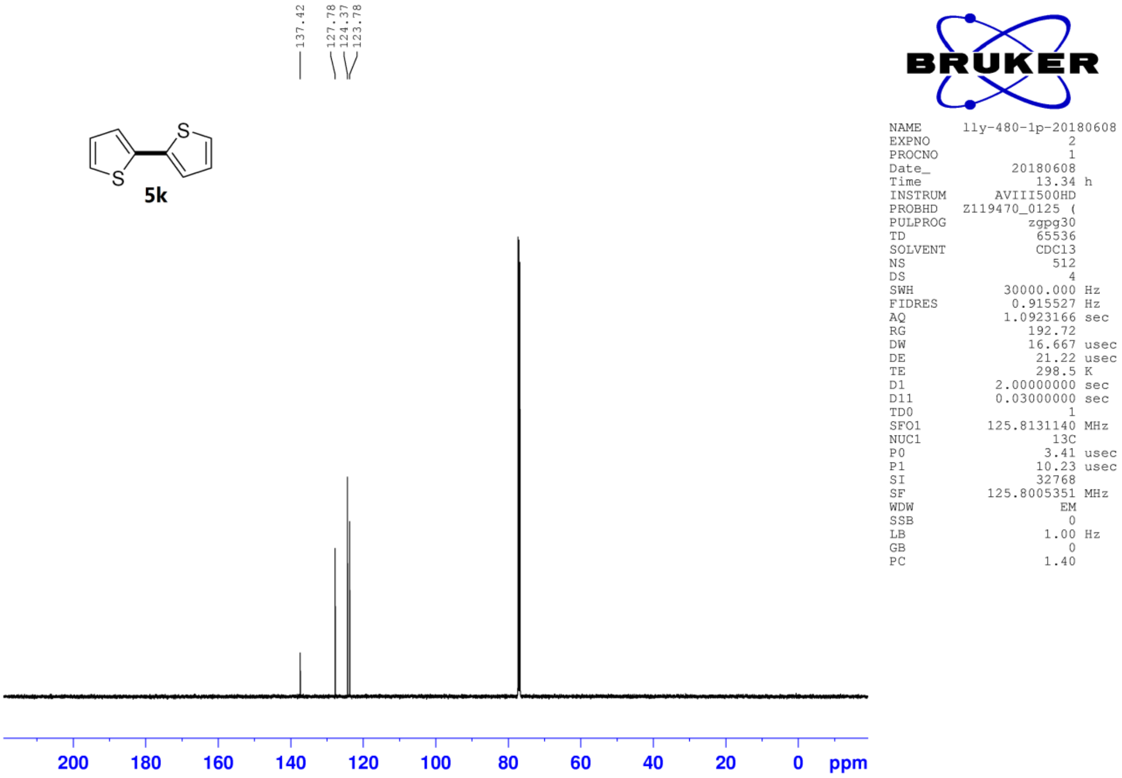

Supplementary Figure 79. ^{13}C NMR spectra for compound **5k**

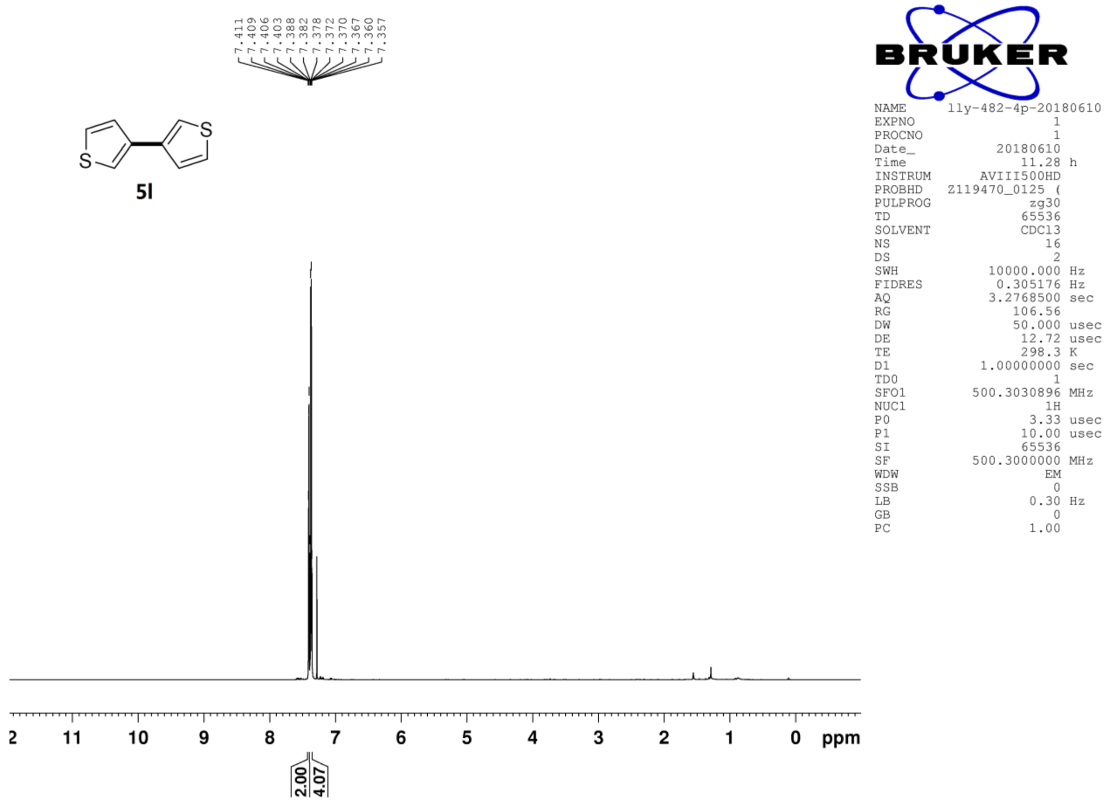

Supplementary Figure 80. ^{1}H NMR spectra for compound **5l**

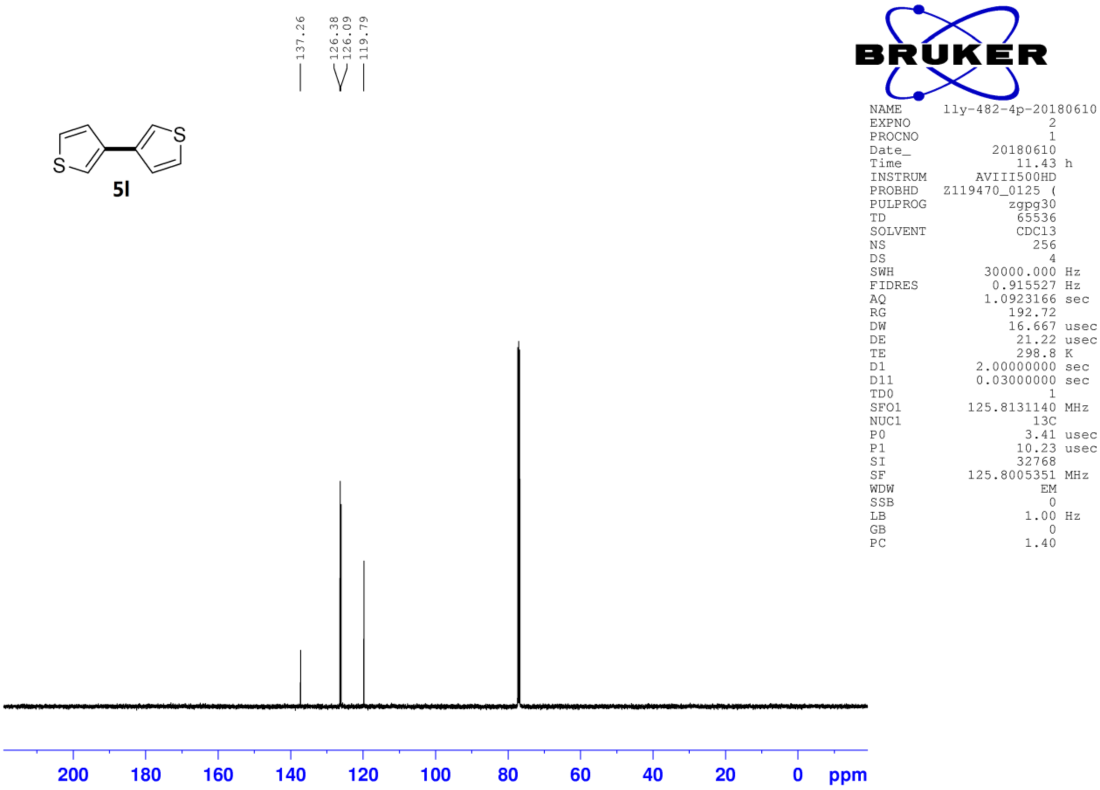

Supplementary Figure 81. ^{13}C NMR spectra for compound **5l**

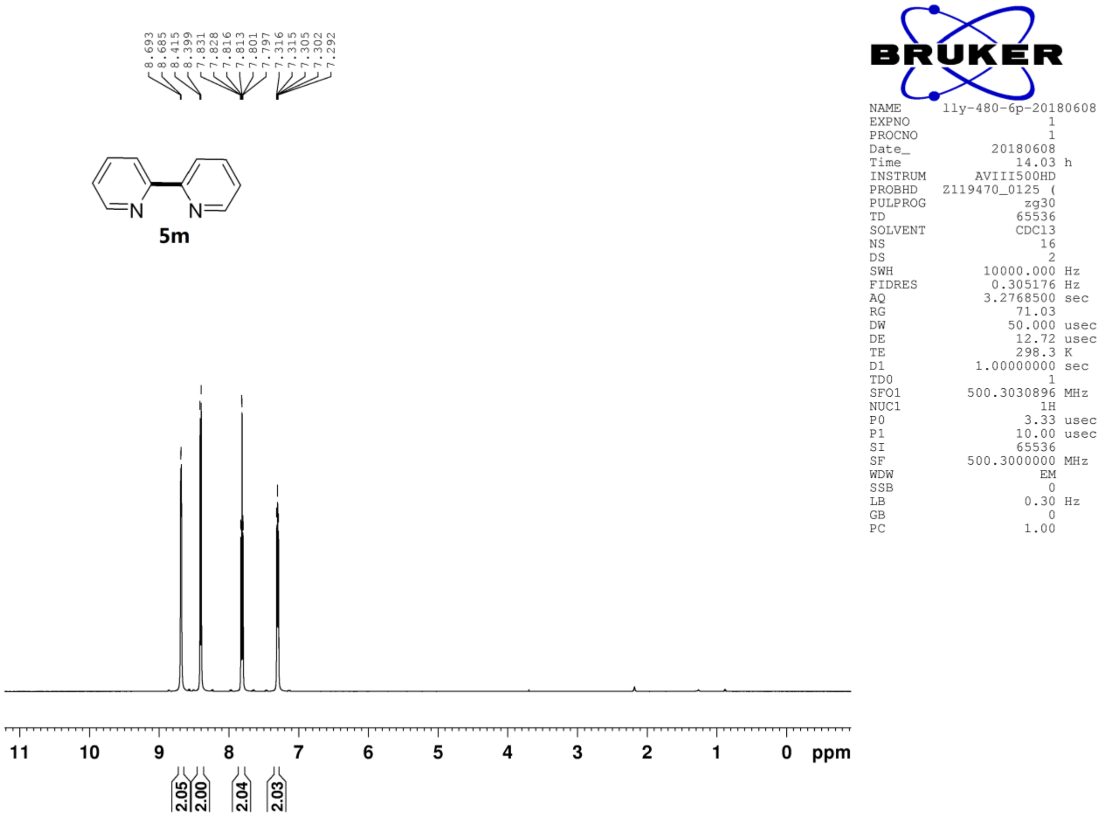

Supplementary Figure 82. ^{1}H NMR spectra for compound **5m**

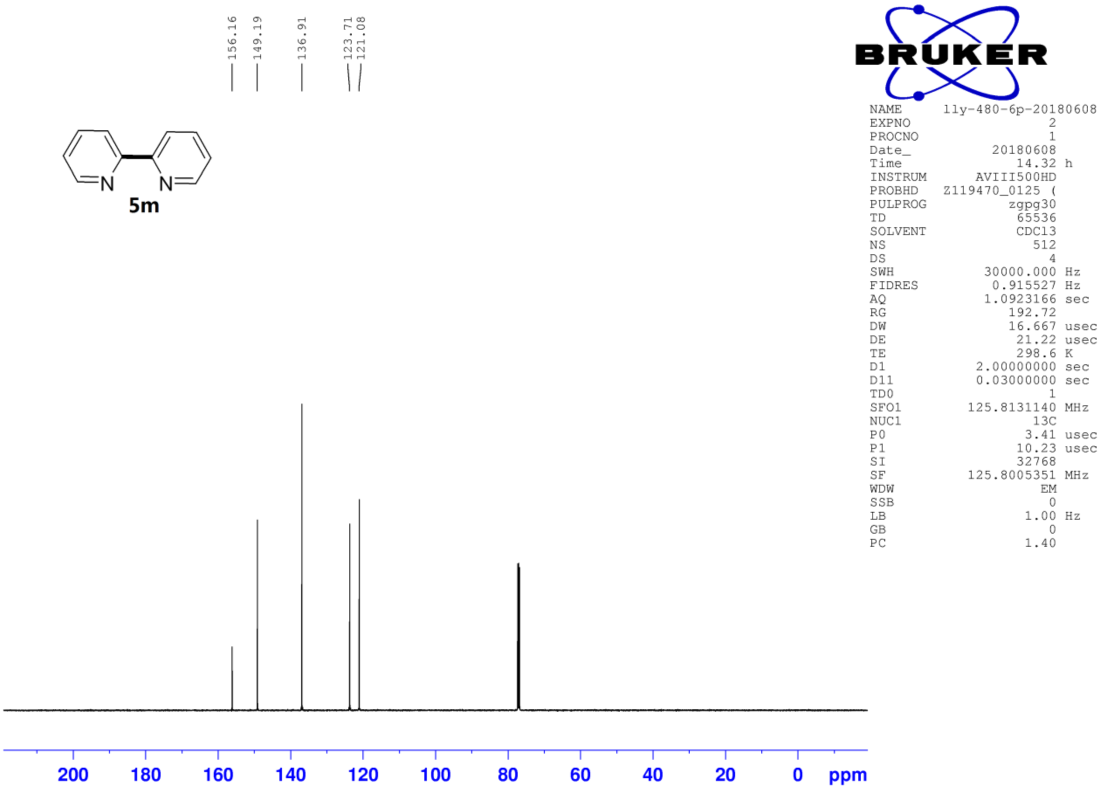

Supplementary Figure 83. ^{13}C NMR spectra for compound **5m**

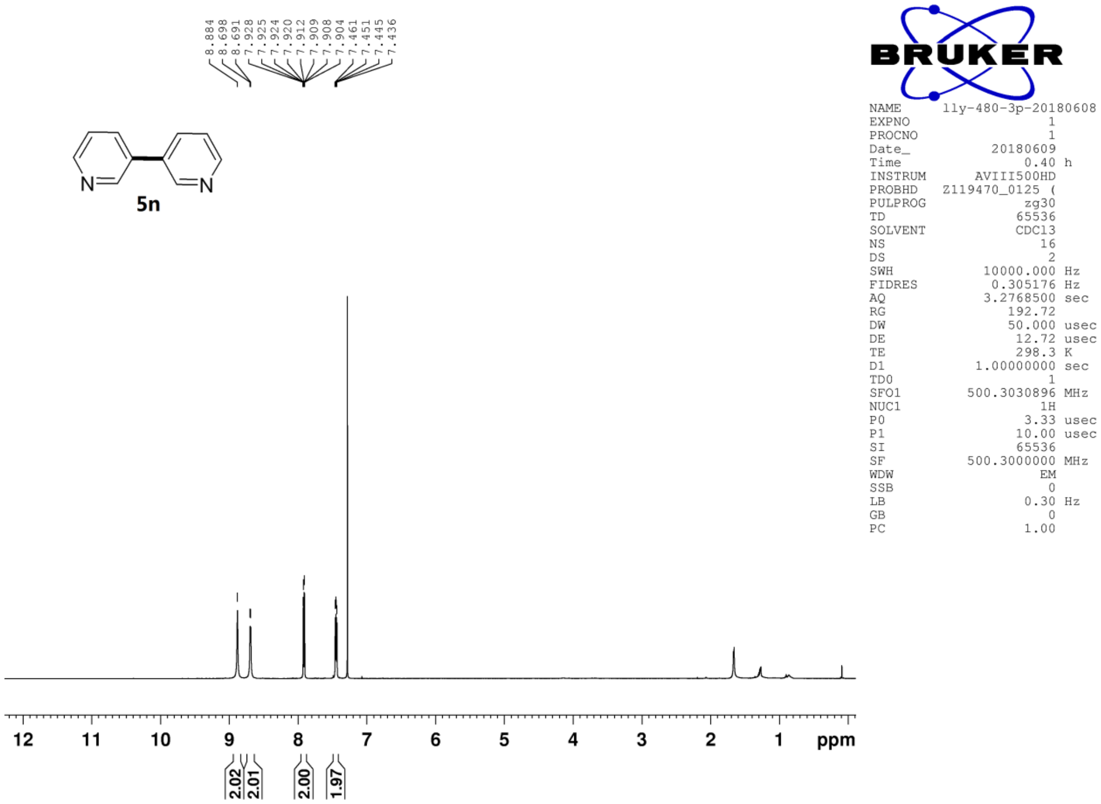

Supplementary Figure 84. ^{1}H NMR spectra for compound **5n**

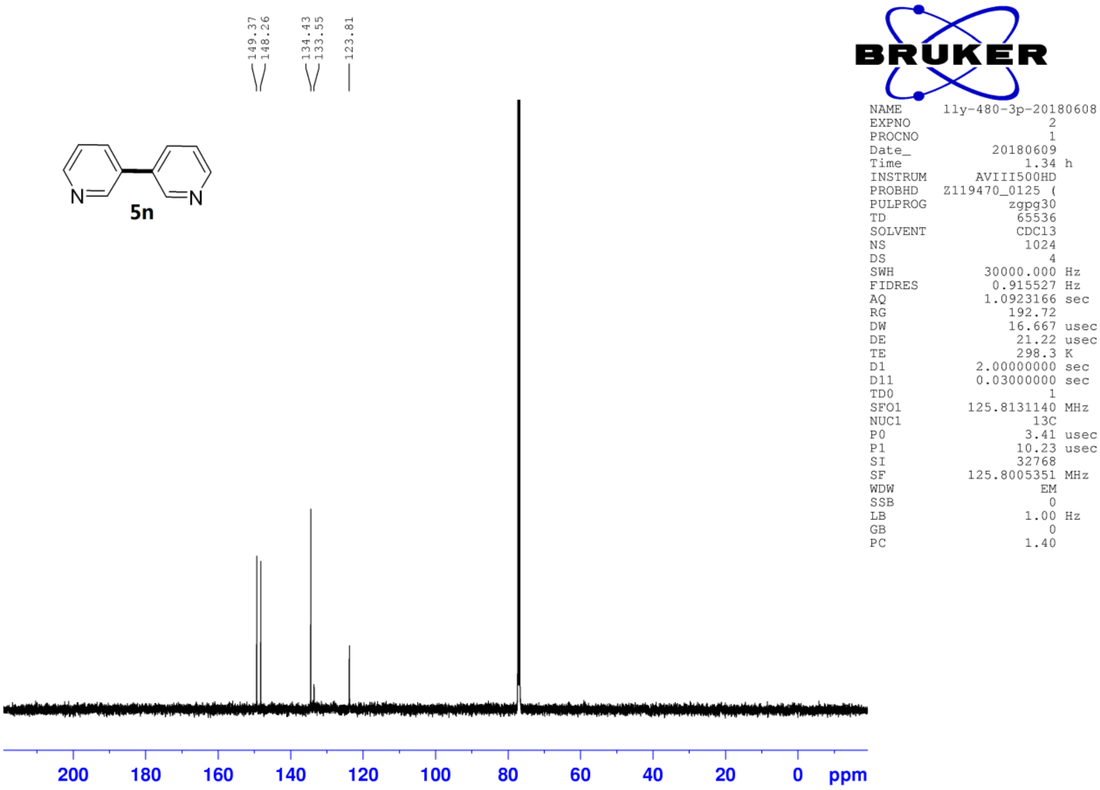

Supplementary Figure 85. ^{13}C NMR spectra for compound **5n**

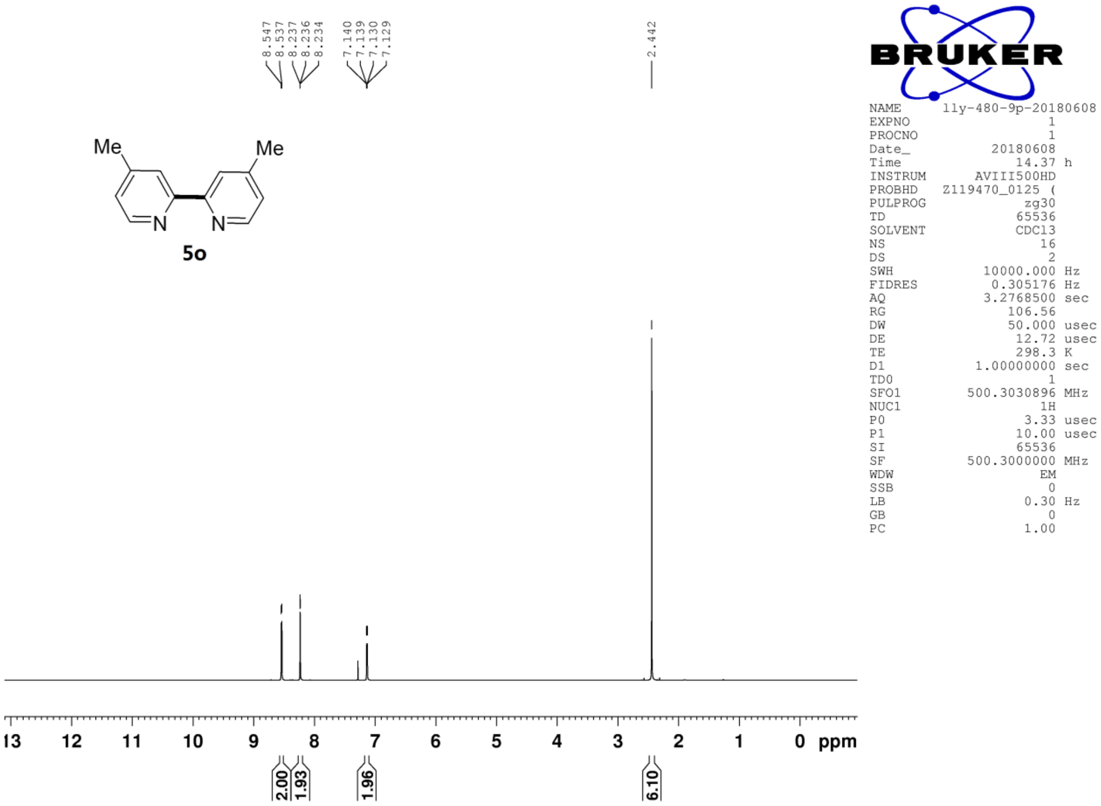

Supplementary Figure 86. ^{1}H NMR spectra for compound **5o**

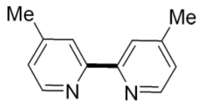

5o

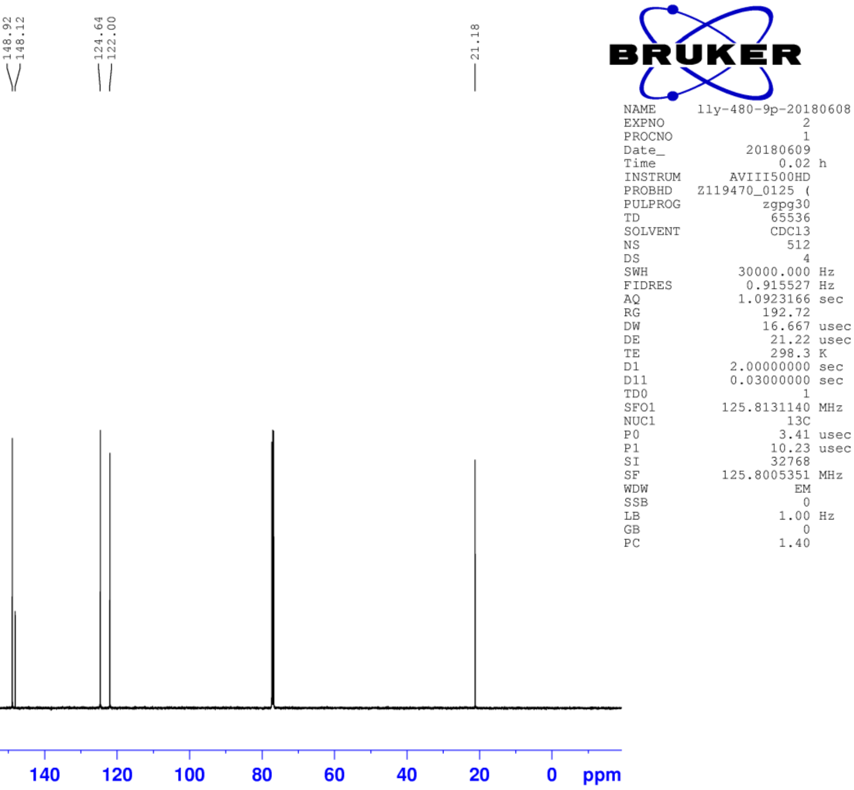

Supplementary Figure 87. ^{13}C NMR spectra for compound **5o**

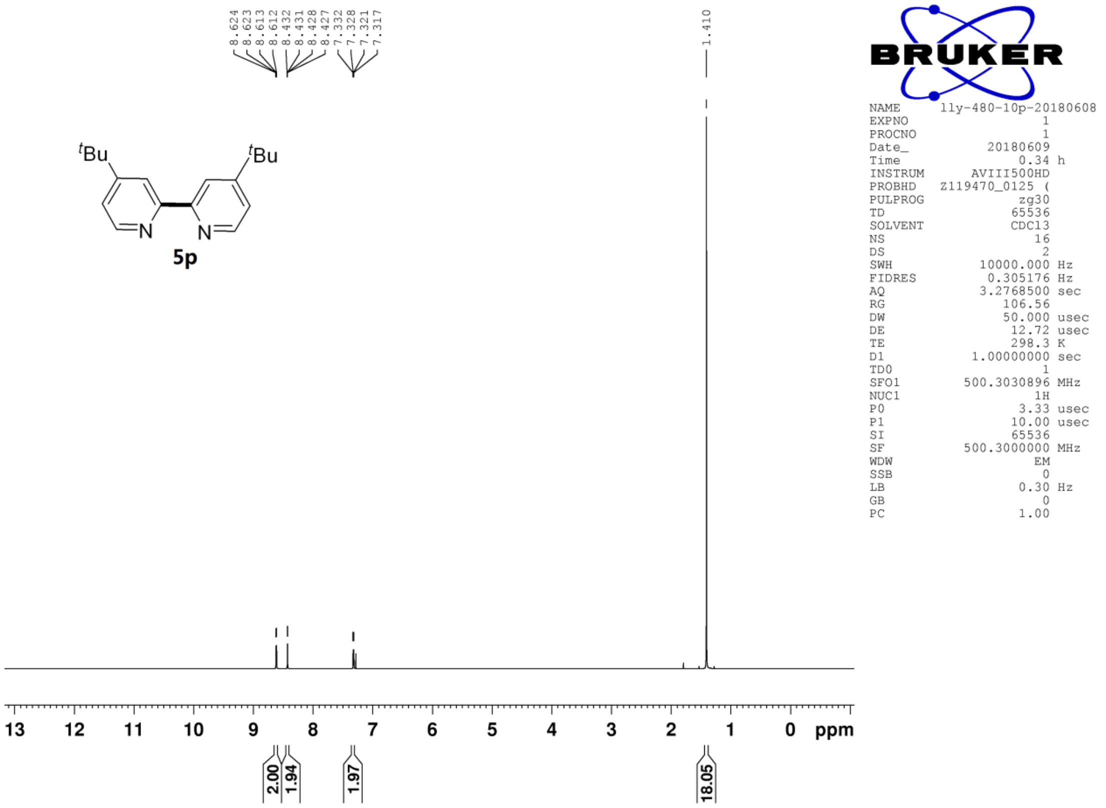

Supplementary Figure 88. ^{1}H NMR spectra for compound **5p**

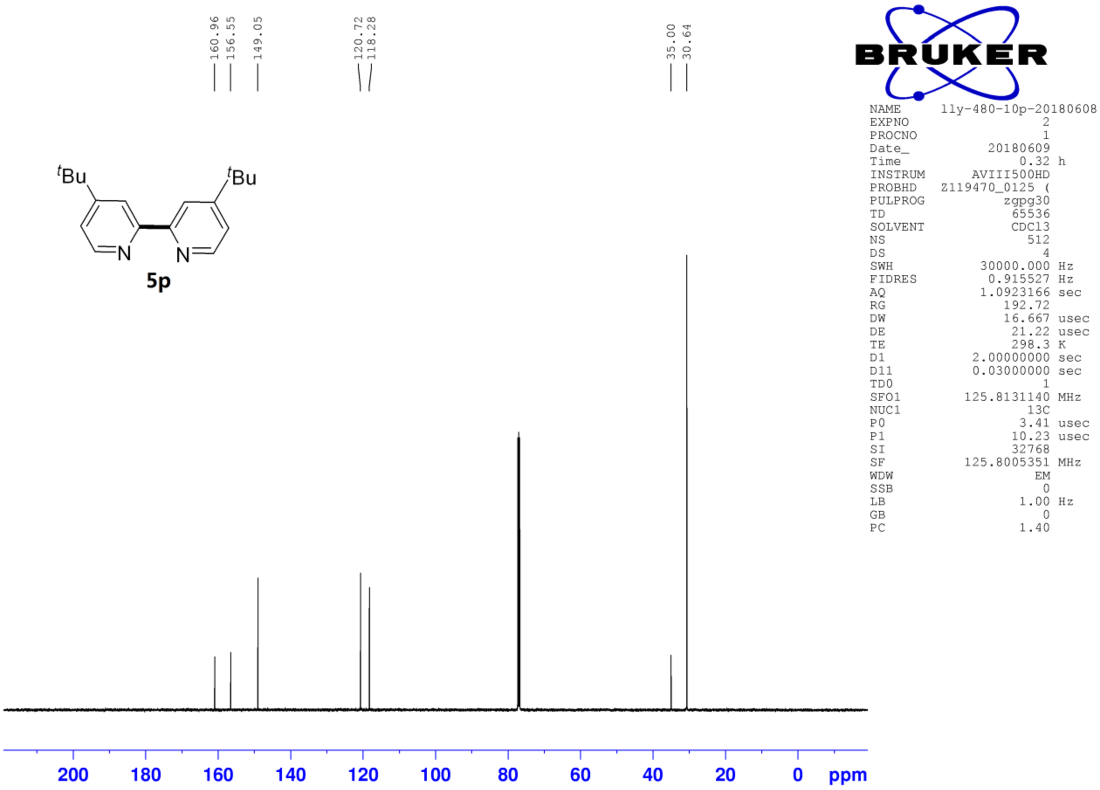

Supplementary Figure 89. ^{13}C NMR spectra for compound **5p**

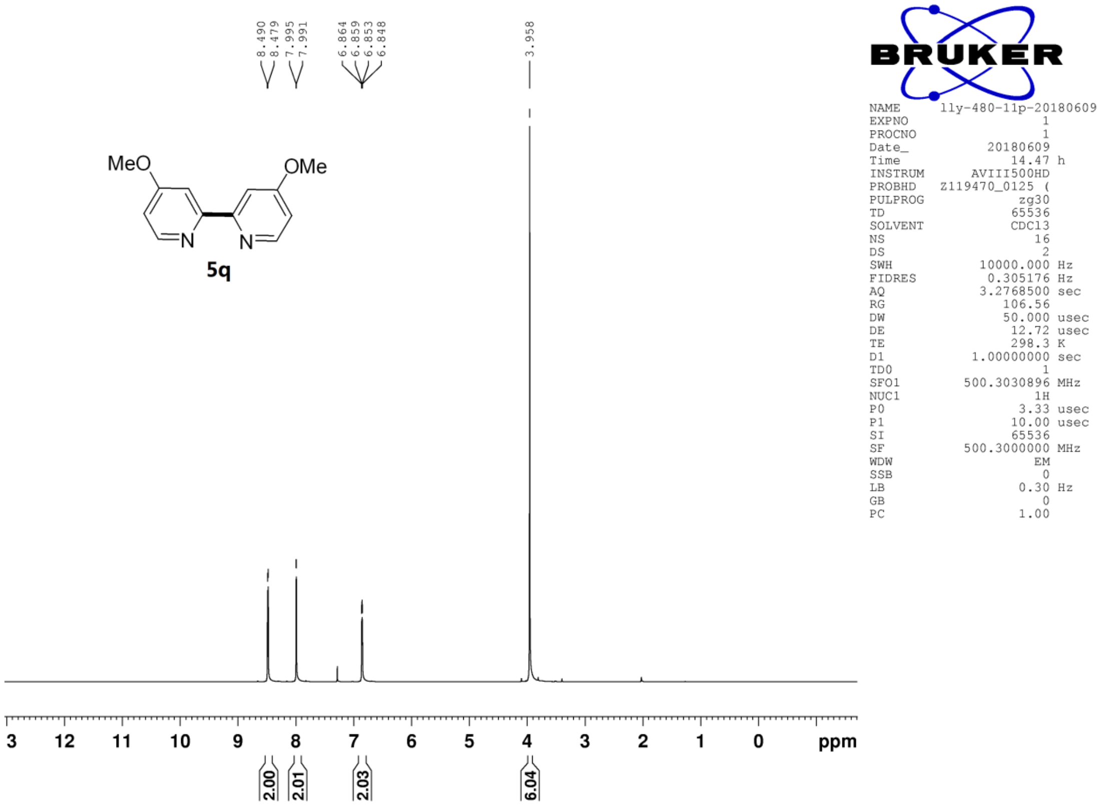

Supplementary Figure 90. ^{1}H NMR spectra for compound **5q**

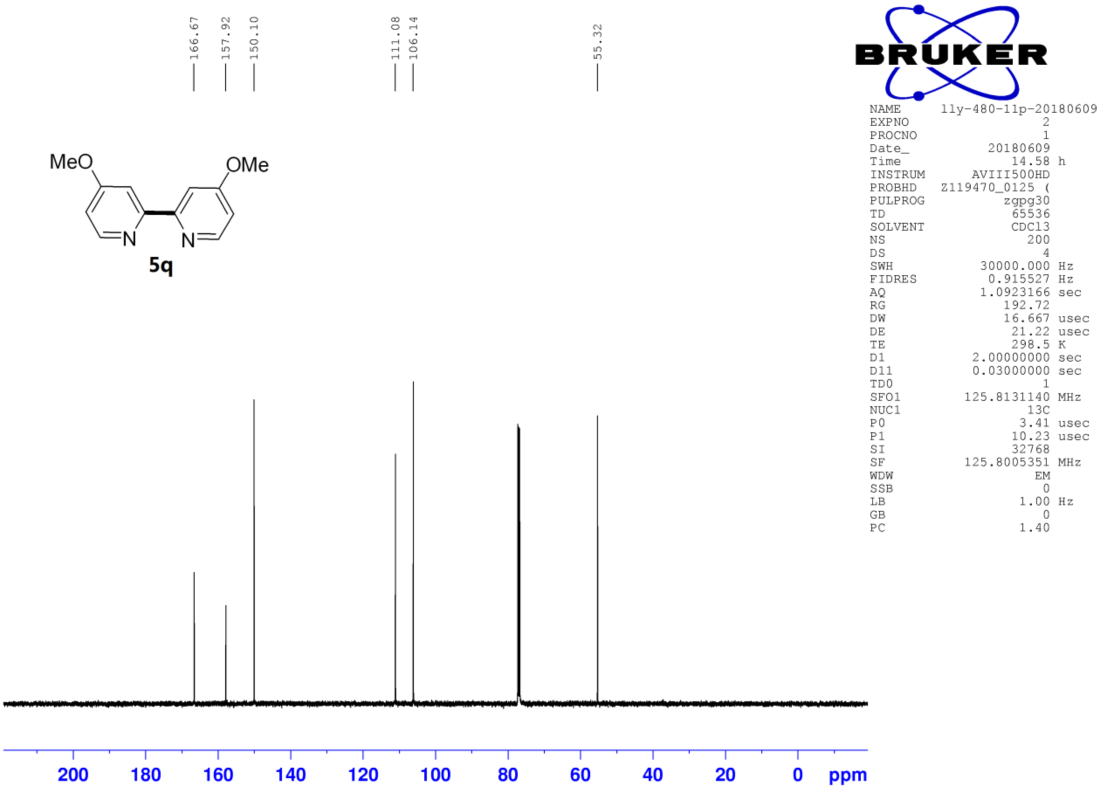

Supplementary Figure 91. ^{13}C NMR spectra for compound **5q**

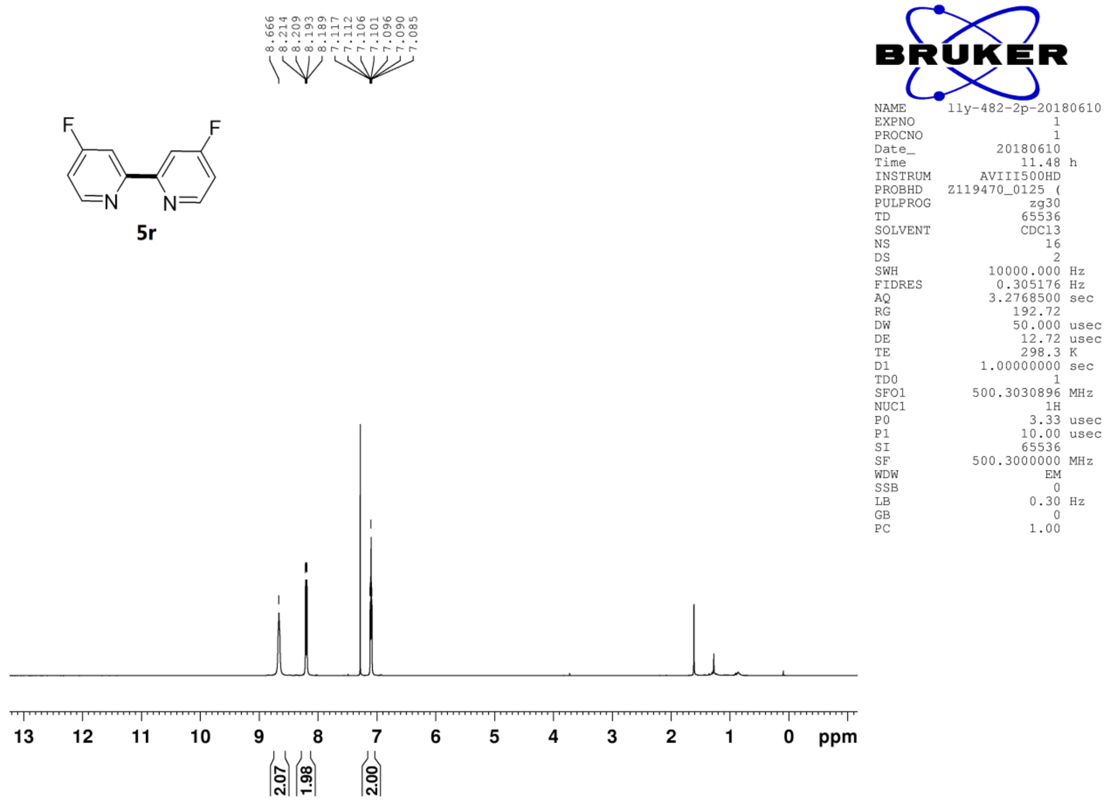

Supplementary Figure 92. ^{1}H NMR spectra for compound **5r**

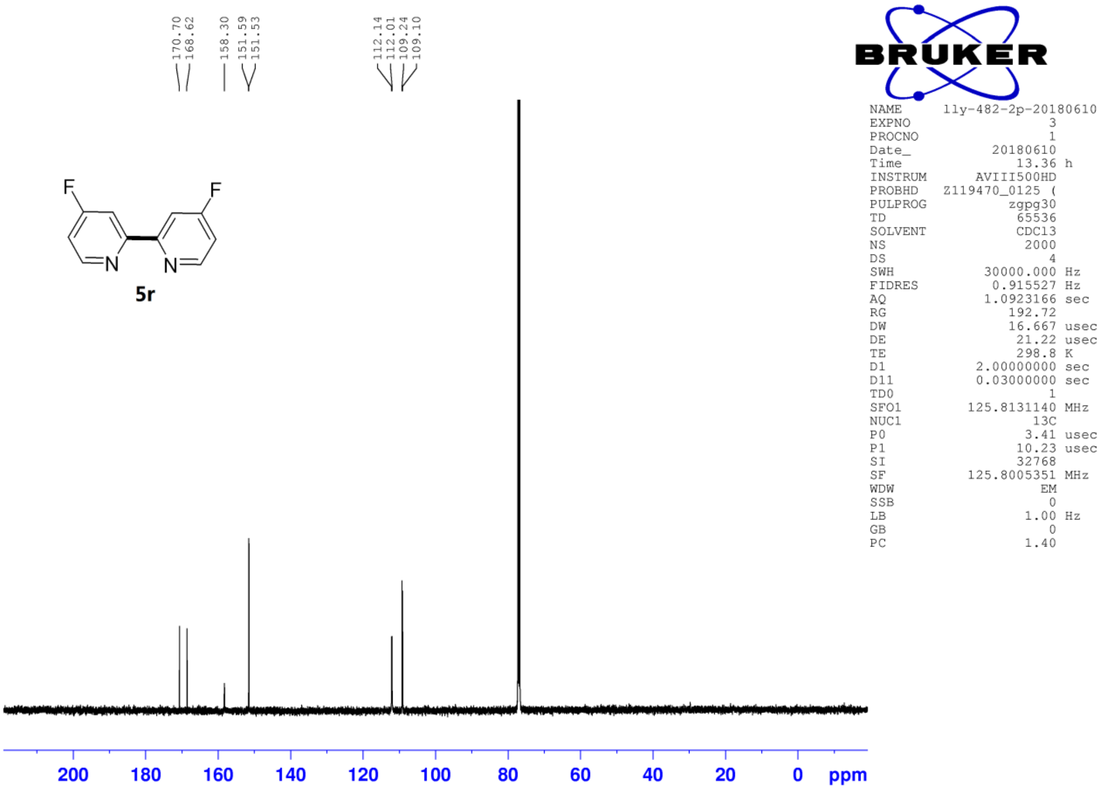

Supplementary Figure 93. ^{13}C NMR spectra for compound **5r**

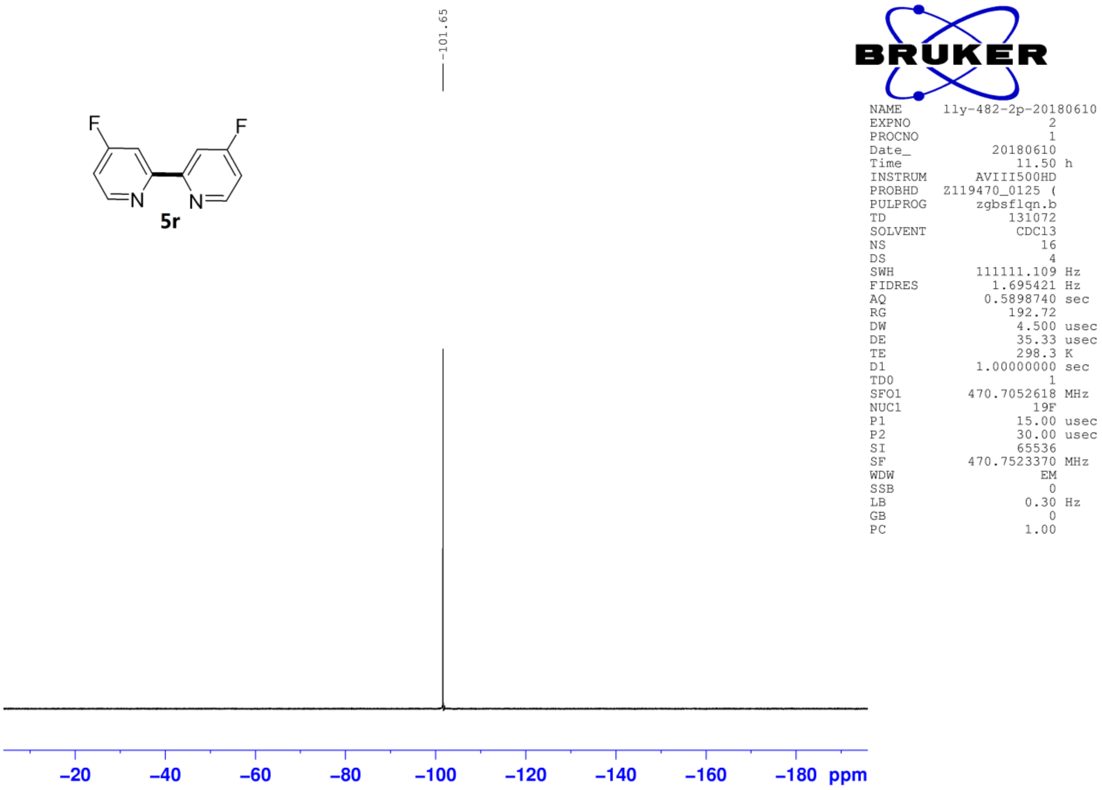

Supplementary Figure 94. ^{19}F NMR spectra for compound **5r**

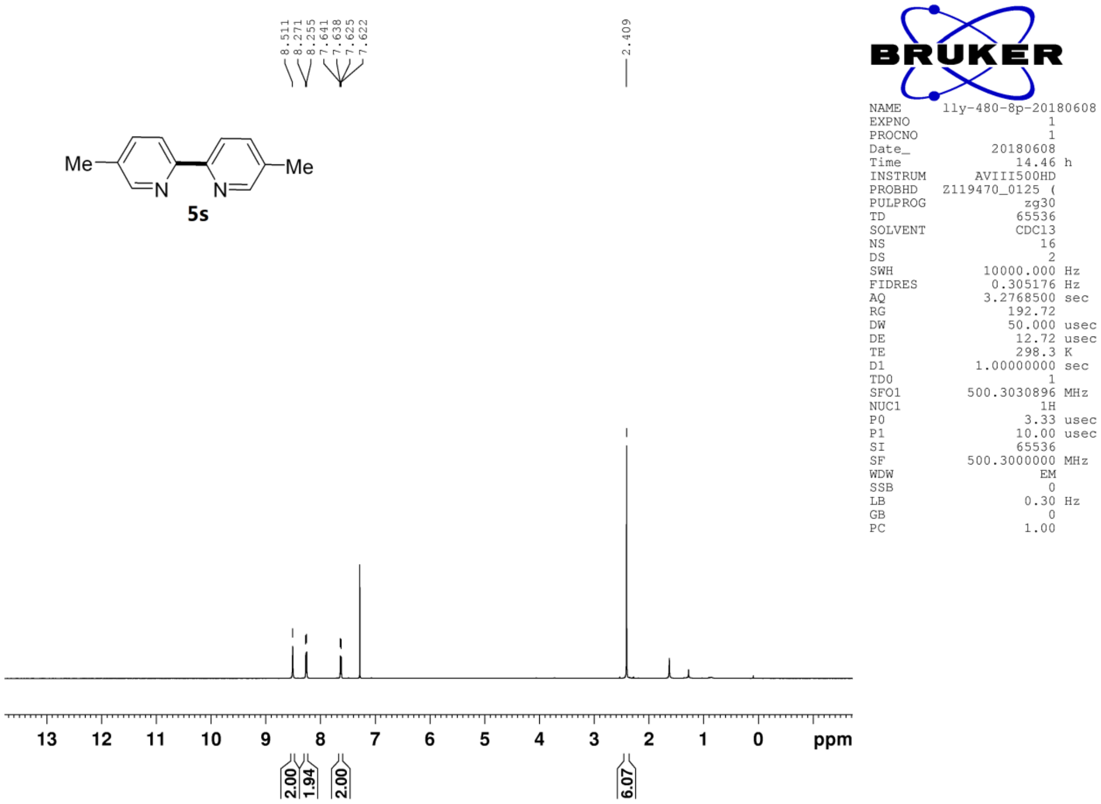

Supplementary Figure 95. ^{1}H NMR spectra for compound **5s**

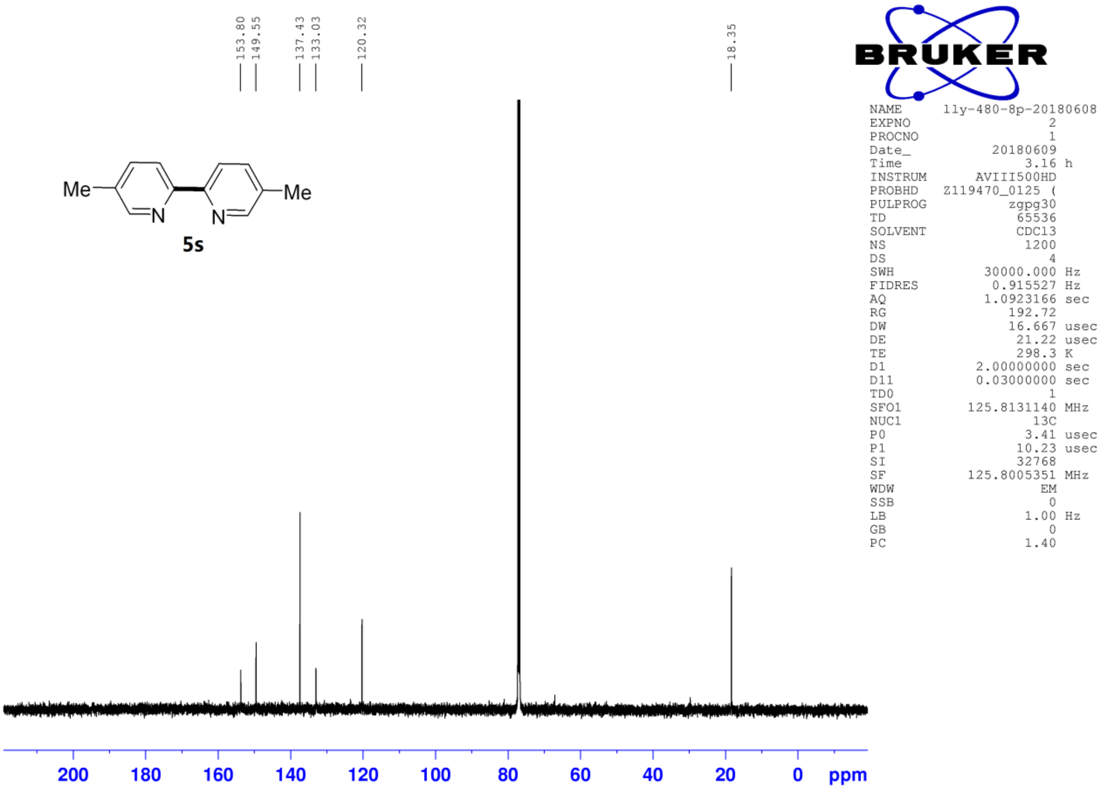

Supplementary Figure 96. ^{13}C NMR spectra for compound **5s**

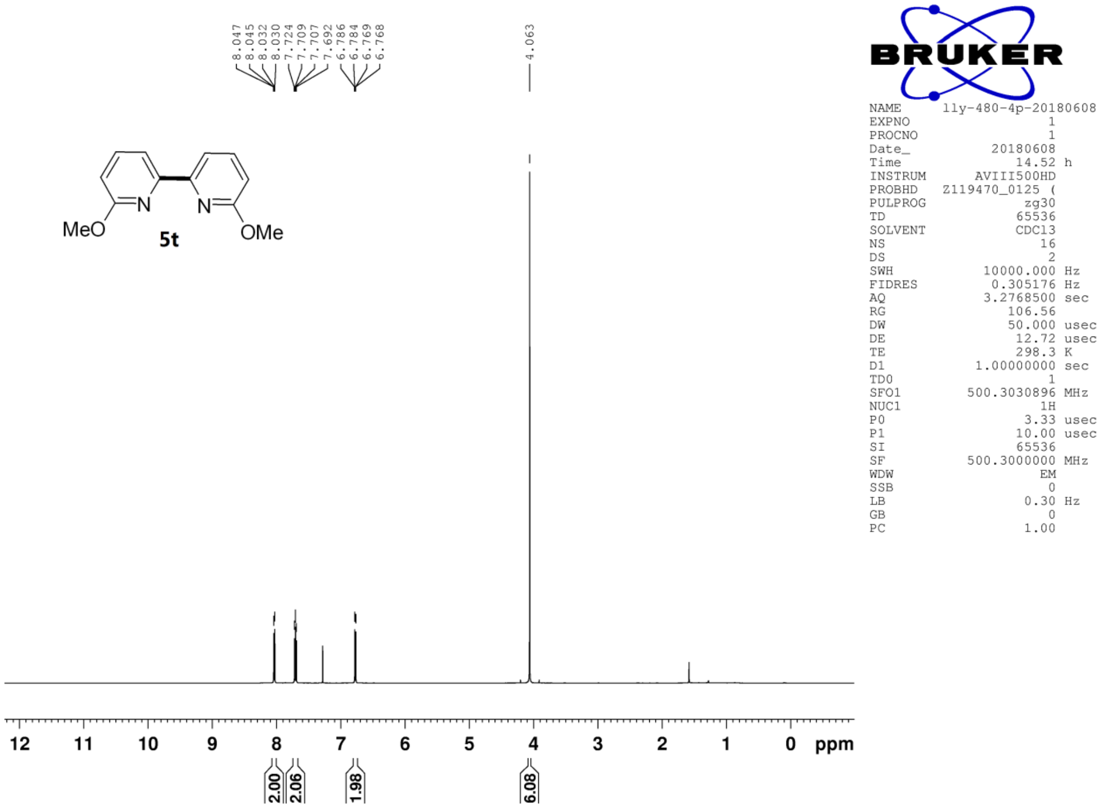

Supplementary Figure 97. ^{1}H NMR spectra for compound **5t**

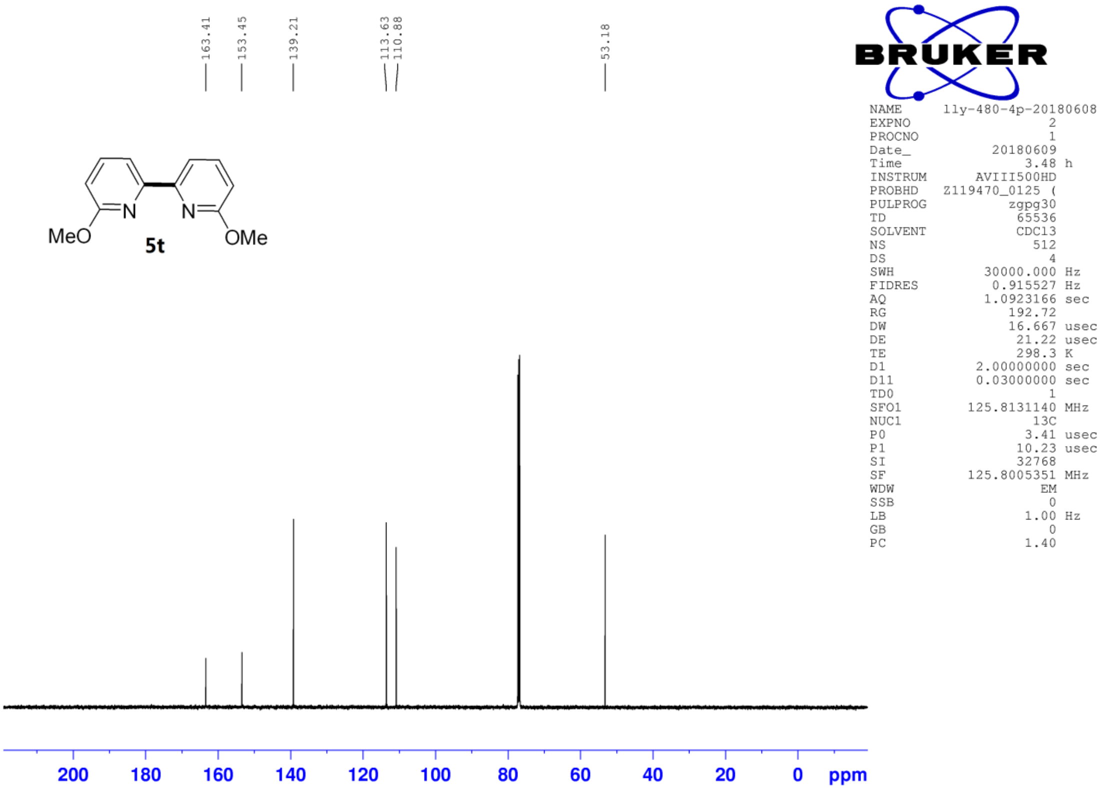

Supplementary Figure 98. ^{13}C NMR spectra for compound **5t**

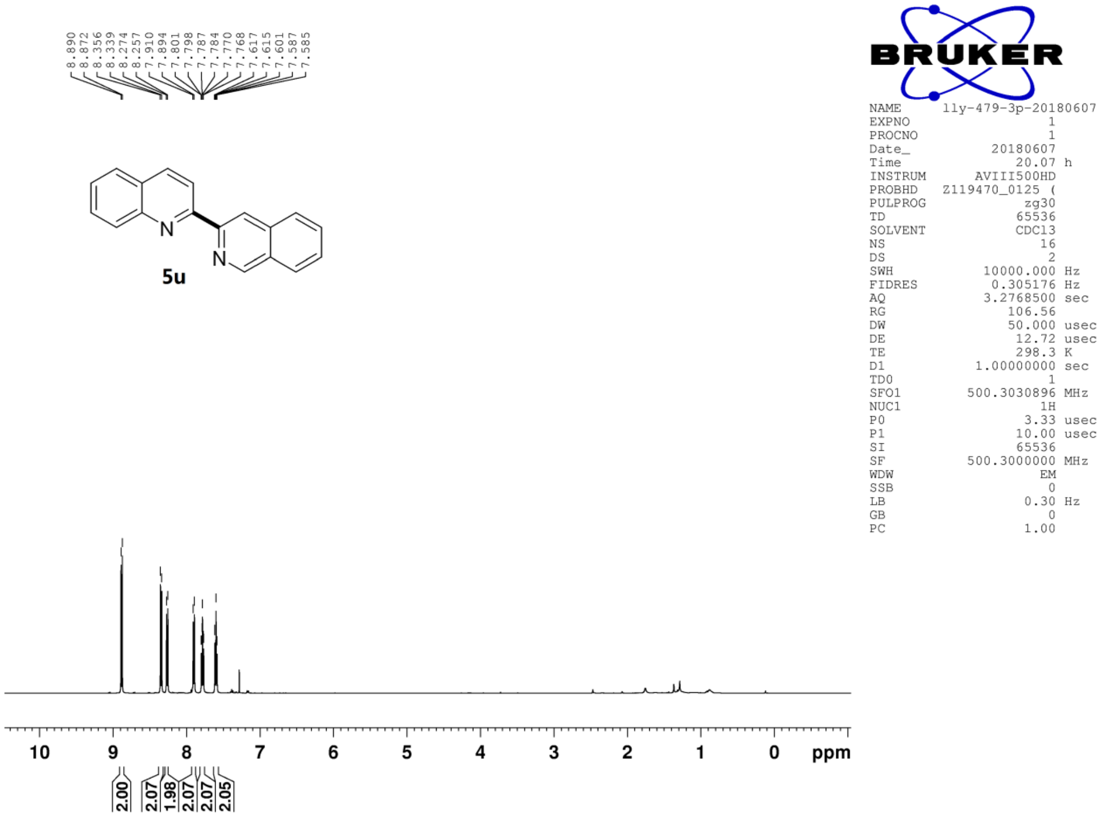

Supplementary Figure 99. ^{1}H NMR spectra for compound **5u**

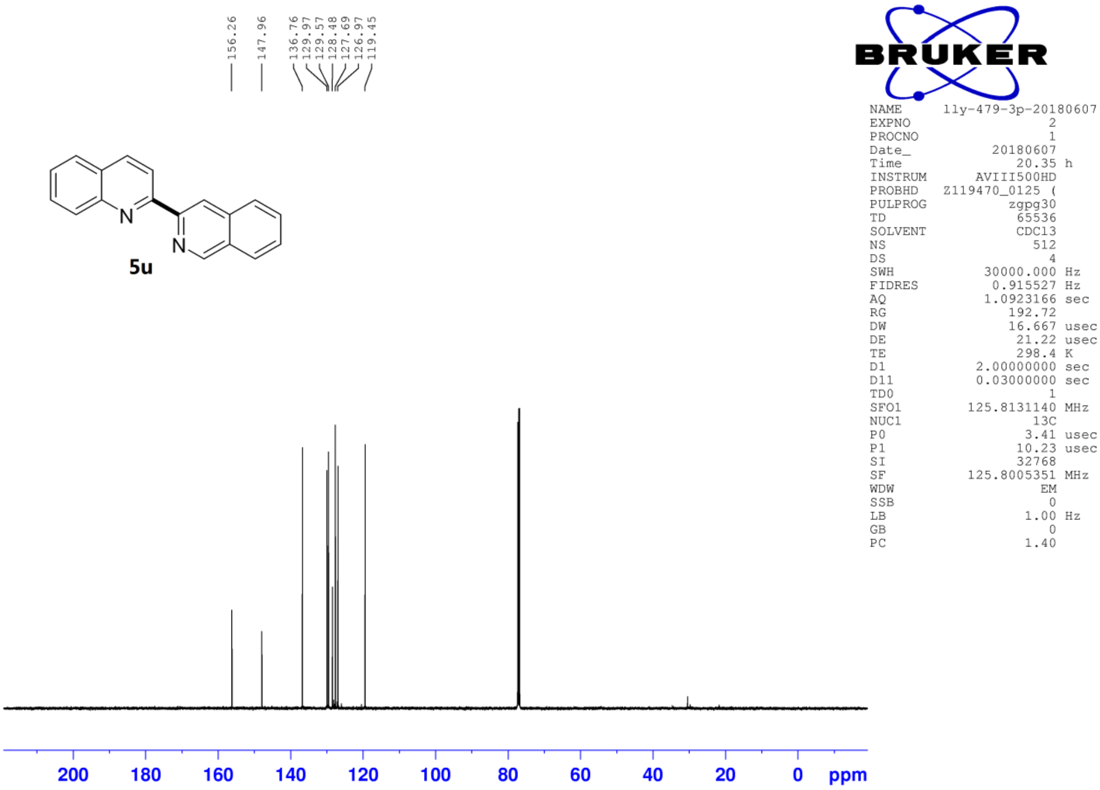

Supplementary Figure 100. ^{13}C NMR spectra for compound **5u**

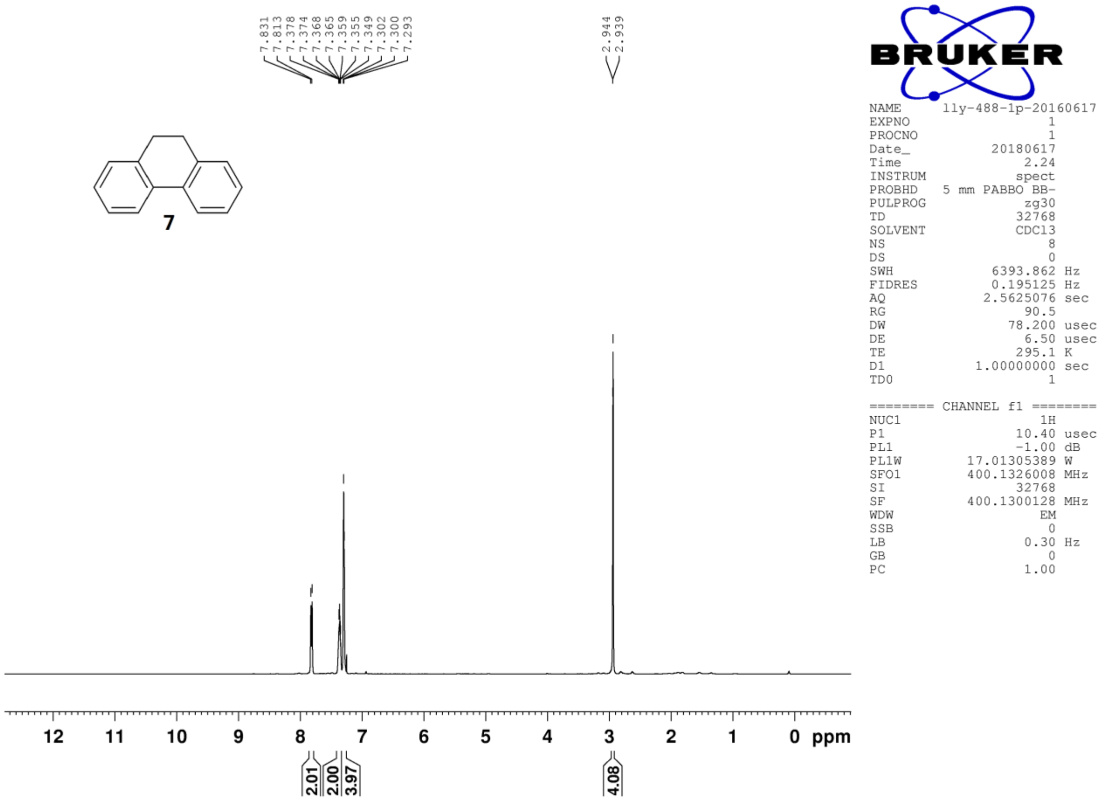

Supplementary Figure 101. ^{1}H NMR spectra for compound **7**

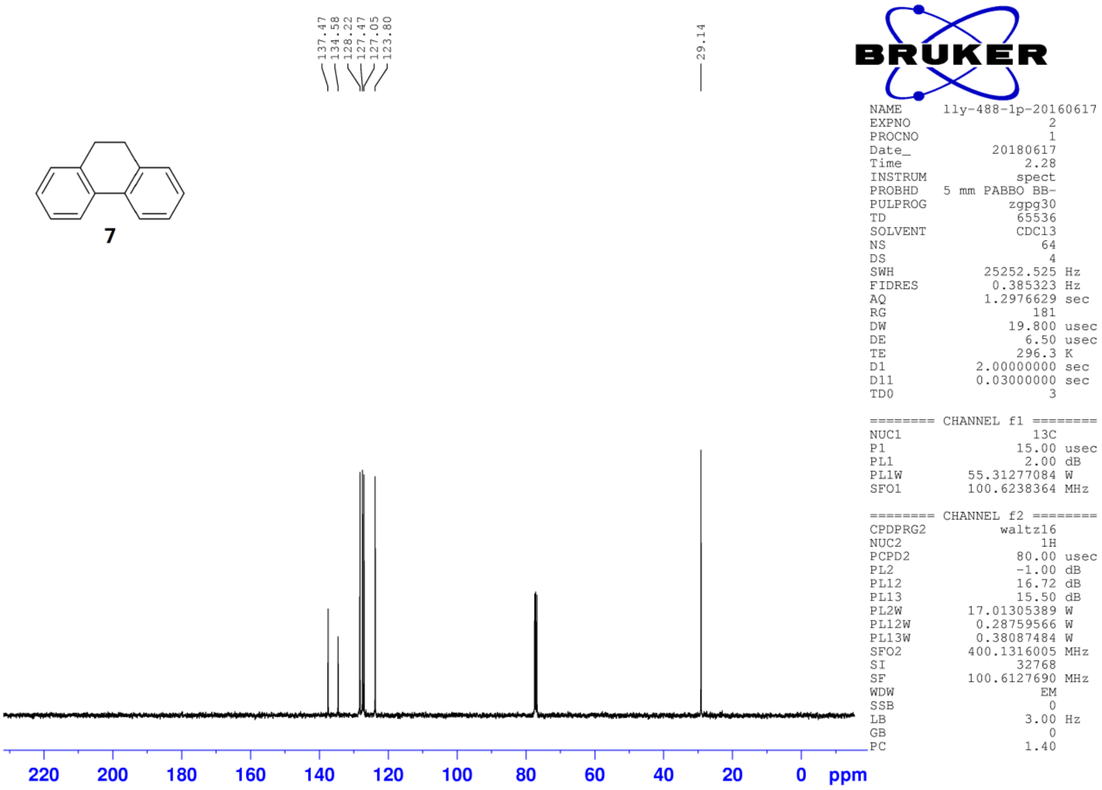

Supplementary Figure 102. ^{13}C NMR spectra for compound **7**

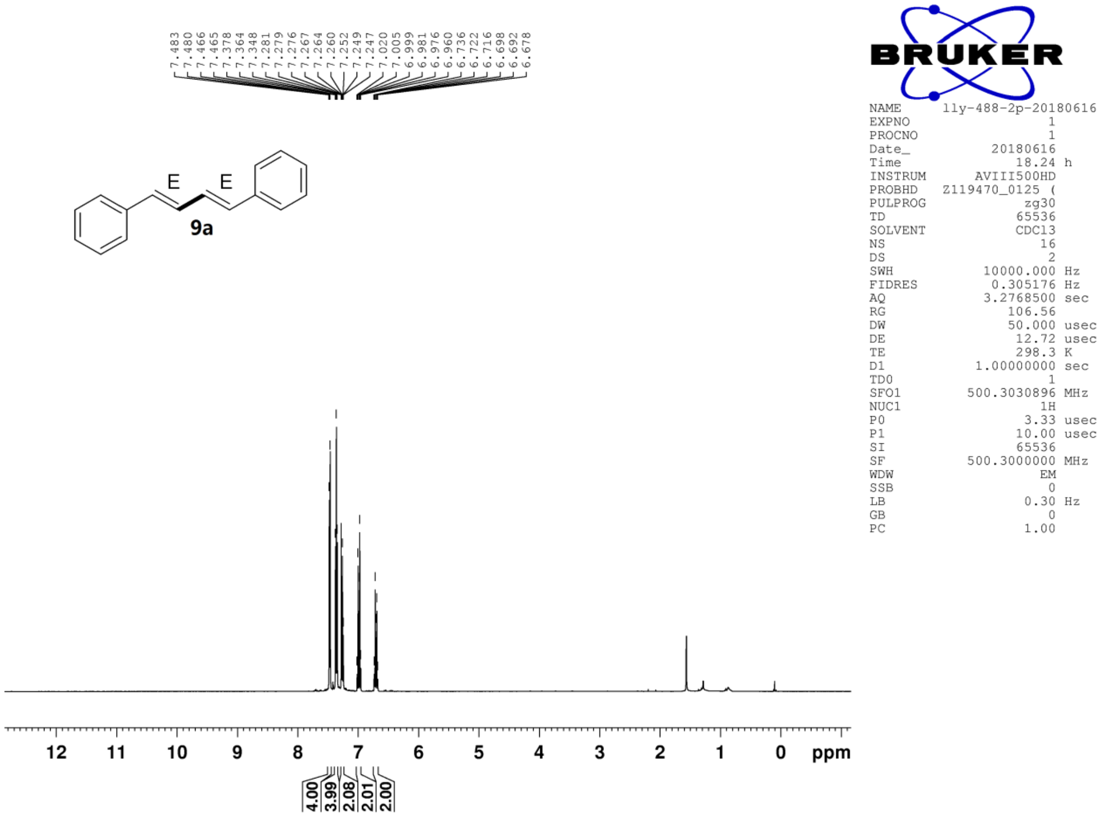

Supplementary Figure 103. ^{1}H NMR spectra for compound **9a**

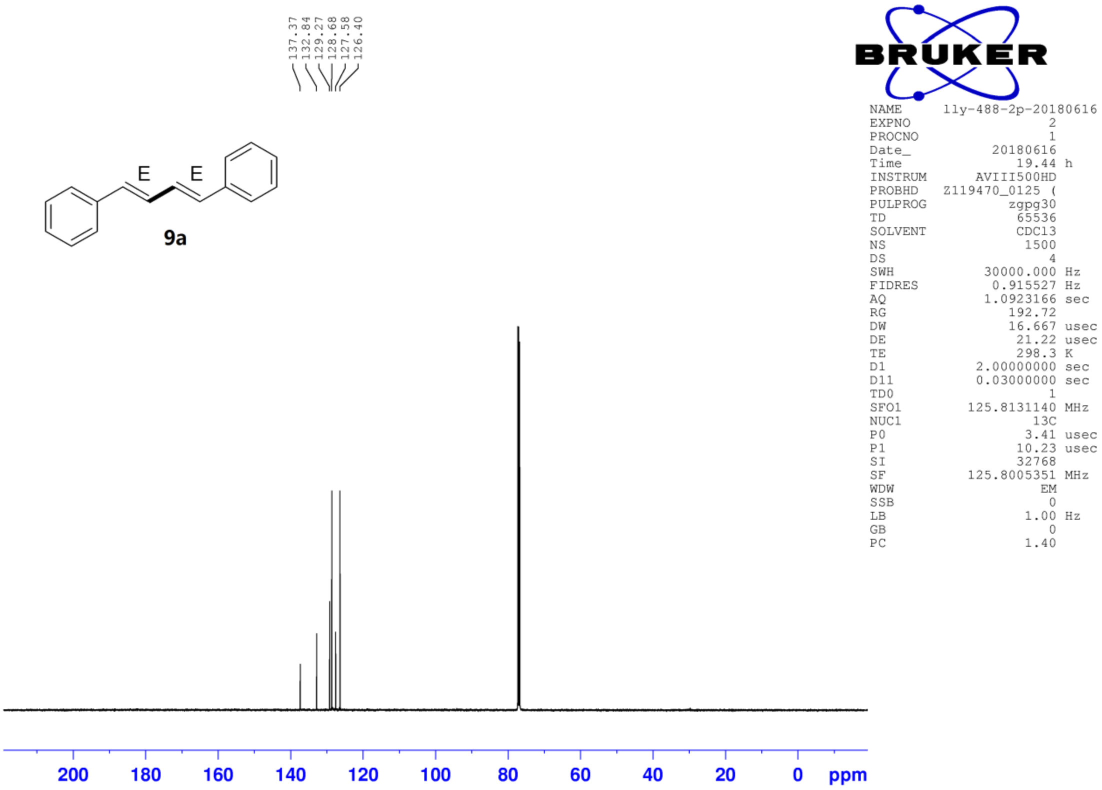

Supplementary Figure 104. ^{13}C NMR spectra for compound **9a**

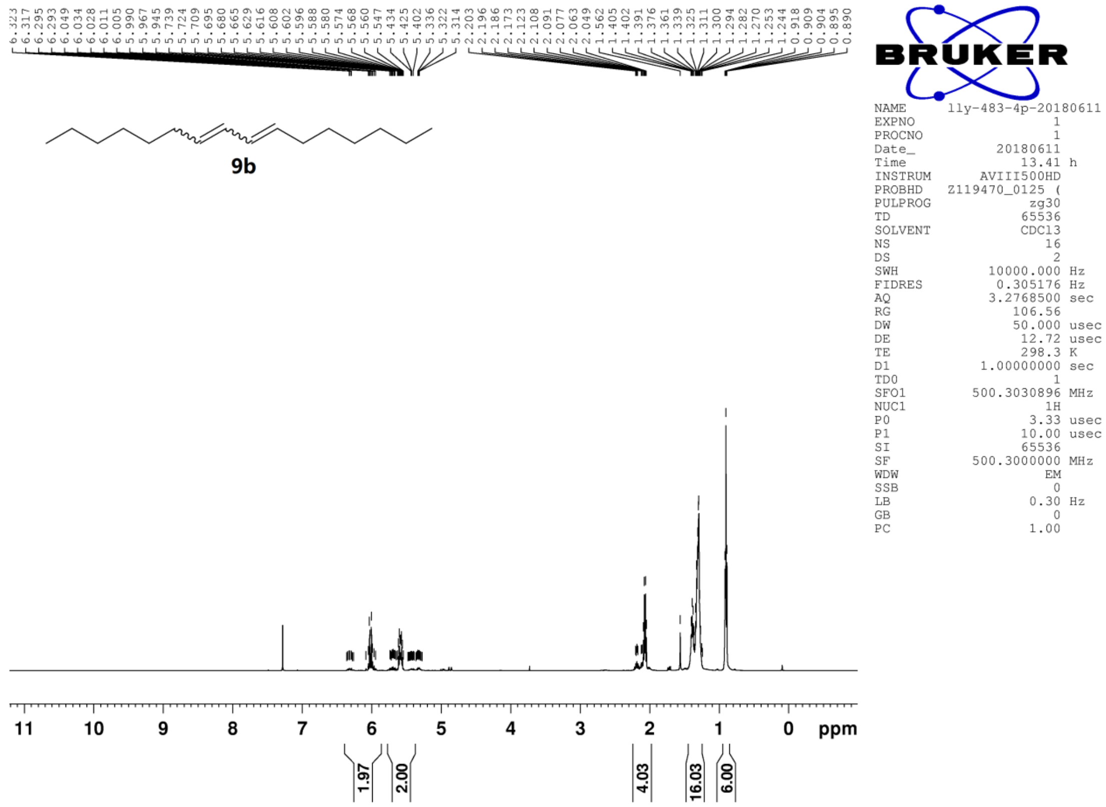

Supplementary Figure 105. ^{1}H NMR spectra for compound **9b**

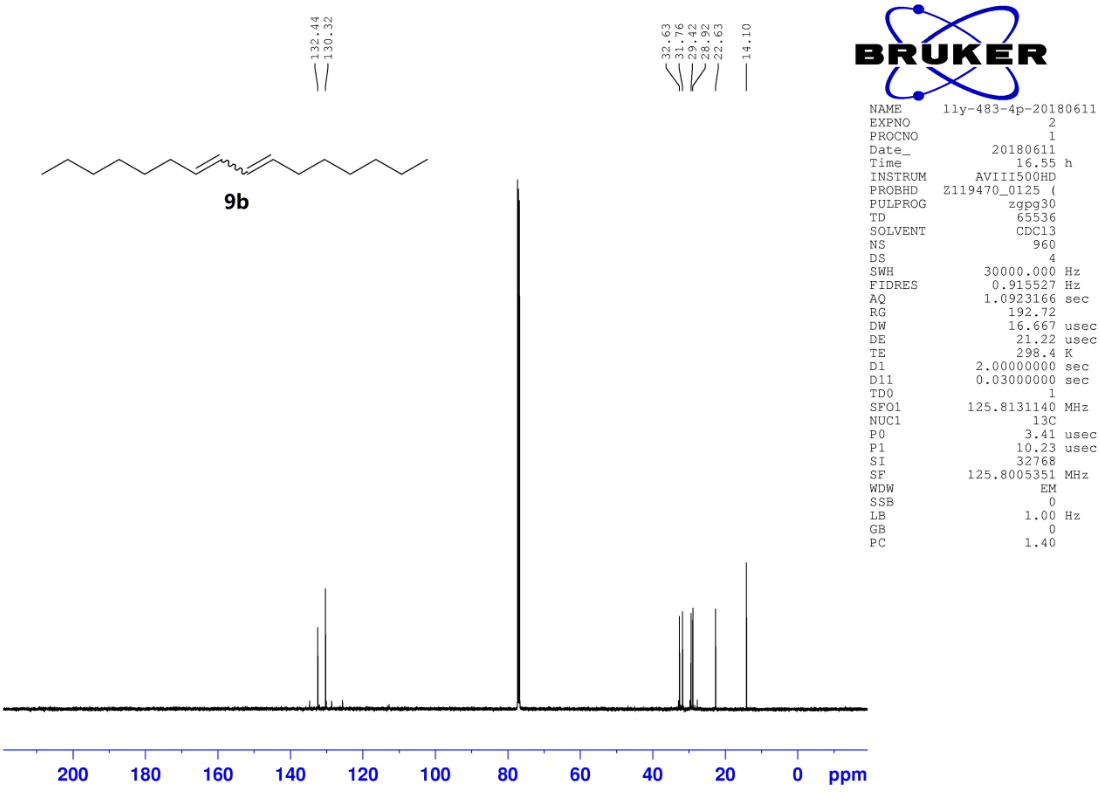

Supplementary Figure 106. ^{13}C NMR spectra for compound **9b**

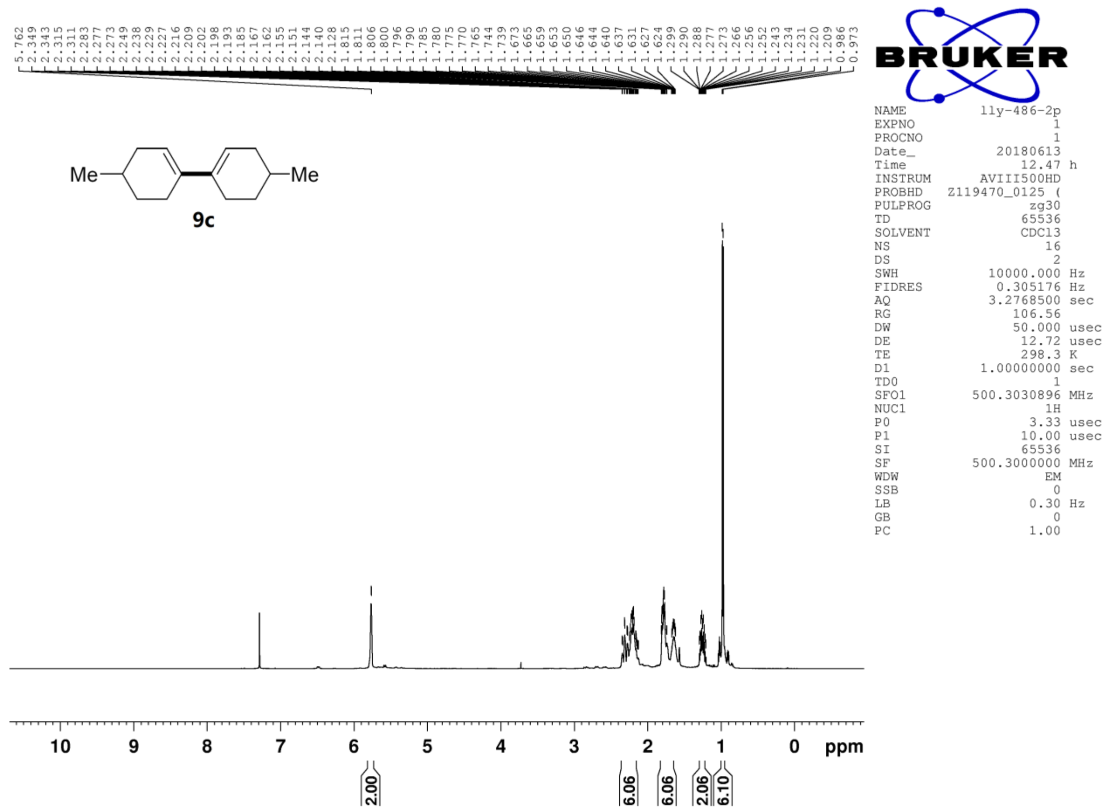

Supplementary Figure 107. ^{1}H NMR spectra for compound **9c**

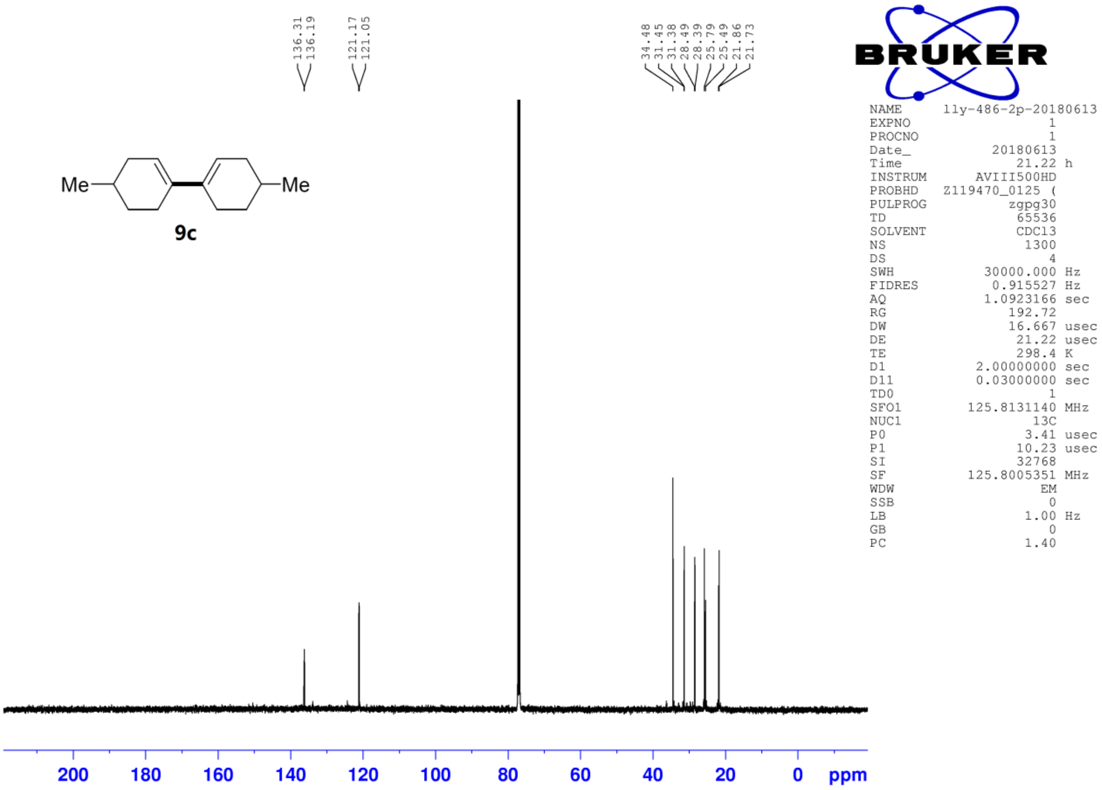

Supplementary Figure 108. ^{13}C NMR spectra for compound **9c**

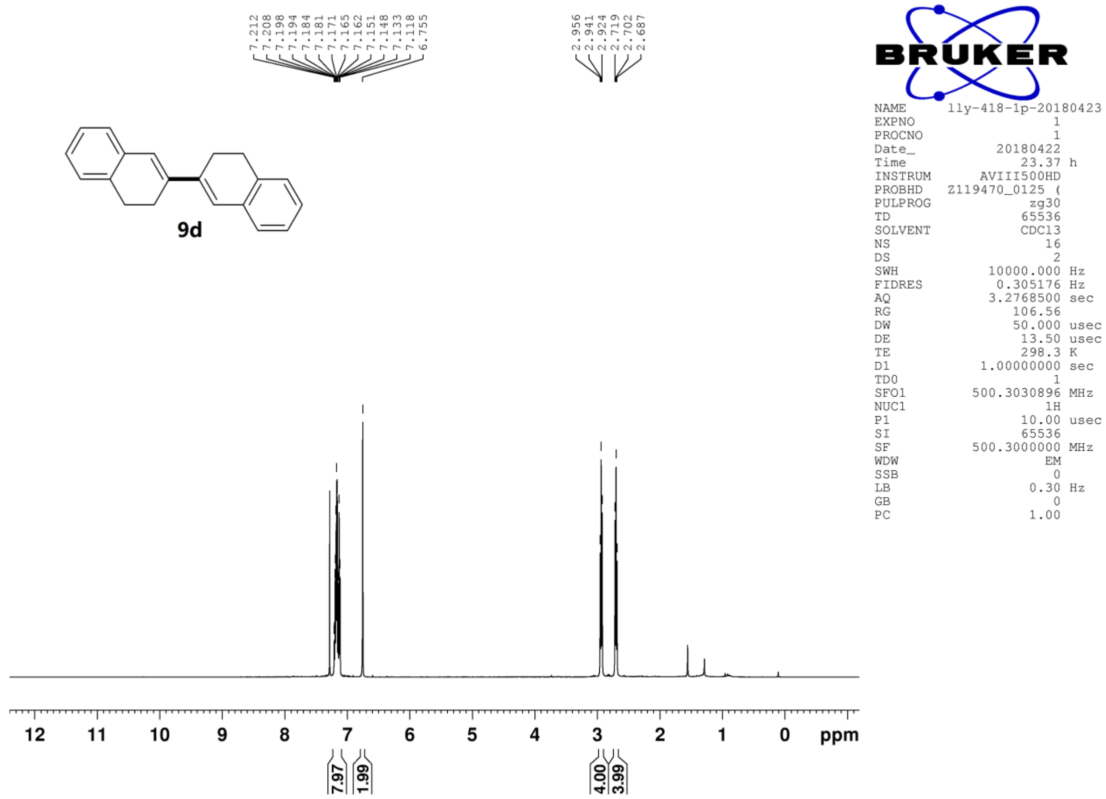

Supplementary Figure 109. ^{1}H NMR spectra for compound **9d**

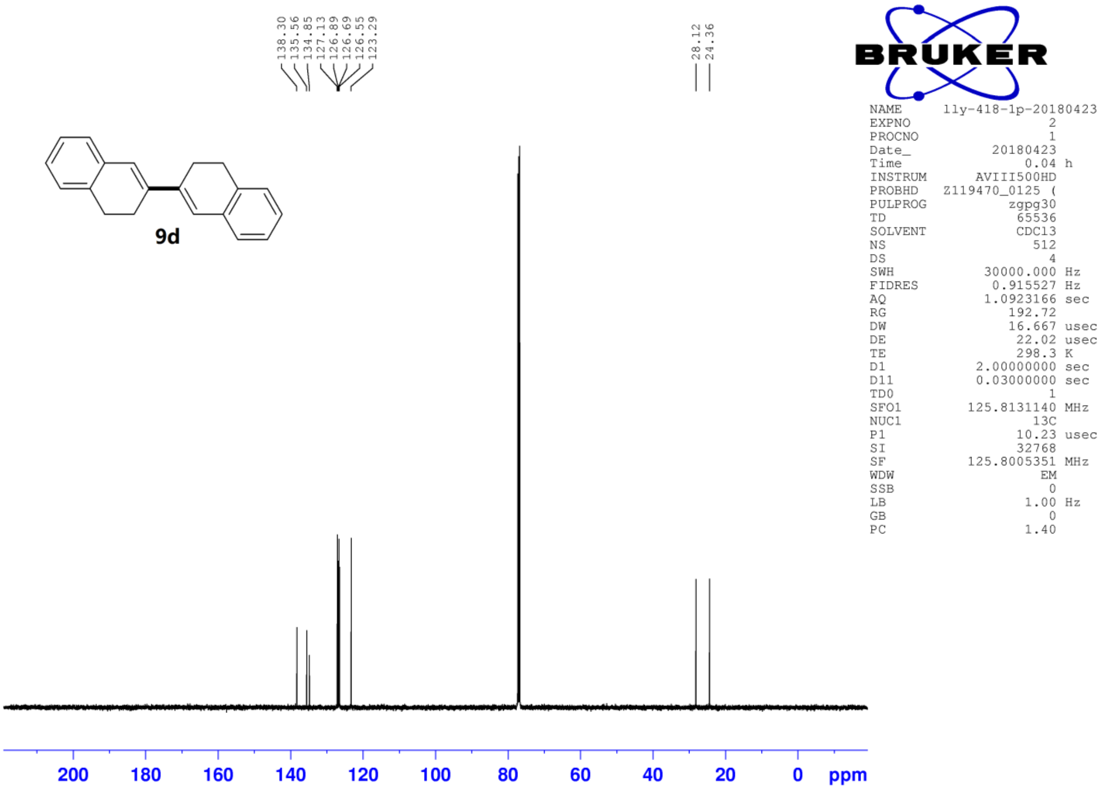

Supplementary Figure 110. ^{13}C NMR spectra for compound **9d**

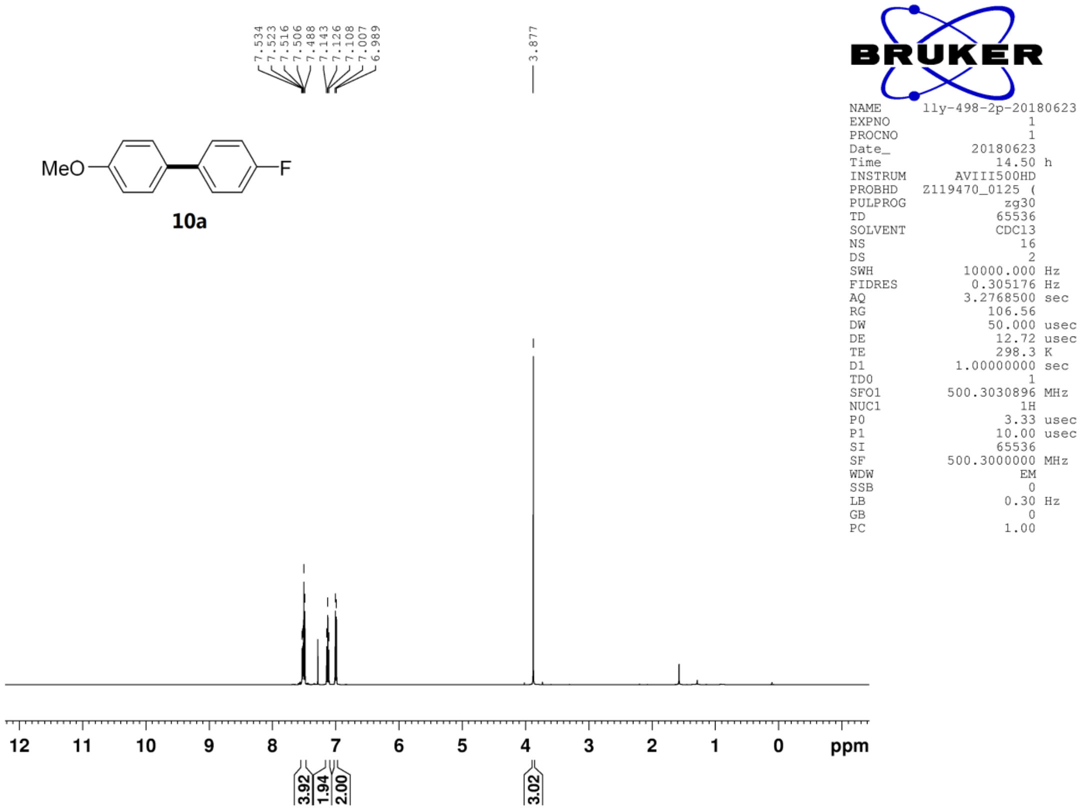

Supplementary Figure 111. ^{1}H NMR spectra for compound **10a**

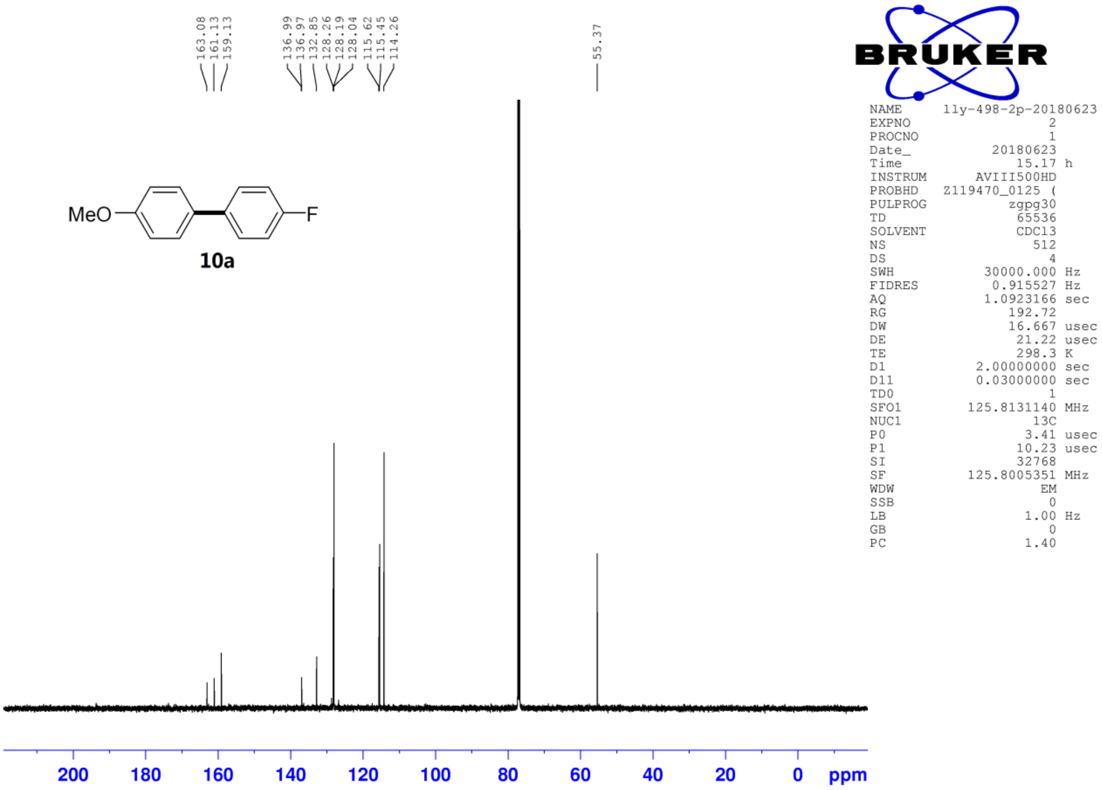

Supplementary Figure 112. ^{13}C NMR spectra for compound **10a**

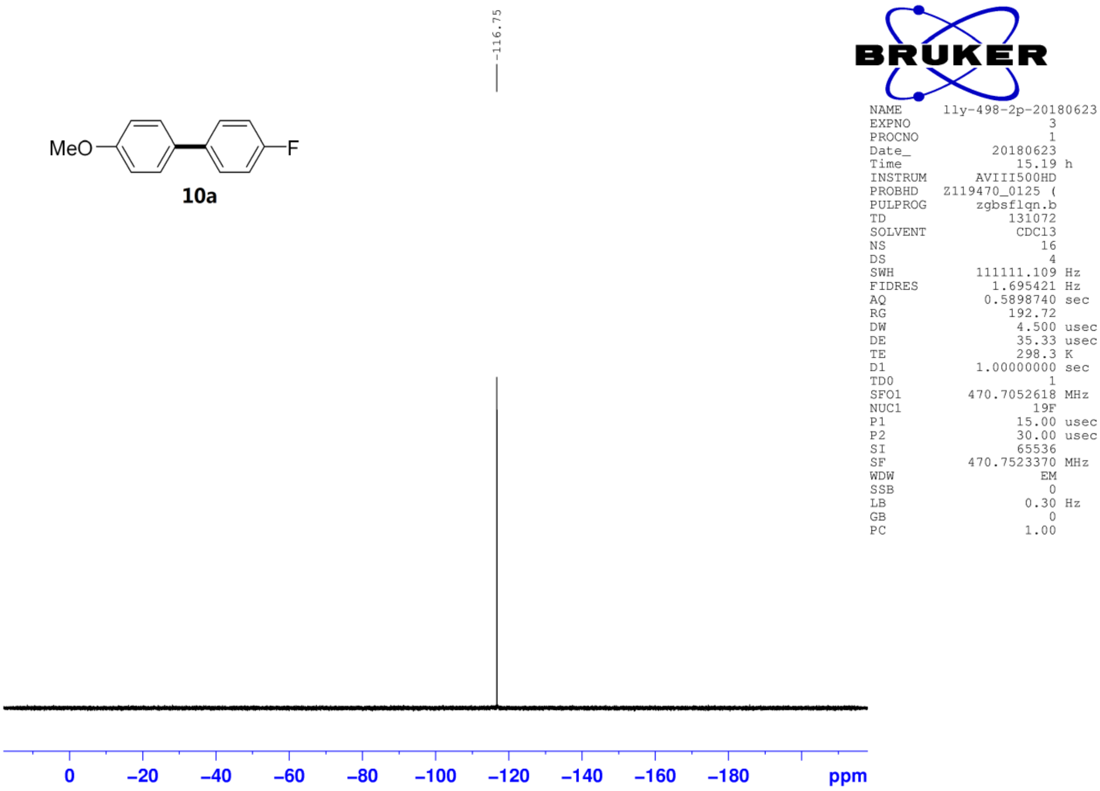


```
NAME       lly-498-2p-20180623
EXPNO                    3
PROCNO                   1
Date_             20180623
Time                 15.19 h
INSTRUM         AVIII500HD
PROBHD     Z119470_0125 (
PULPROG        zgbsflqn.b
TD                  131072
SOLVENT              CDCl3
NS                      16
DS                       4
SWH            111111.109 Hz
FIDRES            1.695421 Hz
AQ               0.5898740 sec
RG                  192.72
DW                   4.500 usec
DE                   35.33 usec
TE                   298.3 K
D1              1.00000000 sec
TD0                      1
SFO1           470.7052618 MHz
NUC1                   19F
P1                   15.00 usec
P2                   30.00 usec
SI                   65536
SF             470.7523370 MHz
WDW                     EM
SSB                      0
LB                    0.30 Hz
GB                       0
PC                    1.00
```

Supplementary Figure 113. ^{19}F NMR spectra for compound **10a**

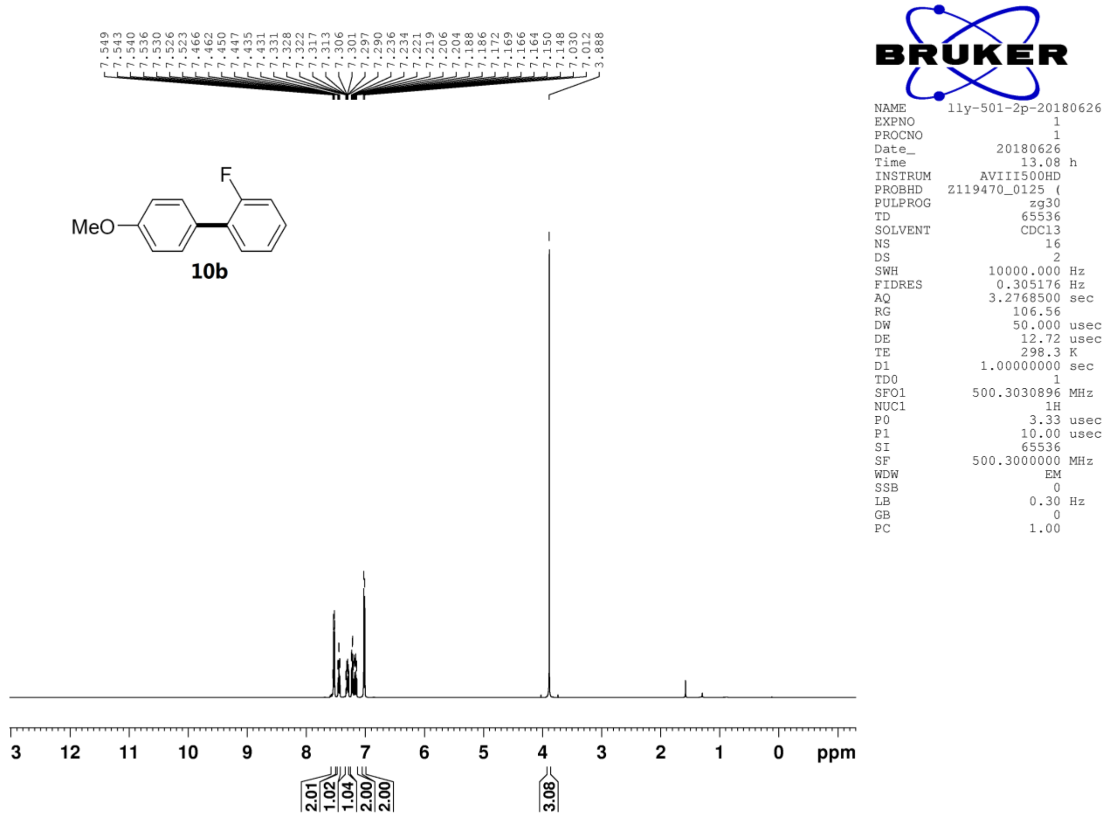

Supplementary Figure 114. ^{1}H NMR spectra for compound **10b**

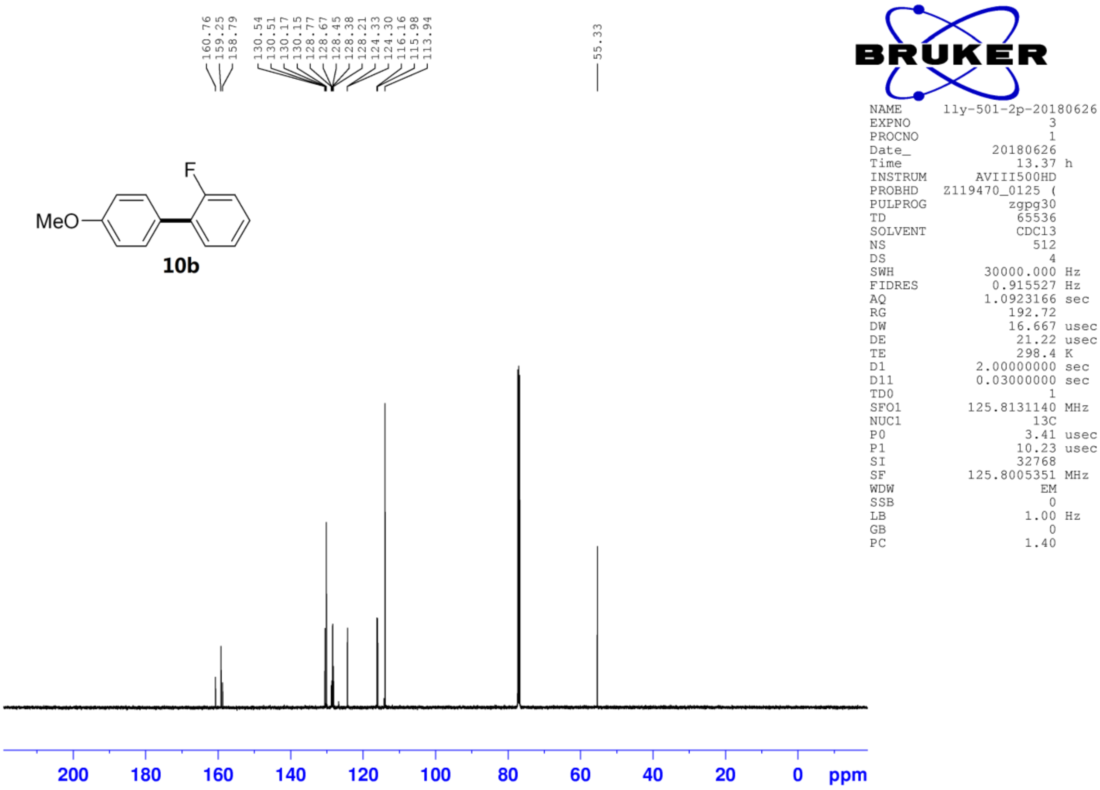

Supplementary Figure 115. ^{13}C NMR spectra for compound **10b**

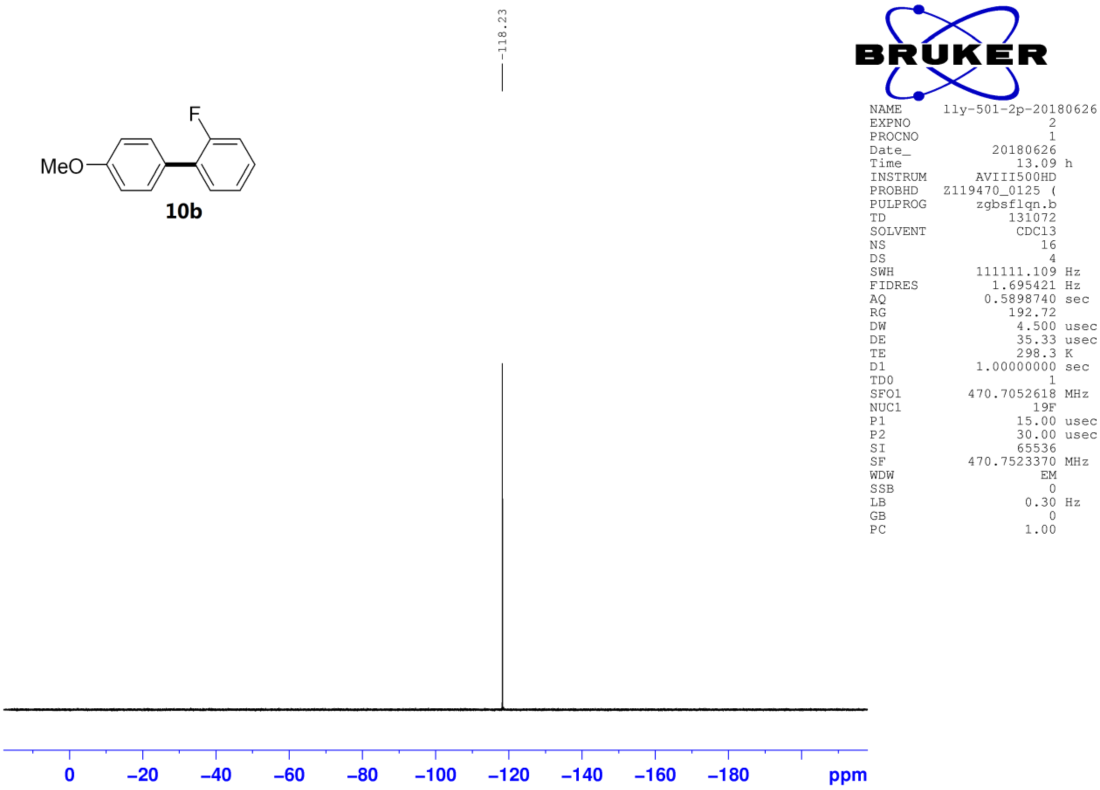

Supplementary Figure 116. ^{19}F NMR spectra for compound **10b**

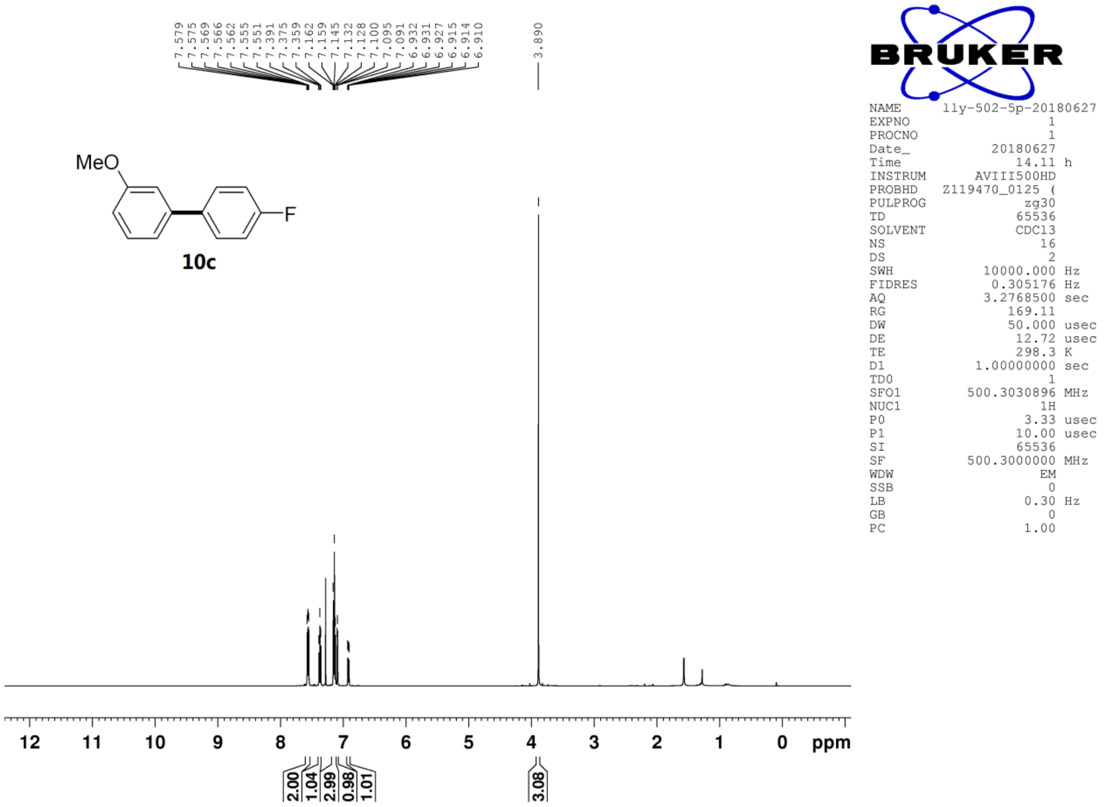

Supplementary Figure 117. ^{1}H NMR spectra for compound **10c**

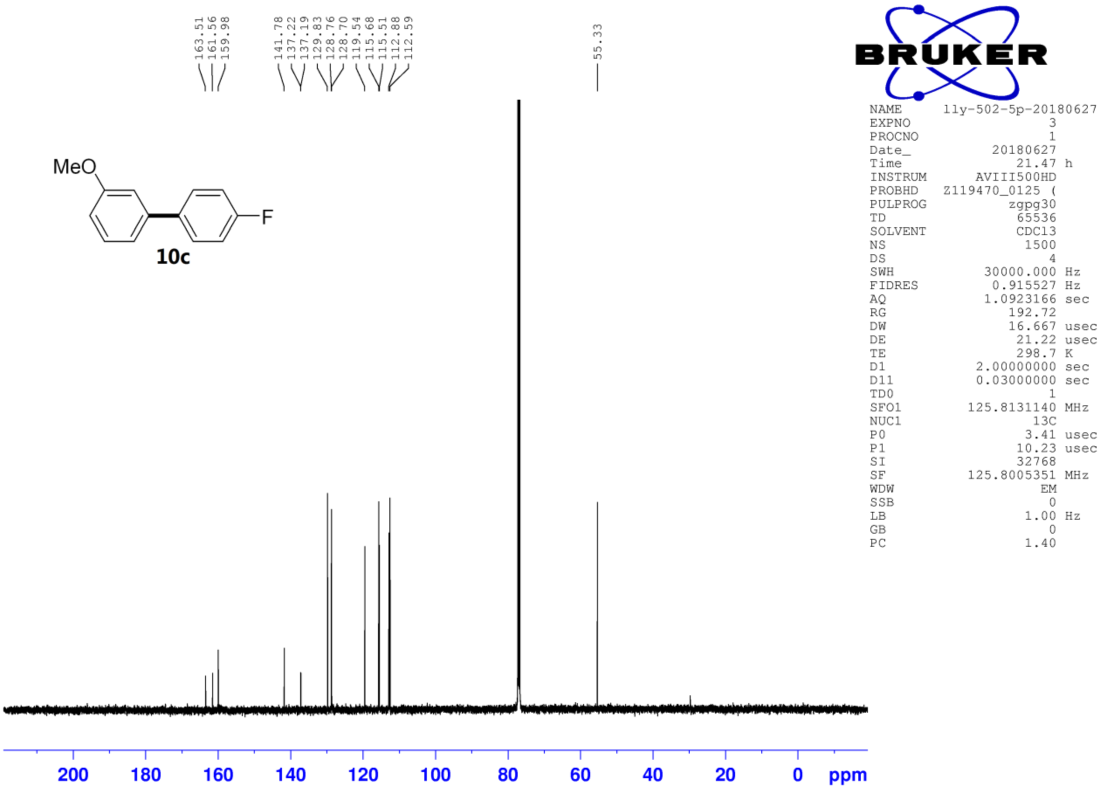

Supplementary Figure 118. ^{13}C NMR spectra for compound **10c**

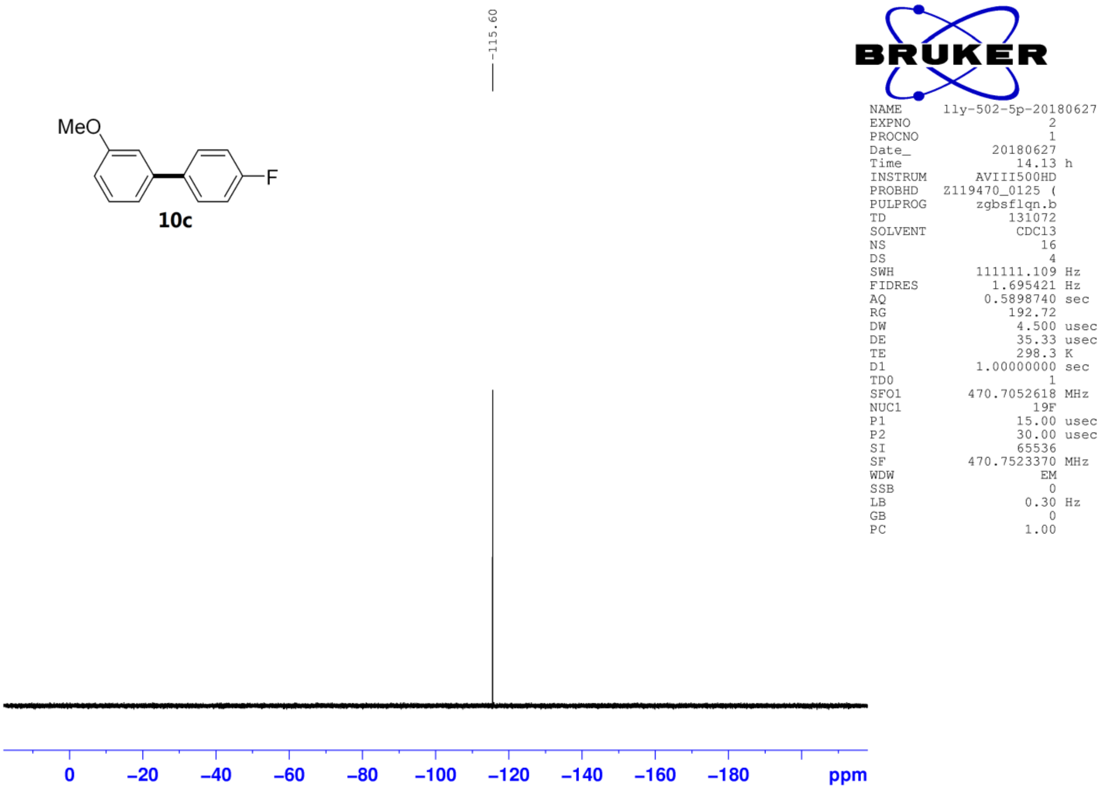

Supplementary Figure 119. ^{19}F NMR spectra for compound **10c**

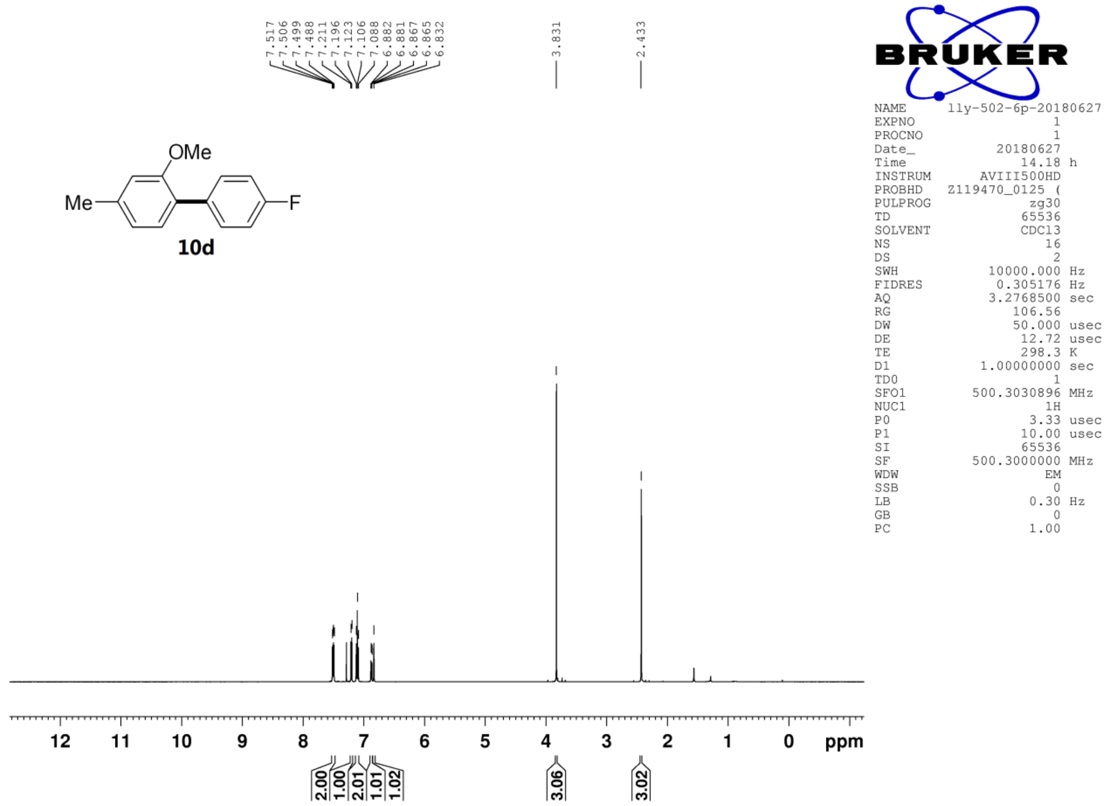

Supplementary Figure 120. ^{1}H NMR spectra for compound **10d**

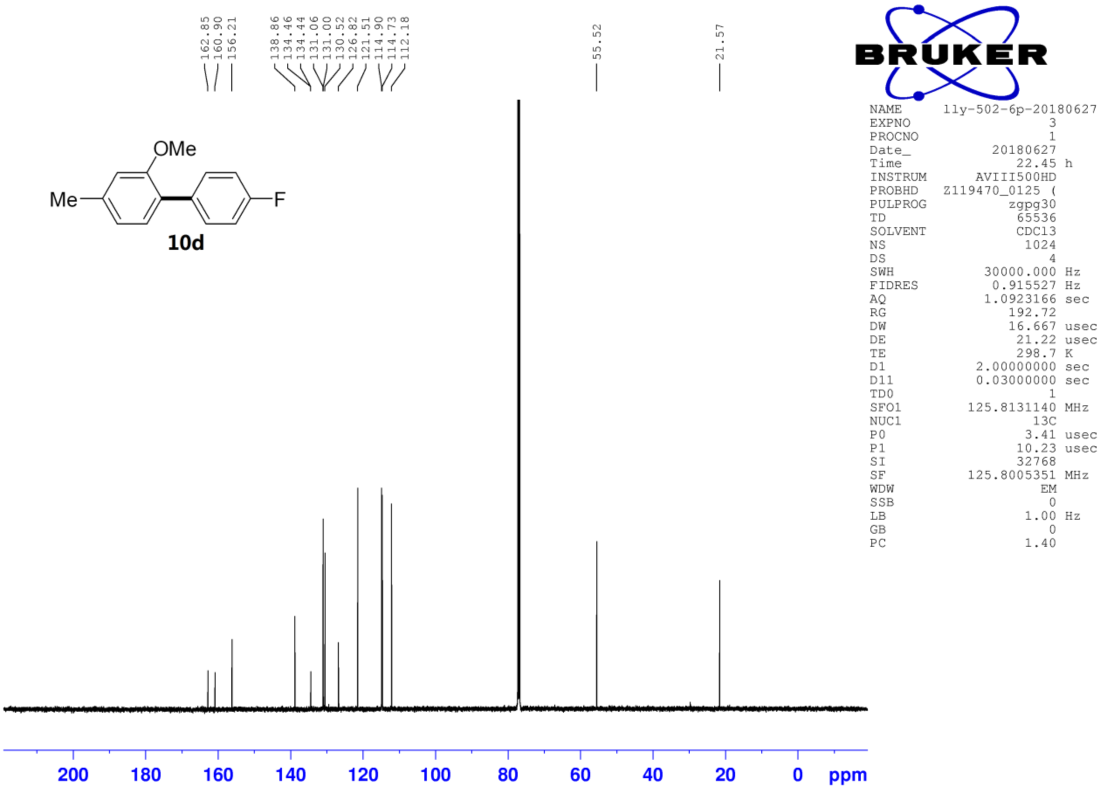

Supplementary Figure 121. ^{13}C NMR spectra for compound **10d**

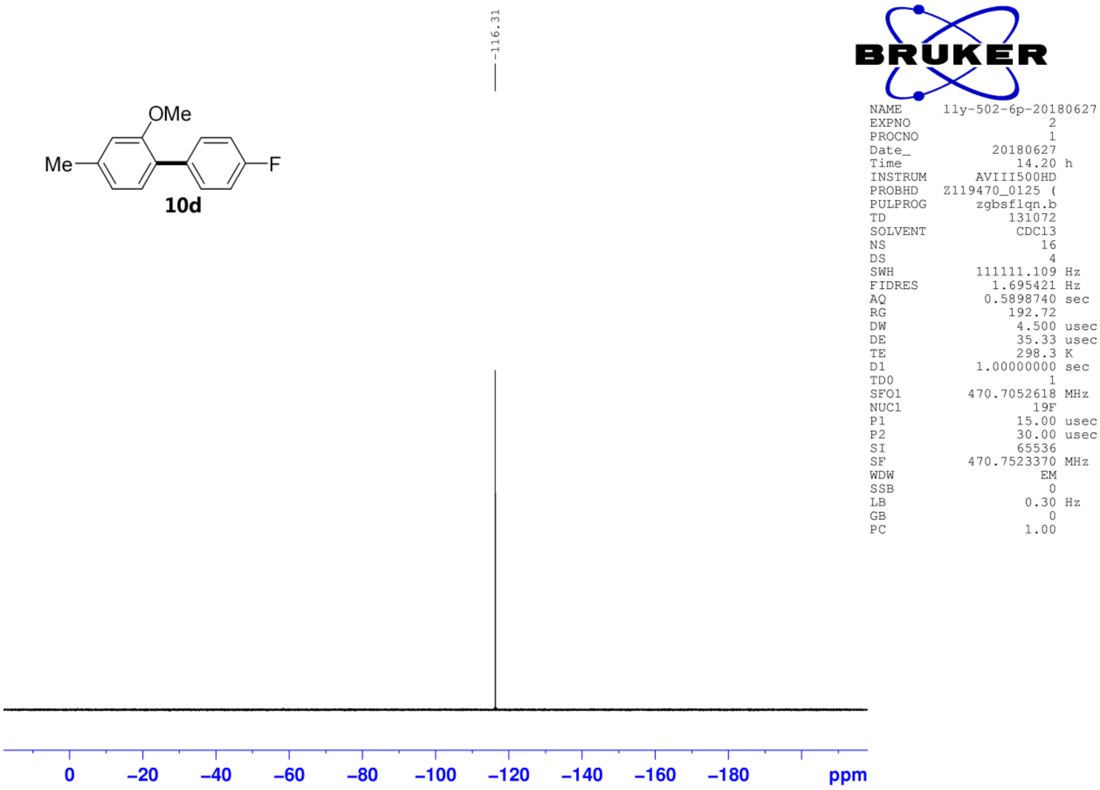

Supplementary Figure 122. ^{19}F NMR spectra for compound **10d**

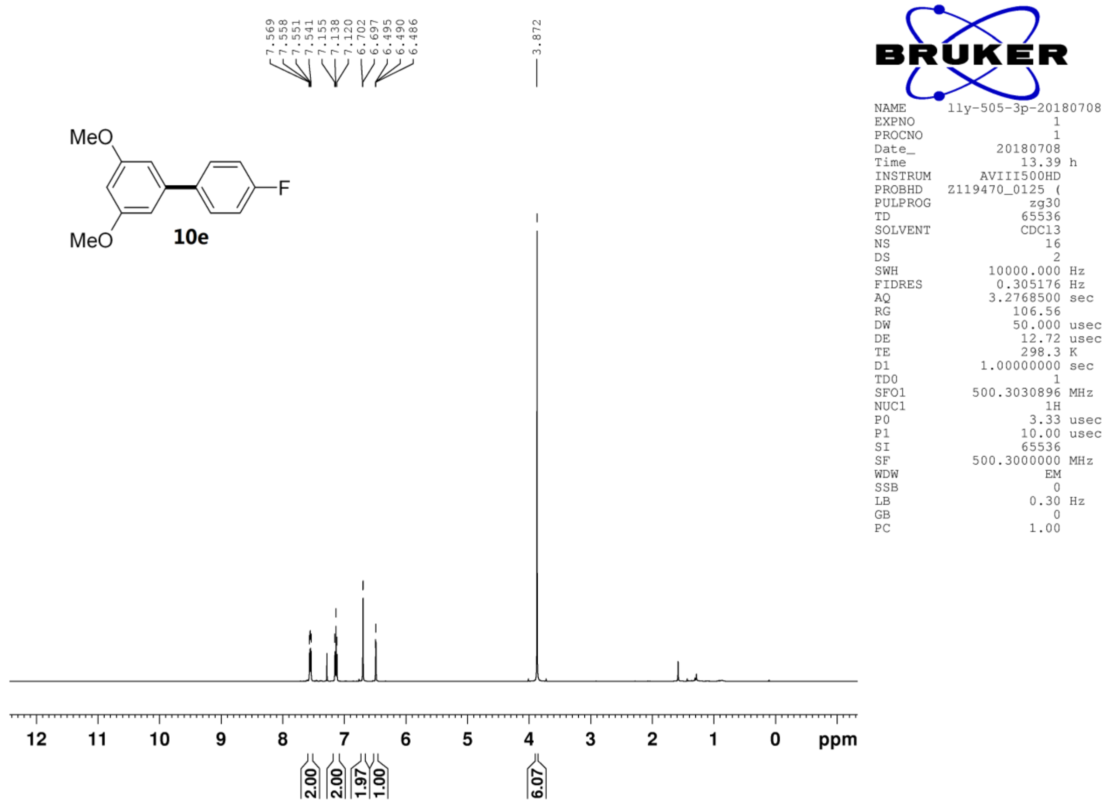

Supplementary Figure 123. ^{1}H NMR spectra for compound **10e**

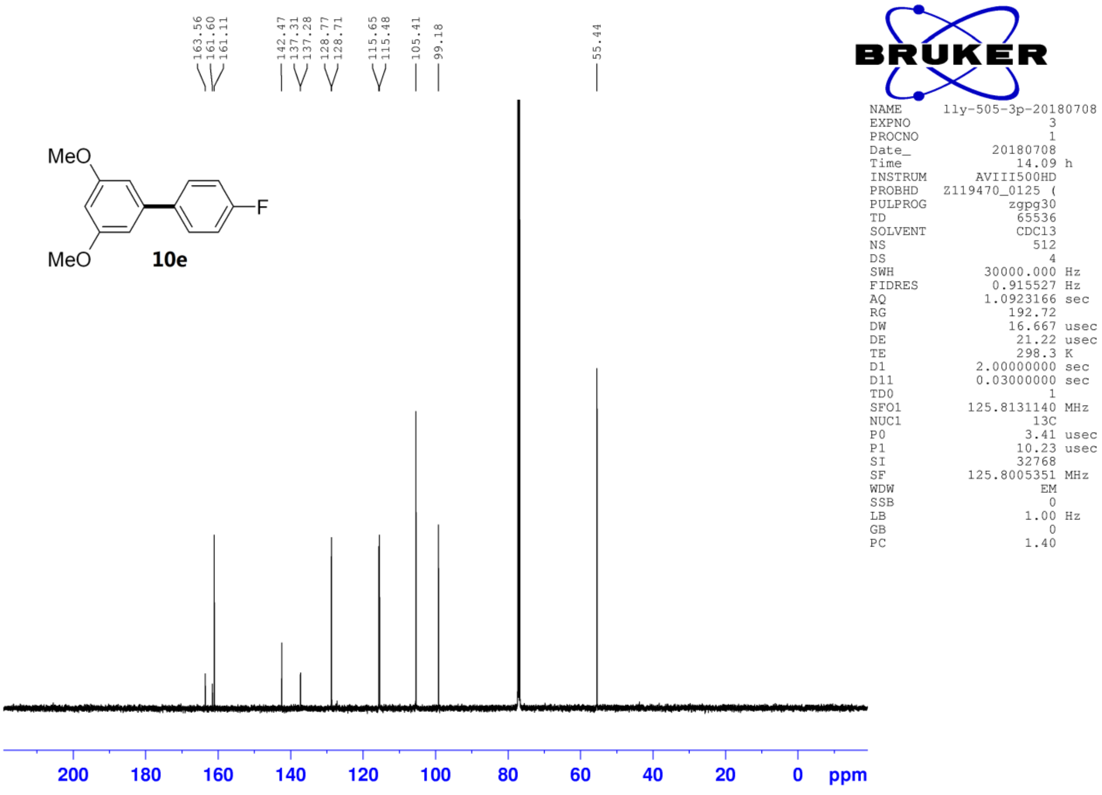

Supplementary Figure 124. ^{13}C NMR spectra for compound **10e**

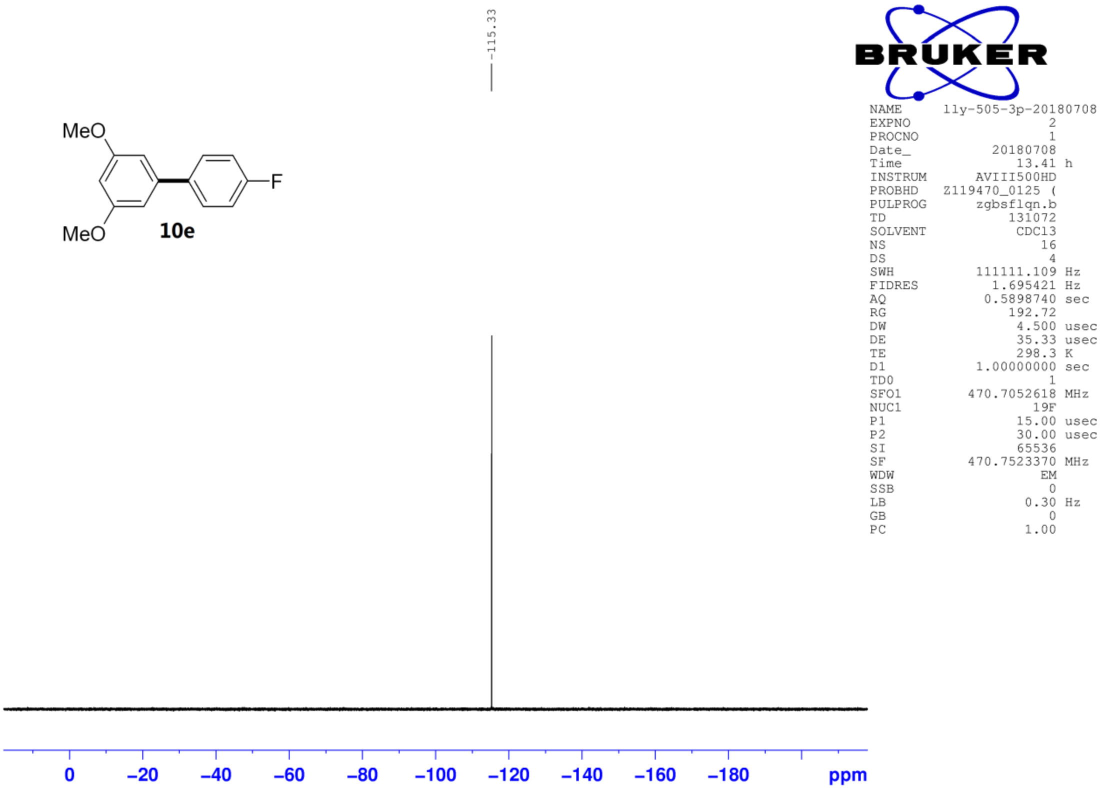

Supplementary Figure 125. ^{19}F NMR spectra for compound **10e**

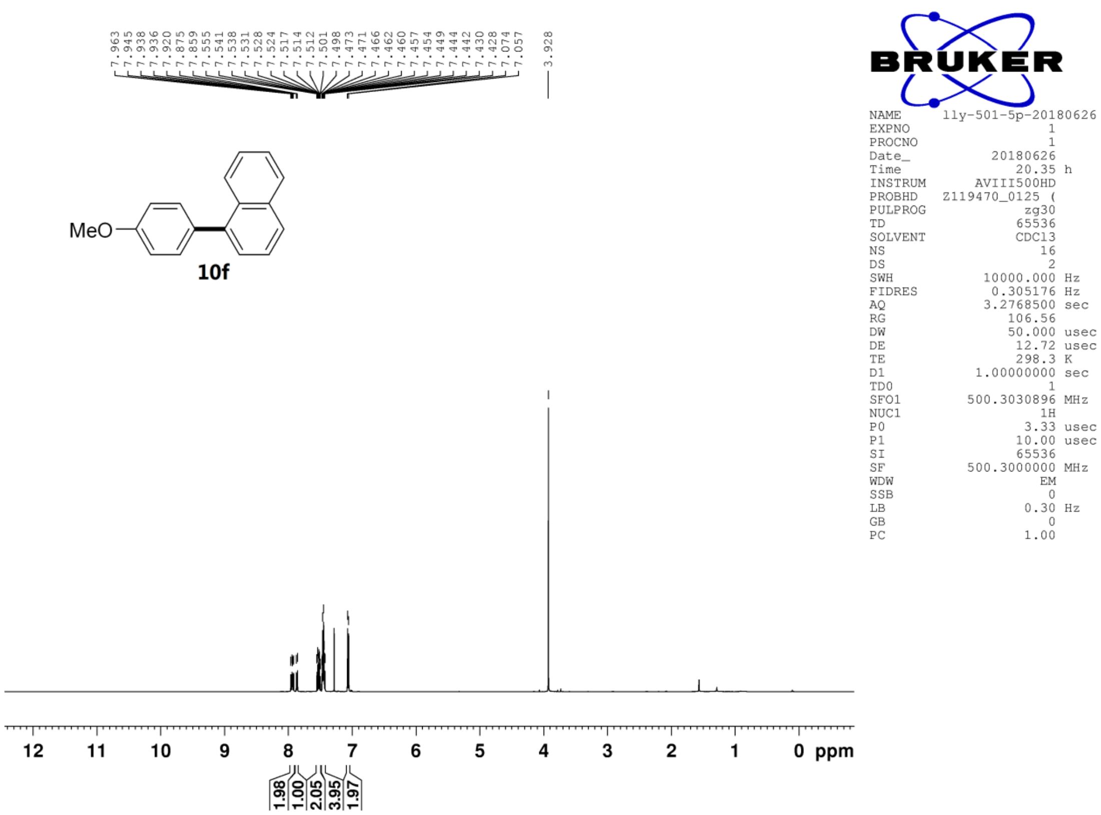

Supplementary Figure 126. ^{1}H NMR spectra for compound **10f**

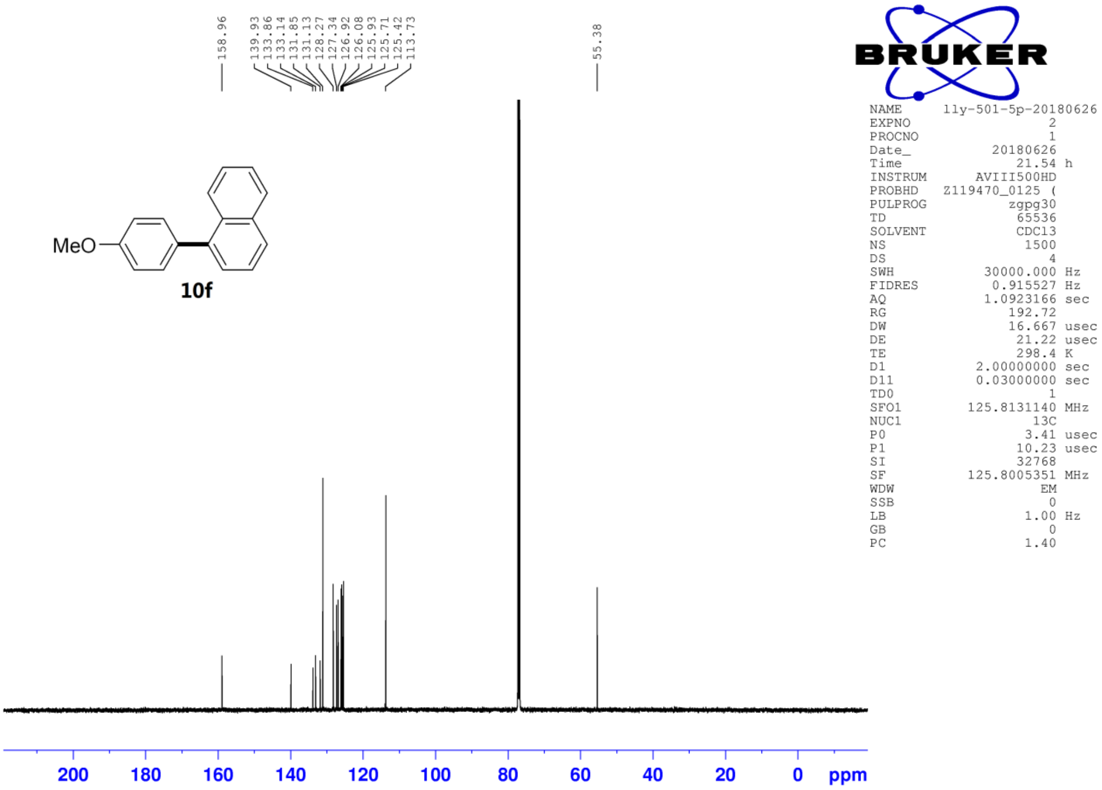

Supplementary Figure 127. ^{13}C NMR spectra for compound **10f**

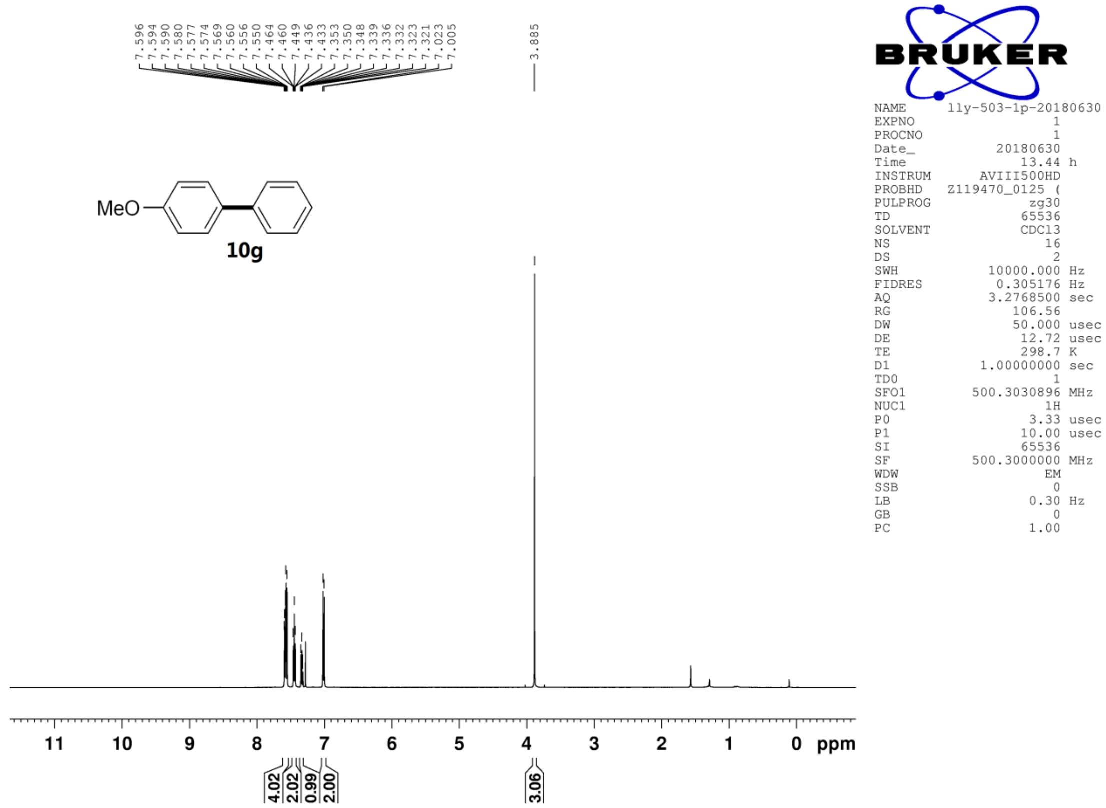

Supplementary Figure 128. ^{1}H NMR spectra for compound **10g**

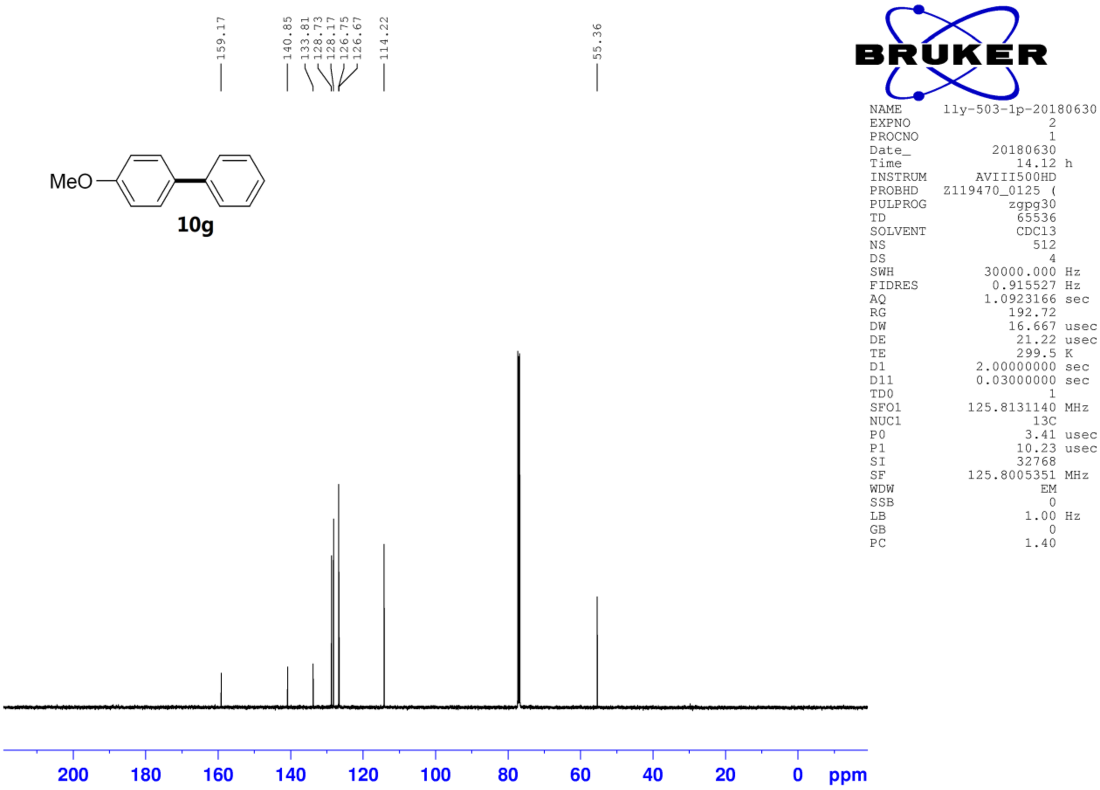

Supplementary Figure 129. ^{13}C NMR spectra for compound **10g**

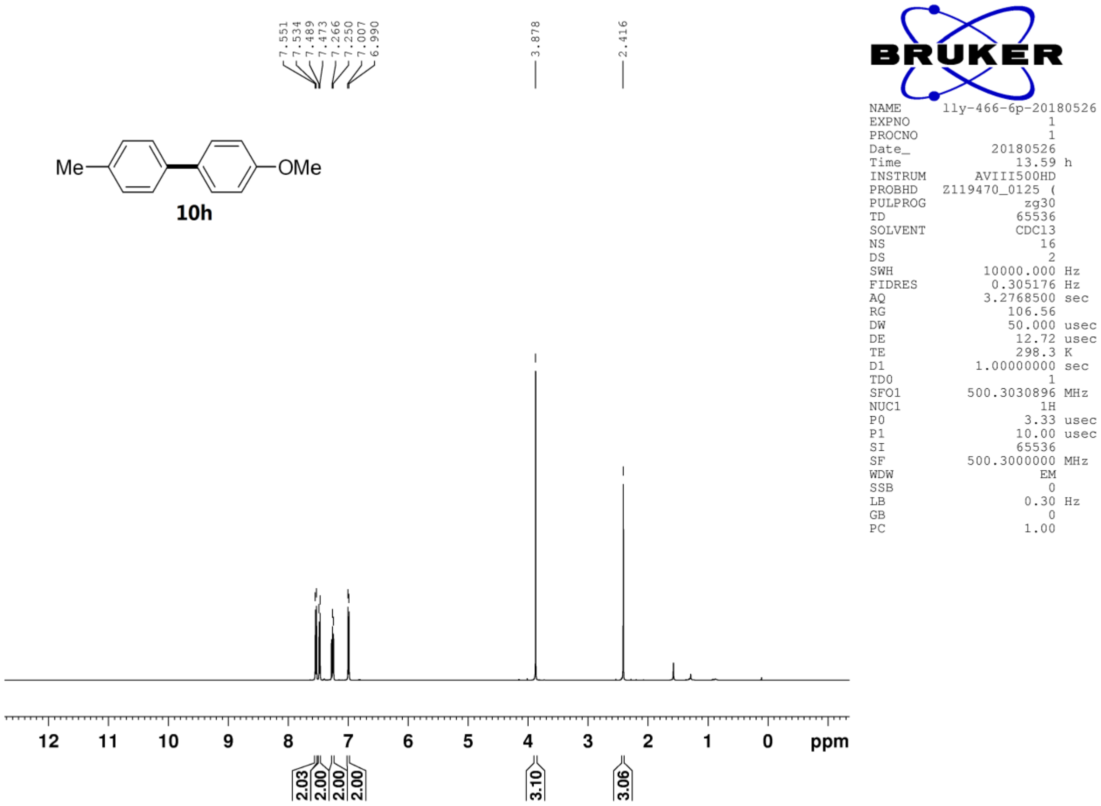

Supplementary Figure 130. ^{1}H NMR spectra for compound **10h**

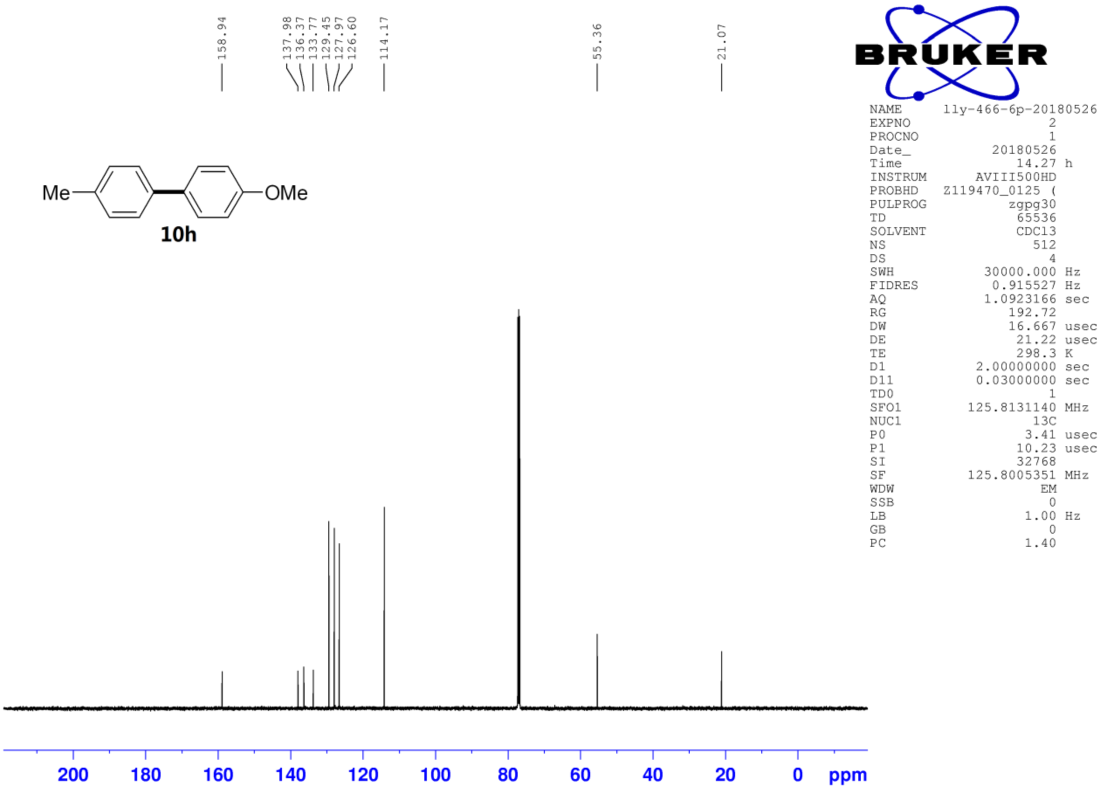

Supplementary Figure 131. ^{13}C NMR spectra for compound **10h**

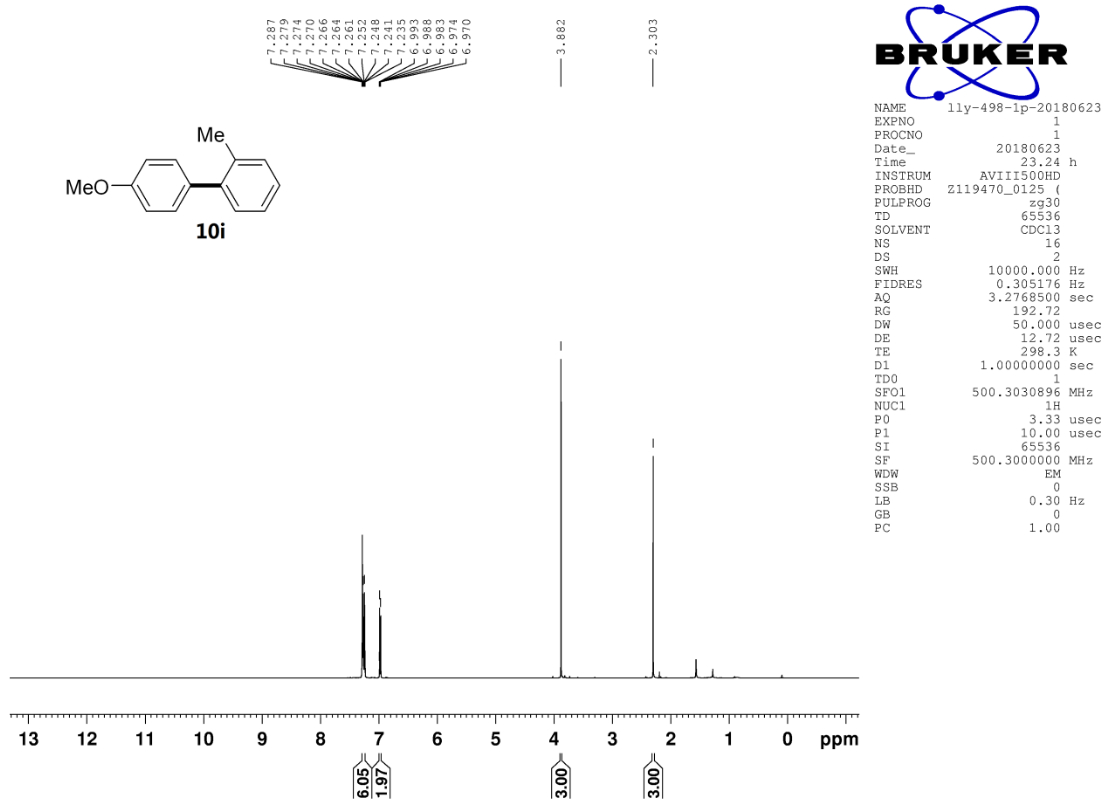

Supplementary Figure 132. ^{1}H NMR spectra for compound **10i**

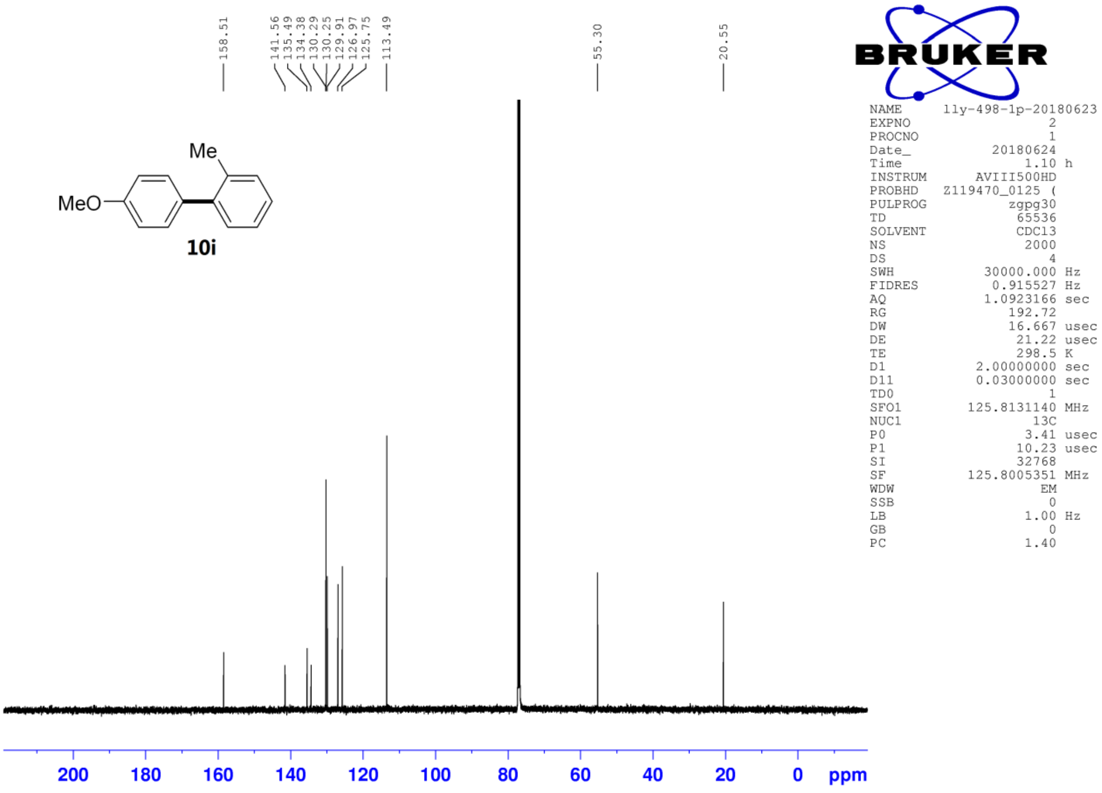

Supplementary Figure 133. ^{13}C NMR spectra for compound **10i**

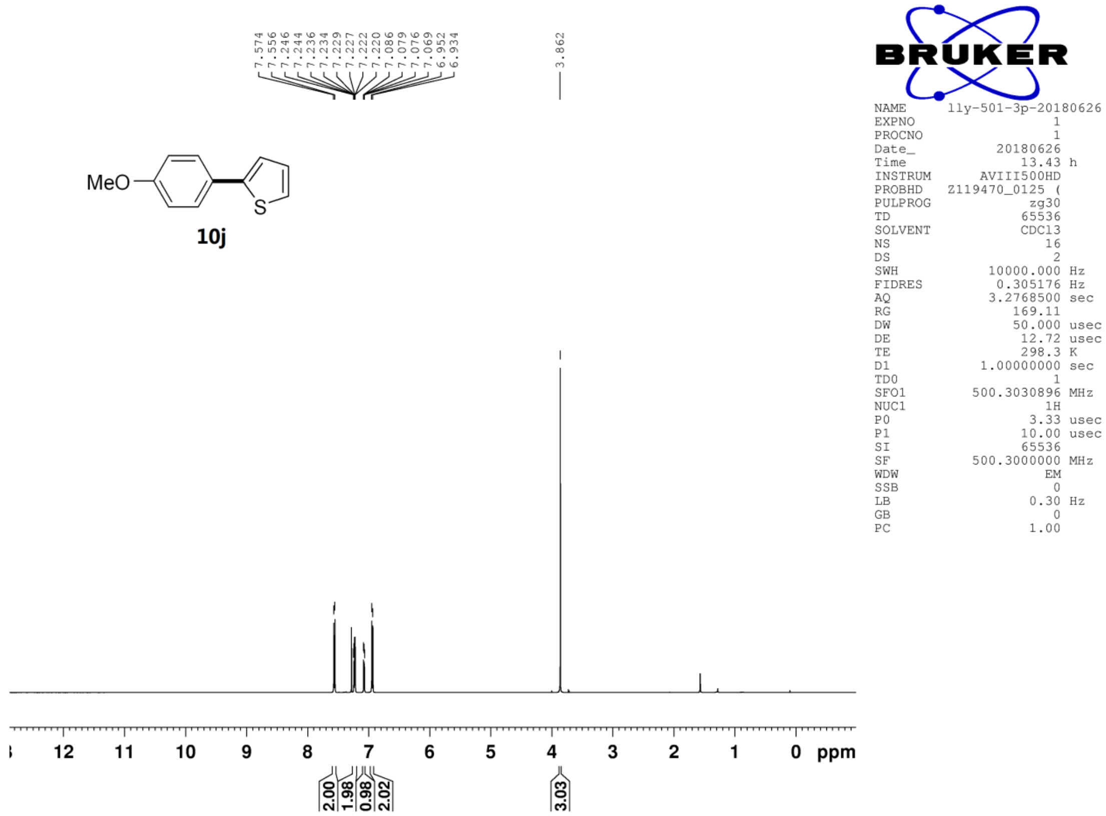

Supplementary Figure 134. ^{1}H NMR spectra for compound **10j**

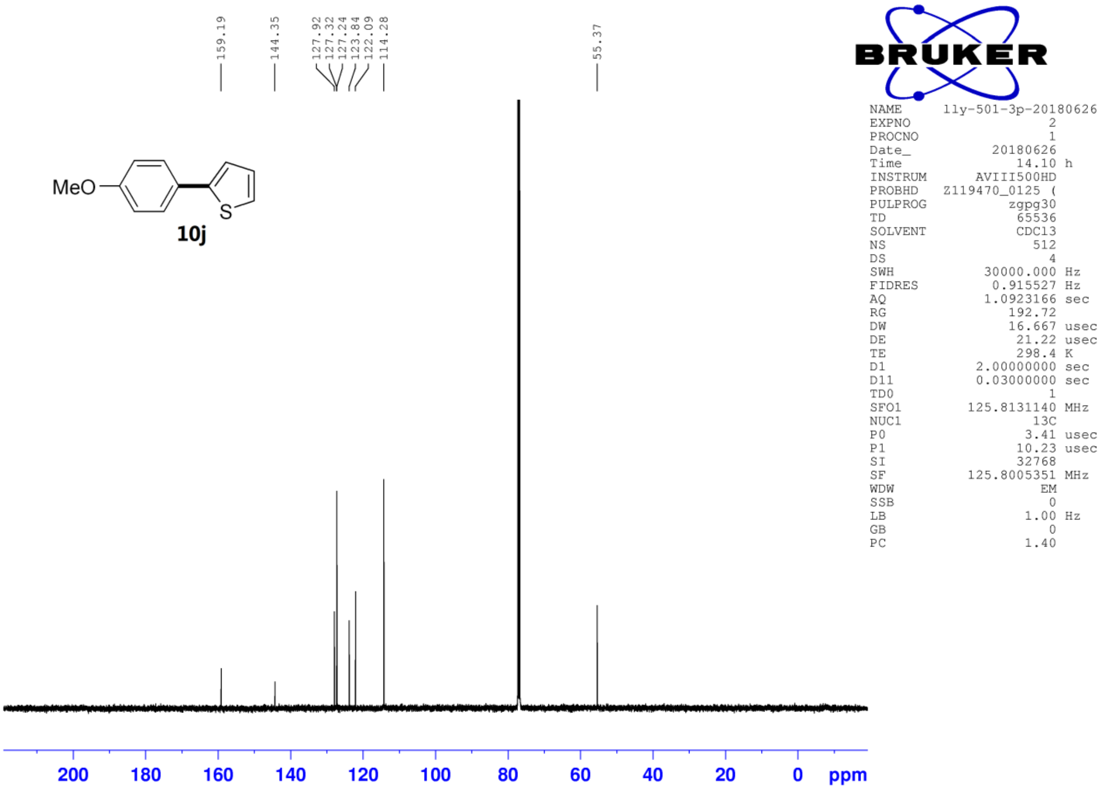

Supplementary Figure 135. ^{13}C NMR spectra for compound **10j**

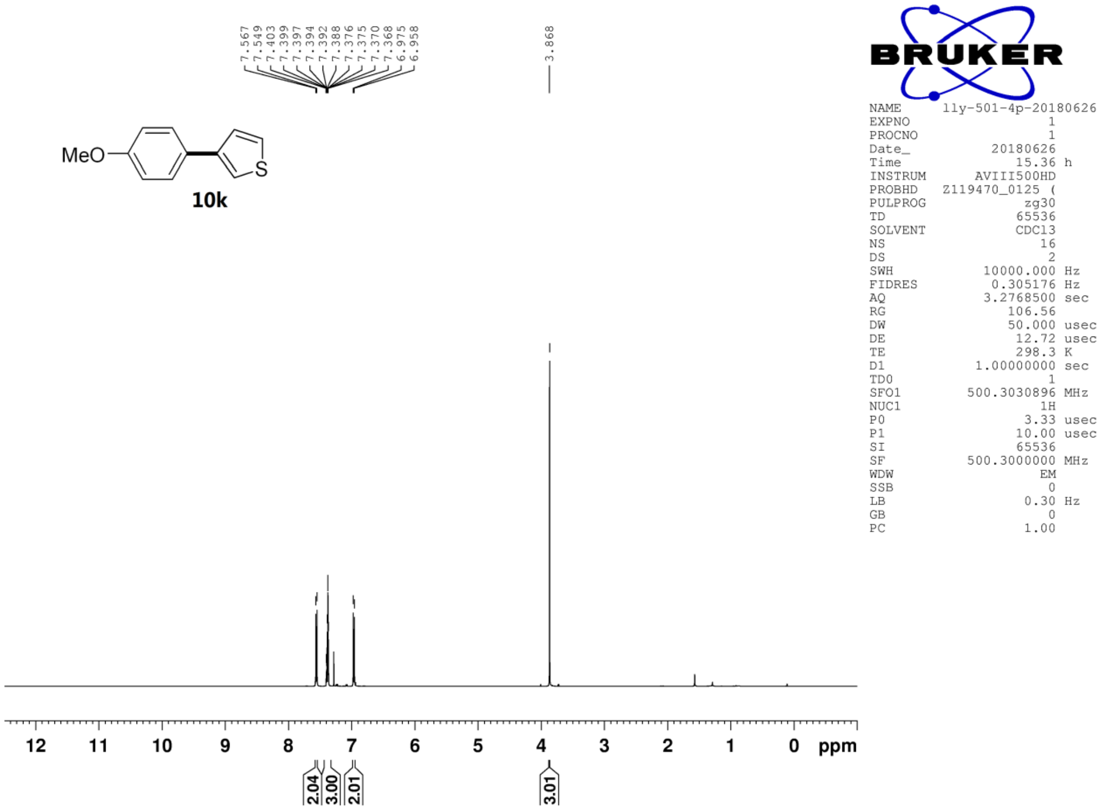

Supplementary Figure 136. ^{1}H NMR spectra for compound **10k**

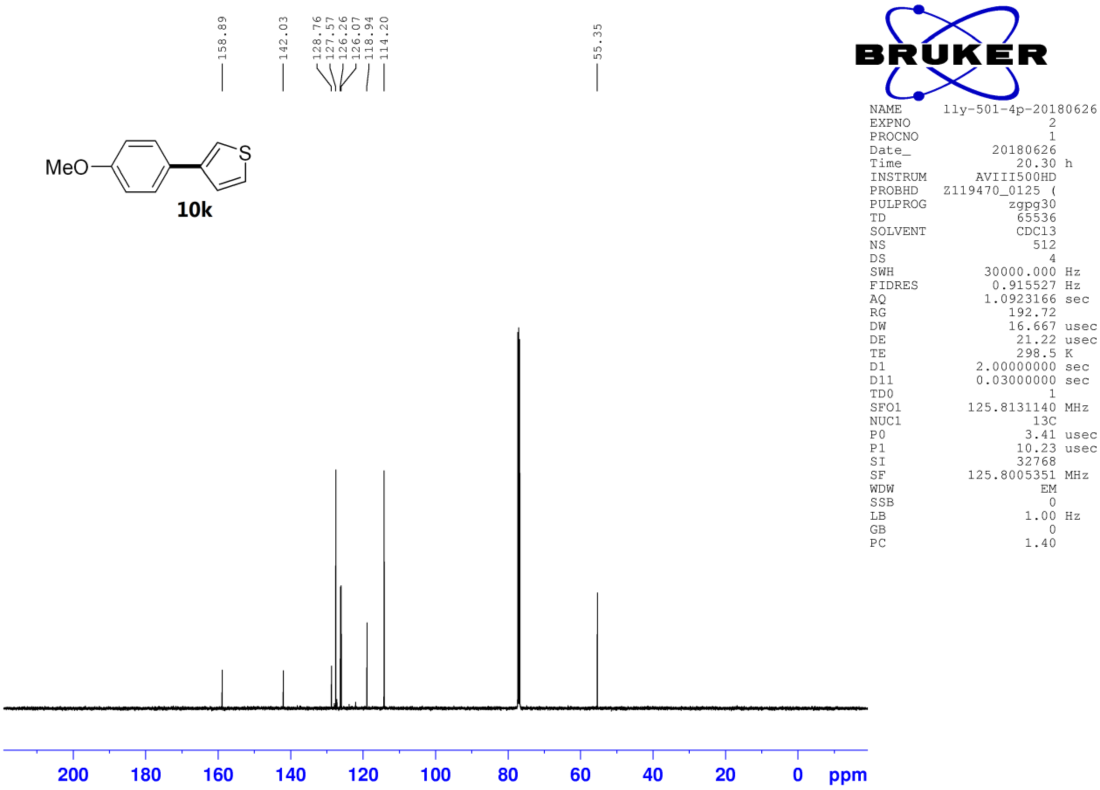

Supplementary Figure 137. ^{13}C NMR spectra for compound **10k**

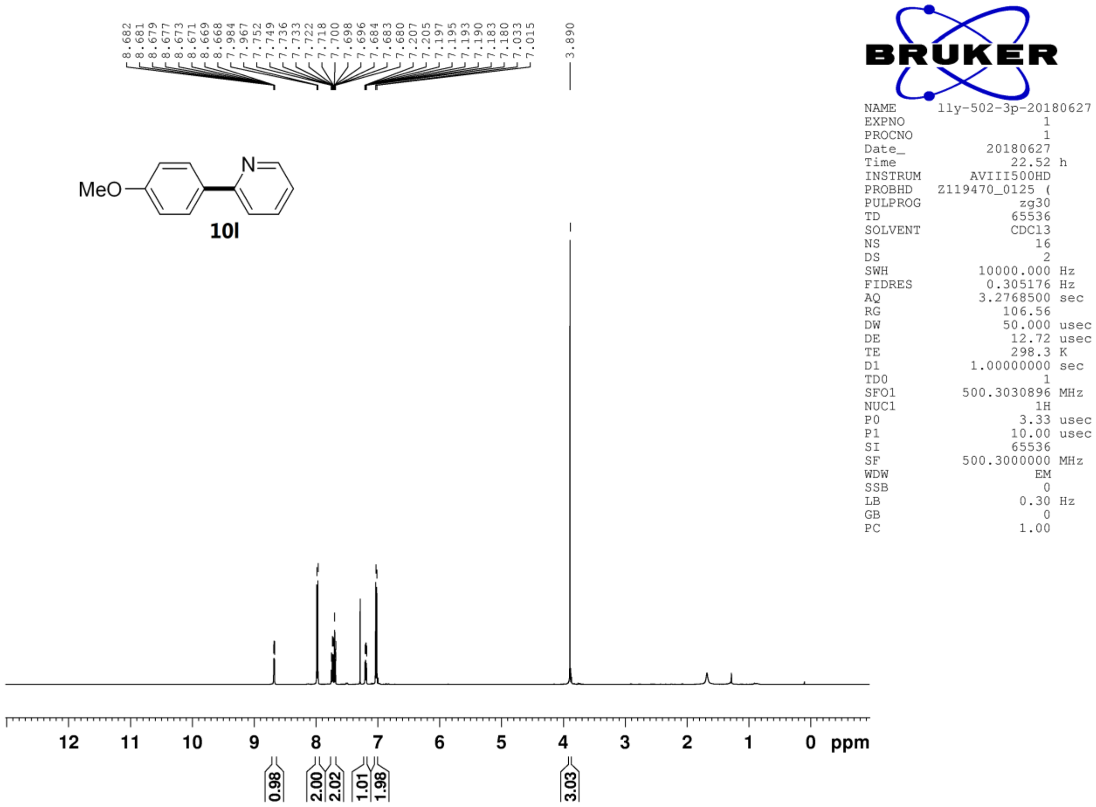

Supplementary Figure 138. ^{1}H NMR spectra for compound **10l**

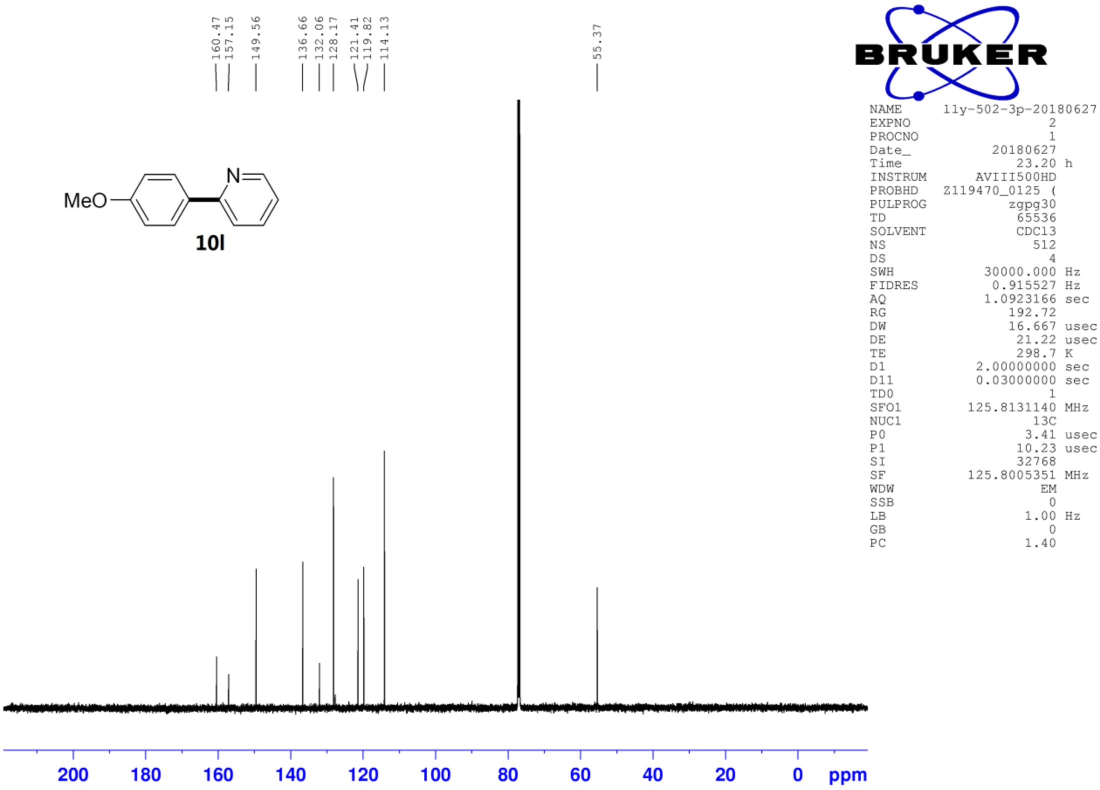

Supplementary Figure 139. ^{13}C NMR spectra for compound **10l**

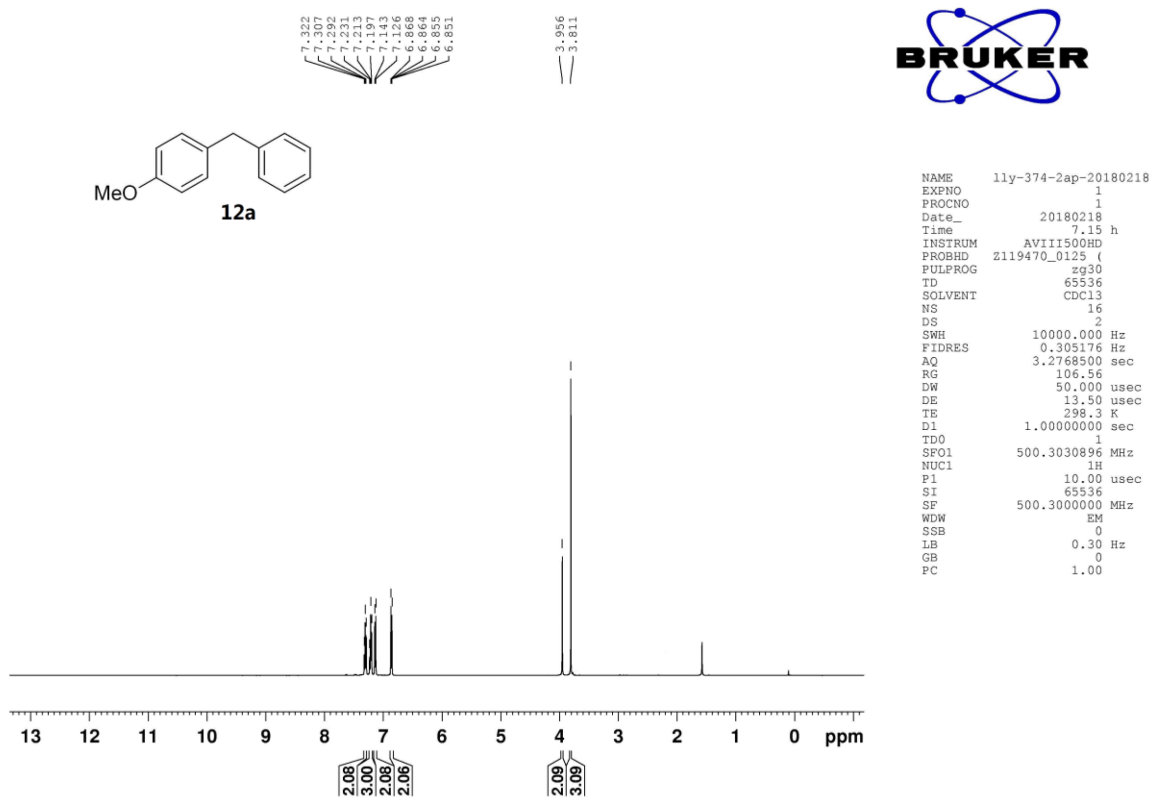

Supplementary Figure 140. ^{1}H NMR spectra for compound **12a**

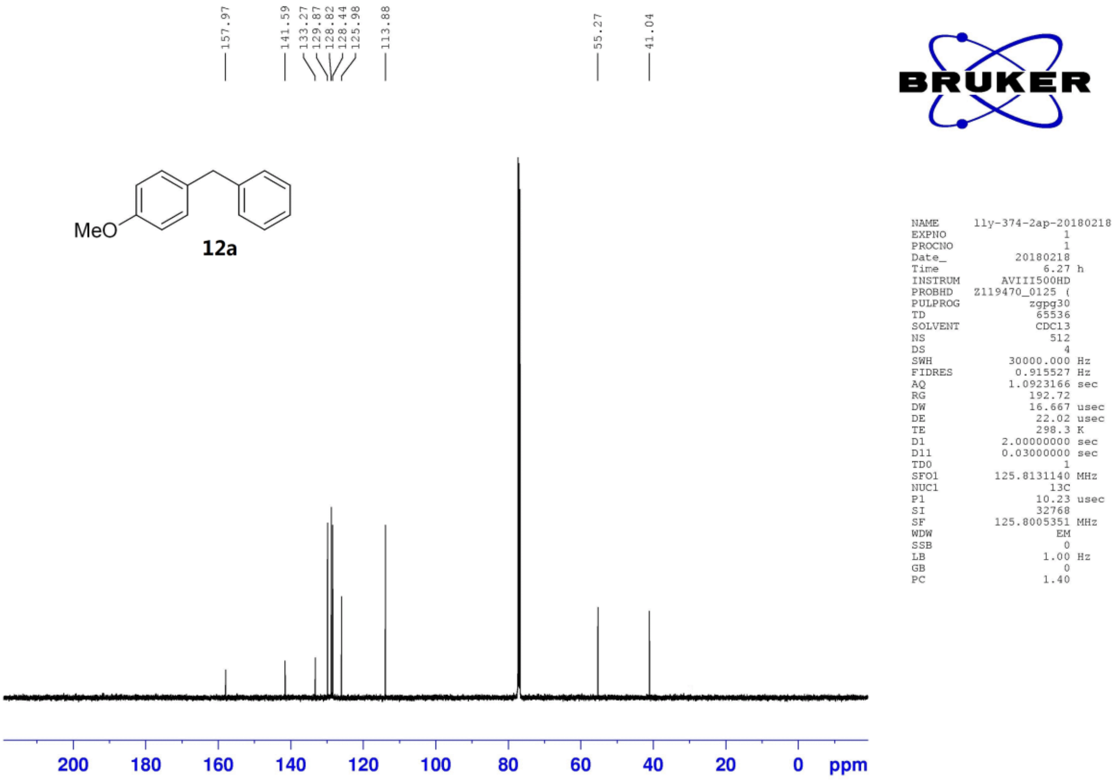

Supplementary Figure 141. ^{13}C NMR spectra for compound **12a**

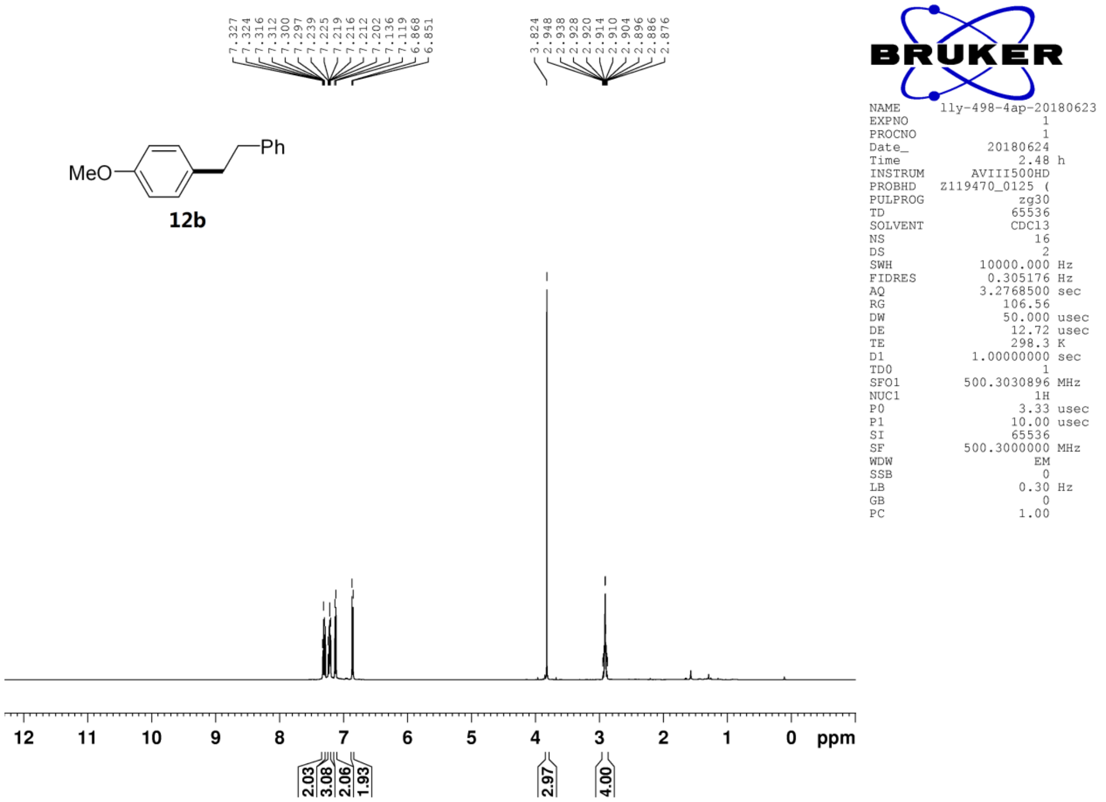

Supplementary Figure 142. ^{1}H NMR spectra for compound **12b**

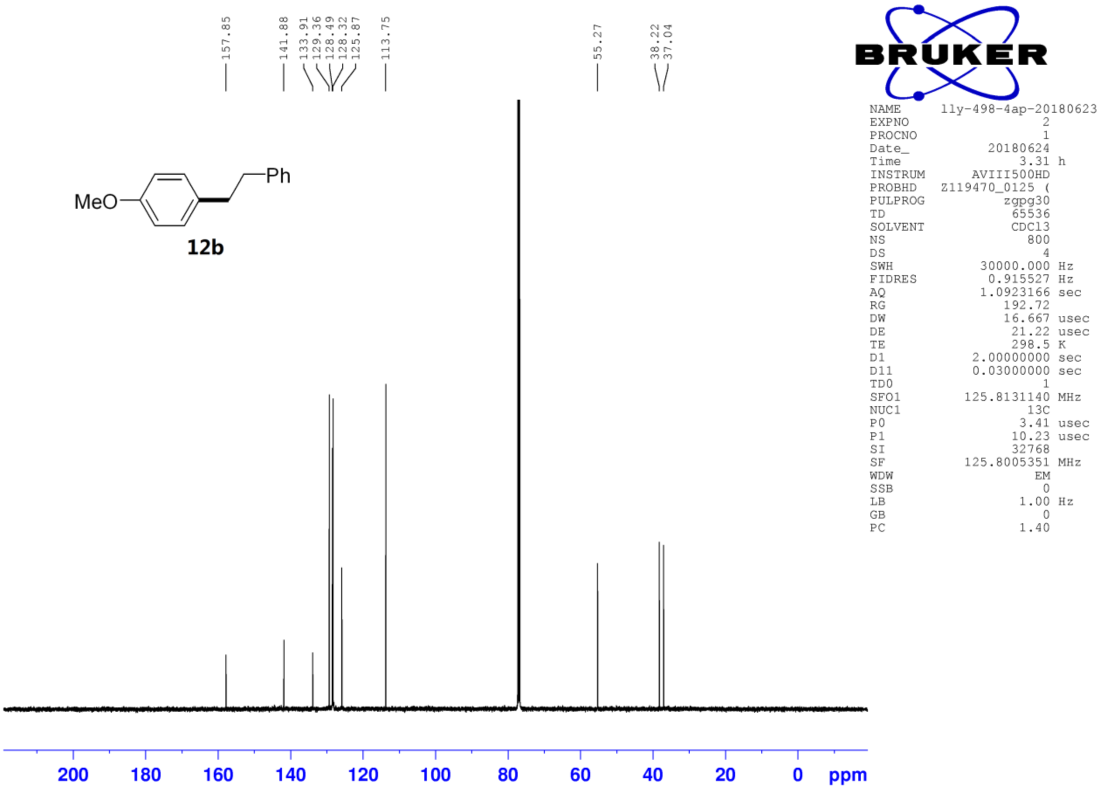

Supplementary Figure 143. ^{13}C NMR spectra for compound **12b**

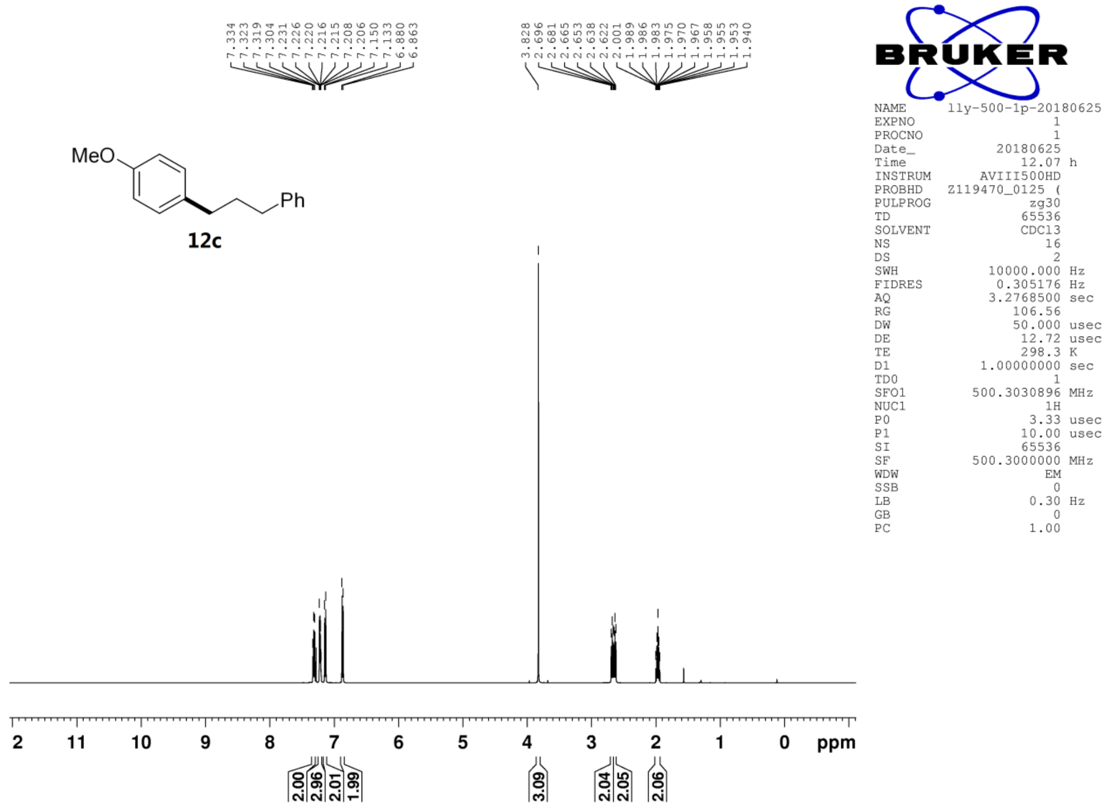

Supplementary Figure 144. ^{1}H NMR spectra for compound **12c**

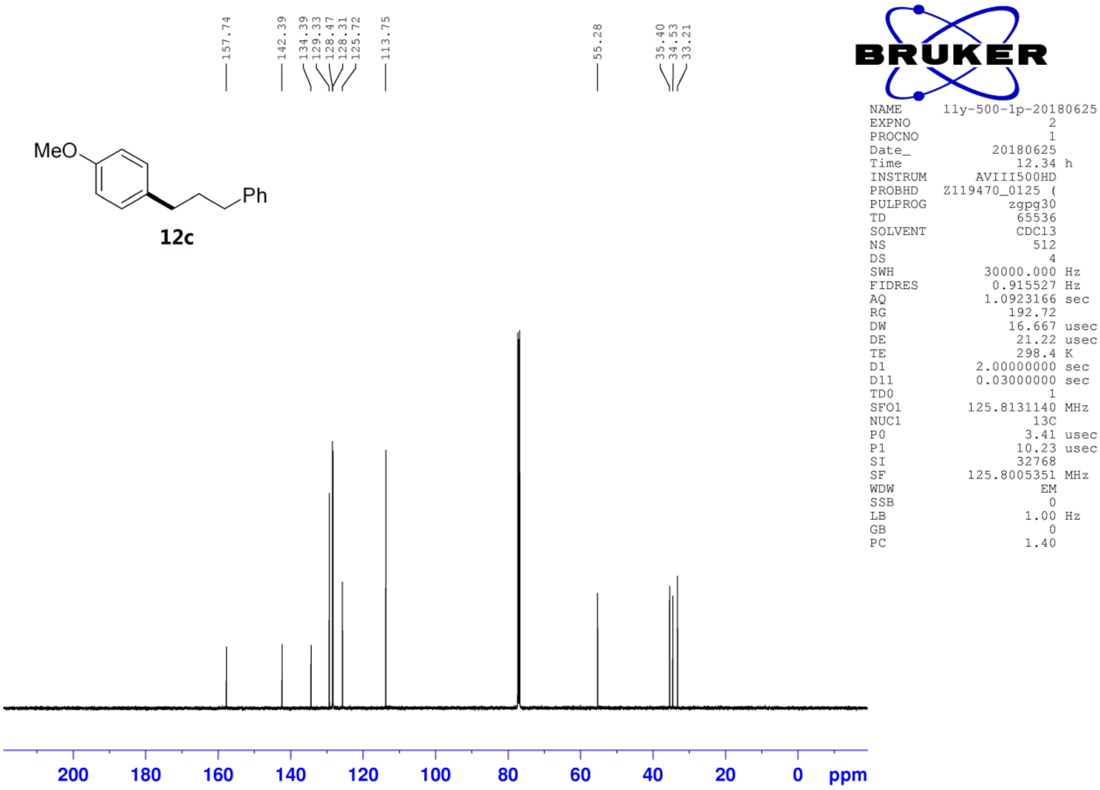

Supplementary Figure 145. ^{13}C NMR spectra for compound **12c**

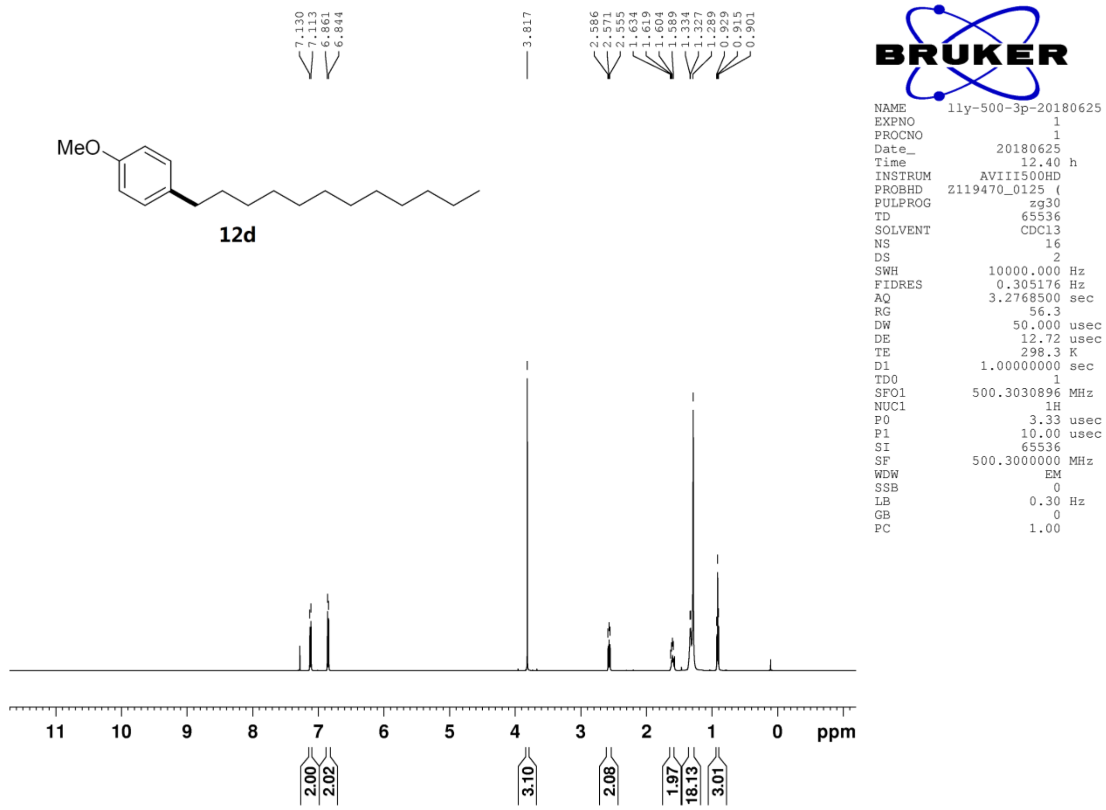

Supplementary Figure 146. ^{1}H NMR spectra for compound **12d**

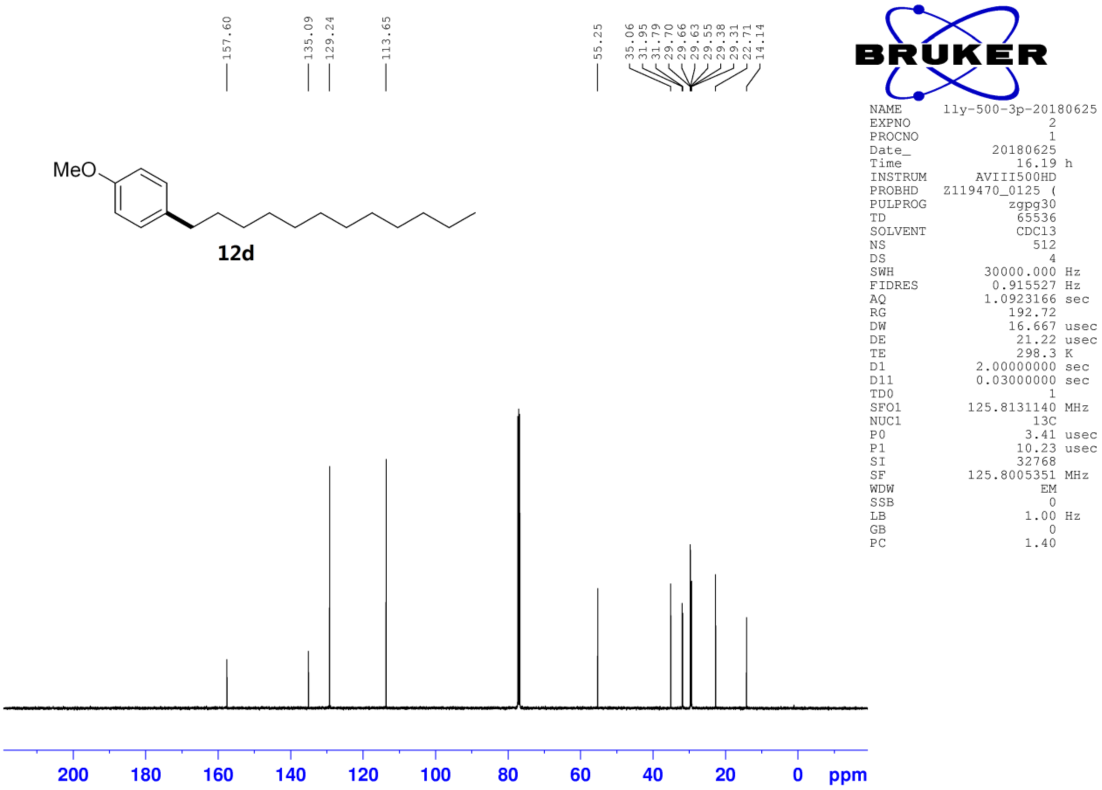

Supplementary Figure 147. ^{13}C NMR spectra for compound **12d**

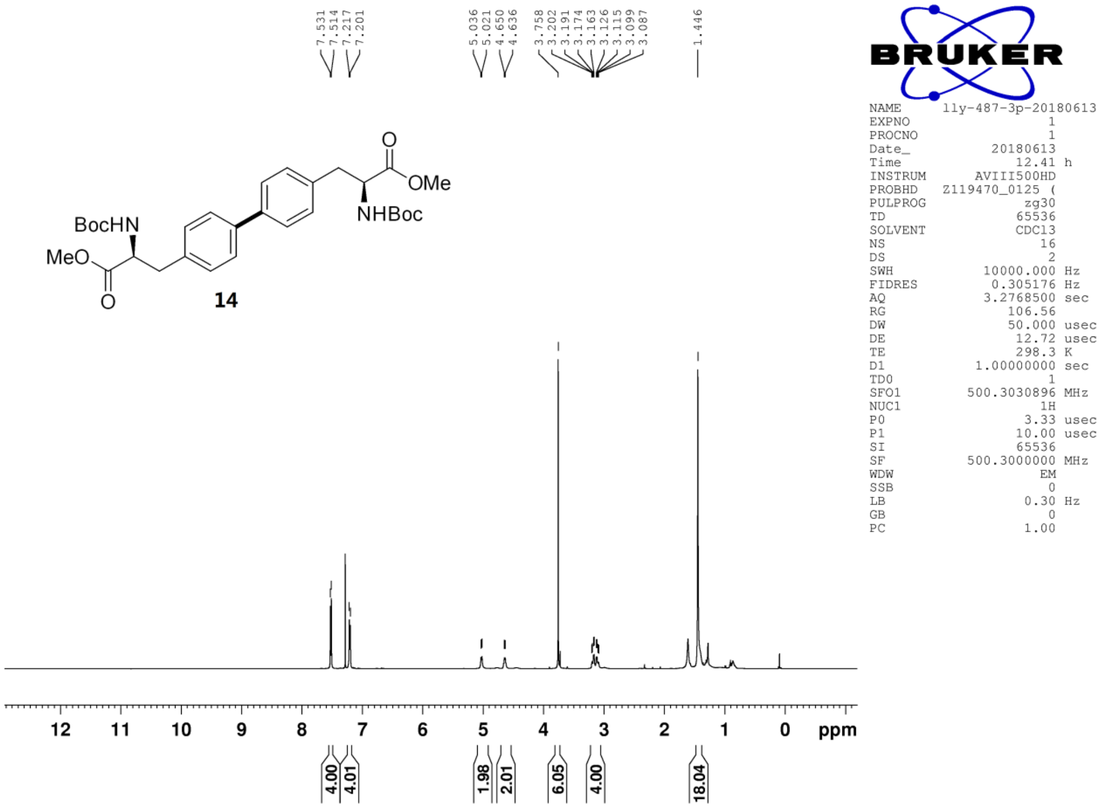

Supplementary Figure 148. ^{1}H NMR spectra for compound **14**

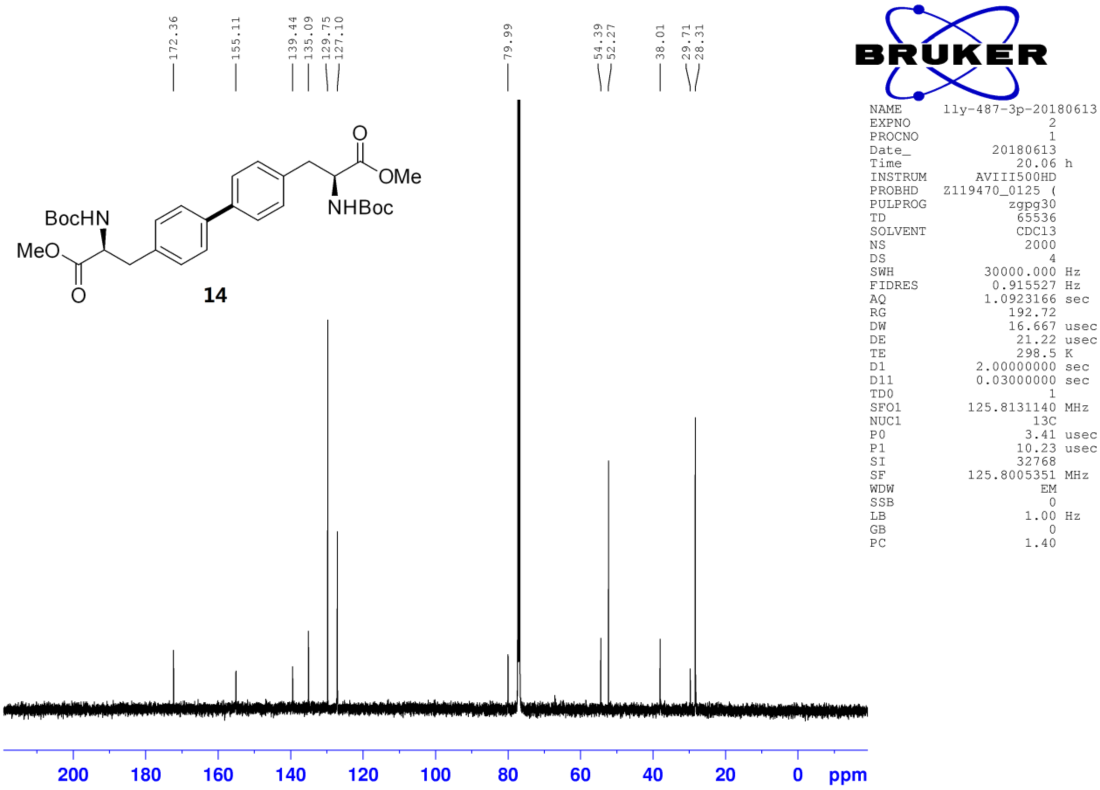

Supplementary Figure 149. ^{13}C NMR spectra for compound **14**

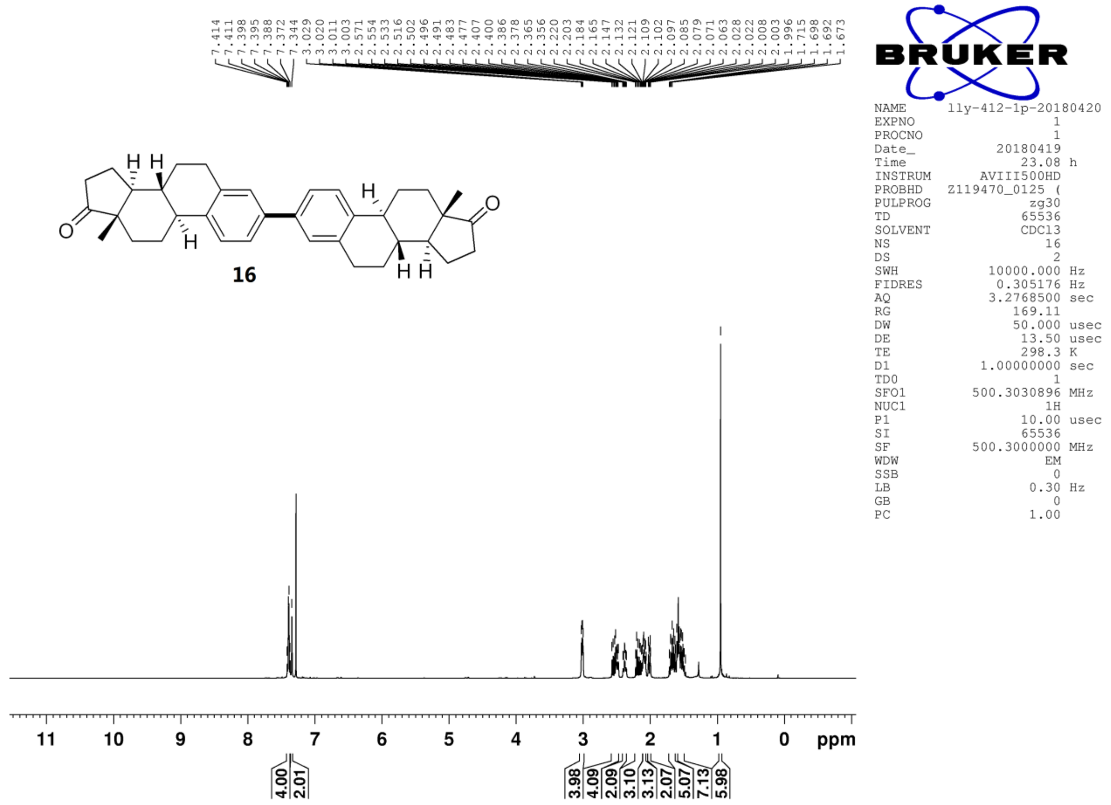

Supplementary Figure 150. ^{1}H NMR spectra for compound **16**

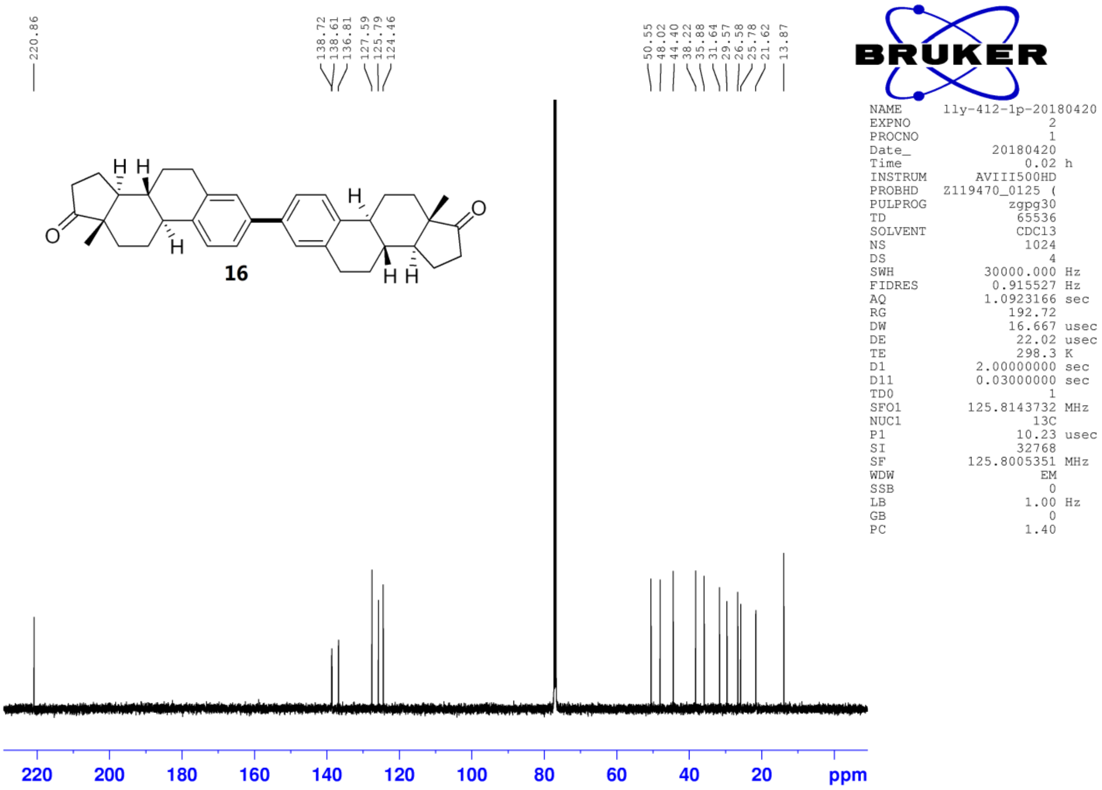

Supplementary Figure 151. ^{13}C NMR spectra for compound **16d**

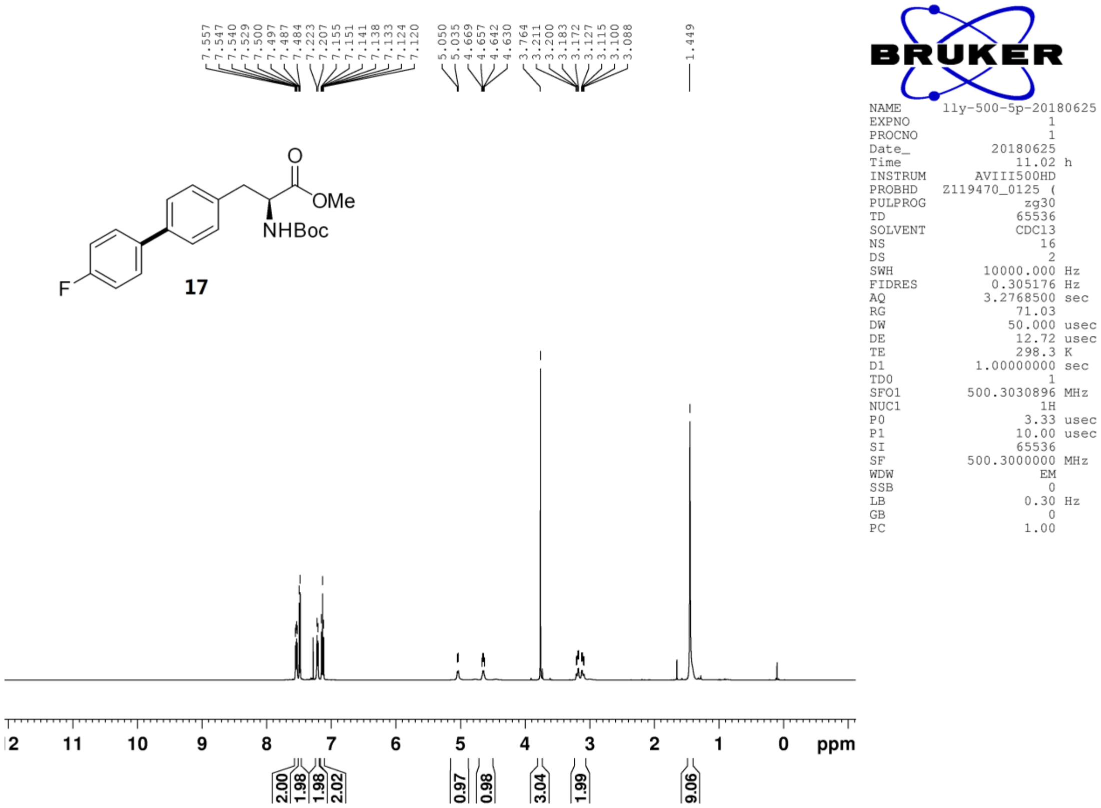

Supplementary Figure 152. ^{1}H NMR spectra for compound **17**

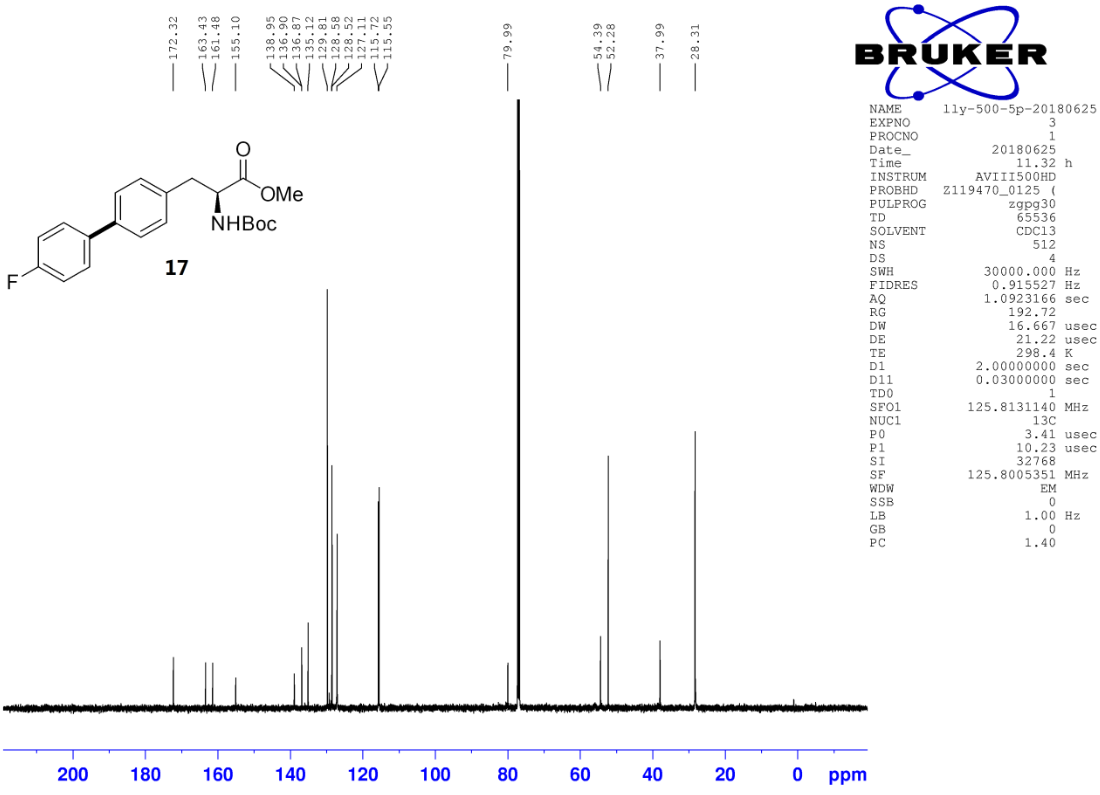

Supplementary Figure 153. ^{13}C NMR spectra for compound **17**

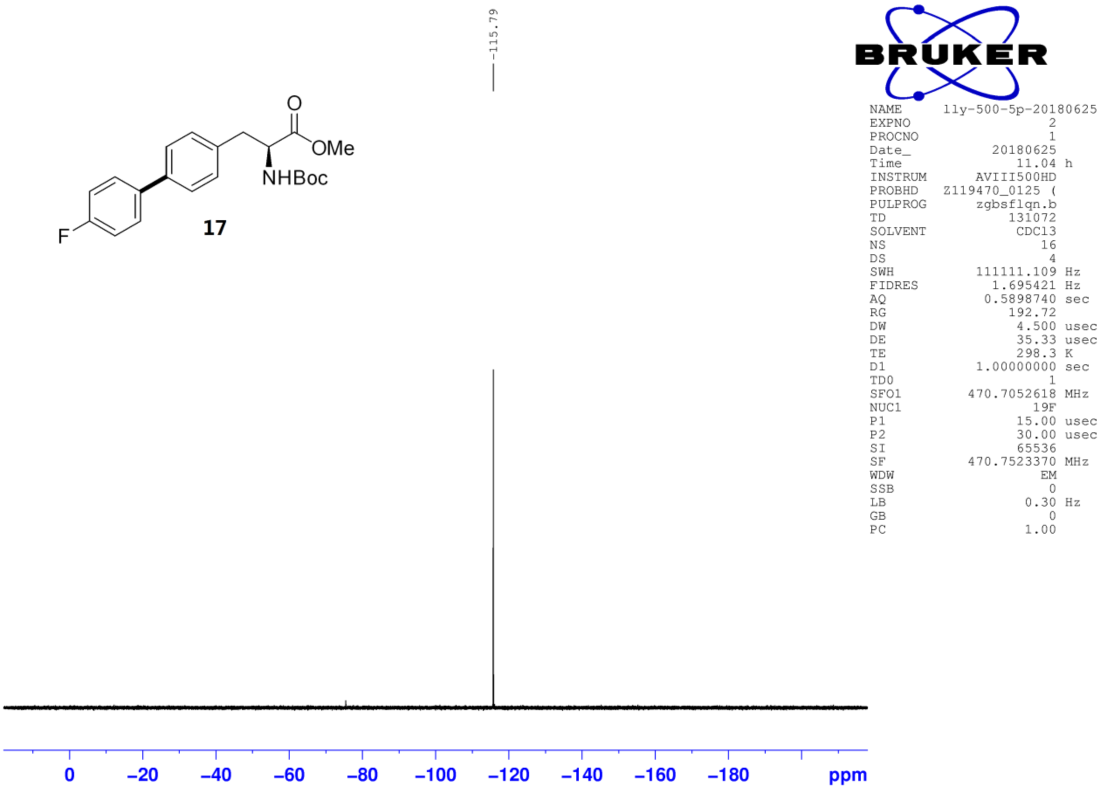

Supplementary Figure 154. ^{19}F NMR spectra for compound **17**

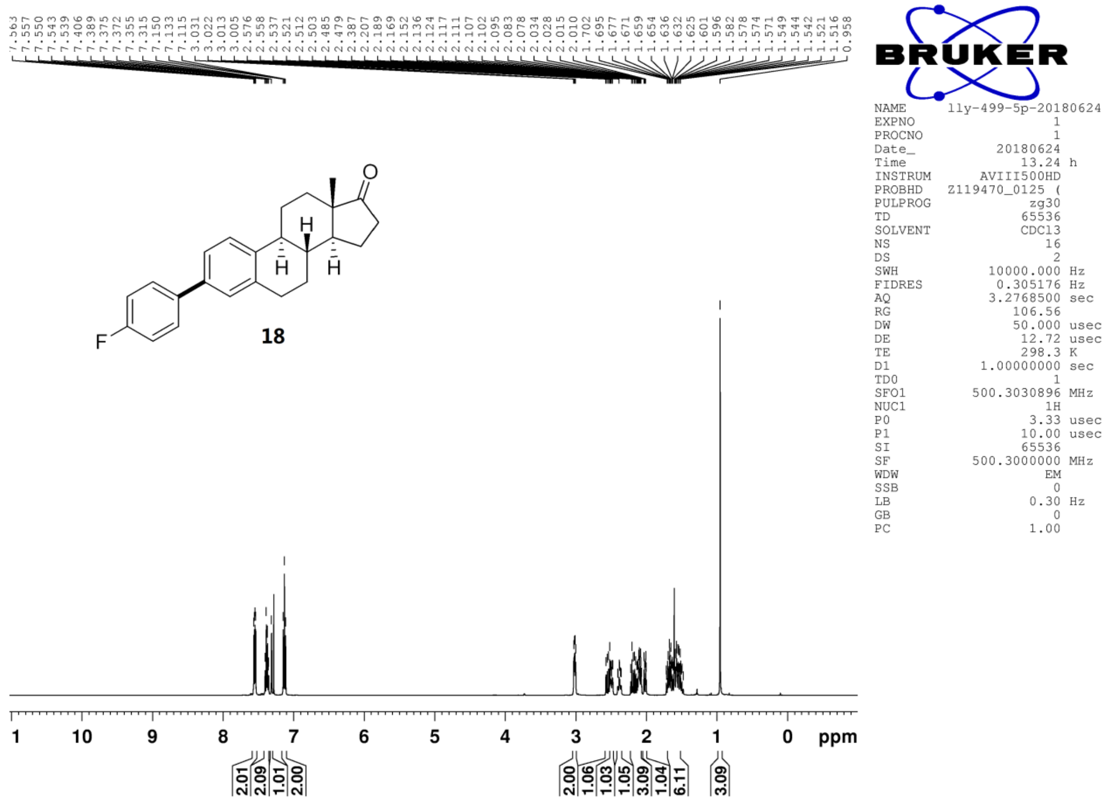

Supplementary Figure 155. ^{1}H NMR spectra for compound **18**

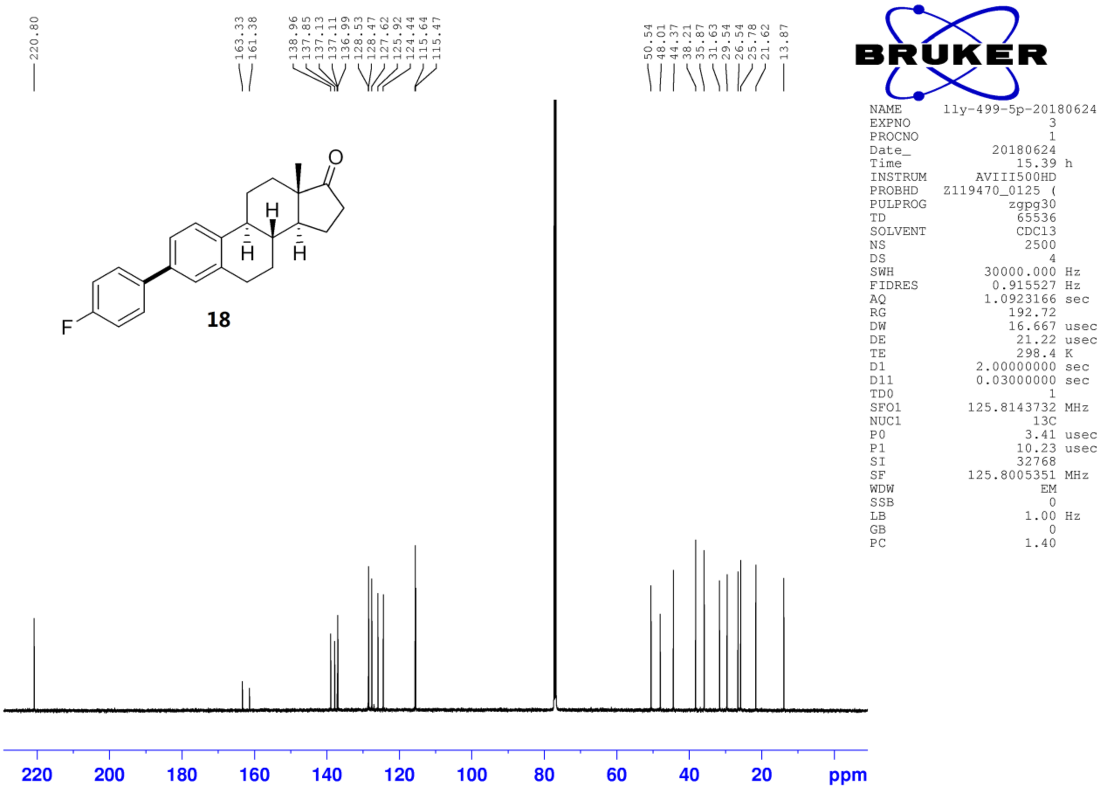

Supplementary Figure 156. ^{13}C NMR spectra for compound **18**

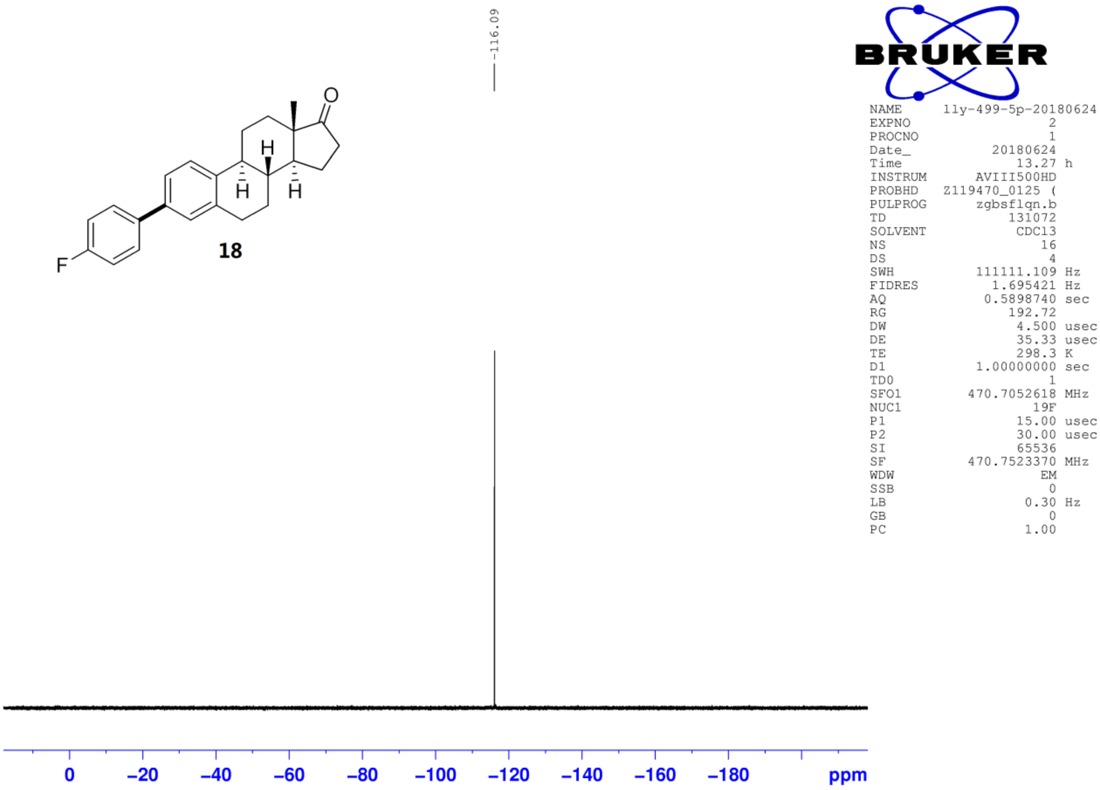

Supplementary Figure 157. ^{19}F NMR spectra for compound **18**

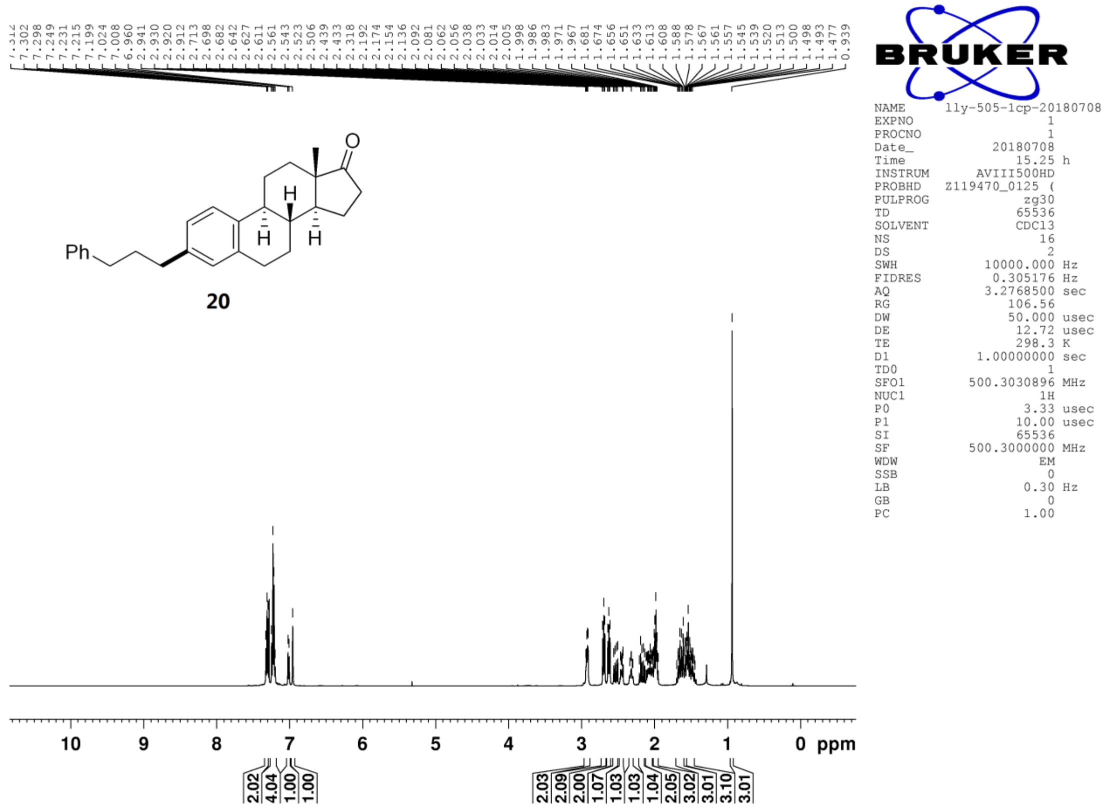

Supplementary Figure 158. ^{1}H NMR spectra for compound **20**

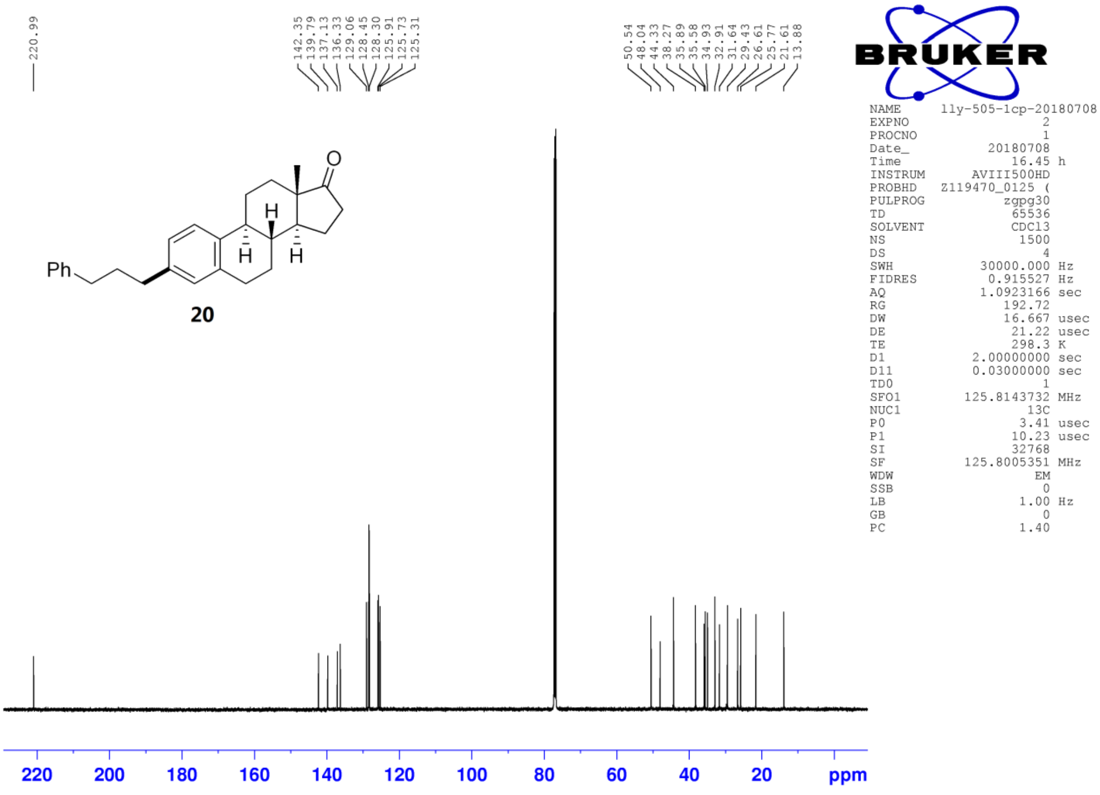

Supplementary Figure 159. ^{13}C NMR spectra for compound **20**

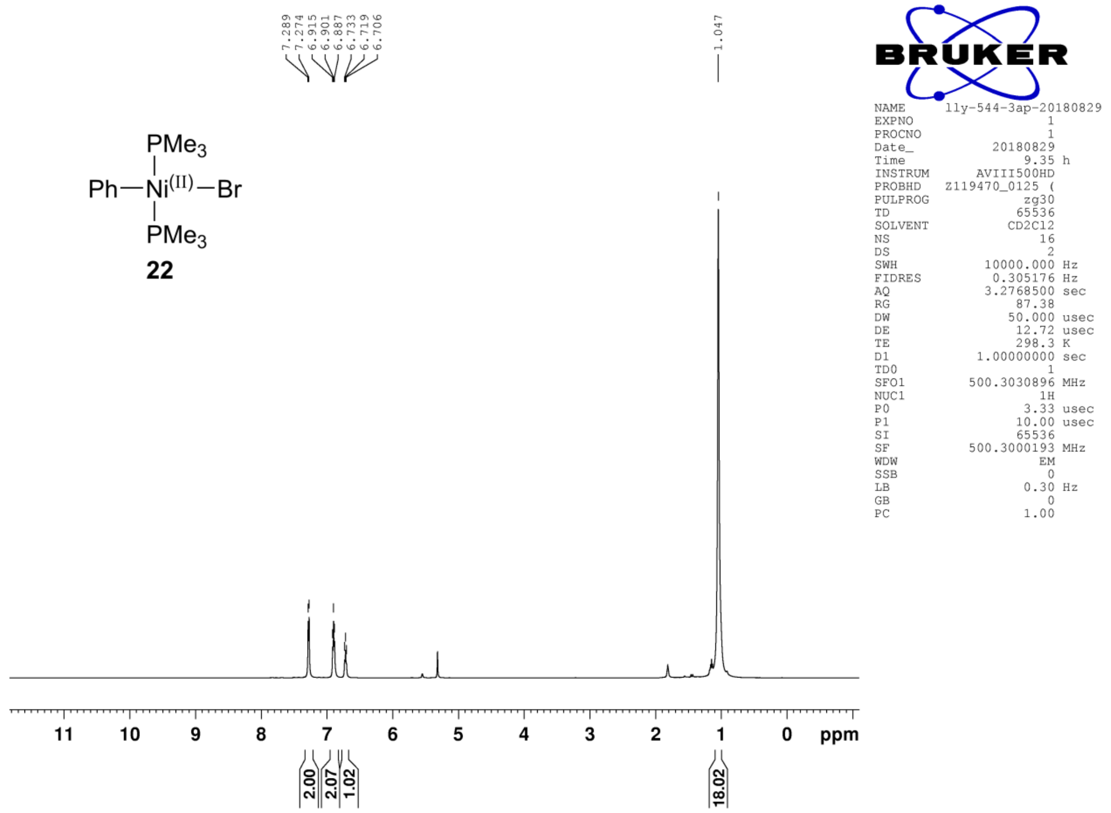

Supplementary Figure 160. ^{1}H NMR spectra for compound **22**

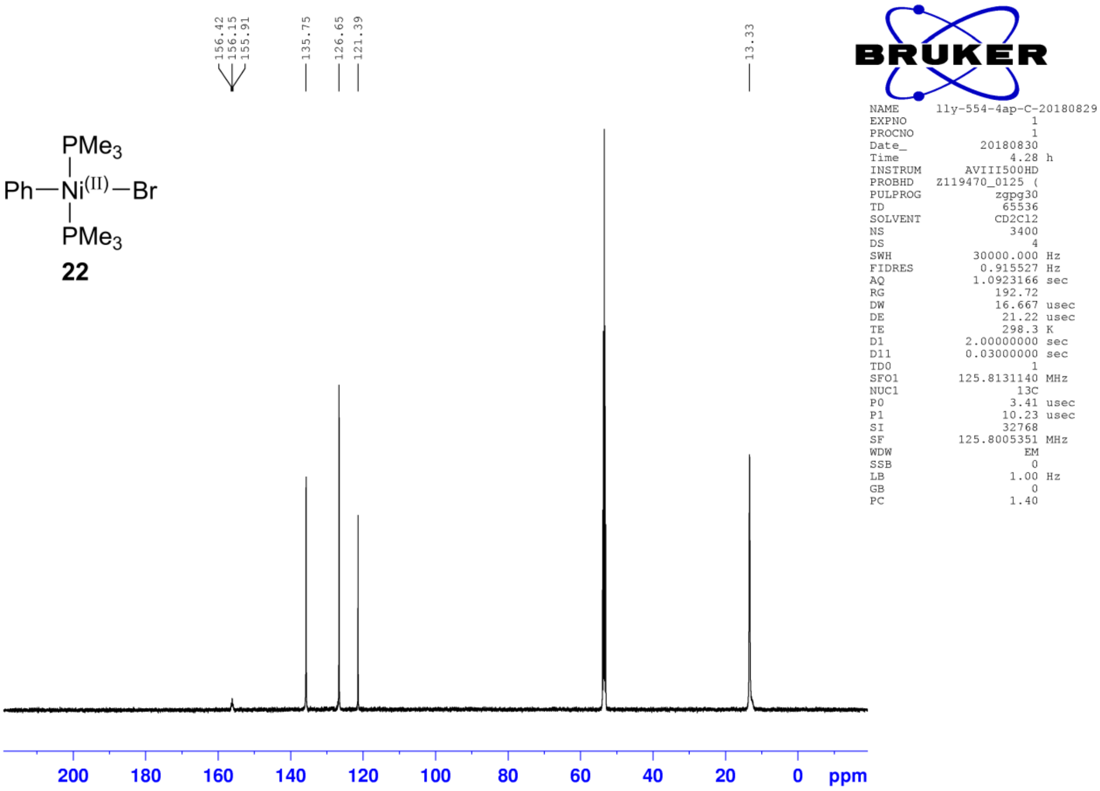

Supplementary Figure 161. ^{13}C NMR spectra for compound **22**

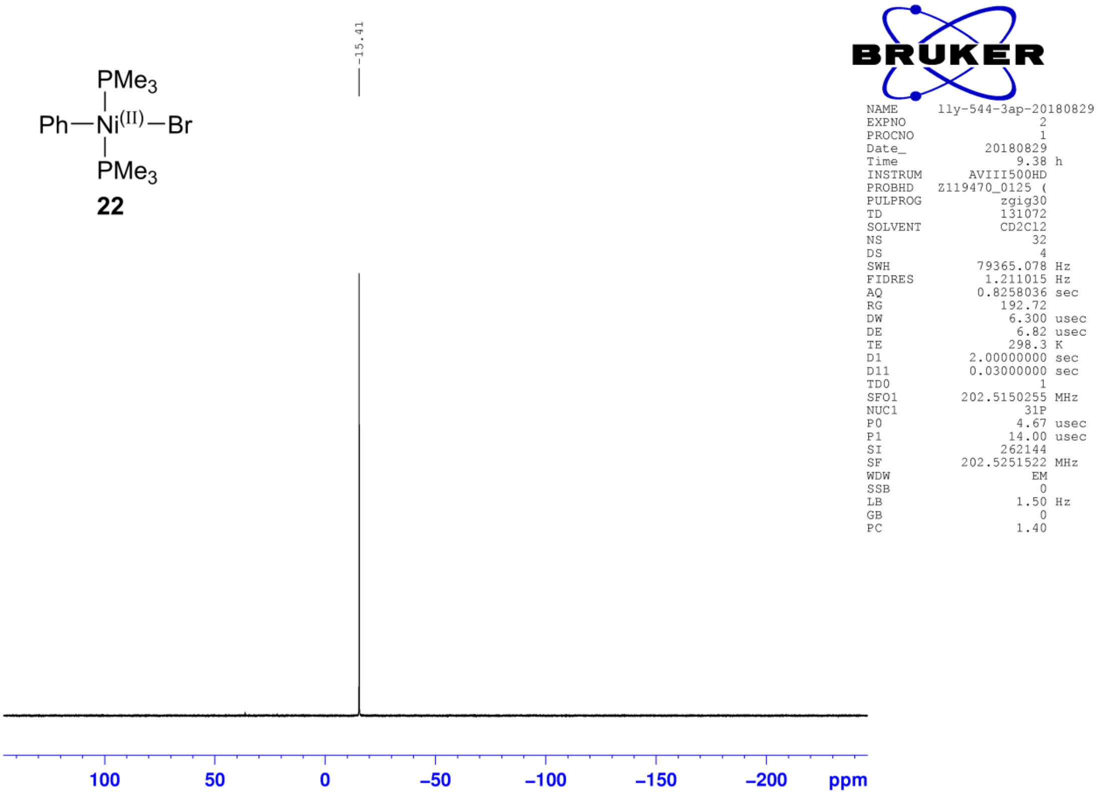

Supplementary Figure 162. ^{31}P NMR spectra for compound **22**

Supplementary References

[1] Dzhevakov, P. B., Topchiy, M. A., Zharkova, D. A., Morozov, O. S., Asachenko, A. F. & Nechaev, M. S. Miyaura Borylation and One-Pot Two-Step Homocoupling of Aryl Chlorides and Bromides under Solvent-Free Conditions. *Adv. Synth. Catal.* **358**, 977-983 (2016).

[2] Chen, S.-Y., Zhang, J., Li, Y.-H., Wen, J., Bian, S.-Q. & Yu, X.-Q. Cobalt-Catalyzed Homo-Coupling of Aryl and Alkenyl Bromide Using Atmospheric Oxygen as Oxidant. *Tetrahedron Lett*. **50**, 6795-6797 (2009).

[3] Kobayashi, T.-A., Abe, F. & Tanaka, M. Palladium Complex-Catalyzed Carboalkoxylation of Bis(chloromethyl)arenes. *J. Mol. Catal*. **45**, 91-109 (1988).

[4] Yao, P. Pd-Catalyzed Homocoupling of Arylhydrazines via C-N Cleavage Under O_2. *Appl. Organomet. Chem.* **28**, 194-197 (2014).

[5] Toummini, D., Ouazzani, F. & Taillefer, M. Iron-Catalyzed Homocoupling of Aryl Halides and Derivatives in the Presence of Alkyllithiums. *Org. Lett*. **15**, 4690-4693 (2013).

[6] Peng, Z., Li, N., Sun, X., Wang, F., Xu, L., Jiang, C., Song, L. & Yan, Z.-F. The Transition-Metal-Catalyst-Free Oxidative Homocoupling of Organomanganese Reagents Prepared by the Insertion of Magnesium into Organic Halides in the Presence of $MnCl_2 \cdot 2LiCl$. *Org. Biomol. Chem*. **12**, 7800-7809 (2014).

[7] Nakamura, K., Tobisu, M. & Chatani, N. Nickel-Catalyzed Formal Homocoupling of Methoxyarenes for the Synthesis of Symmetrical Biaryls via C-O Bond Cleavage. *Org. Lett*. **17**, 6142-6145 (2015).

[8] Du, F., Zhou, Q., Liu, D., Fang, T., Shi, Y., Du, Y. & Chen, G. Dimerization of Aromatic Compounds Using Palladium-Carbon-Catalyzed Suzuki-Miyaura Cross-Coupling by One-Pot Synthesis. *Synlett* **29**, 779-784 (2018).

[9] Minisci, F., Recupero, F., Cecchetto, A., Gambarotti, C., Punta, C., Paganelli, R., Pedulli, G. F. & Fontana, F. Solvent and Temperature Effects in the Free Radical Aerobic Oxidation of Alkyl and Acyl Aromatics Catalysed by Transition Metal Salts and *N*-Hydroxyphthalimide: New Processes for the Synthesis of *p*-Hydroxybenzoic Acid, Diphenols, and Dienes for Liquid Crystals and Cross-Linked Polymers. *Org. Proc. Res. Dev*. **8**, 163-168 (2004).

[10] Masciocchi, N., Galli, S., Colombo, V., Maspero, A., Palmisano, G., Seyyedi, B., Lambert, C & Bordiga, S. Cubic Octanuclear Ni(II) Clusters in Highly Porous Polypyrazolyl-Based Materials. *J. Am. Chem. Soc*. **132**, 7902-7904 (2010).

[11] Krasovskiy, A., Tishkov, A., del Amo, V., Mayr, H & Knochel, P. Transition-Metal-Free Homocoupling of Organomagnesium Compounds. *Angew. Chem. Int. Ed*. **45**, 5010-5014 (2006).

[12] Gao, H., Zhou, Z., Kwon, D.-H., Coombs, J., Jones, S., Behnke, N. E., Ess, D. H. & Kürti, L. Rapid Heteroatom Transfer to Arylmetals Utilizing Multifunctional Reagent Scaffolds. *Nat. Chem*. **9**, 681-688 (2017).

[13] Kawashima, T., Takao, T. & Suzuki, H. Dehydrogenative Coupling of 4-Substituted Pyridines Catalyzed by Diruthenium Complexes. *J. Am. Chem. Soc*. **129**, 11006-11007 (2007).

[14] Rahil, R., Sengmany, S., Le Gall, E. & Léonel, E. Nickel-Catalyzed Electrochemical Reductive Homocouplings of Aryl and Heteroaryl Halides: A Useful Route to Symmetrical Biaryls. *Synthesis* **50**, 146-154 (2018).

[15] Skórka, Ł., Filapek, M., Zur, L., Małecki, J. G., Pisarski, W., Olejnik, M., Danikiewicz, W., Krompiec, S. Highly Phosphorescent Cyclometalated Iridium(III) Complexes for Optoelectronic Applications: Fine Tuning of the Emission Wavelength through Ancillary Ligands. *J. Phys. Chem. C*. **120,** 7284-7294 (2016).

[16] Dai, X.-J & Li, C.-J. En Route to a Practical Primary Alcohol Deoxygenation. *J. Am. Chem. Soc*. **138**, 5433-5440 (2016).

[17] Cahiez, G., Chaboche, C., Mahuteau-Betzer, F. & Ahr, M. Iron-Catalyzed Homo-Coupling of Simple and Functionalized Arylmagnesium Reagents. *Org. Lett.* **7**, 1943-1946 (2005).

[18] Mandelbaum, A. & Cais, M. Polycyclic Studies. I. Synthesis of Triphenylenes Through Diels-Alder Adducts. *J. Org. Chem*. **26**, 2633-2640 (1961).

[19] Shirakawa, E., Sato, T., Imazaki, Y., Kimura, T. & Hayashi, T. *Chem. Commun*. **0**, 4513-4515 (2007).

[20] Erb, W., Albini, M., Rouden, J. & Blanchet, J. Sequential One-Pot Access to Molecular Diversity through Aniline Aqueous Borylation. *J. Org. Chem.* **79**, 10568-10580 (2014).

[21] Soulé, J.-F., Miyamura, H. & Kobayashi, S. Copolymer-Incarcerated Nickel Nanoparticles with N-Heterocyclic Carbene Precursors as Active Cross-Linking Agents for Corriu-Kumada-Tamao Reaction. *J. Am. Chem. Soc.* **135**, 10602-10605 (2013).

[22] Ackerman, L. K. G., Lovell, M. M. & Weix, D. J. Multimetallic Catalyzed Cross-Coupling of Aryl Bromides with Aryl Triflates. *Nature* **524**, 454-457 (2015).

[23] Muto, K., Yamaguchi, J., Musaev, D. G. & Itami, K. Decarbonylative Organoboron Cross-Coupling of Esters by Nickel Catalysis. *Nat. Commun.* **6,** 7508-7515 (2015).

[24] Lv, L., Zhu, D., Tang, J., Qiu, Z., Li, C.-C., Gao, J. & Li, C.-J. Cross-Coupling of Phenol Derivatives with Umpolung Aldehydes Catalyzed by Nickel. *ACS Catal*. **8**, 4622-4627 (2018).

[25] Ding, S., Xu, L. & Li, P. Copper-Catalyzed Boron-Selective C(sp^2)-C(sp^3) Oxidative Cross-Coupling of Arylboronic Acids and Alkyltrifluoroborates Involving a Single-Electron Transmetalation Process. *ACS Catal*. **6**, 1329-1333 (2016).

[26] Dupuy, S., Zhang, K.-F., Goutierre, A.-S. & Baudoin, O. Terminal-Selective Functionalization of Alkyl Chains by Regioconvergent Cross-Coupling. *Angew. Chem. Int. Ed*. **55**, 14793-14797 (2016).

[27] Carmona, E., Paneque, M. & Poveda, M. L. Synthesis and Characterization of Some New Organometallic Complexes of Nickel(II) Containing Trimethylphosphine. *Polyhedron* **8**, 285-291 (1989).

[28] Percec, V., Bae, J.-Y., Zhao, M. & Hill, D. H. Aryl Mesylates in Metal-Catalyzed Homo-Coupling and Cross-Coupling Reactions. 1. Functional Symmetrical Biaryls from Phenols via Nickel-Catalyzed Homo-Coupling of Their Mesylates. *J. Org. Chem.* **60**, 176-185 (1995).
